# Health effects associated with exposure to secondhand smoke: a Burden of Proof study

**DOI:** 10.1038/s41591-023-02743-4

**Published:** 2024-01-09

**Authors:** Luisa S. Flor, Jason A. Anderson, Noah Ahmad, Aleksandr Aravkin, Sinclair Carr, Xiaochen Dai, Gabriela F. Gil, Simon I. Hay, Matthew J. Malloy, Susan A. McLaughlin, Erin C. Mullany, Christopher J. L. Murray, Erin M. O’Connell, Chukwuma Okereke, Reed J. D. Sorensen, Joanna Whisnant, Peng Zheng, Emmanuela Gakidou

**Affiliations:** 1 Institute for Health Metrics and Evaluation, University of Washington, Seattle, WA USA; 2 Department of Health Metrics Sciences, School of Medicine, University of Washington, Seattle, WA USA; 3 Department of Global Health, University of Washington, Seattle, WA USA

**Keywords:** Risk factors, Diseases

## Abstract

Despite a gradual decline in smoking rates over time, exposure to secondhand smoke (SHS) continues to cause harm to nonsmokers, who are disproportionately children and women living in low- and middle-income countries. We comprehensively reviewed the literature published by July 2022 concerning the adverse impacts of SHS exposure on nine health outcomes. Following, we quantified each exposure–response association accounting for various sources of uncertainty and evaluated the strength of the evidence supporting our analyses using the Burden of Proof Risk Function methodology. We found all nine health outcomes to be associated with SHS exposure. We conservatively estimated that SHS increases the risk of ischemic heart disease, stroke, type 2 diabetes and lung cancer by at least around 8%, 5%, 1% and 1%, respectively, with the evidence supporting these harmful associations rated as weak (two stars). The evidence supporting the harmful associations between SHS and otitis media, asthma, lower respiratory infections, breast cancer and chronic obstructive pulmonary disease was weaker (one star). Despite the weak underlying evidence for these associations, our results reinforce the harmful effects of SHS on health and the need to prioritize advancing efforts to reduce active and passive smoking through a combination of public health policies and education initiatives.

## Abstract (web summary)

A meta-analysis using the Burden of Proof method reported consistent evidence supporting harmful associations between exposures to secondhand smoke and nine health outcomes.

## Main

Tobacco use is one of the leading risk factors for disease burden and mortality worldwide, contributing to 229.8 million (95% uncertainty interval: 213.1–246.4 million) disability-adjusted life years and 8.7 million (8.1–9.3 million) deaths in 2019 (ref. ^1^). Secondhand smoke (SHS) exposure, alternatively referred to as passive or involuntary smoking, is a major tobacco-related public health concern for nonsmokers. Despite a gradual decline in smoking rates over the past half-century^2^, it is estimated that approximately 37% of the global population is still exposed to the smoke emitted from the burning end of tobacco products or exhaled from smokers, with higher rates of exposure among women and children compared to men, and evident racial and economic disparities^3,4^. This is concerning as tobacco smoke is composed of thousands of chemicals and compounds, including many carcinogens, which when inhaled damage the human body and lead to disease and death^5^.

The 2019 Global Burden of Diseases, Injuries, and Risk Factors Study (GBD) estimated that 1.3 million (1.0–1.6) deaths were attributable to SHS globally in 2019, with the largest burden concentrated in low- and middle-income countries^6^. These patterns have made SHS a priority for tobacco control efforts, especially after the adoption of the World Health Organization’s Framework Convention on Tobacco Control, a global treaty aimed at implementing evidence-based measures to reduce both active and passive smoking^7^. Therefore, providing an updated summary of the exposure–response relationship between SHS and multiple adverse health outcomes, as well as innovatively quantifying the strength of the evidence supporting these relationships, is essential to continue to inform tobacco control policy, research funders and clinical recommendations and guide individual decisions related to smoking practices.

Over time, advances in understanding the harms of SHS have raised awareness of the importance of protecting nonsmokers from tobacco smoke. Smoke-free initiatives, in particular, have changed attitudes and social norms toward SHS exposure and have been a key contributor to the decline of smoking prevalence^8^. Nevertheless, as world populations grow, the number of smokers continues to rise, increasing the number of nonsmokers at risk of SHS exposure^9^.

Over the past decades, the body of evidence concerning the relationship between SHS and health has greatly evolved with the outline of plausible biological mechanisms and in-depth consideration of the available evidence, moving from the first reported association with lung cancer in the 1986 Surgeon Generals’ report^10^ to the inference of causal relationships between SHS and a range of diseases affecting and adverse health outcomes for adults and children, including cardiovascular diseases, some respiratory illnesses, middle ear disease, low birth weight and sudden infant death syndrome^11,12^. Additionally, previous research, including meta-analyses, found suggestive evidence of an association between SHS exposure and breast cancer^13–15^. Despite these findings, substantial heterogeneity is detected across and within SHS risk–outcome assessments in terms of quantity and quality of studies and reported strength of associations. Variation across studies in the definitions of risk exposure used is also observed, with some reporting the risk associated with SHS exposure in specific settings^16^ or from specific sources (that is, maternal, paternal)^17^. Furthermore, given the limited availability of studies that assess exposure to tobacco smoke on the basis of environmental and biological samples, and the lack of a standard measure of SHS exposure, the units and dose categories reported across studies vary widely. Together, these inconsistencies can limit the comparability and consolidation of evidence concerning the health effects of SHS.

In this context, in this Article, we aimed to quantify the exposure–response associations between SHS and nine health outcomes—lung and breast cancer, ischemic heart disease (IHD), stroke, chronic obstructive pulmonary disease (COPD), lower respiratory infections, asthma, type 2 diabetes and otitis media—as well as the strength of the available evidence, using an objective, comprehensive and comparative framework. The Burden of Proof Risk Function (BPRF) derives a conservative estimate of the smallest harmful effects of SHS exposure on given health outcomes that are consistent with the available evidence and to summarize the strength of risk–outcome associations and their underlying evidence into a star-rating measure, ranging from one star (weak evidence of an association) to five stars (consistent evidence of a strong association), to aid the interpretation and comparability of results^18^. The main findings and policy implications of this work are summarized in Table 1.

**Table 1:** Policy summary

Background	Although smoking rates have declined globally, SHS is a major public health issue—with over one-third of the world’s population exposed and health effects disproportionately borne by women, children and people in low- and middle-income countries. Comparability across SHS–response associations is constrained by considerable variability across exposure definitions and measurement, study design and results. In the present meta-analysis, we systematically applied the Burden of Proof methodology to synthesize evidence investigating the association between SHS and nine outcomes related to cardiovascular disease, neoplasms and respiratory conditions—in addition to type 2 diabetes and otitis media.
Main findings and limitations	We found statistically significant associations between SHS and all nine outcomes examined, suggesting that SHS exposure is irrefutably harmful to human health. When incorporating measures of known and unexplained between-study heterogeneity to generate conservative estimates of SHS-related health risk consistent with available evidence, the strongest relationships were seen for cardiovascular conditions that include IHD and stroke (the two major causes of disease burden worldwide), along with type 2 diabetes and lung cancer; for these four outcomes, SHS exposure was conservatively estimated to increase disease risk by at least around 8%, 5%, 1% and 1%, respectively. The strength of the evidence on the relationship between SHS and breast cancer, COPD, lower respiratory infections, asthma and otitis media is rated as weak, and can benefit from additional higher-quality studies. Inconsistencies in case definitions and exposure measures and definitions used in the input data may limit the accuracy and generalizability of our findings. Moreover, to standardize results across studies, we modeled SHS exposure as a dichotomous variable, which may have oversimplified SHS risk profiles by discounting effects related to intensity and frequency of exposure. Additionally, the nine disease outcomes we investigated are unlikely to capture the full disease burden associated with SHS exposure.
Policy implications	Our meta-analysis of attributable health risks experienced by nonsmokers exposed to SHS suggests that SHS should be an area of concern for policymakers, health professionals and individual citizens. Although some of the SHS–disease associations we estimated were relatively weak, this is due in part to inconsistencies in methods and results across input studies. Moreover, the relatively high prevalence of SHS—and of the disease outcomes it is associated with—magnifies the need to prioritize reducing SHS exposure through a combination of public health policies and education initiatives. In addition to supporting strategies that promote active smoking cessation and noninitiation, it is essential to continue enacting, implementing and enforcing laws that establish smoke-free public areas. It is likewise imperative to raise awareness of the adverse consequences of SHS exposure in order to promote voluntary smoking restrictions in private homes, where women and children are disproportionately affected.

## Results

### Overview

**Extended Data Fig. 1:**
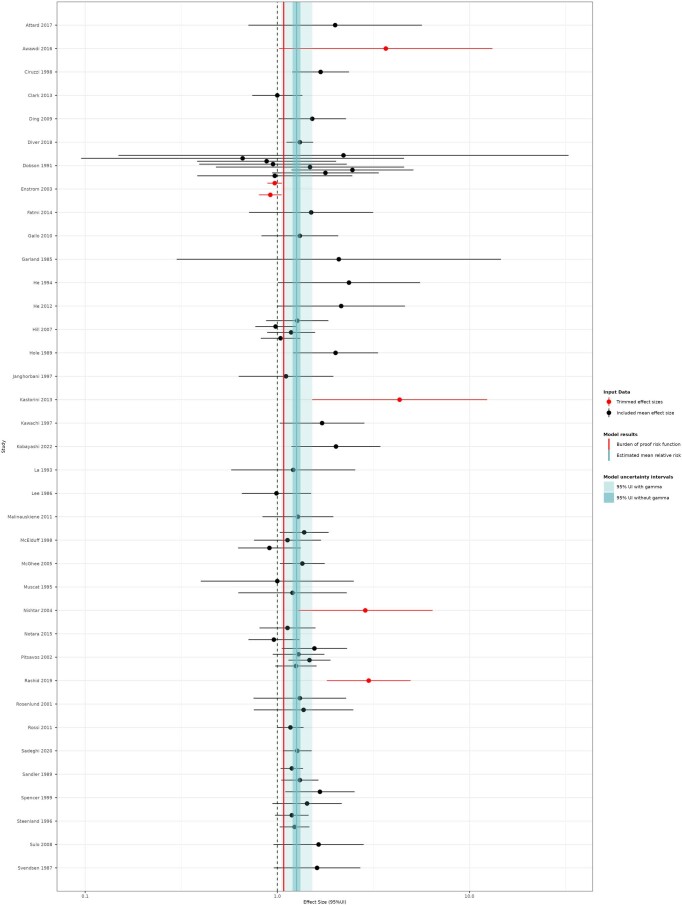
Forest plot of the association between secondhand smoke exposure and ischemic heart disease. This forest plot presents the estimated mean relative risk, its 95% uncertainty intervals (UI), and the data points underlying the estimates for ischemic heart disease in association with secondhand smoke exposure (two-star rating of the risk-outcome relationship). The color of the point indicates whether the point was detected and trimmed as an outlier. The light blue interval corresponds to the 95% UI incorporating between-study heterogeneity; the dark blue interval corresponds to the 95% UI without between-study heterogeneity. The black vertical dotted line reflects the null relative risk value (one) and the red vertical line is the burden of proof function at the 5th quantile for this harmful risk-outcome association. The black data points and horizontal lines each correspond to a mean effect size and 95% UI from the included study identified on the y-axis. We included multiple observations from a single study when effects were reported by location or source of exposure and/or separately by sex or other subgroups. See Supplementary Table 4 for more details on included observations from each study (n = 37 studies).

**Extended Data Fig. 2:**
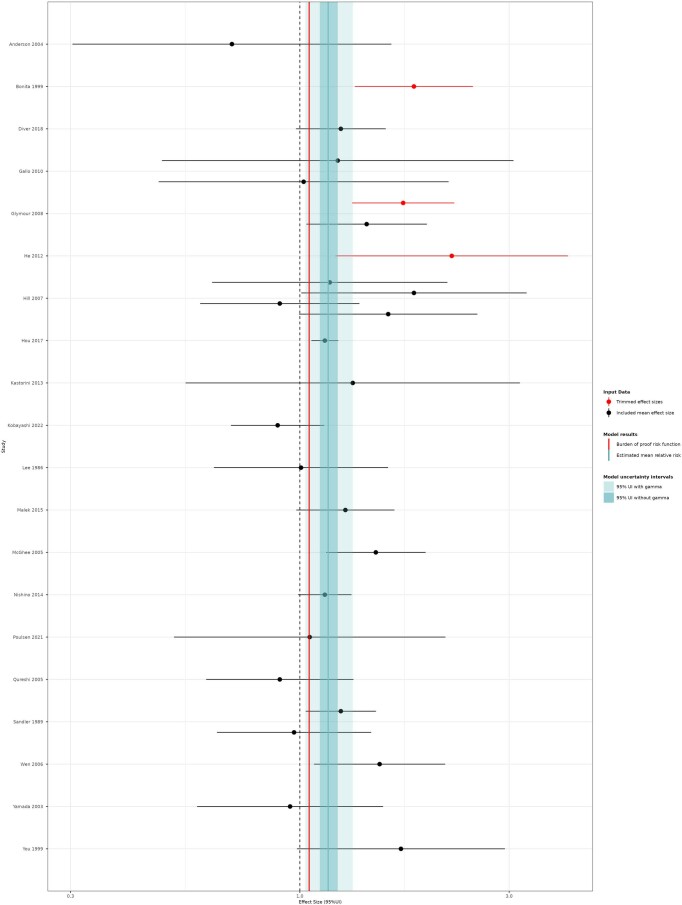
Forest plot of the association between secondhand smoke exposure and stroke. This forest plot presents the estimated mean relative risk, its 95% uncertainty intervals (UI), and the data points underlying the estimates for ischemic heart disease in association with secondhand smoke exposure (two-star rating of the risk-outcome relationship). The color of the point indicates whether the point was detected and trimmed as an outlier. The light blue interval corresponds to the 95% UI incorporating between-study heterogeneity; the dark blue interval corresponds to the 95% UI without between-study heterogeneity. The black vertical dotted line reflects the null relative risk value (one) and the red vertical line is the burden of proof function at the 5th quantile for this harmful risk-outcome association. The black data points and horizontal lines each correspond to a mean effect size and 95% UI from the included study identified on the y-axis. We included multiple observations from a single study when effects were reported by location or source of exposure and/or separately by sex or other subgroups. See Supplementary Table 5 for more details on included observations from each study (n = 20 studies).

**Extended Data Fig. 3:**
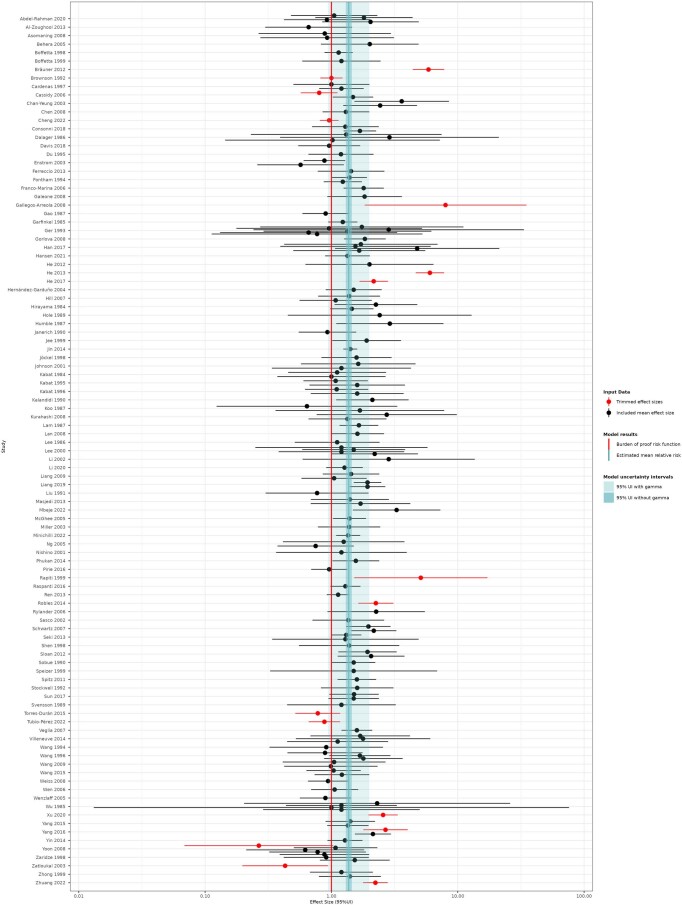
Forest plot of the association between secondhand smoke exposure and lung cancer. This forest plot presents the estimated mean relative risk, its 95% uncertainty intervals (UI), and the data points underlying the estimates for ischemic heart disease in association with secondhand smoke exposure (two-star rating of the risk-outcome relationship). The color of the point indicates whether the point was detected and trimmed as an outlier. The light blue interval corresponds to the 95% UI incorporating between-study heterogeneity; the dark blue interval corresponds to the 95% UI without between-study heterogeneity. The black vertical dotted line reflects the null relative risk value (one) and the red vertical line is the burden of proof function at the 5th quantile for this harmful risk-outcome association. The black data points and horizontal lines each correspond to a mean effect size and 95% UI from the included study identified on the y-axis. We included multiple observations from a single study when effects were reported by location or source of exposure and/or separately by sex or other subgroups. See Supplementary Table 6 for more details on included observations from each study (n = 104 studies).

**Extended Data Fig. 4:**
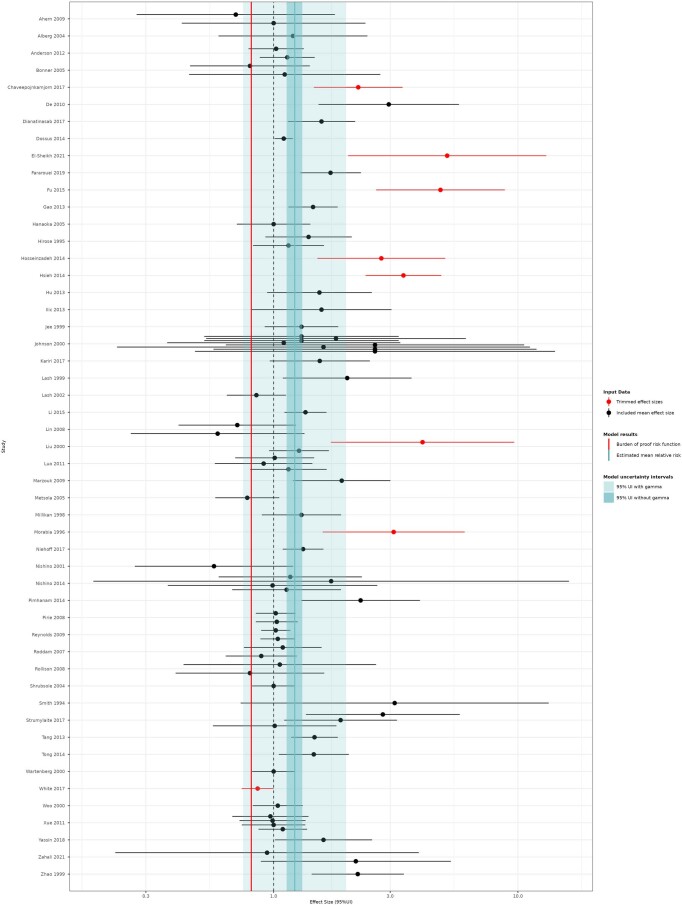
Forest plot of the association between secondhand smoke exposure and breast cancer. This forest plot presents the estimated mean relative risk, its 95% uncertainty intervals (UI), and the data points underlying the estimates for ischemic heart disease in association with secondhand smoke exposure (one-star rating of the risk-outcome relationship). The color of the point indicates whether the point was detected and trimmed as an outlier. The light blue interval corresponds to the 95% UI incorporating between-study heterogeneity; the dark blue interval corresponds to the 95% UI without between-study heterogeneity. The black vertical dotted line reflects the null relative risk value (one) and the red vertical line is the burden of proof function at the 5th quantile for this harmful risk-outcome association. The black data points and horizontal lines each correspond to a mean effect size and 95% UI from the included study identified on the y-axis. We included multiple observations from a single study when effects were reported by location or source of exposure and/or separately by sex or other subgroups. See Supplementary Table 7 for more details on included observations from each study (n = 51 studies).

**Extended Data Fig. 5:**
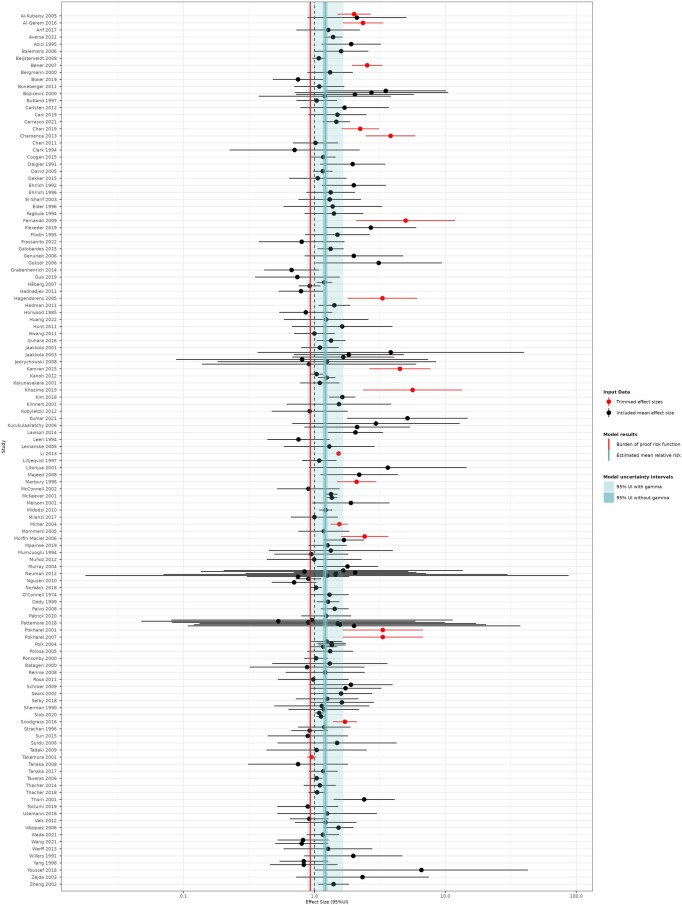
Forest plot of the association between secondhand smoke exposure and asthma. This forest plot presents the estimated mean relative risk, its 95% uncertainty intervals (UI), and the data points underlying the estimates for ischemic heart disease in association with secondhand smoke exposure (one-star rating of the risk-outcome relationship). The color of the point indicates whether the point was detected and trimmed as an outlier. The light blue interval corresponds to the 95% UI incorporating between-study heterogeneity; the dark blue interval corresponds to the 95% UI without between-study heterogeneity. The black vertical dotted line reflects the null relative risk value (one) and the red vertical line is the burden of proof function at the 5th quantile for this harmful risk-outcome association. The black data points and horizontal lines each correspond to a mean effect size and 95% UI from the included study identified on the y-axis. We included multiple observations from a single study when effects were reported by location or source of exposure and/or separately by sex or other subgroups. See Supplementary Table 8 for more details on included observations from each study (n = 125 studies).

**Extended Data Fig. 6:**
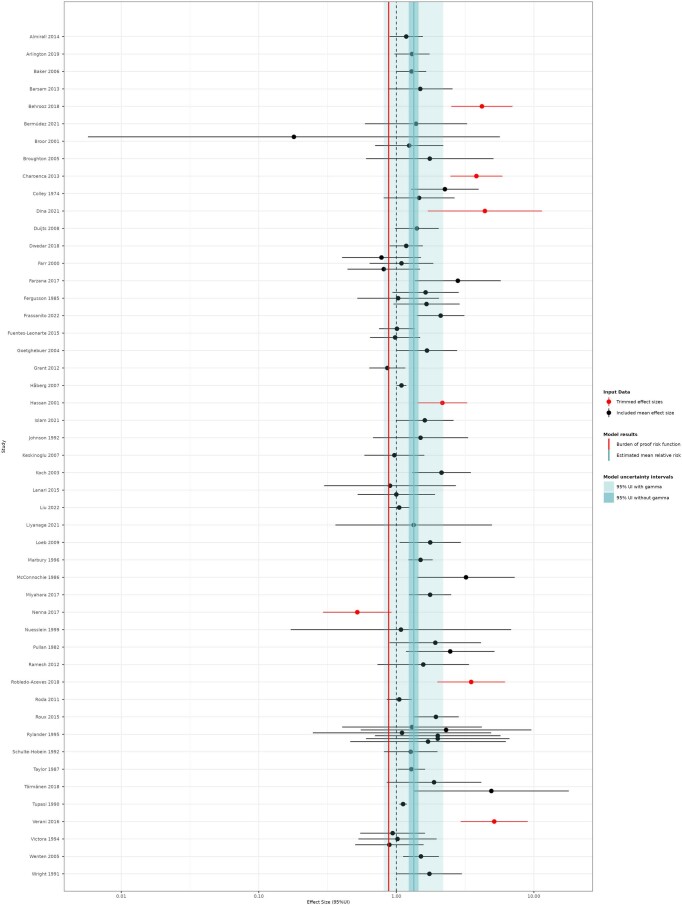
Forest plot of the association between secondhand smoke exposure and lower respiratory infections. This forest plot presents the estimated mean relative risk, its 95% uncertainty intervals (UI), and the data points underlying the estimates for ischemic heart disease in association with secondhand smoke exposure (one-star rating of the risk-outcome relationship). The color of the point indicates whether the point was detected and trimmed as an outlier. The light blue interval corresponds to the 95% UI incorporating between-study heterogeneity; the dark blue interval corresponds to the 95% UI without between-study heterogeneity. The black vertical dotted line reflects the null relative risk value (one) and the red vertical line is the burden of proof function at the 5th quantile for this harmful risk-outcome association. The black data points and horizontal lines each correspond to a mean effect size and 95% UI from the included study identified on the y-axis. We included multiple observations from a single study when effects were reported by location or source of exposure and/or separately by sex or other subgroups. See Supplementary Table 9 for more details on included observations from each study (n = 50 studies).

**Extended Data Fig. 7:**
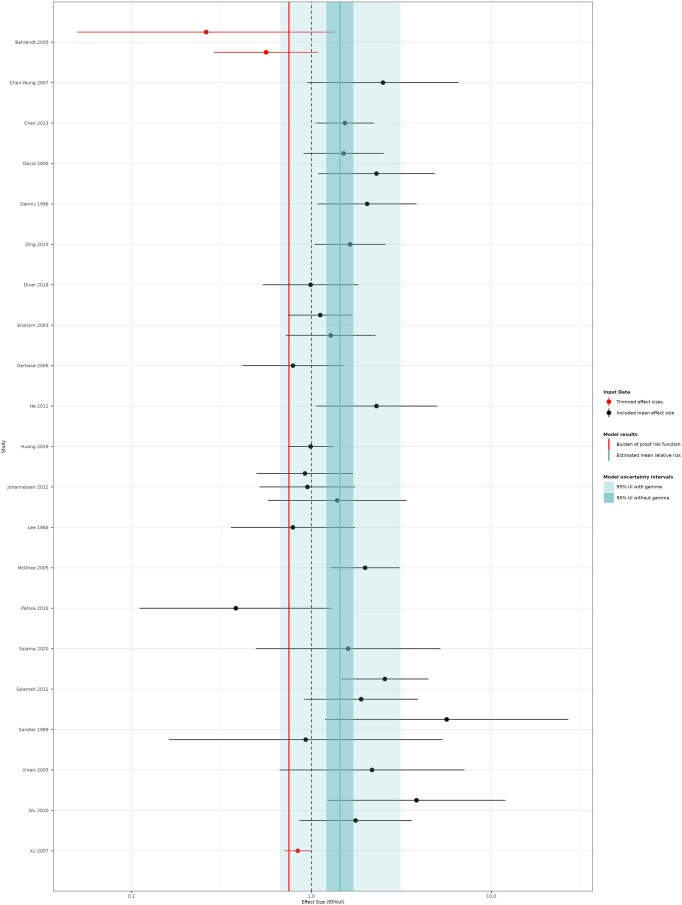
Forest plot of the association between secondhand smoke exposure and chronic obstructive pulmonary disease. This forest plot presents the estimated mean relative risk, its 95% uncertainty intervals (UI), and the data points underlying the estimates for ischemic heart disease in association with secondhand smoke exposure (one-star rating of the risk-outcome relationship). The color of the point indicates whether the point was detected and trimmed as an outlier. The light blue interval corresponds to the 95% UI incorporating between-study heterogeneity; the dark blue interval corresponds to the 95% UI without between-study heterogeneity. The black vertical dotted line reflects the null relative risk value (one) and the red vertical line is the burden of proof function at the 5th quantile for this harmful risk-outcome association. The black data points and horizontal lines each correspond to a mean effect size and 95% UI from the included study identified on the y-axis. We included multiple observations from a single study when effects were reported by location or source of exposure and/or separately by sex or other subgroups. See Supplementary Table 10 for more details on included observations from each study (n = 21 studies).

**Extended Data Fig. 8:**
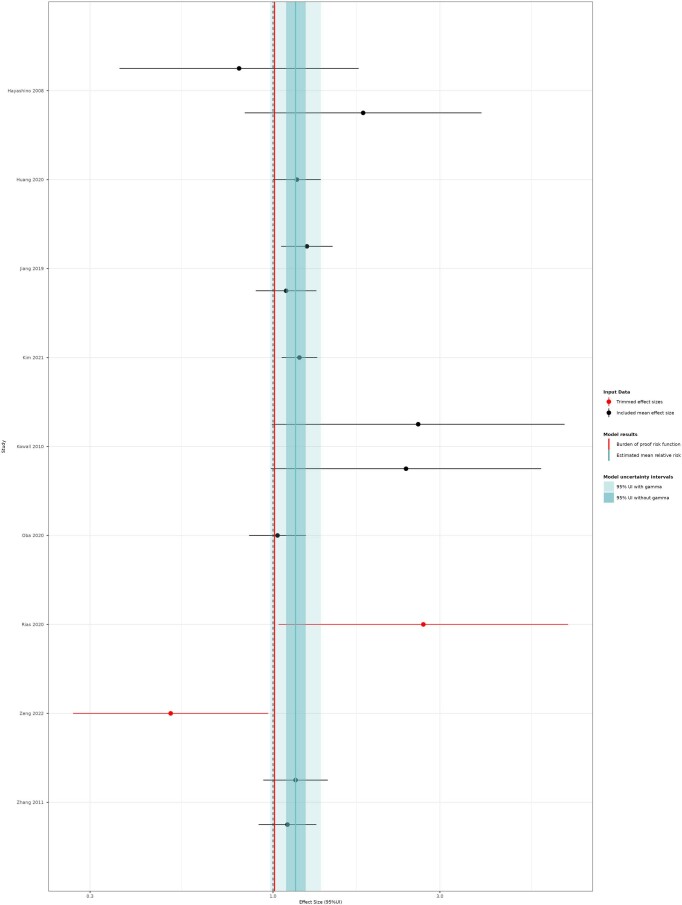
plot of the association between secondhand smoke exposure and type 2 diabetes mellitus. This forest plot presents the estimated mean relative risk, its 95% uncertainty intervals (UI), and the data points underlying the estimates for ischemic heart disease in association with secondhand smoke exposure (two-star rating of the risk-outcome relationship). The color of the point indicates whether the point was detected and trimmed as an outlier. The light blue interval corresponds to the 95% UI incorporating between-study heterogeneity; the dark blue interval corresponds to the 95% UI without between-study heterogeneity. The black vertical dotted line reflects the null relative risk value (one) and the red vertical line is the burden of proof function at the 5th quantile for this harmful risk-outcome association. The black data points and horizontal lines each correspond to a mean effect size and 95% UI from the included study identified on the y-axis. We included multiple observations from a single study when effects were reported by location or source of exposure and/or separately by sex or other subgroups. See Supplementary Table 11 for more details on included observations from each study (n = 9 studies).

**Extended Data Fig. 9:**
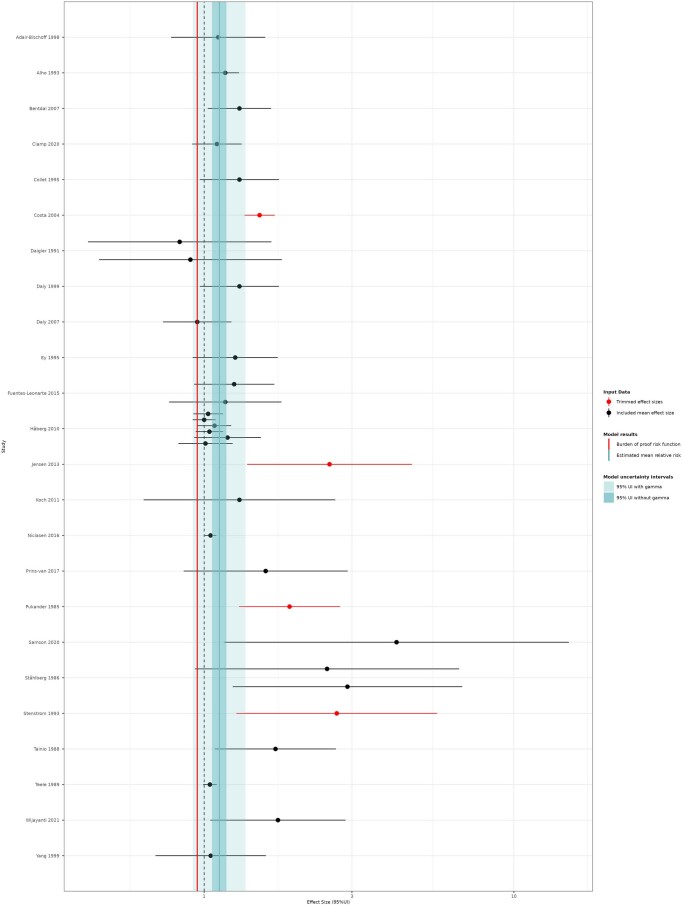
plot of the association between secondhand smoke exposure and otitis media. This forest plot presents the estimated mean relative risk, its 95% uncertainty intervals (UI), and the data points underlying the estimates for ischemic heart disease in association with secondhand smoke exposure (one-star rating of the risk-outcome relationship). The color of the point indicates whether the point was detected and trimmed as an outlier. The light blue interval corresponds to the 95% UI incorporating between-study heterogeneity; the dark blue interval corresponds to the 95% UI without between-study heterogeneity. The black vertical dotted line reflects the null relative risk value (one) and the red vertical line is the burden of proof function at the 5th quantile for this harmful risk-outcome association. The black data points and horizontal lines each correspond to a mean effect size and 95% UI from the included study identified on the y-axis. We included multiple observations from a single study when effects were reported by location or source of exposure and/or separately by sex or other subgroups. See Supplementary Table 12 for more details on included observations from each study (n = 24 studies).

Following the Preferred Reporting Items for Systematic Reviews and Meta-Analyses (PRISMA) guidelines^19^, we systematically searched the literature for studies reporting associations between SHS exposure and each of the nine health outcomes of interest. Definitions of each of the outcomes are reported in Supplementary Table 1. In total, we reviewed 7,109 unique records published between 1 January 1970 and 31 July 2022 identified in PubMed and Web of Science. Through citation searching, 1,972 additional records were identified for screening. Following our predefined inclusion and exclusion criteria (Methods), 410 publications reporting relative risks (RRs) associated with SHS measured as a dichotomous exposure remained for inclusion in our analyses. The data extraction template is presented in Supplementary Table 2, and the review workflow is detailed for each health outcome in the PRISMA flow diagrams (Supplementary Figs. 1–9). The majority of the studies used a case–control design (*n* = 235), followed by prospective cohort (*n* = 156), nested case–control (*n* = 10), retrospective cohort (*n* = 5), case–cohort (*n* = 3) and case-crossover (*n* = 1) designs. The BPRF analyses for asthma (*n* = 125)^20–144^ and lung cancer (*n* = 104)^145–248^ reported in the present study were based on evidence from the highest number of studies, while COPD (*n* = 21)^48,177,208,225,236,249–264^ and type 2 diabetes (*n* = 9)^265–273^ analyses were based on the lowest number of studies. The included studies represent 623 observations from over 178 locations (Supplementary Table 3). Pooled RR estimates for each SHS risk–outcome relationship are provided in Table 2, along with key analytic parameters and characteristics. Forest plots depicting each risk–outcome association are presented in the Extended Data file (Extended Data Figs. 1–9), and all included effect sizes by study are reported in Supplementary Tables 4–12.

**Table 2:** Strength of the evidence for the relationship between exposure to SHS and the nine health outcomes analyzed

Health outcome	RR (95% UI without gamma)	RR (95% UI with gamma)	BPRF	ROS	Star rating	Publication bias	No. of studies	Selected bias covariates	Risk–outcome pair included in GBD 2021
IHD	1.26 (1.2–1.32)	1.26 (1.05–1.52)	1.08	0.04		No	37	Baseline exposure assessment; study design (not prospective cohort)	Y
Stroke	1.16 (1.11–1.22)	1.16 (1.03–1.32)	1.05	0.02		No	20	Selection bias; self-reported outcome	Y
Type 2 diabetes mellitus	1.16 (1.09–1.24)	1.16 (0.98–1.37)	1.01	0.005		No	9	None	Y
Tracheal, bronchus and lung cancer	1.37 (1.3–1.45)	1.37 (0.94–1.99)	1.00	0.001		No	104	Not controlled for smoking	Y
Otitis media	1.12 (1.06–1.18)	1.12 (0.92–1.36)	0.95	−0.03		No	24	Study design (not prospective cohort); self-reported outcome	Y
Asthma	1.21 (1.16–1.26)	1.21 (0.88–1.66)	0.93	−0.04		No	125	Self-reported outcome; children population	N
Lower respiratory infections	1.34 (1.23–1.45)	1.34 (0.81–2.19)	0.88	−0.06		No	50	Not representative population; ever SHS exposure	Y
Breast cancer	1.22 (1.13–1.31)	1.22 (0.75–1.98)	0.81	−0.11		No	51	Study design (not prospective cohort); not controlled for smoking	Y
COPD	1.44 (1.21–1.71)	1.44 (0.67–3.12)	0.75	−0.14		No	21	Selection bias; not controlled for smoking	Y

The reported mean RR and its 95% uncertainty interval (UI) reflect the risk an individual who has been exposed to secondhand smoking has of developing the outcome of interest relative to that of someone who has not been exposed to secondhand smoking. Gamma is the estimated between-study heterogeneity. We report the 95% UI when not incorporating between-study heterogeneity—‘95% UI without gamma’—and when accounting for between-study heterogeneity—‘95% UI with gamma’. The BPRF is calculated for risk–outcome pairs that were found to have significant relationships at an 0.05 level of significance when not incorporating between-study heterogeneity (that is, the lower bound of the 95% UI without gamma does not cross the null RR value of 1). The BPRF corresponds to the fifth-quantile estimate of RR accounting for between-study heterogeneity closest to the null for each risk–outcome pair, and it reflects the most conservative estimate of excess risk associated with secondhand smoking that is consistent with the available data. Since we define secondhand smoking exposure as a dichotomous risk factor, that is, an individual either has been exposed or has not, the ROS is calculated as the signed value of log(BPRF) divided by 2. Negative ROSs indicate that the evidence of the association is very weak and inconsistent. For ease of interpretation, we have transformed the ROS and BPRF into a star rating (1–5) with a higher rating representing a larger effect with stronger evidence. The potential existence of publication bias, which, if present, would affect the validity of the results, was tested using Egger’s regression. Included studies represent all available relevant data identified through our systematic reviews from January 1970 through July 2022. The selected bias covariates were chosen for inclusion in the model using an algorithm that systematically detects bias covariates that correspond to significant sources of bias in the included observations. If selected, the observations were adjusted to better reflect the gold standard values of the covariate. For more information about the candidate bias covariates that were selected for in each model, see Supplementary Information.

### Cardiovascular diseases

**Fig. 1:**
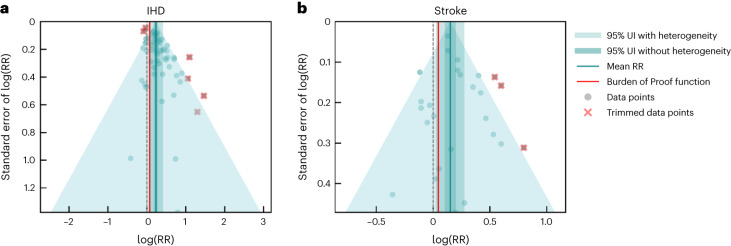
Modified funnel plots for SHS exposure and two cardiovascular outcomes. **a**,**b**, These modified funnel plots show the residuals of the reported mean RR relative to 0, the null value, on the *x* axis and the residuals of the standard error, as estimated from both the reported standard error and gamma, relative to 0 on the *y* axis, for IHD (**a**) and stroke (**b**). The light-blue vertical interval corresponds to the 95% uncertainty interval incorporating between-study heterogeneity; the dark-blue vertical interval corresponds to the 95% uncertainty interval (UI) without between-study heterogeneity; the dots are each included observation; the red Xs are outliered observations; the gray dotted line reflects the null log(RR); the blue line is the mean log(RR) for SHS and the outcome of interest; and the red line is the Burden of Proof function at the fifth quantile for these harmful risk–outcome associations.

We identified 37 studies (59 observations)^177,207,208,215,225,236,252,262,274–302^ quantifying the relationship between SHS exposure and IHD and 20 studies (26 observations)^176,207,208,225,236,252,262,278,296,297,303–312^ assessing the relationship between SHS and stroke (Table 2 and Supplementary Tables 4 and 5). Our conservative analysis of the effect of SHS on IHD yielded an estimated RR of 1.26 (1.05–1.52) (Table 2, Fig. 1a and Extended Data Fig. 1), inclusive of between-study heterogeneity (gamma). We estimated the BPRF—which corresponds to the fifth quantile of RR closest to null and represents the lowest estimate of harmful SHS risk consistent with available evidence—to be 1.08, suggesting that SHS exposure increases an individual’s risk of IHD by a conservative minimum of 8%. In the BPRF framework, this translates to a risk–outcome score (ROS) of 0.04, which distinguishes the SHS–IHD relationship as a two-star risk–outcome pair, which can be interpreted as weak evidence of an association based on the available data (Table 2). Covariates accounting for cases where exposure to SHS was measured at baseline only (rather than multiple times during follow-up) and use of nonprospective cohort design were found to be statistically significant and were adjusted for within our final model (Table 2).

Similarly, a weak but statistically significant relationship was found between SHS exposure and the risk of stroke. The estimated RR and uncertainty inclusive of between-study heterogeneity was 1.16 (1.03–1.32) (Table 2, Fig. 1b and Extended Data Fig. 2). Based on our conservative interpretation of the data, we estimated a BPRF of 1.05, indicating that exposure to tobacco smoke was associated with at least a 5% higher risk of stroke. This corresponds to a ROS of 0.02 and a two-star rating, consistent with weak evidence. In the final model, we adjusted for potential selection bias (based on percentage follow-up for longitudinal study designs and percentages of cases and controls for which exposure data could be ascertained for case–control designs) and for studies based on self-reported outcomes, as these covariates were found to be statistically significant by our bias covariate algorithm (Table 2).

**Extended Data Fig. 10:**
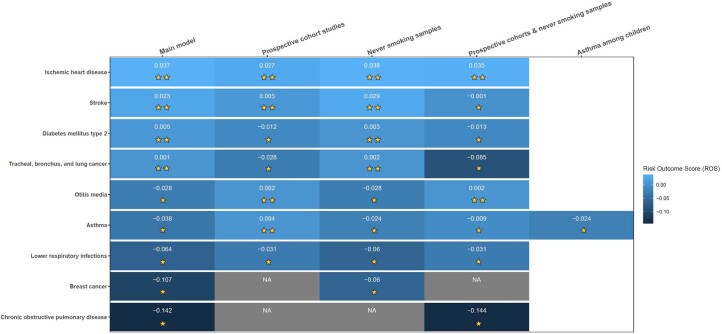
Summarized results of the primary model and sensitivity analyses conducted across all nine health outcomes. This heatmap reports the summarized results of the main model and the sensitivity analyses (columns) conducted for each of the nine health outcomes (rows) reported in this study. Detailed results for each of the sensitivity models are presented in the Supplementary Information (Supplementary Tables 13–16). Sensitivity analyses reflect the impact of restricting the input data to 1) prospective cohort studies, 2) observations associated with never-smokers, and 3) both prospective cohort studies and never-smoking samples. For asthma, we additionally restrict the data to children population aged 16 or less. General model parameters remained constant across models; we trimmed 10% of the data if more than 10 observations were available for the specific model. The color of the blue boxes and the number depicted in each box corresponds to the resulting risk-outcome score (ROS) calculated for models in which the estimates of association without incorporating between-study heterogeneity were statistically significant. Grey boxes depict models that did not pass this threshold and, thus, ROS did not apply (NA). For models that did pass this threshold, the ROS reflects a conservative interpretation of the data that aligns with the Burden of Proof approach incorporating between-study heterogeneity and other sources of uncertainty. The ROS is translated into a star rating from 1 to 5 stars based on thresholds outlined in Zheng et al. The star rating for each model result is reported as the yellow stars in each box. A one-star association suggests that there is weak evidence supporting estimates of an association between the risk and the outcome. A two-star association reflects that there is weak-to-moderate evidence suggesting an association between the risk and outcome, and additional stars illustrate increasing strength of evidence.

The two-star rating for IHD was consistent with sensitivity analyses in which we restricted the models to studies with a prospective cohort design (Supplementary Table 13), subset to observations of never smokers only (Supplementary Table 14), and applied both these restrictions at the same time (Supplementary Table 15). When restricted to prospective cohort data for never smokers only, the association between SHS and stroke was downgraded to one star (ROS −0.001) (Extended Data Fig. 10). We did not detect publication bias, as identified by Egger’s regression test, in the primary analysis or in any of the sensitivity analyses for the cardiovascular outcomes (Table 2 and Supplementary Tables 13–15).

### Cancer

**Fig. 2:**
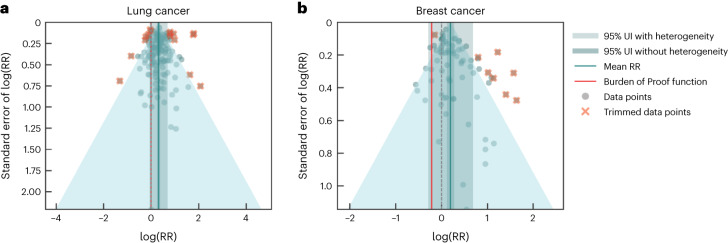
Modified funnel plots for SHS exposure and two cancer outcomes. **a**,**b**, These modified funnel plots show the residuals of the reported mean RR relative to 0, the null value, on the *x* axis and the residuals of the standard error, as estimated from both the reported standard error and gamma, relative to 0 on the *y* axis, for lung cancer (**a**) and breast cancer (**b**). The light-blue vertical interval corresponds to the 95% uncertainty interval incorporating between-study heterogeneity; the dark-blue vertical interval corresponds to the 95% uncertainty interval (UI) without between-study heterogeneity; the dots are each included observation; the red Xs are outliered observations; the gray dotted line reflects the null log(RR); the blue line is the mean log(RR) for SHS and the outcome of interest; the red line is the Burden of Proof function at the fifth quantile for these harmful risk–outcome associations.

The conservative BPRF analysis indicated that passive smoking was weakly associated with an increased risk of lung cancer, based on a BPRF of 1.00 and a corresponding ROS of 0.001 (Table 2), which translates to a two-star rating at the lower threshold of the two-star range and suggests that SHS exposure was associated with at least around 1% higher risk of lung cancer. When between-study heterogeneity and other sources of uncertainty were accounted for, the estimated RR was 1.37 (0.94–1.99) (Table 2, Fig. 2a and Extended Data Fig. 3). The bias covariate algorithm selected observations that did not originally control for smoking to be adjusted in the final model (Table 2). In a sensitivity analysis in which we restricted the data to prospective cohort studies, the strength of the association was even lower (BPRF 0.95, ROS −0.03), downgrading the relationship to a one-star rating (Extended Data Fig. 10 and Supplementary Table 13).

Our conservative BPRF analysis also found weak evidence of a harmful association between exposure to tobacco smoke and risk of breast cancer (BPRF 0.81, ROS −0.11, one-star rating; Table 2). The meta-analysis, which is supported by 51 unique studies^170,220,313–361^ and 79 observations (Supplementary Table 7), yielded an RR of 1.22 (0.75–1.98), inclusive of between-study heterogeneity (Table 2, Fig. 2b and Extended Data Fig. 4). In our model, observations that did not control for smoking and those from study designs other than prospective cohorts were adjusted since these covariates were found to be significant by our algorithm (Table 2). In further sensitivity analyses, the one-star relationship was still observed when we restricted to observations from never smokers only (Extended Data Fig. 1 and Supplementary Table 14). However, when restricting to prospective cohort studies, we found no statistically significant evidence of an association between exposure to SHS and the risk of breast cancer in our fixed-effect model without between-study heterogeneity; that is, the estimated RR and associated uncertainty without gamma includes the null. These risk–outcome pairs are automatically assigned a zero-star rating, and the BPRF and ROS are not computed (Extended Data Fig. 10 and Supplementary Table 13).

Based on Egger’s regression test, no significant evidence of publication bias was found for the main lung cancer and breast cancer models or the exploratory models (Table 2 and Supplementary Tables 13–15). Visual inspection of the funnel plots supported this finding (Fig. 2).

### Respiratory conditions

**Fig. 3:**
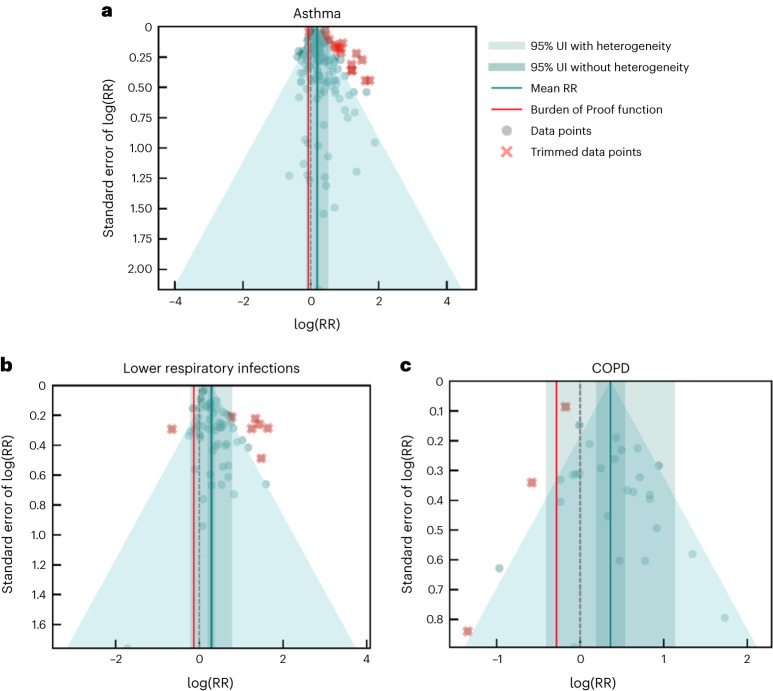
Modified funnel plots for SHS exposure and three respiratory outcomes. These modified funnel plots show the residuals of the reported mean RR relative to 0, the null value, on the *x* axis and the residuals of the standard error, as estimated from both the reported standard error and gamma, relative to 0 on the *y* axis, for asthma (**a**), lower respiratory infections (**b**) and COPD (**c**). The light-blue vertical interval corresponds to the 95% uncertainty interval incorporating between-study heterogeneity; the dark-blue vertical interval corresponds to the 95% uncertainty interval (UI) without between-study heterogeneity; the dots are each included observation; the red Xs are outliered observations; the gray dotted line reflects the null log(RR); the blue line is the mean log(RR) for SHS and the outcome of interest; the red line is the Burden of Proof function at the fifth quantile for these harmful risk–outcome associations.

We evaluated the association between exposure to SHS and three respiratory conditions: asthma, lower respiratory infections and COPD. Based on the conservative BPRF framework, the evidence supporting each of these relationships was weak (one-star rating), when between-study heterogeneity and other sources of bias were taken into account. Across these outcomes, no significant publication bias was detected in the primary models (Table 2) or in the sensitivity analyses (Supplementary Tables 13–16). For SHS and asthma, a risk–outcome pair not yet included in the GBD, the estimated RR incorporating between-study heterogeneity into the uncertainty was 1.21 (0.88–1.66) (Table 2, Fig. 3a and Extended Data Fig. 5). Data points associated with a self-reported diagnosis and those restricted to children (age ≤16 years) were adjusted for in our main model, as the corresponding bias covariates were found to be statistically significant (Table 2). The BPRF and ROS were 0.93 and −0.04, respectively, which equates to a one-star risk classification. When restricting to prospective cohort studies, a two-star rating for the relationship between SHS and asthma was observed (Extended Data Fig. 10 and Supplementary Tables 13).

The meta-analysis of the risk of lower respiratory infections associated with SHS exposure included 50 studies^53,64,91,134,362–407^ and 66 observations (Supplementary Table 9) and yielded an RR and uncertainty interval inclusive of between-study heterogeneity of 1.34 (0.81–2.19) (Table 2, Fig. 3b and Extended Data Fig. 6). The BPRF (0.88) and corresponding ROS (−0.06) translated into a one-star rating, consistent with weak evidence of an association between passive smoking and increased risk of lower respiratory infections. The covariate selection algorithm flagged studies performed among populations that were not generalizable and those that used exposure definitions other than current SHS (for example, ever exposure to SHS) to be adjusted in our final model (Table 2). The strength of association as measured in the BPRF framework was not sensitive to any additional restrictions we applied to the input data, meaning that the one-star rating was still observed when we subset the data to prospective cohorts, never-smoking samples and a combination of the two (Extended Data Fig. 10 and Supplementary Tables 13–15).

Similar to the results for asthma and lower respiratory infections, the ROS for COPD was also negative (−0.14), equating to a one-star rating, indicating weak evidence of an association between SHS exposure and the risk of COPD. When accounting for between-study heterogeneity, the RR was 1.44 (0.67–3.12) (Table 2, Fig. 3c and Extended Data Fig. 7). Covariates representing studies that did not control for smoking and those with potential selection bias were found to be significant in our primary model and were adjusted for accordingly (Table 2). When including observations from seven prospective cohorts only, we found no statistically significant evidence of an association between SHS exposure and COPD when not including between-study heterogeneity (RR 1.21 (0.93–1.57, without gamma)). This was similar to the result we found when subsetting the data to never-smoking populations (RR 1.15 (0.95–1.40, without gamma)). The one-star association was observed, however, in a sensitivity analysis in which we applied both data restrictions simultaneously (Extended Data Fig. 10 and Supplementary Tables 13–15).

### Other health outcomes

**Fig. 4:**
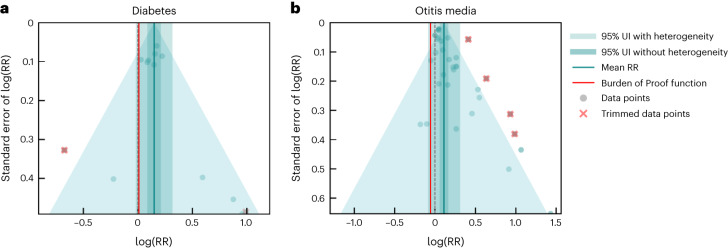
Modified funnel plots for SHS exposure and type 2 diabetes and otitis media. **a**,**b**, These modified funnel plots show the residuals of the reported mean RR relative to 0, the null value, on the *x* axis and the residuals of the standard error, as estimated from both the reported standard error and gamma, relative to 0 on the *y* axis, for type 2 diabetes (**a**) and otitis media (**b**). The light-blue vertical interval corresponds to the 95% uncertainty interval incorporating between-study heterogeneity; the dark-blue vertical interval corresponds to the 95% uncertainty interval (UI) without between-study heterogeneity; the dots are each included observation; the red Xs are outliered observations; the gray dotted line reflects the null log(RR); the blue line is the mean log(RR) for SHS and the outcome of interest; the red line is the Burden of Proof function at the fifth quantile for these harmful risk–outcome associations.

Our conservative Burden of Proof assessment found evidence of weak harmful effects between SHS exposure and risk of type 2 diabetes, with an RR of 1.16 (0.98–1.37) when accounting for between-study heterogeneity (Table 2, Fig. 4a and Extended Data Fig. 8). The BPRF value was 1.01 with a corresponding ROS of 0.005, which suggests that passive smoking is associated with at least a 1% higher risk of type 2 diabetes, translating to a two-star risk. The two-star relationship remained consistent in our sensitivity analysis in which we subset the input data to observations of never smokers only (Extended Data Fig. 10 and Supplementary Table 14). Restricting the data to prospective cohort studies resulted in a downgrade in star rating to a one-star risk (Extended Data Fig. 10 and Supplementary Table 13). Moreover, the automated covariate selection did not find any significant bias covariates for inclusion in the main or alternative final models (Table 2 and Supplementary Tables 13–15). No publication bias was found in the type 2 diabetes models.

For otitis media, our meta-analysis of 24 studies^132,385,408–429^ and 32 observations (Supplementary Table 12) yielded an RR of 1.12 (0.92–1.36) when accounting for between-study heterogeneity (Table 2, Fig. 4b and Extended Data Fig. 9). The corresponding BPRF was 0.95, which equates to a ROS of −0.03 and a one-star rating (weak evidence of association). Bias covariates that captured nonprospective cohort studies and studies in which the outcome of interest was self-reported (rather than diagnosed by a doctor) were detected as significant and adjusted for within our final model (Table 2). All studies included in our otitis media model were conducted in never-smoker populations (or classified as such given the age of the studied population (Methods and Supplementary Information Section 2.2)); however, when restricting our analysis to prospective cohort studies, the ROS was slightly higher, elevating the risk–outcome relationship to a two-star rating, with no bias covariates found statistically significant (Extended Data Fig. 10 and Supplementary Table 13). We found no publication bias in our primary model, but a statistically significant evidence of publication bias was found in our prospective cohort sensitivity analysis.

## Discussion

In this study, we applied the Burden of Proof framework to quantify the relationship between exposure to SHS and nine health outcomes and to assess the strength of the evidence underlying these associations^430^. As suggested by our estimates not accounting for between-study heterogeneity, we found evidence that passive smoking is associated with statistically significant increases in the risk of all nine health outcomes. When taking the BPRF to conservatively interpret the available data by accounting for between-study heterogeneity and other sources of bias, the evidence suggests that being exposed to SHS increased the risk of IHD, stroke and type 2 diabetes by a minimum of 8%, 5% and 1%, respectively, corresponding to two-star associations with SHS. The two-star rating was also found for the relationship with lung cancer, for which SHS was found to increase the risk by a minimum of around 1%. The available evidence of associations between SHS and otitis media, asthma, lower respiratory infections, breast cancer and COPD are weaker and these risk–outcome pairs were classified as one-star associations.

As long known, being exposed to SHS is irrefutably harmful to human health and our findings are broadly in support of tobacco control measures aimed at protecting nonsmokers from tobacco smoke. Overall, we found SHS to have small to moderate quantitative impacts on health—mean effect sizes range from 1.12 for otitis media to 1.44 for COPD—which is in line with previous assessments^13,431–441^ and anticipated on the basis of mechanistic processes leading to diseases^5^. The modest strength of the association coupled with heterogeneity present in the underlying data across all nine risk–outcome pairs analyzed resulted in a body of evidence rated as weak under the proposed BPRF rating system (one and two stars), despite the relatively large number of studies included for some of the outcomes.

Nonetheless, even under our conservative interpretation of the available data using the BPRF approach, a particular area of considerable increased risk is cardiovascular health. This finding is consistent with the conclusions drawn by other studies in regard to both IHD and stroke^431,442–445^. In previous dose–response analyses, the harmful effects of SHS on cardiovascular diseases have been found even at low doses of exposure^446–448^. This is of particular concern as IHD and stroke are the two major causes of premature death and loss of healthy life worldwide^449^. Similarly, our findings also suggest that the risk of lung cancer and type 2 diabetes are also elevated for those exposed to SHS. Lung cancer was the fifth leading cause of death globally in 2019 and type 2 diabetes was the eighth leading cause, highlighting the potential benefit that could be achieved for these causes and overall disease burden by further reducing active and passive smoking^449^.

For otitis media, asthma, lower respiratory infections, breast cancer and COPD, the evidence supporting an association with passive smoking is even weaker, with a one-star rating. In the BPRF framework, one-star associations denote risk–outcome pairs for which it would not be surprising if the inclusion of additional data, when available, modifies our findings. Although we found evidence suggesting an association between SHS exposure and these other investigated health outcomes, the associations did not achieve statistical significance when using the BPRF approach to capture uncertainty that accounts for between-study heterogeneity. These findings highlight that the lack of consistent findings across studies is a major factor underlying the weak ROSs assigned to these exposure–outcome associations. The substantial inconsistency across studies with different designs and degrees of selection and information bias is not unusual for a risk factor with weak strength of associations, such as SHS exposure. In particular, we found insufficient evidence to support an association with SHS when restricting to prospective cohort studies (breast cancer) and never smokers (COPD), even when not incorporating between-study heterogeneity in our estimates of uncertainty. Indeed, authors have drawn markedly different conclusions about the presence and magnitude of association between passive exposure to tobacco smoke and breast cancer, especially when accounting for age group and menopausal status^11,12,346,350,450^. Because breast cancer is the most frequent type of cancer in women and accounts for substantial morbidity and mortality, research should continue to examine its association with exposure to SHS^451^.

Our study contributes to previous iterations of the GBD by not only increasing the number of studies informing each of the existing SHS–outcome associations but by assessing the relationship between passive smoking and asthma, a risk–outcome pair not yet incorporated into the GBD but deemed eligible for further consideration. Similar to our findings, population-specific meta-analyses found positive associations between passive exposure to tobacco smoke and both an overall increase in asthma risk within the Asian population^452^ and the occurrence of childhood asthma^453^. Expanding the evidence base around SHS and other health outcomes is a means to more accurately capture the full breadth of disease burden attributable to this risk.

Furthermore, the BPRF framework employed in this study addresses many of the limitations of existing meta-analytical approaches^18^. Given the high degree of inconsistency observed across results in the SHS literature, using the BPRF to capture the unexplained sources of variation between studies is particularly relevant for our study. Moreover, the translation of our conservative findings surrounding the health effects of SHS into a star rating simplifies the communication and interpretation of the available evidence. However, viewed in isolation, neither the calculated effect sizes nor the BPRF or star ratings imply causality or lack thereof. These are some of the components to be considered when defining health policy and research funding priorities. The high prevalence of exposure to SHS in a scenario with an increasing number of smokers and the harmful associations with conditions of global relevance warrant policy focus even with weak evidence supporting the analyses when compared to other less prevalent risks associated with rare or less severe outcomes and strong supporting evidence.

In spite of the observed variability in the SHS data, which accounts in part for the ROS and star-rating results we obtained, our study reaffirms that exposure to SHS is a harmful risk factor of great public health importance. As outlined by the World Health Organization, smoke-free policies in combination with strategies promoting active smoking cessation and noninitiation are among the most effective tobacco control interventions to reduce passive smoking and protect health^454^. Studies of the effects of smoke-free laws found that hospital admission and mortality rates for cardiovascular and respiratory conditions decreased after the implementation of smoking bans^455–459^. However, comprehensive smoke-free legislation (that is, covering all indoor public places) is in place in only 67 countries, protecting less than 25% of the world’s population^7^. Therefore, faster-paced implementation and adequate enforcement of this type of policy can play an important role in minimizing the burden of smoking-attributable diseases and deaths among nonsmokers. Moreover, private homes remain a major source of SHS exposure, particularly for women and children^3,460^, and our findings can help reinforce awareness of the adverse consequences of SHS exposure and promote adoption of voluntary restrictions in homes^461^.

When interpreting this study’s results, a number of limitations need to be taken into consideration, most of which are associated with the limitations of the available data, which in turn may have led to an underestimation of the RRs in our findings. First, we used studies in which exposure to SHS was self-reported, either directly or measured by proxy (that is, living with a smoking parent or spouse), and this can result in misclassification of exposed and nonexposed participants. Second, the information collected by surveys frequently asks about current exposure; this means that we lack information on cumulative exposure to SHS and formerly exposed individuals could have been misclassified as unexposed. Third, to account for the lack of a standardized way of capturing exposure to SHS in existing studies, we classify exposure to SHS as dichotomous (exposed or unexposed); however, this may oversimplify the risk profile associated with SHS by not accounting for differences in intensity or frequency of exposure. Fourth, our results draw upon data that rely on a range of exposure definitions. For example, the underlying studies capture information about exposure to SHS at either home or work and, in the absence of these, at any location more broadly. Previous studies have found different effect sizes for SHS exposure at home and at work^442,443,462^, a factor that was not investigated in our analysis. However, a covariate was created to assess if data points associated with exposure at any location were significantly different from those associated with exposure at work or home, which is the SHS definition adopted by the GBD. Because we use the GBD exposure definition, we also do not include data for exposure in public settings, which are largely limited. In the included studies, those not exposed at work or home may be exposed to SHS at other settings, and this bias, similar to our first limitation above, will tend to underestimate the true RR. Finally, despite the inclusion of asthma, a new health outcome to be considered for inclusion in the GBD, the outcomes assessed here do not necessarily reflect the harms associated with SHS in full. Future efforts could synthesize the available evidence concerning the relationship between SHS and other health outcomes for which some evidence of an association exist, for example, maternal outcomes and low birth weight^463^.

In conclusion, our study, which examines the relationship between SHS exposure and nine health outcomes using the BPRF framework developed by Zheng and colleagues^430^, reaffirms that SHS should be an area of priority for policymakers, physicians and public health advocates for strengthening tobacco-control measures, especially in locations with high smoking and SHS prevalence. Due to heterogeneity and uncertainty in the data, small effect sizes, small numbers of studies or a combination of these reasons, the existing strength of evidence on the health effects of SHS was considered weak, especially for the relationship with otitis media, asthma, lower respiratory infections, breast cancer and COPD. Even when applying a conservative interpretation of the evidence, our results suggest that exposure to SHS increases the risk to nonsmokers for cardiovascular outcomes, lung cancer and type 2 diabetes. Prospective cohort studies with greater consistency in case definitions, more precise measurement of exposures and larger samples can result in less inconsistent data, and thus more targeted recommendations.

## Methods

### Overview

In this study, we employed the BPRF methodology developed by Zheng and colleagues^430^ to conservatively estimate the association between SHS exposure and nine health outcomes and assess the strength of the evidence supporting each of these associations. We define SHS as the current exposure, among nonsmokers, to smoke from any combustible tobacco product at home or at work, the same definition used in the GBD studies. BPRF methods have already been employed to assess the health effects associated with smoking^464^, high systolic blood pressure^465^ and consumption of unprocessed red meat^466^ and vegetables^467^. Specifically, the BPRF framework uses a meta-regression–Bayesian, regularized, trimmed (MR-BRT) tool to estimate pooled RRs, along with uncertainty intervals, accounting for systematic bias, within-study correlation and unexplained between-study heterogeneity. Briefly, we followed the six analytical steps included in the BPRF meta-analytical approach, namely: (1) conducting a systematic review and extracting data from identified studies reporting on the association between SHS exposure and the outcomes of interest; (2) estimating a pooled RR that compares the risk of being exposed to SHS relative to those not exposed to SHS; (3) testing and adjusting for systematic sources of bias within input sources; (4) quantifying unexplained between-study heterogeneity while adjusting for within-study correlation and the number of studies; (5) evaluating publication and reporting bias; and (6) estimating the BPRF to generate a conservative estimate of the risk associated with SHS exposure and to compute a corresponding ROS. The BPRF is defined as the 5th (if harmful) or 95th (if protective) quantile estimate of the risk closest to the null estimate, with the 5th quantile reflecting the smallest harmful effect of a risk exposure on a given health outcome that is consistent with the available evidence. The ROS, which is the signed value of the log RR, reflects the effect size and strength of evidence for each risk–outcome association estimated. ROSs are translated into a star-rating scale from 1 to 5 to aid the interpretation of the results. We describe each of these steps below, and further details are available elsewhere^430^.

Similar to previous studies using BPRF methods^464–467^, the RRs, BPRFs and ROSs estimated in this study are not specific to or disaggregated by certain populations, meaning that we did not estimate RRs separately by geography, sex or age group. However, the assessment of the association between SHS and breast cancer relied on studies that were conducted in female-only populations. For asthma, we conducted a children-specific sensitivity analysis that is described along other sensitivity analyses below.

The present study complies with the PRISMA guidelines^19^ (Supplementary Tables 17 and 18 and Supplementary Figs. 1–9) and Guidelines for Accurate and Transparent Health Estimates Reporting (GATHER) recommendations (Supplementary Table 19)^468^. As a component of the GBD, the present analysis was approved by the University of Washington institutional review board committee (study no. 9060).

### Health outcomes of interest

We selected outcomes on the basis of the availability of epidemiological evidence on their potential relationship with SHS. Eight out of the nine outcomes of interest—lung and breast cancer, IHD, stroke, COPD, lower respiratory infections, type 2 diabetes and otitis media—constitute SHS risk–outcome pairs considered in previous iterations of the GBD and were initially selected using the World Cancer Research Fund criteria for convincing or probable evidence as detailed in Murray et al.^1^. Through review of published meta-analyses and systematic reviews and consultations with key external experts, we identified asthma as an additional health outcome of interest to SHS researchers and one for which sufficient literature was available to enable BPRF analytic methods; we therefore included it in our analysis. Reference and alternative definitions of each of the outcomes are listed in Supplementary Table 1.

### Systematic review

We conducted separate systematic reviews to identify peer-reviewed literature reporting relative measures of association quantifying the relationship between SHS exposure and each health outcome of interest. We searched PubMed and Web of Science for studies published between 1 January 1970 and 31 July 2022. Furthermore, we reviewed the citation lists of the systematic reviews and meta-analyses captured in our searches to identify additional pertinent studies.

Briefly, after deduplicating the search results, each study’s title and abstract were manually screened by a single reviewer for inclusion eligibility. Subsequently, the full text was retrieved and screened, and data were extracted from those studies that passed our inclusion criteria of being published in English; being a case–control, cohort, case-cohort or case-crossover study conducted in participant groups likely to be generalizable; using suitable exposure and outcome definitions; and reporting both a relative measure of association (that is, RR, odds ratio or hazard ratio) and some measure of uncertainty (for example, sample size, standard error or confidence intervals). In terms of outcome definitions, studies using either a reference or an alternative health outcome definition met our inclusion criteria (Supplementary Table 1). As for SHS exposure, we included studies with varied SHS definitions, including proxies, but restricted to those reporting dichotomous current or ever exposure (that is, yes/no exposure). We excluded studies reporting only former exposure to SHS and those only assessing exposure in specific public settings. To better match our SHS definition, we also excluded studies and observations reporting health risk for current smokers. Finally, for all outcomes but otitis media, lower respiratory infections and asthma, we excluded studies that exclusively assessed childhood exposure to SHS to best account for the exposure temporality reflected in the SHS definition in GBD. In the case that multiple studies provided estimates from the same cohort, we included only the study with the largest sample or follow-up period so as not to duplicate data. The search strings used in each database, detailed inclusion and exclusion criteria, and outcome-specific PRISMA flow diagrams are available in Supplementary Figs. 1–9.

Data from eligible publications were manually extracted into a template designed to capture information about study and sample characteristics, exposure and outcome definitions, ascertainment methods, effect size and corresponding uncertainty reported for each model/population, and covariates included in the statistical analyses. We also assessed each study for risk of potential bias following the Grading of Recommendations, Assessment, Development and Evaluations (GRADE) approach and recorded the information in the extraction template^469^. As part of the exposure definition review, we cataloged multiple aspects of SHS exposure linked to each reported effect size, including the location of exposure (home or/and work combined; home; work; or any/unspecified location), the source of exposure (family; parental; maternal; paternal; spouse; or any/unspecified source), the timing of exposure (current or ever), and the smoking status of the exposed population (nonsmoker; never smoker; former smoker; or any/unspecified). Those studies performed only among children aged 15 years or less with an original ‘unspecified’ smoking status were reassigned to ‘adjusted never smokers’ and treated as ‘never smokers’ and ‘controlled for smoking’ in our analyses. In the GBD, we assume no smoking prevalence for ages under 10 years; given the small prevalence for ages 10–15 and since most of the identified childhood studies included those past age 10, we believe this classification best reflects the smoking status of the studied population in these cases. All extracted data underwent manual quality assurance by the research team to verify accuracy. For a full list of extracted variables, with corresponding definitions, see Supplementary Table 2.

### Estimating pooled RRs for each risk–outcome pair

We selected the effect sizes to be used in our meta-analytic approach within each included study and health outcome based on a prioritization cascade. All included effect sizes are reported in Supplementary Tables 4–12. Starting with the exposure definition, we chose the data points that closest matched the GBD risk definition in terms of the smoking status of the exposed population, followed by the location of exposure, the source of exposure, and the temporality. Thus, data points for nonsmokers currently exposed to SHS at home or work combined were prioritized over the other ones. In the absence of this exact definition, we prioritized the inclusion of effect sizes for each/any of the components of the GBD risk definition (that is, never smoker; former smoker; home; work) over those associated with a broader definition (that is, any/unspecified location or smoking status). Due to data sparsity, ‘ever exposure’ definitions were accepted for inclusion if results for ‘current exposure’ were not available. We did not include observations referring to exposure in specific settings other than home or work (for example, public settings or public transportation) or exposure among current smokers. Bias covariates were created to capture the impact of using alternate exposure definitions.

After this first selection stage, we proceeded with identifying the least granular analyses to be used in our models. For example, within each study and outcome, sex- and age-specific results were dropped in favor of aggregated data points, and results associated with the entire study population were retained over those for subgroup analyses when possible. We also favored observations reporting the risk of incidence and mortality combined over those that estimated each outcome type separately in cases where both were available. Moreover, for stroke, we dropped observations for subtypes (ischemic and hemorrhagic stroke) in favor of those for overall stroke due to data availability restrictions and to allow for best comparability across studies. In our last data selection step, the most-adjusted remainder data points within each study outcome were selected for inclusion in our analyses. This selection process is described in more detail in Supplementary Information.

To reduce the influence on our model of multiple observations coming from the same study, we adjusted the standard errors of effect sizes reported for multiple non-mutually exclusive exposure groups in each study by a factor matching the number of repeated measurements within each age–sex–smoking status group (Supplementary Information Section 2.2).

Finally, we used the MR-BRT tool to conduct each risk–outcome meta-regression analysis with the log-space RR of the outcome modeled as the dependent variable and exposure to SHS as the dichotomous independent variable (exposed to SHS versus not exposed to SHS). These analyses generated a single estimate of pooled RR of the given health outcome occurring for those exposed to SHS relative to unexposed counterparts. Following the BPRF methodology, we applied a 10% likelihood-based data-trimming algorithm to detect and remove outliers that may otherwise over-influence the model. This approach is suggested for all analyses with more than ten data points; therefore, it was implemented across all of our primary risk–outcome assessments and most of our sensitivity analyses^470^.

### Testing and adjusting for biases across study designs and characteristics

Following the GRADE approach, we used the extracted data related to specific study characteristics to create binary covariates that captured potential sources of systematic bias within our input datasets. These covariates reflected the risk of bias associated with study design (prospective cohorts versus others), representativeness of the study population, exposure measurement (measured at baseline only versus multiple times during follow-up), outcome assessment method (self-report versus medical records), degree of control for confounding, and potential for selection bias (based on percentage follow-up for longitudinal study designs and percentages of cases and controls for which exposure data could be ascertained for case–control designs). Additionally, given SHS-specific characteristics, we created covariates to indicate whether a study controlled for smoking, regardless of other confounders, and whether the definition of SHS matched the one in GBD in terms of the location of exposure (home or work exposure versus broader definitions). A covariate reflecting studies performed among females only was also created. For the stroke models, we created two bias covariates to account for possible differences between studies reporting subtype-specific effect size only and those reporting stroke as an aggregated outcome; for asthma we created a specific covariate to indicate if a study was performed among children only (≤16 years old). Detailed information about each of the bias covariates is provided in Supplementary Information Section 5 (Supplementary Table 20). We systematically tested for the effect of bias covariates using a selection algorithm, which uses a step-wise Lasso strategy to identify statistically significant covariates at a threshold of 0.05, and adjusted for the selected bias covariates in the final model used to generate the RR estimates. Covariates were eligible for testing if there was a minimum of two data points in the model associated with each covariate value. If multiple covariates had the same distribution of values within a model, we randomly selected one of the covariates to be tested.

### Quantifying remaining between-study heterogeneity

After adjusting for study-level bias covariates, we used a linear mixed-effects model to capture the remaining unexplained between-study heterogeneity, in which we included a study-level random slope (gamma) and a study-level random intercept for within-study correlation. We derived the uncertainty of gamma using the inverse Fisher information matrix, which is sensitive to the number of studies, study design and reported uncertainty. The draws of gamma are used to derive the conservative uncertainty interval estimate for our RR (with gamma), estimated from both the uncertainty surrounding the mean effect and the 95th quantile of between-study heterogeneity. The RR without gamma, as reported in Table 2, is reported with an uncertainty derived without fully accounting for between-study heterogeneity and reflects the RR estimates that are typically reported in traditional meta-analyses, while that with gamma better reflects the degree of consistency across the underlying studies. In this study, the RR metric of primary interest was the pooled RR with 95% uncertainty intervals that are inclusive (using gamma) of the effect of between-study heterogeneity. The estimated gamma for each risk–outcome primary assessment is presented in Supplementary Table 21.

### Evaluating publication and reporting bias

To assess the presence of publication or reporting bias, we visually inspected the funnel plots (Figs. 1–4) produced for each risk–outcome evaluation, which show the residuals of the reported mean RR against the residuals of the standard error from each individual study. Visual inspection of the plots was accompanied by Egger’s regression tests to test for significant correlation between the standard error and the reported effect size. We did not find evidence of publication or reporting bias across any of the risk–outcome pairs in our primary models. We found publication bias for otitis media in one of our sensitivity analyses. We flagged the potential publication bias but did not correct for it in the model.

### Estimating the BPRF

In our final step, we estimated the BPRF, which reflects the most conservative estimate of the association between exposure to SHS and the selected health outcomes that is consistent with the available evidence. For dichotomous harmful risk factors, the BPRF corresponds to the fifth quantile of RR closest to null, derived from the RR model inclusive of between-study heterogeneity. For each risk–outcome pair, the BPRF can be used to compute measures of increased or decreased risk of developing the health outcome due to exposure to the risk factor. BPRF values can be converted into ROSs, defined as the signed value of the average log RR of the BPRF. Large positive ROSs correspond to strong and consistent evidence of an association, while small positive ROSs and negative ROSs reflect weak evidence for an association, based on the available data. To facilitate the interpretation and comparison of the ROS results, the BPRF framework translates the ROS into star rating categories ranging from one to five (one star, ≤0.0 ROS; two stars, >0.0–0.14 ROS; three stars, >0.14–0.41 ROS; four stars, >0.41–0.62 ROS; five stars, >0.62 ROS). A one-star rating indicates weak evidence of association, while a five-star rating indicates very strong evidence. Zero-star risk–outcome pairs are not based on ROSs values but are defined as pairs for which there is no evidence of a statistically significant association between the risk and the health outcome when not accounting for between-study heterogeneity (that is, the 95% uncertainty interval without gamma crosses the null). Risk–outcome pairs receiving a one- through five-star rating are eligible for inclusion in the GBD.

### Model validation

The validity of the BPRF approach to meta-analyze data extracted across studies has been extensively and rigorously evaluated by Zheng and colleagues^430^. For the present study, we conducted three main sensitivity analyses to examine the robustness of our primary findings to our data input in which we kept most of the model parameters consistent but (1) restricted our analysis to studies with a prospective cohort design; (2) subset our input data to never-smoking samples only; and (3) applied both these restrictions in conjunction. For asthma, specifically, we ran an additional model in which we restrict the data to those studies performed among children only (≤16 years old). The only modification in our model parameters was related to the implementation of the 10% data trimming, which is dependent on the number of observations available for each outcome model (that is, data are trimmed only if ten observations or more are included). We present the detailed results of these sensitivity analyses in Supplementary Tables 13–16.

### Statistical analysis and reproducibility

Analyses were carried out using R version 4.0.5 and Python version 3.10.9.

This investigation relied on existing published data. No statistical method was used to predetermine sample size. For each health outcome, we included all studies that met our inclusion criteria. This study did not engage in primary data collection, randomization or blinding. Therefore, data exclusions were not relevant to the present study, and, as such, no data were excluded from the analyses. We have made our data and code available to foster reproducibility.

### Reporting summary

Further information on research design is available in the Nature Portfolio Reporting Summary linked to this article.

## Online content

Any methods, additional references, Nature Portfolio reporting summaries, source data, extended data, supplementary information, acknowledgements, peer review information; details of author contributions and competing interests; and statements of data and code availability are available at 10.1038/s41591-023-02743-4.

### Supplementary information

Supplementary Information Supplementary information.

Reporting Summary

## Data Availability

The findings from this study are supported by data extracted from published literature. We cite all studies included in our analyses in our manuscript. Studies’ characteristics are presented in Supplementary Table 3, and data points included in each analysis are available in Supplementary Tables 4–12. Details on data sources can also be found on the Burden of Proof visualization tool (https://vizhub.healthdata.org/burden-of-proof/).

## Code availability

All code used for these analyses is publicly available online (https://github.com/ihmeuw-msca/burden-of-proof/).
