# Author Correction: Health effects associated with exposure to secondhand smoke: a Burden of Proof study

**DOI:** 10.1038/s41591-024-02832-y

**Published:** 2024-01-30

**Authors:** Luisa S. Flor, Jason A. Anderson, Noah Ahmad, Aleksandr Aravkin, Sinclair Carr, Xiaochen Dai, Gabriela F. Gil, Simon I. Hay, Matthew J. Malloy, Susan A. McLaughlin, Erin C. Mullany, Christopher J. L. Murray, Erin M. O’Connell, Chukwuma Okereke, Reed J. D. Sorensen, Joanna Whisnant, Peng Zheng, Emmanuela Gakidou

**Affiliations:** 1 Institute for Health Metrics and Evaluation, University of Washington, Seattle, WA USA; 2 Department of Health Metrics Sciences, School of Medicine, University of Washington, Seattle, WA USA; 3 Department of Global Health, University of Washington, Seattle, WA USA

**Keywords:** Risk factors, Diseases

Correction to: *Nature Medicine* 10.1038/s41591-023-02743-4, published online 9 January 2024.

In the version of the article initially published, the surname of Reed J. D. Sorensen appeared incorrectly (as Sorenson) and has now been corrected in the HTML and PDF versions of the article.

