# Supplementary material for: Health effects associated with exposure to secondhand smoke: a Burden of Proof study
Source: Nat Med. 2024 Jan 9;30(1):149–67. doi: 10.1038/s41591-023-02743-4 (PMC10803272; doi:10.1038/s41591-023-02743-4)
Supplement: Supplementary file 1 — Supplementary information. [file 41591_2023_2743_MOESM1_ESM.pdf]

nature medicine

Article

https://doi.org/10.1038/s41591-023-02743-4

Health effects associated with exposure to secondhand smoke: a Burden of Proof study

In the format provided by the authors and unedited

Table of Contents

Section 1: Data source identification and assessment 3

Section 1.1: Literature searches 3

Section 1.1.1: PubMed search strings 3

Section 1.1.2: Web of Science Search Strings 10

Section 1.2: Inclusion and exclusion criteria 14

Section 1.3: Outcome definitions 15

Table S1. Definitions of nine included health outcomes 15

Section 1.4: PRISMA diagrams 18

Section 1.5: Data extraction 27

Table S2. Data Extraction Template 27

Section 2: Data Inputs 36

Section 2.1: Study characteristics 37

Table S3. Summary of study characteristics 37

Section 2.2: Effect size data details 93

Table S4. Summary of data inputs ischemic heart disease 95

Table S5. Summary of data inputs for stroke 101

Table S6. Summary of data inputs for tracheal, bronchus, and lung cancer 104

Table S7. Summary of data inputs for breast cancer 122

Table S8. Summary of data inputs for asthma 129

Table S9. Summary of data inputs for lower respiratory infections 145

Table S10. Summary of data inputs for chronic obstructive pulmonary disease 153

Table S12. Summary of data inputs for diabetes mellitus type 2 158

Table S12. Summary of data inputs for otitis media 160

Section 3: Sensitivity Analyses 163

Section 3.1: Sensitivity analyses results 164

Table S13. Strength of the evidence for the relationship between secondhand smoking when including prospective cohort studies only 164

Table S14. Strength of the evidence for the relationship between secondhand smoking when including observations from never-smokers only 165

Table S15. Strength of the evidence for the relationship between secondhand smoking when including prospective cohorts and observations from never-smokers only 166

Table S16. Strength of the evidence for the relationship between secondhand smoking and asthma when including data from children aged 16 or less only 167

Section 4: GATHER and PRISMA checklists 168
Section 4.1: PRISMA 168
Table S17. PRISMA 2020 Abstract Checklist 168
Table S18. PRISMA 2020 Manuscript Checklist 169
Section 4.2: GATHER 171
Table S19. GATHER Checklist 171
Section 5: Study quality & bias assessment 172
Table S20. Bias covariates tested in the primary model for each health outcome, by study 175
Section 6: Between-study Heterogeneity 216
Table S21. Between-study heterogeneity (gamma) for each risk-outcome pair 216

Section 1: Data source identification and assessment

Data for the present analysis was identified through two systematic reviews for each of our nine health outcomes of interest. Each data source was evaluated on the basis of pre-established inclusion criteria described in Section 1.2.

Section 1.1: Literature searches

We conducted separate systematic reviews for ischemic heart disease (IHD); stroke; type 2 diabetes mellitus; lower respiratory infections; breast cancer; tracheal, bronchus, and lung cancer; otitis media; asthma; and chronic obstructive pulmonary disease (COPD). These reviews encompassed two databases: PubMed and Web of Science. These databases were selected to cover a broad swath of peer-reviewed global and regional literature. The search strings were last run on August 2, 2022, for PubMed, and September 27-28, 2022, for Web of Science to capture all studies, irrespective of publication language, published from January 1, 1970 through July 31, 2022. The search strings used for each outcome-database search are shown below.

Section 1.1.1: PubMed search strings

Ischemic Heart Disease

(Tobacco smoke pollution [MeSH Terms] OR second-hand[Title/Abstract] OR secondhand[Title/Abstract] OR environmental tobacco[Title/Abstract] OR tobacco smoke[Title/Abstract] OR cigarette smoke[Title/Abstract] OR passive smok*[Title/Abstract] OR involuntary smok*[Title/Abstract] OR parental smoking[Title/Abstract] OR maternal smoking[Title/Abstract]) AND

(Coronary Artery Disease[Mesh] OR Myocardial Ischemia[Mesh] OR atherosclerosis[Mesh] OR Coronary Artery Disease[TiAb] OR Myocardial Ischemia[TiAb] OR cardiac ischemia[TiAb] OR silent ischemia[TiAb] OR atherosclerosis [TiAb] OR Ischaemic heart disease[TiAb] OR Ischemic heart disease[TiAb] OR coronary heart disease[TiAb] OR myocardial infarction[TiAb] OR heart attack[TiAb] OR heart infarction[TiAb]) AND

(Case-Control Studies[MeSH Terms] OR Cross-Over Studies[MeSH Terms] OR Cohort Studies[MeSH Terms] OR Systematic Review[Publication Type] OR Meta-Analysis[Publication Type] OR “systematic review”[Title/Abstract] OR “meta-analysis”[Title/Abstract] OR “cohort”[Title/Abstract] OR “cross-over”[Title/Abstract] OR “crossover”[Title/Abstract] OR “case-control”[Title/Abstract] OR “prospective”[Title/Abstract] OR “retrospective”[Title/Abstract] OR “longitudinal”[Title/Abstract] OR “follow-up”[Title/Abstract] OR Dose-Response Relationship, Drug[MeSH Terms] OR “dose-response”[Title/Abstract]) AND

(Risk[MeSH Terms] OR Odds Ratio[MeSH Terms] OR “risk”[Title/Abstract] OR “odds ratio”[Title/Abstract] OR “cross-product ratio”[Title/Abstract] OR “hazards ratio”[Title/Abstract] OR “hazard ratio”[Title/Abstract]) AND

(“2020/01/01”[PDat] : “2022/07/31”[Pdat]) NOT

(animals[MeSH Terms] NOT Humans[MeSH Terms])

Stroke

(Tobacco smoke pollution [MeSH Terms] OR second-hand[Title/Abstract] OR secondhand[Title/Abstract] OR environmental tobacco[Title/Abstract] OR tobacco smoke[Title/Abstract] OR cigarette smoke[Title/Abstract] OR passive smok*[Title/Abstract] OR involuntary smok*[Title/Abstract] OR parental smoking[Title/Abstract] OR maternal smoking[Title/Abstract]) AND

(brain infarction[MeSH Terms] OR stroke[MeSH Terms] OR intracranial hemorrhages[MeSH Terms] OR "stroke"[Title/Abstract] OR "brain infarction"[Title/Abstract] OR "cerebral infarction"[Title/Abstract] OR "intracerebral hemorrhage"[Title/Abstract] OR "intracerebral haemorrhage"[Title/Abstract] OR "subarachnoid hemorrhage"[Title/Abstract] OR "subarachnoid haemorrhage"[Title/Abstract]) AND

(Case-Control Studies[MeSH Terms] OR Cross-Over Studies[MeSH Terms] OR Cohort Studies[MeSH Terms] OR Systematic Review[Publication Type] OR Meta-Analysis[Publication Type] OR "systematic review"[Title/Abstract] OR "meta-analysis"[Title/Abstract] OR "cohort"[Title/Abstract] OR "cross-over"[Title/Abstract] OR "crossover"[Title/Abstract] OR "case-control"[Title/Abstract] OR "prospective"[Title/Abstract] OR "retrospective"[Title/Abstract] OR "longitudinal"[Title/Abstract] OR "follow-up"[Title/Abstract] OR Dose-Response Relationship, Drug[MeSH Terms] OR "dose-response"[Title/Abstract]) AND

(Risk[MeSH Terms] OR Odds Ratio[MeSH Terms] OR "risk"[Title/Abstract] OR "odds ratio"[Title/Abstract] OR "cross-product ratio"[Title/Abstract] OR "hazards ratio"[Title/Abstract] OR "hazard ratio"[Title/Abstract]) AND

("2020/01/01"[Pdat] : "2022/07/31"[Pdat]) NOT

(animals[MeSH Terms] NOT Humans[MeSH Terms])

Type 2 Diabetes mellitus

(Tobacco smoke pollution [MeSH Terms] OR second-hand[Title/Abstract] OR secondhand[Title/Abstract] OR environmental tobacco[Title/Abstract] OR tobacco smoke[Title/Abstract] OR cigarette smoke[Title/Abstract] OR passive smok*[Title/Abstract] OR involuntary smok*[Title/Abstract] OR parental smoking[Title/Abstract] OR maternal smoking[Title/Abstract]) AND

(Diabetes Mellitus, Type 2[MeSH Terms] OR "diabetes"[Title/Abstract]) AND

(Case-Control Studies[MeSH Terms] OR Cross-Over Studies[MeSH Terms] OR Cohort Studies[MeSH Terms] OR Systematic Review[Publication Type] OR Meta-Analysis[Publication Type] OR "systematic review"[Title/Abstract] OR "meta-analysis"[Title/Abstract] OR "cohort"[Title/Abstract] OR "cross-over"[Title/Abstract] OR "crossover"[Title/Abstract] OR "case-control"[Title/Abstract] OR "prospective"[Title/Abstract] OR "retrospective"[Title/Abstract] OR "longitudinal"[Title/Abstract] OR "follow-up"[Title/Abstract] OR Dose-Response Relationship, Drug[MeSH Terms] OR "dose-response"[Title/Abstract]) AND

(Risk[MeSH Terms] OR Odds Ratio[MeSH Terms] OR “risk”[Title/Abstract] OR “odds ratio”[Title/Abstract] OR “cross-product ratio”[Title/Abstract] OR “hazards ratio”[Title/Abstract] OR “hazard ratio”[Title/Abstract]) AND

(“1970/01/01”[Pdat] : “2022/07/31”[Pdat]) NOT

(animals[MeSH Terms] NOT Humans[MeSH Terms])

Lower respiratory infections

(Tobacco smoke pollution [MeSH Terms] OR second-hand[Title/Abstract] OR secondhand[Title/Abstract] OR environmental tobacco[Title/Abstract] OR tobacco smoke[Title/Abstract] OR cigarette smoke[Title/Abstract] OR passive smok*[Title/Abstract] OR involuntary smok*[Title/Abstract] OR parental smoking[Title/Abstract] OR maternal smoking[Title/Abstract]) AND

(pneumonia[MeSH Terms] OR bronchiolitis[MeSH Terms] OR “lower respiratory”[Title/Abstract] OR “pneumonia”[Title/Abstract] OR “bronchiolitis”[Title/Abstract] OR “pulmonary inflammation”[Title/Abstract] OR “lung inflammation”[Title/Abstract]) AND

(Case-Control Studies[MeSH Terms] OR Cross-Over Studies[MeSH Terms] OR Cohort Studies[MeSH Terms] OR Systematic Review[Publication Type] OR Meta-Analysis[Publication Type] OR “systematic review”[Title/Abstract] OR “meta-analysis”[Title/Abstract] OR “cohort”[Title/Abstract] OR “cross-over”[Title/Abstract] OR “crossover”[Title/Abstract] OR “case-control”[Title/Abstract] OR “prospective”[Title/Abstract] OR “retrospective”[Title/Abstract] OR “longitudinal”[Title/Abstract] OR “follow-up”[Title/Abstract] OR Dose-Response Relationship, Drug[MeSH Terms] OR “dose-response”[Title/Abstract]) AND

(Risk[MeSH Terms] OR Odds Ratio[MeSH Terms] OR “risk”[Title/Abstract] OR “odds ratio”[Title/Abstract] OR “cross-product ratio”[Title/Abstract] OR “hazards ratio”[Title/Abstract] OR “hazard ratio”[Title/Abstract]) AND

(“1970/01/01”[Pdat] : “2022/07/31”[Pdat]) NOT

(animals[MeSH Terms] NOT Humans[MeSH Terms])

Breast cancer

(Tobacco smoke pollution [MeSH Terms] OR second-hand[Title/Abstract] OR secondhand[Title/Abstract] OR environmental tobacco[Title/Abstract] OR tobacco smoke[Title/Abstract] OR cigarette smoke[Title/Abstract] OR passive smok*[Title/Abstract] OR involuntary smok*[Title/Abstract] OR parental smoking[Title/Abstract]OR maternal smoking[Title/Abstract]) AND

(breast neoplasm[MeSH Terms] OR “breast cancer”[Title/Abstract] OR “breast cancers”[Title/Abstract] OR “breast neoplasm”[Title/Abstract] OR “breast neoplasms”[Title/Abstract] OR “mammary cancer”[MeSH Terms] OR “mammary cancers”[Title/Abstract] OR “breast malignant neoplasm”[Title/Abstract] OR “breast malignant neoplasms”[Title/Abstract] OR “mammary carcinoma”[Title/Abstract] OR “mammary carcinomas”[Title/Abstract] OR “breast carcinoma”[Title/Abstract] OR “breast carcinomas”[Title/Abstract] OR “mammary neoplasm”[Title/Abstract] OR “mammary neoplasms”[Title/Abstract] OR “breast tumor”[Title/Abstract] OR “breast tumors”[Title/Abstract] OR “cancer of the breast”[Title/Abstract] OR “cancers of the breast”[Title/Abstract] OR “neoplasm of the breast”[Title/Abstract] OR “tumor of the breast”[Title/Abstract]) AND

(Case-Control Studies[MeSH Terms] OR Cross-Over Studies[MeSH Terms] OR Cohort Studies[MeSH Terms] OR Systematic Review[Publication Type] OR Meta-Analysis[Publication Type] OR “systematic review”[Title/Abstract] OR “meta-analysis”[Title/Abstract] OR “cohort”[Title/Abstract] OR “cross-over”[Title/Abstract] OR “crossover”[Title/Abstract] OR “case-control”[Title/Abstract] OR “prospective”[Title/Abstract] OR “retrospective”[Title/Abstract] OR “longitudinal”[Title/Abstract] OR “follow-up”[Title/Abstract] OR Dose-Response Relationship, Drug[MeSH Terms] OR “dose-response”[Title/Abstract]) AND

(Risk[MeSH Terms] OR Odds Ratio[MeSH Terms] OR “risk”[Title/Abstract] OR “odds ratio”[Title/Abstract] OR “cross-product ratio”[Title/Abstract] OR “hazards ratio”[Title/Abstract] OR “hazard ratio”[Title/Abstract]) AND

(“2020/01/01”[Pdat] : “2022/07/31”[Pdat]) NOT (animals[MeSH Terms] NOT Humans[MeSH Terms])

Tracheal, bronchus, and lung cancer

(((((((Tobacco smoke pollution [MeSH Terms] OR second-hand[Title/Abstract] OR secondhand[Title/Abstract] OR environmental tobacco[Title/Abstract] OR tobacco smoke[Title/Abstract] OR cigarette smoke[Title/Abstract] OR passive smok*[Title/Abstract] OR involuntary smok*[Title/Abstract] OR parental smoking[Title/Abstract] OR maternal smoking[Title/Abstract]))) AND

((lung neoplasm[MeSH Terms] OR “lung cancer”[Title/Abstract] OR “lung cancers”[Title/Abstract] OR “lung neoplasms”[Title/Abstract] OR “pulmonary neoplasm”[Title/Abstract] OR “pulmonary cancer”[Title/Abstract] OR “pulmonary cancers”[Title/Abstract] OR “pulmonary neoplasms”[Title/Abstract] OR “lung malignant neoplasm”[Title/Abstract] OR “lung malignant neoplasms”[Title/Abstract] OR “pulmonary malignant neoplasm”[Title/Abstract] OR “pulmonary malignant neoplasms”[Title/Abstract] OR “pulmonary carcinoma”[Title/Abstract] OR “pulmonary carcinomas”[Title/Abstract] OR “lung carcinoma”[Title/Abstract] OR “lung carcinomas”[Title/Abstract] OR “lung tumor”[Title/Abstract] OR “pulmonary tumors”[Title/Abstract] OR “cancer of the lung”[Title/Abstract] OR “cancers of the lung”[Title/Abstract] OR “neoplasm of the lung”[Title/Abstract] OR “tumor of the lung”[Title/Abstract] OR “bronchus cancer”[Title/Abstract] OR “bronchi cancer”[Title/Abstract] OR “bronchial cancer”[Title/Abstract] OR “bronchus cancers”[Title/Abstract] OR “bronchi cancers”[Title/Abstract] OR “bronchial cancers”[Title/Abstract] OR “bronchus neoplasm”[Title/Abstract] OR “bronchi neoplasm”[Title/Abstract] OR “bronchial neoplasm”[Title/Abstract] OR “bronchus neoplasms”[Title/Abstract] OR “bronchi neoplasms”[Title/Abstract] OR “bronchial neoplasms”[Title/Abstract] OR “bronchus malignant neoplasm”[Title/Abstract] OR “bronchi malignant neoplasm”[Title/Abstract] OR “bronchial malignant neoplasm”[Title/Abstract] OR “bronchus malignant neoplasms”[Title/Abstract] OR “bronchi malignant neoplasms”[Title/Abstract] OR “bronchial malignant neoplasms”[Title/Abstract] OR “bronchus carcinoma”[Title/Abstract] OR “bronchi carcinoma”[Title/Abstract] OR “bronchial carcinoma”[Title/Abstract] OR “bronchus carcinomas”[Title/Abstract] OR “bronchi carcinomas”[Title/Abstract] OR “bronchial carcinomas”[Title/Abstract] OR “bronchus tumor”[Title/Abstract] OR “bronchi tumor”[Title/Abstract] OR “bronchial tumor”[Title/Abstract] OR “bronchus tumors”[Title/Abstract] OR “bronchi tumors”[Title/Abstract] OR “bronchial tumors”[Title/Abstract] OR “cancer of the bronchus”[Title/Abstract] OR “cancer of the bronchi”[Title/Abstract] OR “cancers of the bronchus”[Title/Abstract] OR “cancers of the bronchi”[Title/Abstract] OR “neoplasm of the bronchus”[Title/Abstract] OR “neoplasm of the bronchi”[Title/Abstract] OR “neoplasms of the bronchus”[Title/Abstract] OR “neoplasms of the bronchi”[Title/Abstract] OR “tumor of the bronchus”[Title/Abstract] OR “tumor of the bronchi”[Title/Abstract] OR “tumors of the bronchus”[Title/Abstract] OR “tumors of the bronchi”[Title/Abstract] OR “trachea cancer”[Title/Abstract] OR “tracheal cancer”[Title/Abstract] OR “trachea cancers”[Title/Abstract] OR “tracheal cancers”[Title/Abstract] OR “trachea neoplasm”[Title/Abstract] OR “tracheal neoplasm”[Title/Abstract] OR “trachea neoplasms”[Title/Abstract] OR “tracheal neoplasms”[Title/Abstract] OR “trachea malignant neoplasm”[Title/Abstract] OR “tracheal malignant neoplasm”[Title/Abstract] OR “trachea malignant neoplasms”[Title/Abstract] OR “tracheal malignant neoplasms”[Title/Abstract] OR “trachea carcinoma”[Title/Abstract] OR “tracheal carcinoma”[Title/Abstract] OR “trachea carcinomas”[Title/Abstract] OR “tracheal carcinomas”[Title/Abstract] OR “trachea tumor”[Title/Abstract] OR “tracheal tumor”[Title/Abstract] OR “trachea tumors”[Title/Abstract] OR “tracheal tumors”[Title/Abstract] OR “cancer of the trachea”[Title/Abstract] OR “cancers of the trachea”[Title/Abstract] OR “neoplasm of the trachea”[Title/Abstract] OR “neoplasms of the trachea”[Title/Abstract] OR “tumor of the bronchus”[Title/Abstract] OR “tumor of the bronchi”[Title/Abstract] OR “tumors of the bronchus”[Title/Abstract] OR “tumors of the bronchi”[Title/Abstract]))) AND

((Case-Control Studies[MeSH Terms] OR Cross-Over Studies[MeSH Terms] OR Cohort Studies[MeSH Terms] OR Systematic Review[Publication Type] OR Meta-Analysis[Publication Type] OR “systematic review”[Title/Abstract] OR “meta-analysis”[Title/Abstract] OR “cohort”[Title/Abstract] OR “cross-over”[Title/Abstract] OR “crossover”[Title/Abstract] OR “case-control”[Title/Abstract] OR “prospective”[Title/Abstract] OR “retrospective”[Title/Abstract] OR “longitudinal”[Title/Abstract] OR “follow-up”[Title/Abstract] OR Dose-Response Relationship, Drug[MeSH Terms] OR “dose-response”[Title/Abstract]))) AND

((Risk[MeSH Terms] OR Odds Ratio[MeSH Terms] OR “risk”[Title/Abstract] OR “odds ratio”[Title/Abstract] OR “cross-product ratio”[Title/Abstract] OR “hazards ratio”[Title/Abstract] OR “hazard ratio”[Title/Abstract]))) AND

(“1970/01/01”[Pdat] : “2022/07/31”[Pdat])) NOT

((animals[MeSH Terms] NOT Humans[MeSH Terms]))

Otitis media

(Tobacco smoke pollution [MeSH Terms] OR “second-hand”[Title/Abstract] OR “secondhand”[Title/Abstract] OR “environmental tobacco”[Title/Abstract] OR “tobacco smoke”[Title/Abstract] OR “cigarette smoke”[Title/Abstract] OR passive smok*[Title/Abstract] OR involuntary smok*[Title/Abstract] OR “parental smoking”[Title/Abstract] OR “maternal smoking”[Title/Abstract]) AND

(Otitis Media[MeSH Terms] OR “otitis media”[Title/Abstract] OR “middle ear infection” [Title/Abstract] OR “middle ear disease” [Title/Abstract] OR “ear infection”[Title/Abstract] OR “ear disease”[Title/Abstract] OR “otitis” [Title/Abstract]) AND

(Case-Control Studies[MeSH Terms] OR Cross-Over Studies[MeSH Terms] OR Cohort Studies[MeSH Terms] OR Systematic Review[Publication Type] OR Meta-Analysis[Publication Type] OR “systematic review”[Title/Abstract] OR “meta-analysis”[Title/Abstract] OR “cohort”[Title/Abstract] OR “cross-over”[Title/Abstract] OR “crossover”[Title/Abstract] OR “case-control”[Title/Abstract] OR “prospective”[Title/Abstract] OR “retrospective”[Title/Abstract] OR “longitudinal”[Title/Abstract] OR “follow-up”[Title/Abstract] OR Dose-Response Relationship, Drug[MeSH Terms] OR “dose-response”[Title/Abstract]) AND

(Risk[MeSH Terms] OR Odds Ratio[MeSH Terms] OR “risk”[Title/Abstract] OR “odds ratio”[Title/Abstract] OR “cross-product ratio”[Title/Abstract] OR “hazards ratio”[Title/Abstract] OR “hazard ratio”[Title/Abstract]) AND

(“2020/01/01”[Pdat] : “2022/07/31”[Pdat]) NOT

(animals[MeSH Terms] NOT Humans[MeSH Terms])

Asthma

(Tobacco smoke pollution [MeSH Terms] OR second-hand[Title/Abstract] OR secondhand[Title/Abstract] OR environmental tobacco[Title/Abstract] OR tobacco smoke[Title/Abstract] OR cigarette smoke[Title/Abstract] OR passive smok*[Title/Abstract] OR involuntary smok*[Title/Abstract] OR parental smoking[Title/Abstract] OR maternal smoking[Title/Abstract]) AND

("Asthma"[Mesh] OR "asthma"[tiab]) AND

(Case-Control Studies[MeSH Terms] OR Cross-Over Studies[MeSH Terms] OR Cohort Studies[MeSH Terms] OR Systematic Review[Publication Type] OR Meta-Analysis[Publication Type] OR "systematic review"[Title/Abstract] OR "meta-analysis"[Title/Abstract] OR "cohort"[Title/Abstract] OR "cross-over"[Title/Abstract] OR "crossover"[Title/Abstract] OR "case-control"[Title/Abstract] OR "prospective"[Title/Abstract] OR "retrospective"[Title/Abstract] OR "longitudinal"[Title/Abstract] OR "follow-up"[Title/Abstract] OR Dose-Response Relationship, Drug[MeSH Terms] OR "dose-response"[Title/Abstract]) AND

(Risk[MeSH Terms] OR Odds Ratio[MeSH Terms] OR "risk"[Title/Abstract] OR "odds ratio"[Title/Abstract] OR "cross-product ratio"[Title/Abstract] OR "hazards ratio"[Title/Abstract] OR "hazard ratio"[Title/Abstract]) AND

("1970/01/01"[Pdat] : "2022/07/31"[Pdat]) NOT

(animals[MeSH Terms] NOT Humans[MeSH Terms])

Chronic obstructive pulmonary disease

(Tobacco smoke pollution [MeSH Terms] OR second-hand[Title/Abstract] OR secondhand[Title/Abstract] OR environmental tobacco[Title/Abstract] OR tobacco smoke[Title/Abstract] OR cigarette smoke[Title/Abstract] OR passive smok*[Title/Abstract] OR involuntary smok*[Title/Abstract] OR parental smoking[Title/Abstract] OR maternal smoking[Title/Abstract]) AND

(Pulmonary Disease, Chronic Obstructive[Mesh] OR "COPD"[Title/Abstract] OR "emphysema"[Title/Abstract] OR "chronic obstructive pulmonary disease"[Title/Abstract]) AND

(Case-Control Studies[MeSH Terms] OR Cross-Over Studies[MeSH Terms] OR Cohort Studies[MeSH Terms] OR Systematic Review[Publication Type] OR Meta-Analysis[Publication Type] OR "systematic review"[Title/Abstract] OR "meta-analysis"[Title/Abstract] OR "cohort"[Title/Abstract] OR "cross-over"[Title/Abstract] OR "crossover"[Title/Abstract] OR "case-control"[Title/Abstract] OR "prospective"[Title/Abstract] OR "retrospective"[Title/Abstract] OR "longitudinal"[Title/Abstract] OR "follow-up"[Title/Abstract] OR Dose-Response Relationship, Drug[MeSH Terms] OR "dose-response"[Title/Abstract]) AND

(Risk[MeSH Terms] OR Odds Ratio[MeSH Terms] OR "risk"[Title/Abstract] OR "odds ratio"[Title/Abstract] OR "cross-product ratio"[Title/Abstract] OR "hazards ratio"[Title/Abstract] OR "hazard ratio"[Title/Abstract]) AND

("2020/01/01"[Pdat] : "2022/07/31"[Pdat]) NOT

(animals[MeSH Terms] NOT Humans[MeSH Terms])

Section 1.1.2: Web of Science Search Strings

Ischemic Heart Disease

(TS=(smok* NEAR/4 exposure) OR TS=second$hand OR TS=”environmental tobacco” OR (TS=”tobacco smok* pollution” OR TS=”tobacco smok*”) OR TS=”cigarette smok*” OR TS=”passive smok*” OR TS=”involuntary smok*” OR TS=”parental smok*” OR TS=”maternal smok*”) AND

(TS=”coronary artery disease” OR TS=”myocardial isch$emia” OR TS=”cardiac isch$emia” OR TS=”silent isch$emia” OR TS=atherosclerosis OR TS=”isch$emic heart disease” OR TS=”coronary heart disease” OR TS=”myocardial infarction” OR TS=”heart attack” OR TS=”heart infarction”) AND

(TS=”systematic review” OR TS=meta$analysis OR TS=cohort OR TS=cross$over OR TS=case$control OR TS=prospective OR TS=retrospective OR TS=longitudinal OR TS=follow$up OR TS=dose$response) AND

(TS=risk OR TS=”odds ratio” OR TS=”cross$product ratio” OR TS=”hazard$ ratio”) AND

DOP=1970-01-01/2022-07-31 NOT

(TS=animal$ NOT TS=human$)

Stroke

(TS=(smok* NEAR/4 exposure) OR TS=second$hand OR TS=”environmental tobacco” OR (TS=”tobacco smok* pollution” OR TS=”tobacco smok*”) OR TS=”cigarette smok*” OR TS=”passive smok*” OR TS=”involuntary smok*” OR TS=”parental smok*” OR TS=”maternal smoking”) AND

(TS=stroke OR TS=”brain infarction” OR TS=”cerebral infarction” OR TS=”isch$emic stroke” OR TS=”cerebral h$emorrhage” OR TS=”intracerebral h$emorrhage” OR TS=”subarachnoid h$emorrhage”) AND

(TS=”systematic review” OR TS=meta$analysis OR TS=cohort OR TS=cross$over OR TS=case$control OR TS=prospective OR TS=retrospective OR TS=longitudinal OR TS=follow$up OR TS=dose$response) AND

(TS=risk OR TS=”odds ratio” OR TS=”cross$product ratio” OR TS=”hazard$ ratio”) AND

DOP=1970-01-01/2022-07-31 NOT

(TS=animal$ NOT TS=human$)

Type 2 Diabetes mellitus

(TS=(smok* NEAR/4 expsoure) OR TS=second$hand OR TS=”environmental tobacco” OR (TS=”tobacco smok* pollution” OR TS=”tobacco smok*”) OR TS=”cigarette smok*” OR TS=”passive smok*” OR TS=”involuntary smok*” OR TS=”parental smok*” OR TS=”maternal smok*”) AND

(TS=”diabetes mellitus type 2” OR TS=”type 2 diabetes mellitus” OR TS=diabetes) AND

(TS=”systematic review” OR TS=meta$analysis OR TS=cohort OR TS=cross$over OR TS=case$control OR TS=prospective OR TS=retrospective OR TS=longitudinal OR TS=follow$up OR TS=dose$response) AND

(TS=risk OR TS=”odds ratio” OR TS=”cross$product ratio” OR TS=”hazard$ ratio”) AND

DOP=1970-01-01/2022-07-31 NOT

(TS=animal$ NOT TS=human$)

Lower respiratory infections

(TS=(smok* NEAR/4 exposure) OR TS=second$hand OR TS=”environmental tobacco” OR (TS=”tobacco smok* pollution” OR TS=”tobacco smok*”) OR TS=”cigarette smok*” OR TS=”passive smok*” OR TS=”involuntary smok*” OR TS=”parental smok*” OR TS=”maternal smok*”) AND

(TS=pneumonia OR TS=bronchiolitis OR TS=”lower respiratory” OR TS=”pulmonary inflammation” OR TS=”lung inflammation”) AND

(TS=”systematic review” OR TS=meta$analysis OR TS=cohort OR TS=cross$over OR TS=case$control OR TS=prospective OR TS=retrospective OR TS=longitudinal OR TS=follow$up OR TS=dose$response) AND

(TS=risk OR TS=”odds ratio” OR TS=”cross$product ratio” OR TS=”hazard$ ratio”) AND

DOP=1970-01-01/2022-07-31 NOT

(TS=animal$ NOT TS=human$)

Breast cancer

(TS=(smok* NEAR/4 exposure) OR TS=second$hand OR TS=”environmental tobacco” OR (TS=”tobacco smok* pollution” OR TS=”tobacco smok*”) OR TS=”cigarette smok*” OR TS=”passive smok*” OR TS=”involuntary smok*” OR TS=”parental smok*” OR TS=”maternal smok*”) AND

(TS=”breast cancer$” OR TS=”breast neoplasm$” OR TS=”mammary cancer$” OR TS=”breast malignant neoplasm$” OR TS=”mammary carcinoma$” OR TS=”breast carcinoma$” OR TS=”mammary neoplasm$” OR TS=”breast tumo$r$” OR TS=”mammary tumo$r$” OR TS=”cancer$ of the breast” OR TS=”neoplasm$ of the breast” OR TS=”tumo$r$ of the breast”) AND

(TS=”systematic review” OR TS=meta$analysis OR TS=cohort OR TS=cross$over OR TS=case$control OR TS=prospective OR TS=retrospective OR TS=longitudinal OR TS=follow$up OR TS=dose$response) AND
(TS=risk OR TS=”odds ratio” OR TS=”cross$product ratio” TS=”hazard$ ratio”) AND

DOP=1970-01-01/2022-07-31 NOT

(TS=animal$ NOT TS=human$)

<u>Tracheal, bronchus, and lung cancer</u>

(TS=(smok* NEAR/4 exposure) OR TS=second$hand OR TS="environmental tobacco" OR (TS="tobacco smok* pollution" OR TS="tobacco smok*") OR TS="cigarette smok*" OR TS="passive smok*" OR TS="involuntary smok*" OR TS="parental smok*" OR TS="maternal smok*") AND

(TS="lung neoplasm" OR TS="lung neoplasms" OR TS="lung cancer" OR TS="lung cancer" OR TS="pulmonary neoplasm" OR TS="pulmonary neoplasms" OR TS="pulmonary cancer" OR TS="pulmonary cancers" OR TS="lung malignant neoplasm" OR TS="lung malignant neoplasms" OR TS="pulmonary malignant neoplasm" OR TS="pulmonary malignant neoplasms" OR TS="pulmonary carcinoma" OR TS="pulmonary carcinomas" OR TS="lung carcinoma" OR TS="lung carcinomas" OR TS="lung tumo$r" OR TS="lung tumo$rs" OR TS="pulmonary tumo$r" OR TS="pulmonary tumo$rs" OR TS="cancer of the lung" OR TS="cancers of the lung" OR TS="neoplasm of the lung" OR TS="neoplasms of the lung" OR TS="tumo$r of the lung" OR TS="tumo$rs of the lung" OR TS="bronch* cancer" OR TS="bronch* cancers" OR TS="bronch* neoplasm" OR TS="bronch* neoplasms" OR TS="bronch* malignant neoplasm" OR TS="bronch* malignant neoplasms" OR TS="bronch* carcinoma" OR TS="bronch* carcinomas" OR TS="bronch* tumo$r" OR TS="bronch* tumo$rs" OR TS="cancer of the bronch*" OR TS="cancers of the bronch*" OR TS="neoplasm of the bronch*" OR TS="neoplasms of the bronch*" OR TS="tumo$r of the bronch*" OR TS="tumo$rs of the bronch*" OR TS="trache* cancer" OR TS="trache* cancers" OR TS="trache* neoplasm" OR TS="trache* neoplasms" OR TS="trache* malignant neoplasm" OR TS="trache* malignant neoplasms" OR TS="trache* carcinoma" OR TS="trache* carcinomas" OR TS="trache* tumo$r" OR TS="trache* tumo$rs" OR TS="cancer of the trachea" OR TS="cancers of the trachea" OR TS="neoplasm of the trachea" OR TS="neoplasms of the trachea") AND

(TS="systematic review" OR TS=meta$analysis OR TS=cohort OR TS=cross$over OR TS=case$control OR TS=prospective OR TS=retrospective OR TS=longitudinal OR TS=follow$up OR TS=dose$response) AND

(TS=risk OR TS="odds ratio" OR TS="cross$product ratio" OR TS="hazard$ ratio") AND

DOP=1970-01-01/2022-07-31 NOT

(TS=animal$ NOT TS=human$)

<u>Otitis media</u>

(TS=(smok* NEAR/4 exposure) OR TS=second$hand OR TS="environmental tobacco" OR (TS="tobacco smok* pollution" OR TS="tobacco smok*") OR TS="cigarette smok*" OR TS="passive smok*" OR TS="involuntary smok*" OR TS="parental smok*" OR TS="maternal smok*") AND

(TS="otitis media" OR TS=otitis OR TS="middle ear infection" OR TS="middle ear disease" OR TS="ear infection" OR TS="ear disease") AND

(TS=”systematic review” OR TS=meta$analysis OR TS=cohort OR TS=cross$over OR TS=case$control OR TS=prospective OR TS=retrospective OR TS=longitudinal OR TS=follow$up OR TS=dose$response) AND

(TS=risk OR TS=”odds ratio” OR TS=”cross$product ratio” OR TS=”hazard$ ratio”) AND

DOP=1970-01-01/2022-07-31 NOT

(TS=animal$ NOT TS=human$)

Asthma

(TS=(smok* NEAR/4 exposure) OR TS=second$hand OR TS=”environmental tobacco” OR (TS=”tobacco smok* pollution” OR TS=”tobacco smok*”) OR TS=”cigarette smok*” OR TS=”passive smok*” OR TS=”involuntary smok*” OR TS=”parental smok*” OR TS=”maternal smok*”) AND

TS=asthma AND

(TS=”systematic review” OR TS=meta$analysis OR TS=cohort OR TS=cross$over OR TS=case$control OR TS=prospective OR TS=retrospective OR TS=longitudinal OR TS=follow$up OR TS=dose$response) AND

(TS=risk OR TS=”odds ratio” OR TS=”cross$product ratio” OR TS=”hazard$ ratio”) AND

DOP=1970-01-01/2022-07-31 NOT

(TS=animal$ NOT TS=human$)

Chronic obstructive pulmonary disease

(TS=(smok* NEAR/4 exposure) OR TS=second$hand OR TS=”environmental tobacco” OR (TS=”tobacco smok* pollution” OR TS=”tobacco smok*”) OR TS=”cigarette smok*” OR TS=”passive smok*” OR TS=”involuntary smok*” OR TS=”parental smok*” OR TS=”maternal smok*”) AND

(TS=”chronic obstructive pulmonary disease” OR TS=COPD OR TS=emphysema OR TS=”chronic bronchitis”) AND

(TS=”systematic review” OR TS=meta$analysis OR TS=cohort OR TS=cross$over OR TS=case$control OR TS=prospective OR TS=retrospective OR TS=longitudinal OR TS=follow$up OR TS=dose$response) AND

(TS=risk OR TS=”odds ratio” OR TS=”cross$product ratio” OR TS=”hazard$ ratio”) AND

DOP=1970-01-01/2022-07-31 NOT

(TS=animal$ NOT TS=human$)

Section 1.2: Inclusion and exclusion criteria

Studies were screened on the basis of pre-determined inclusion and exclusion criteria. These criteria were readily available for all reviewers to consult and were described in detail to the research team prior to screening.

Inclusion criteria:

- Publication date: Study was published on or after January 1, 1970.
- Publication published in English.
- Study design: Study uses a prospective cohort, retrospective cohort, case-cohort, case-control, nested case-control, or case-crossover study design.
- Diverse study sample: Study subjects are not from a highly specific subpopulation (e.g., cancer survivors or diabetes patients) that would more likely than not interfere with the generalizability of the findings.
- Acceptable study definition of risk: Study reports current or ever postnatal secondhand smoke exposure at home, work, or at a broader broader/unspecified location (e.g., any location) among the non-smoker, never smoker and/or former smoker population, with a dichotomous exposure measurement. Any source of exposure was accepted (e.g., maternal, paternal, spouse, any). Studies measuring exposure through direct self-report were accepted, as well as those using a proxy of exposure (e.g., living with a smoker).
- Acceptable study definition of outcome: Study reports the outcome of interest relative to our pre-determined definition of the same outcome, in whole or in part. Both gold-standard outcome definitions and alternative were accepted for inclusion (Table S1).
- Report of relative measure of risk: An effect size or study metadata, such as sample sizes or numbers of cases and controls to produce a crude effect size, is reported for the relationship between the secondhand smoke exposure and the outcomes of interest.
- Minimum required data available: Data are reported to fill in all required fields in the extraction form (Table S2).
- Non duplicate study: The study has not had the same data extracted previously from another study.

Exclusion criteria:

- Publication date: Study was published before January 1, 1970.
- Irrelevant study design: Study does not use a design from the family of cohort and case-control study designs.
- Highly specific subpopulation: Study focuses on a very specific subpopulation (e.g., cancer survivors or diabetes patients) that would more likely than not interfere with the generalizability of the findings.
- Non secondhand smoke exposure: Study does not specifically report results on secondhand smoke exposure (e.g., reports the risk associated with active smoking).

- Undesired definition: Study reports exposure to prenatal exposure to tobacco smoke only (i.e., fetus is exposed during pregnancy by smoking mother or others); Study only reports exposure to secondhand smoke in specific settings other than home or work (i.e., work or home exposure is not reported nor the study reports on broad locations of exposure in which home or work are likely to be included). Study reports exposure measured in a continuous or categorical scale. We excluded any study only reporting on former exposure. For IHD, Stroke, breast and lung cancers, COPD, and type 2 diabetes we excluded studies only reporting exposure during the childhood.
- No report of relative measure of risk: No effect sizes or study metadata are reported for the relationship between the risk and the outcome of interest.
- Minimum required data not present: Not enough data are reported to fill out the required fields in the extraction form.
- Duplicate study: The data reported from a study has been extracted from the same study in another report.

Section 1.3: Outcome definitions

The outcomes examined in this review were defined in accordance with the health outcome definitions used in the Global Burden of Disease study.

Table S1. Definitions of nine included health outcomes

Cause Name	Definition
Ischemic heart disease	• *Acute myocardial infarction (MI):* Definite and possible MI according to the third universal definition of myocardial infarction<br>• When there is clinical evidence of myocardial necrosis in a clinical setting consistent with myocardial ischaemia OR<br>• Detection of a rise and/or fall of cardiac biomarker values and with at least one of the following:<br>• Symptoms of ischaemia,<br>• New or presumed new ST-segment-T wave changes or new left bundle branch block,<br>• Development of pathological Q waves in the ECG,<br>• Imaging evidence of new loss of viable myocardium or new regional wall motion abnormality, or<br>• Identification of an intracoronary thrombus by angiography or autopsy<br>• Sudden (abrupt) unexplained cardiac death, involving cardiac arrest or no evidence of a non-coronary cause of death<br>• Prevalent MI is considered to last from the onset of the event to 28 days after the event and is divided into an acute phase (0-2 days) and subacute (3-28 days)<br>• *Chronic IHD:*<br>• Angina; clinically diagnosed stable exertional angina pectoris or definite angina pectoris according to the Rose Angina Questionnaire, physician diagnosis, or taking nitrate medication for the relief of chest pain<br>• Asymptomatic ischaemic heart disease following myocardial infarction; survival to 28 days following incident MI. The GBD study does not use estimates based on ECG evidence for prior MI, due to its limited specificity and sensitivity.<br>• Also called coronary heart disease or coronary artery disease or atherosclerotic heart disease.

Table S1. Definitions of nine included health outcomes

Cause Name	Definition
	ICD-10 codes: I20-I21.6, I21.9-I25.9, Z82.4-Z82.49, I20-I25.9<br><br>ICD-9 codes: 410-414.9, V17.3
Stroke	Stroke was defined according to WHO criteria of rapidly developed clinical signs of focal disturbance of cerebral function lasting > 24 hours or leading to death. Strokes are categorized as ischemic, intracerebral, and subarachnoid events, depending on whether they are caused by a blockage or rupture of the blood vessels in the brain.<br><br>• Ischemic stroke: An episode of neurological dysfunction due to focal cerebral, spinal, or retinal infarction; also known as acute infarction, atherothrombotic stroke, cerebral infarction, embolic stroke, thrombotic stroke or occlusion of cerebral arteries.<br>• Intracerebral stroke: Stroke with a focal collection of blood in the brain not due to trauma; also known as intracerebral hematoma or intraparenchymal hemorrhage.<br>• Subarachnoid hemorrhage: Non-traumatic stroke due to bleeding into the subarachnoid space of the brain.<br><br>ICD-10 codes: I60, I61, I63, I69.0<br><br>ICD-9 codes: 430-434
Type 2 Diabetes mellitus	Type 2 diabetes is a chronic condition, mostly in adults, where the body forms a resistance to insulin or the pancreas stops producing enough insulin. GBD defines type 2 diabetes as fasting plasma glucose ≥7 mmol/L (126 mg/dL) or those currently treated with drugs or insulin, where fasting plasma glucose is measured from glycated hemoglobin (HbA1c), oral glucose tolerance test (OGTT), or post-prandial glucose test (PPG).<br><br>ICD-10 codes: E08-E08.11, E08.3-E08.9, E12-E12.1, E12.3-E13.11, E13.3-E14.1, E14.3-E14.9, R73-R73.9<br><br>ICD-9 codes: 249-249.31, 249.5-249.91, 362.01-362.07, 790.2-790.29
Lower respiratory infections	This cause incorporates death and disability resulting from lower respiratory infections, including clinician-diagnosed and self-reported cases of pneumonia and bronchiolitis, including self-reported symptoms of acute lower respiratory infections including cough with difficulty breathing, fever, and symptoms in the chest.<br><br>ICD-10 codes: A48.1, J09-J22, J85.1, P23-P23.9, U04<br><br>ICD-9 codes: 073.0-073.6, 079.82, 466-469, 480-489, 513.0, 770.0
Breast cancer	This cause includes death and disability resulting from invasive neoplasms of the breasts, comprised of the connective tissue, glandular tissue, and soft parts.

Table S1. Definitions of nine included health outcomes	
Cause Name	Definition
	ICD-10 codes: C50-C50.9, D05-D05.9, D24-D24.9, D48.6, D49.3, Z12.3-Z12.39, Z80.3, Z85.3, Z86<br><br>ICD-9 codes: 174-175.9, 217-217.8, 233.0, 238.3, 239.3, 610-610.9, V10.3, V16.3
Tracheal, bronchus, and lung cancer	This cause includes death and disability resulting from invasive neoplasms of the trachea, bronchi, and lungs.<br><br>ICD-10 codes: C33-C34.9, D02.1-D02.3, D14.2-D14.3, D38.1, Z12.2, Z80.1- Z80.2, Z85.1-Z85.20<br><br>ICD-9 codes: 162-162.9, 209.21, 212.2-212.3, 231.1-231.2, 235.7, V10.1-V10.20, V16.1- V16.2, V16.4-V16.40
Otitis media	Otitis media is an infection of the middle ear space, including acute otitis media, chronic otitis media, and chronic suppurative otitis media. Study definitions that do not specify middle ear infection or includes otitis media with effusion, also known as "glue ear", are excluded from the present analyses.<br><br>ICD-10 codes: H65-H75.83<br><br>ICD-9 codes: 381-384.9
Asthma	Asthma is a chronic lung disease characterized by reversible airway obstruction due to spasms and secretions in the bronchi usually resulting from an allergic reaction or hypersensitivity and causing difficulty in breathing. Cases are defined as having a doctor's diagnosis and wheezing in the past year. Alternative case definitions include: Self-reported asthma in the past year, self-reported asthma ever, a doctor's diagnosis in the past year, and wheezing in the past year. The time component of the case definition is exact.<br><br>ICD-10 codes: J45, J46<br><br>ICD-9 codes: 493
Chronic obstructive pulmonary disease	COPD is a disease characterized by persistent respiratory symptoms and airflow limitation due to airway and/or alveolar abnormalities, usually caused by significant long-term exposure to noxious particles or gases. COPD includes emphysema and chronic bronchitis and may also be known as chronic obstructive lung disease.<br><br>ICD-10 codes: J41-J42.4, J43-J44.9<br><br>ICD-9 codes: 491-492.9, 496-499

Section 1.4: PRISMA diagrams

Figure S1: PRISMA 2020 flow diagram for secondhand smoke and ischemic heart disease

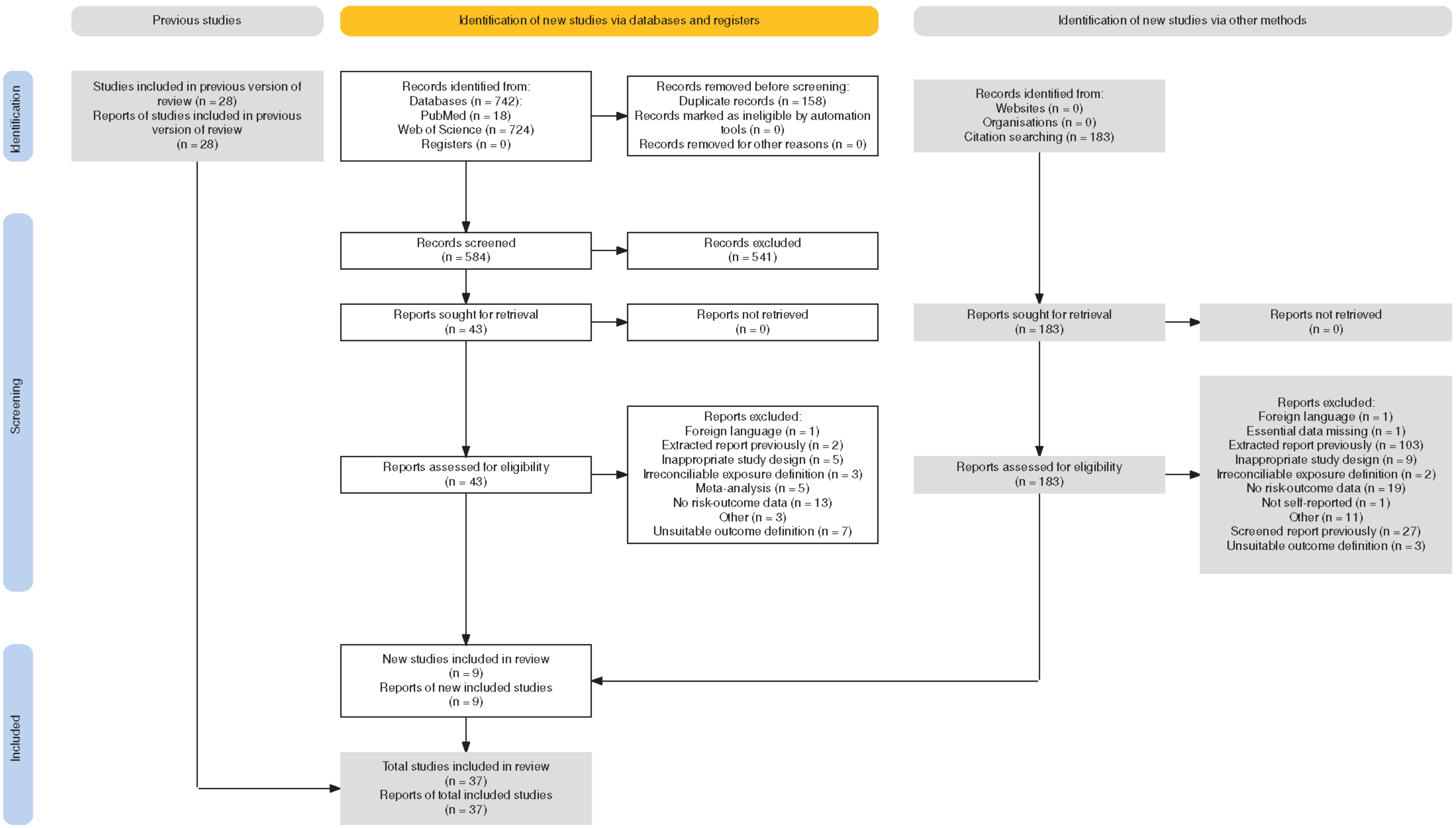

Figure S2: PRISMA 2020 flow diagram for secondhand smoke and stroke

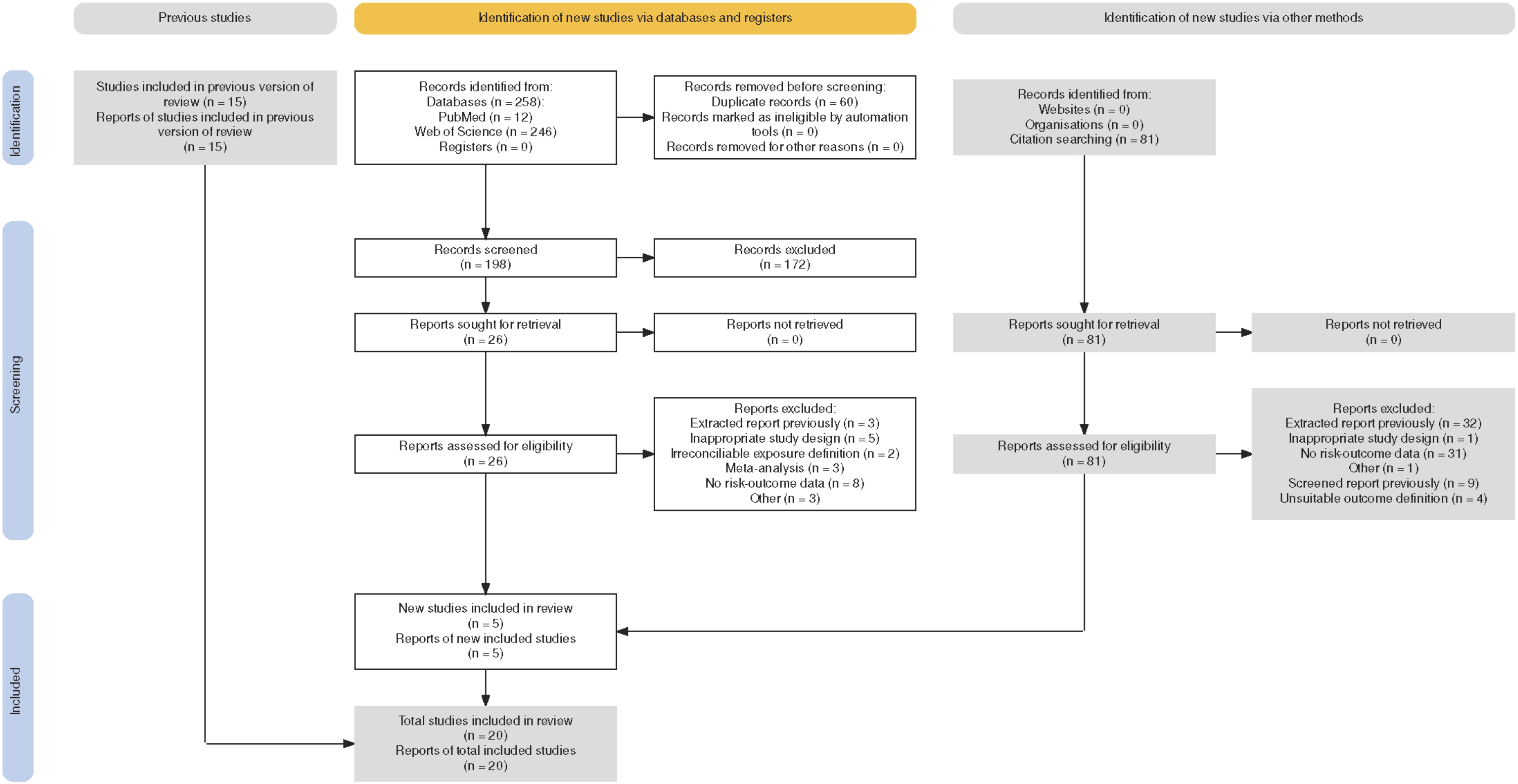

Figure S3: PRISMA 2020 flow diagram for secondhand smoke and tracheal, bronchus, and lung cancer

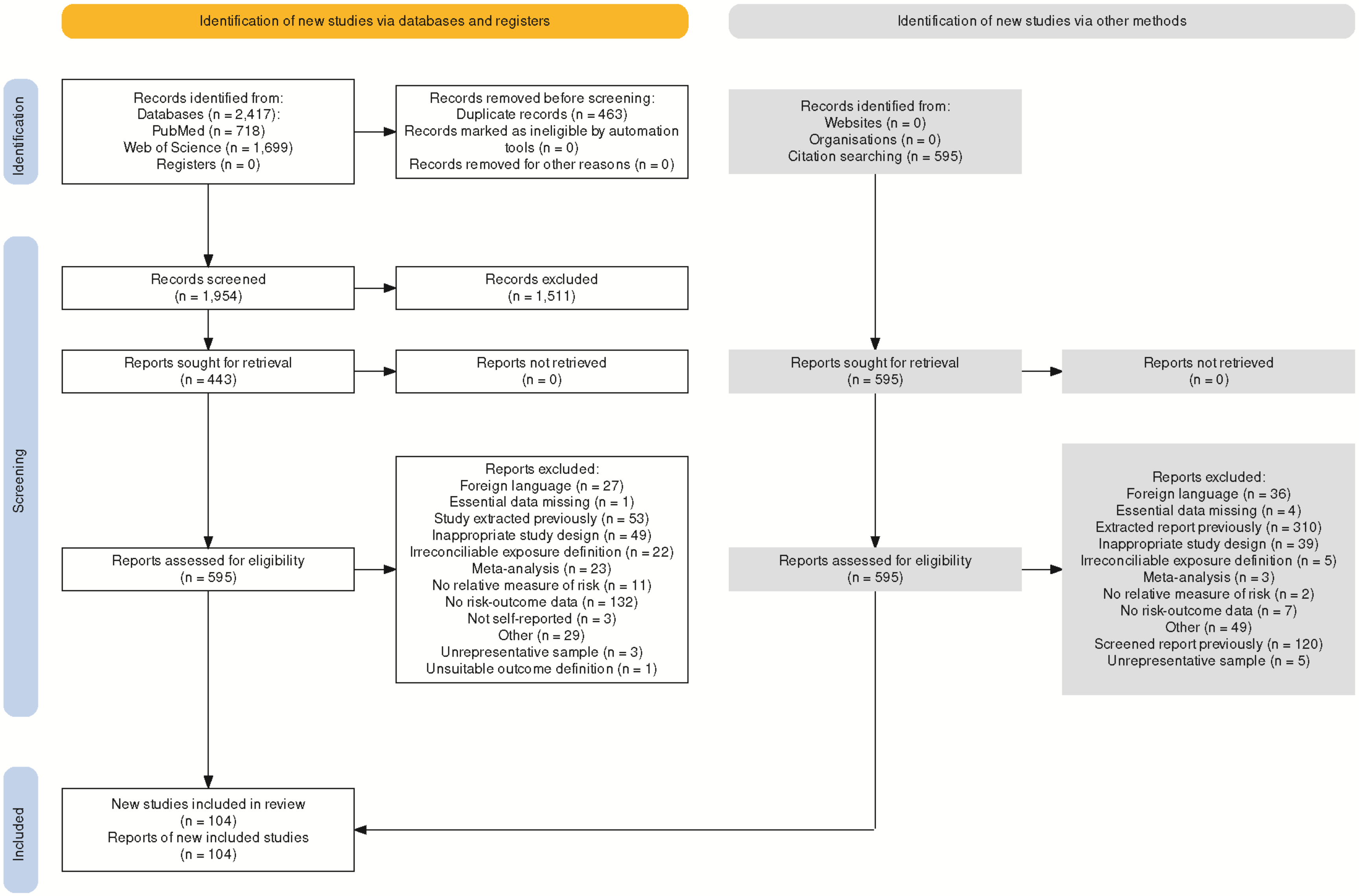

Figure S4: PRISMA 2020 flow diagram for secondhand smoke and breast cancer

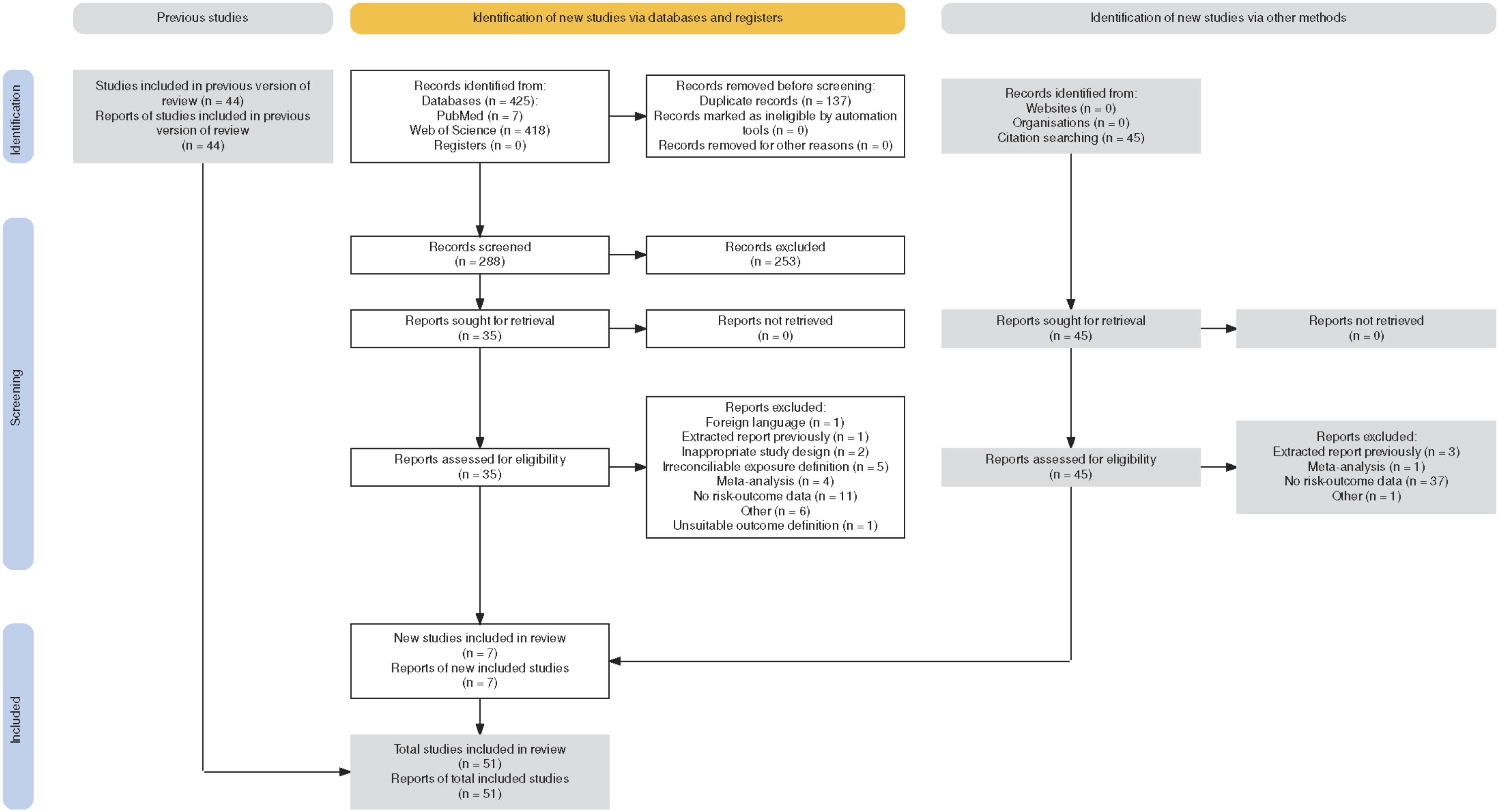

Figure S5: PRISMA 2020 flow diagram for secondhand smoke and asthma

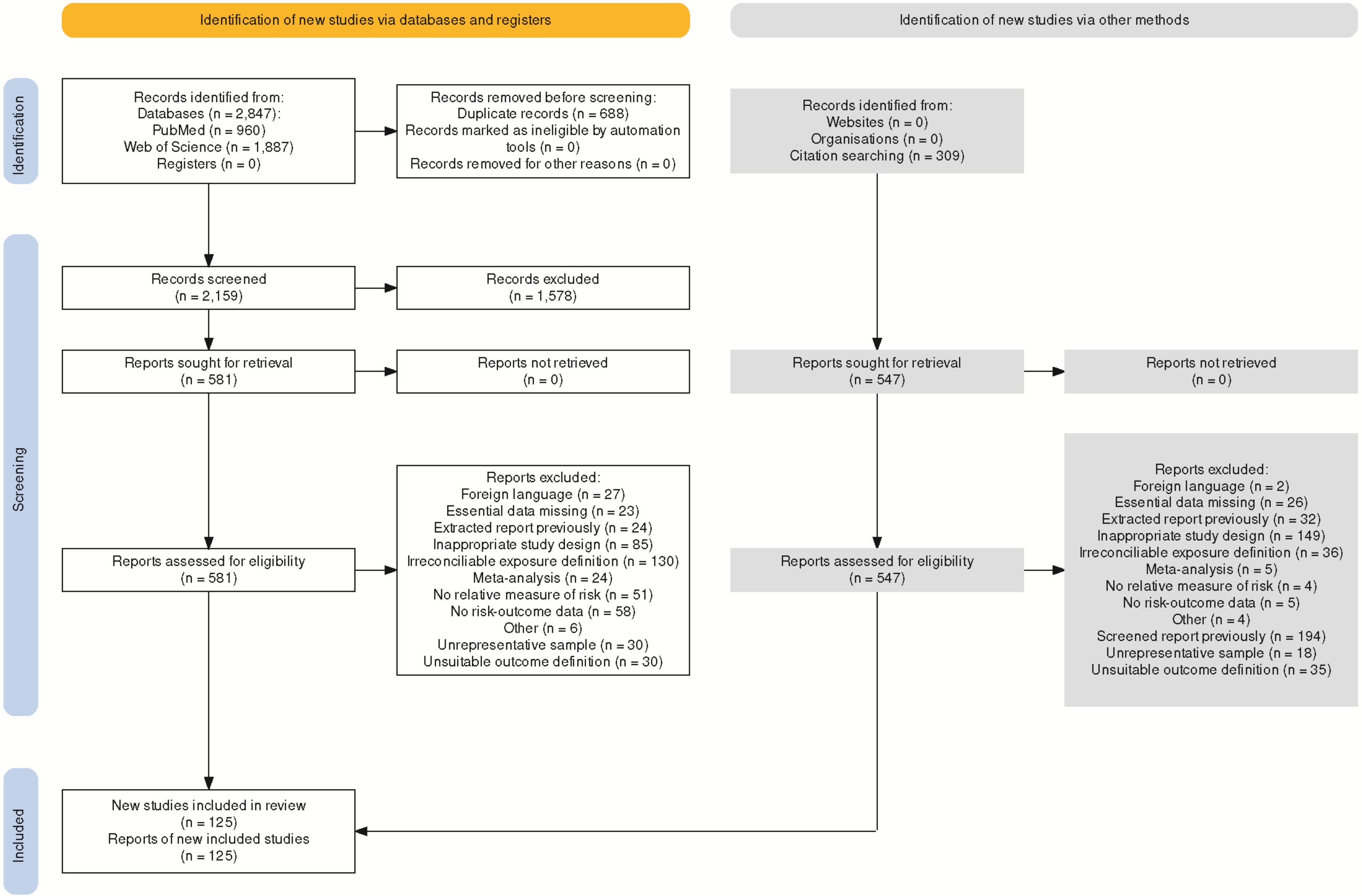

Figure S6: PRISMA 2020 flow diagram for secondhand smoke and lower respiratory infections

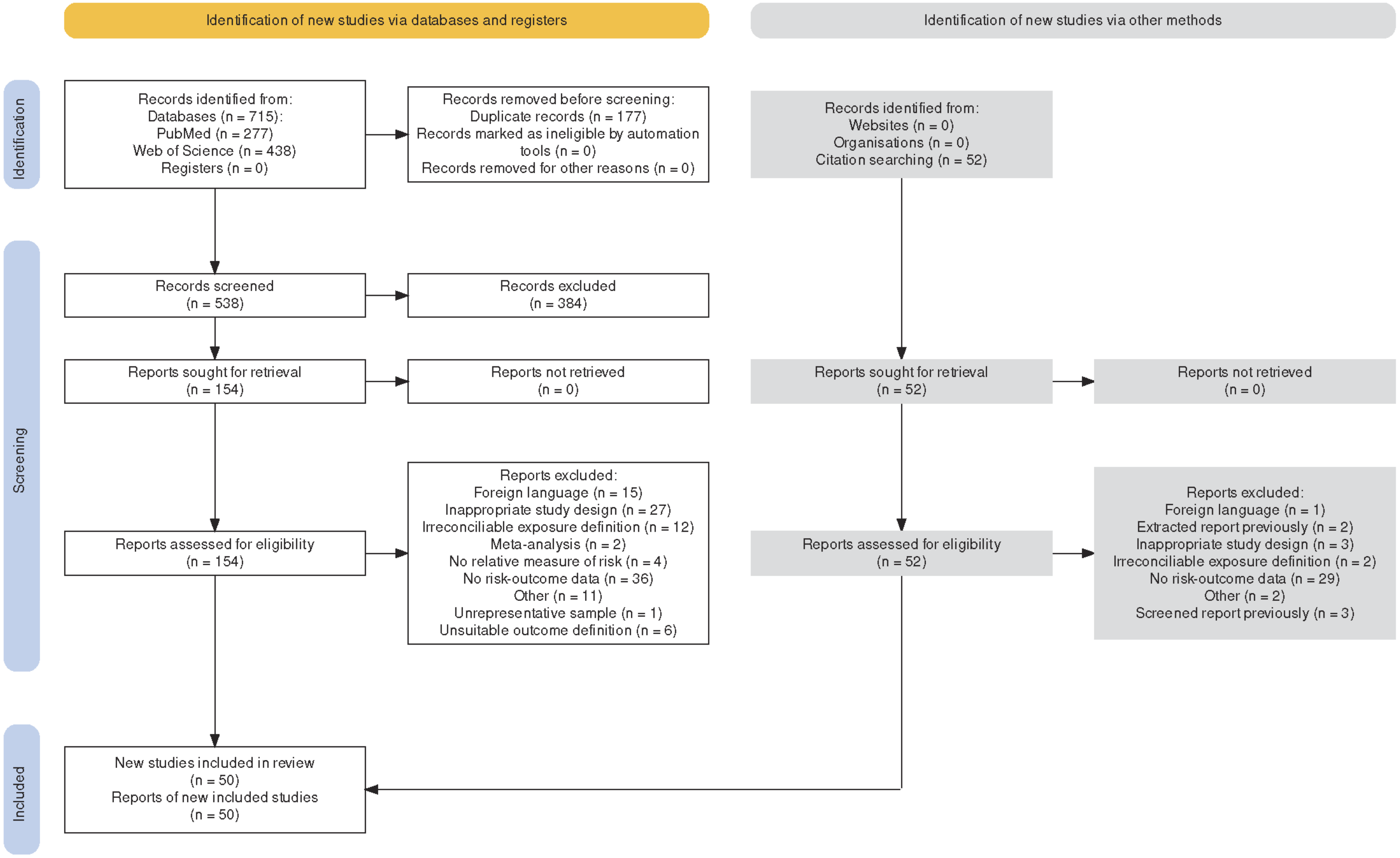

Figure S7: PRISMA 2020 flow diagram for secondhand smoke and chronic obstructive pulmonary disease

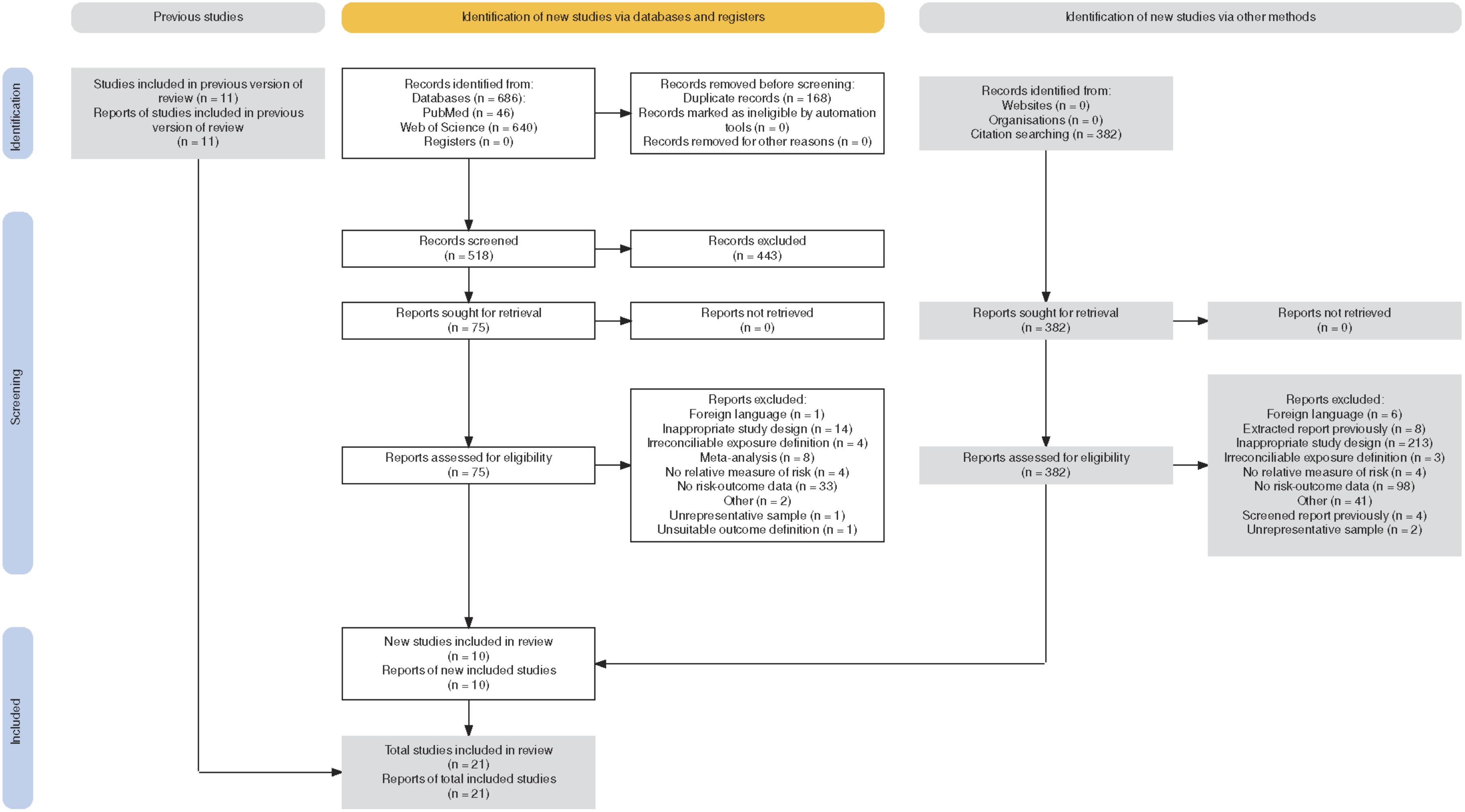

Figure S8: PRISMA 2020 flow diagram for secondhand smoke and type 2 diabetes mellitus

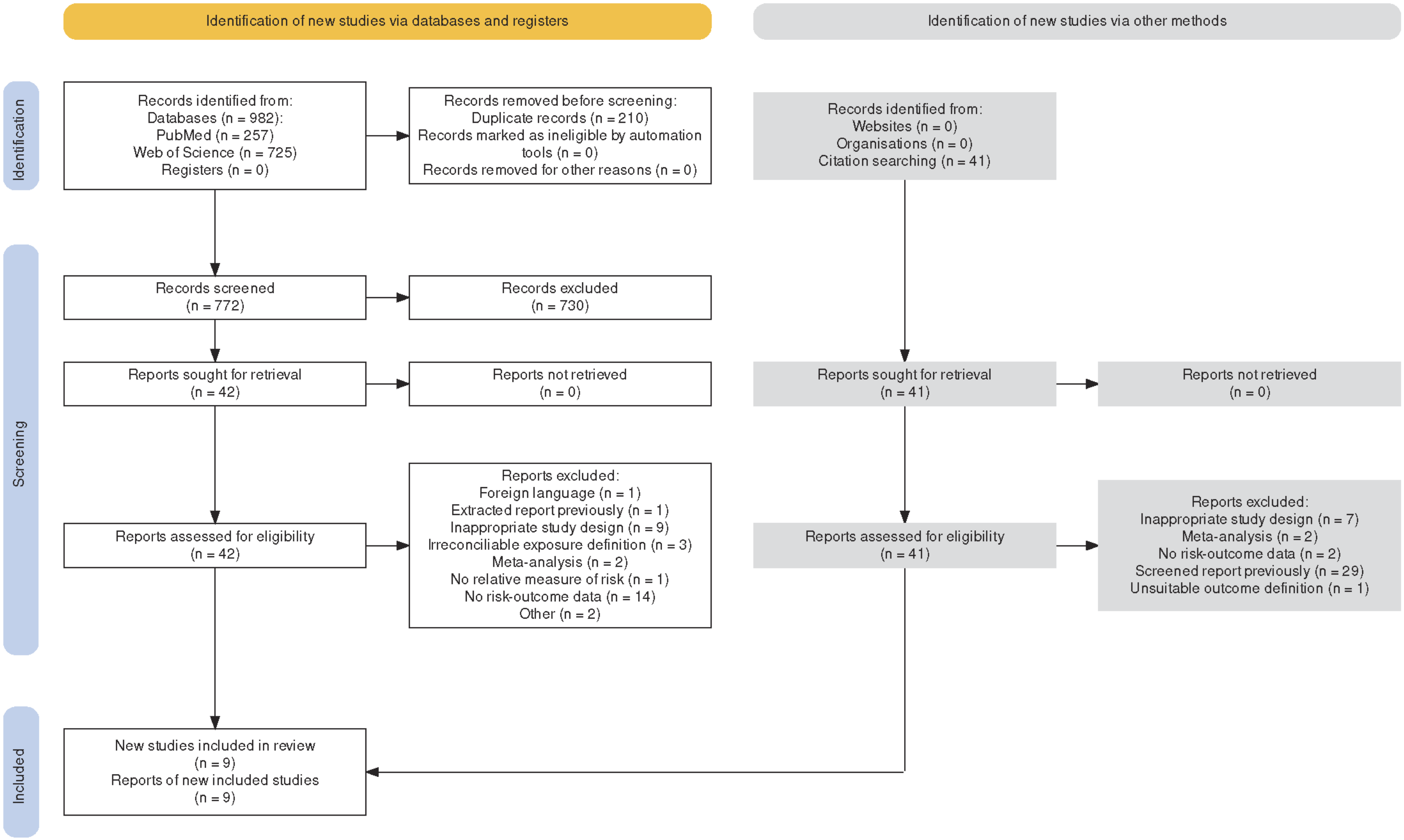

Figure S9: PRISMA 2020 flow diagram for secondhand smoke and otitis media

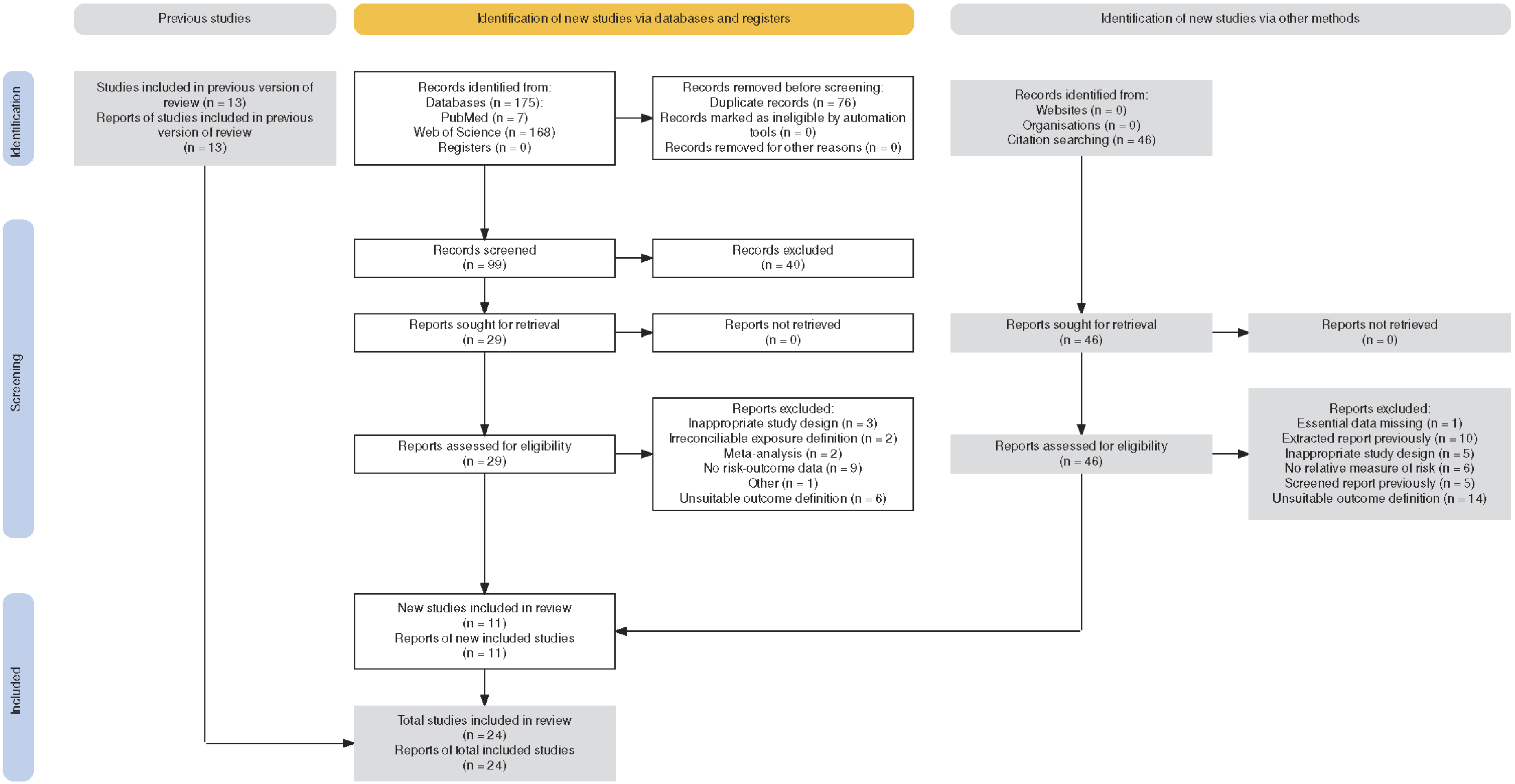

Section 1.5: Data extraction

The extracted variables are described in the template below.

Table S2. Data Extraction Template

Category	Extraction Field Name	Field Type	Definition
Source	extractor	Free text	UW NetID of person completing extraction
	nid	Numerical	Unique identifier for the data created for the Global Health Data Exchange (GHDx)
	underlying_nid	Numerical	NID that identifies the underlying data source; File NIDs will cause errors, only NIDs in the GHDx database are accepted.
	field_citation_value	Free text	Citation of NID pulled from the GHDx
	file_path	Free text	Full file path for the article where it is stored internally in the GHDx database.
	source_type	Categorical	The type of data collection used to evaluate the risk factor.<br>• For cohort studies which conduct data collection of the risk multiple times during follow-up, select 'Survey - longitudinal'.<br>• For cohort studies which conduct data collection of the risk at baseline only and case-cohort studies, select 'Survey - cohort'.<br>• For the family of case-control study designs, select 'Survey - other/unknown'.<br>• If a study was originally cross-sectional, but the study design changed through analysis (e.g., ran a case-control study based on the data from a cross-sectional survey or conducted outcome follow-up of cross-sectional survey participants), select 'Survey - cross-sectional'.
Location & Representativeness	location_name	Categorical	The name of the country or subnational location where the study was conducted. If the effect size is reported for multiple subnational locations, extract the location higher up in the location hierarchy that encompasses all the locations.
	location_id	Numerical	Unique identifier for location based on GBD identifiers; autopopulated from location_name in extraction template
	rep_geography	Binary	Does the geographic coverage of the study match or nearly match that of the GBD geography? 1=yes, 0=no
	rep_population	Binary	Indicate whether the target population of the study is more broadly representative of the population at the study geography. 1=yes, 0=no
	site_memo	Free text	Copy and paste verbatim from the source all the information regarding the location of data collection. Please include facility names (hospitals, clinics, etc.) and street addresses, GPS coordinates, etc., if provided.
	rep_children	Binary	Indicate whether the target population of the study is comprised of mostly or only children. Individuals aged 15 years of age or older or considered adults.
	rep_prevalent_disease	Binary	Is the study aiming to evaluate the risk or mortality of people who have already developed the outcome? 1=yes 0=no

Table S2. Data Extraction Template

Category	Extraction Field Name	Field Type	Definition
Study Population	year_start	Numerical	Year the study was started.
	year_end	Numerical	Year the study was finished (including most recent follow up).
	year_issue	Binary	Flag for any issues with study year that are identified during extraction; 1 = issue flagged for modeler; 0 = no issue flagged.
	age_start	Numerical	Low end of range of ages in study sample used for the effect size.
	age_end	Numerical	High end of range of ages in study sample used for the effect size.
	age_mean	Numerical	Mean of ages in study sample used for the effect size.
	age_sd	Numerical	Standard deviation of ages represented in study sample used for the effect size.
	age_issue	Binary	1 = issue flagged for modeler; 0 = no issue flagged
	age_demographer	Binary	1 = person has had their "age" birthday and not yet their "age+1" birthday; 0 = exact age is used by the study. This field only applies to the value in the age_end field.
	sex	Categorical	Sex identifier: Male, Female, Both
	percent_male	Numerical	Percentage of the study sample that is male (decimal from 0-1)
	sex_issue	Binary	Issues with sex identified during extraction; 0 = no issue flagged; 1 = issue flagged for modeler
Study Design	design	Categorical	Specifies the design of the study.
	study_name	Free Text	The name of the study (e.g., Nurses' Health Study), if provided
	occupational_study	Binary	Indicate whether a study analyzes risks of an outcome within certain occupations. 1 = if a study analyzes risks within occupations 0 = otherwise
	occupational_desc	Free text	If the study is occupational, list the occupations that are assessed for the risk of developing an outcome.
Risk	risk	Categorical	Risk factor selected from a dropdown menu. It should always be "Secondhand smoke".
	rei	Categorical	The human and machine-readable name associated with the risk factor in the GBD.
	risk_def	Free text	Provide a brief description of the risk as reported in the study.
	risk_mapping	Categorical	Select the scope of the study definition of the risk in relation to the GBD definition of the risk for a particular effect size.
Exposure	exp_assess_level	Categorical	Level at which the exposure was assessed.
	exp_instrument	Free text	Specifies the name of the exposure assessment instrument. Unless a specifically-named instrument is reported, leave blank for secondhand smoke.
	exp_assess_period	Categorical	Frequency of exposure assessment
	exp_assess_num	Numerical	If multiple exposure assessments, specify the number of times that exposure was assessed (excluding baseline).
	exp_method_1	Categorical	Specifies the method of exposure assessment.
	exp_method_2	Categorical	Specifies the secondary method of exposure assessment if there are 2 or more.
	exp_method_3	Categorical	Specifies the tertiary method of exposure assessment if there are more than 2.

Table S2. Data Extraction Template

Category	Extraction Field Name	Field Type	Definition
	exp_recall_period	Categorical	The unit of exposure recall used in data collection for self-reported studies. Examples are "days," "weeks," "months," "years," or "point." If a source does not specify or indicate a recall (explicitly or implicitly), assume that the recall is a point estimate and select 'Point'.
	exp_recall_period_value	Numerical	The corresponding integer with "exp_recall_period." For example, if the study said the recall period was 4 weeks, enter 4 in exp_recall_period_value, and 'weeks' in the field exp_recall_period.
	exp_recall_period_other	Free text	If 'other' was selected in exp_recall_period, describe the exposure recall period that the study specified (e.g., recall of exposure from 12 to 18 years).
	exp_type	Categorical	Which form of the exposure was included in relative risk estimation analysis?
Outcome	outcome	Categorical	Outcome of interest selected from a drop-down menu.
	acause	Categorical	The machine-readable name for causes associated with the outcome in the GBD.
	outcome_components	Free text	Denotes the subtype of the outcome, if applicable. If a study is assessing chronic suppurative otitis media, enter this subtype in this column.
	outcome_def	Free text	Brief description of the outcome definition as reported in the study.
	asthma_outcome_type	Categorical	Specify the asthma outcome described in the study and applied to the associated effect size. Value is one of 'Asthma only', 'Asthma and wheezing', or 'Wheezing only'.
	asthma_recall	Categorical	Specify the time period of recall when the outcome was determined. Value is one of 'Past 12 months', 'Ever', 'Other', or 'Unspecified'.
	outcome_type	Categorical	Field to specify if outcome definition incidence of or mortality from a disease endpoint
	outcome_assess_1	Categorical	Method of outcome assessment: Specifies the method of assessment of the study outcome. If more than 1 are appropriate, enter additional methods in the next column labeled outcome_assess_2.
	outcome_assess_2	Categorical	Method of outcome assessment: Specifies a method of assessment of the study outcome. If more than 2 are appropriate, enter additional methods in the next column labeled outcome_assess_3.
	outcome_assess_3	Categorical	Method of outcome assessment: Specifies a method of assessment of the study outcome.
Follow up	duration_fup_measure	Categorical	Type of follow up measure (i.e., mean, median, max, min).
	duration_fup_units	Categorical	Units of follow up duration
	value_of_duration_fup	Numerical	The length of participant follow-up.
Confounders Controlled For	confounders_age	Binary	1 = the study controls for age in the relative risk estimation analysis; 0= age is not controlled for
	confounders_sex	Binary	1 = the study controls for sex in the relative risk estimation analysis; 0 = sex is not controlled for
	confounders_education	Binary	1 = the study controls for education in the relative risk estimation analysis; 0 = education is not controlled for
	confounders_income	Binary	1 = the study controls for income in the relative risk estimation analysis; 0 = income is not controlled for

Table S2. Data Extraction Template

Category	Extraction Field Name	Field Type	Definition
	confounders_smoking	Binary	1 = the study controls for smoking in the relative risk estimation analysis; 0 = smoking is not controlled for
	confounders_alcohol_use	Binary	1 = the study controls for alcohol in the relative risk estimation analysis; 0 = alcohol is not controlled for
	confounders_physical_activity	Binary	1 = the study controls for physical activity in the relative risk estimation analysis; 0 = physical activity is not controlled for
	confounders_dietary_components	Binary	1 = the study controls for dietary components in the relative risk estimation analysis; 0 = dietary components are not controlled for
	confounders_bmi	Binary	1 = the study controls for BMI in the relative risk estimation analysis; 0 = BMI is not controlled for
	confounders_hypertension	Binary	1 = the study controls for hypertension in the relative risk estimation analysis; 0 = hypertension is not controlled for
	confounders_diabetes	Binary	1 = the study controls for diabetes in the relative risk estimation analysis; 0 = diabetes is not controlled for
	confounders_hypercholesterolemia	Binary	1 = the study controls for hypercholesterolemia in the relative risk estimation analysis; 0 = hypercholesterolemia is not controlled for
	confounders_marital_status	Binary	1 = the study controls for marital status in the relative risk estimation analysis; 0 = marital status is not controlled for
	confounders_race_ethnicity	Binary	1 = the study controls for race/ethnicity in the relative risk estimation analysis; 0 = race/ethnicity are not controlled for
	confounders_other	Free Text	List other confounders that not listed but that the effect size is controlled for.
Model flagging	most_adj_model	Binary	1 = the model controls for the most confounders and is the most adjusted for model; 0 = The model is not the most adjusted for model
	least_adj_model	Binary	1 = the model controls for the most confounders and is the most adjusted for model; 0 = The model is not the most adjusted for model
Exposure level definitions	exp_level_lower_sign	Categorical	Select from the drop-down list a mathematical sign to accompany the numerical lower bound of the categorical range of exposure of the exposed subjects.
	exp_level_lower	Numerical	The relevant numerical lower bound of the categorical range of exposure of the exposed subjects
	exp_level_upper_sign	Categorical	A mathematical sign to accompany the numerical upper bound of the categorical range of exposure
	exp_level_upper	Numerical	The relevant numerical upper bound of the categorical range of exposure of the exposed subjects
	unexp_level_lower_sign	Categorical	A mathematical sign to accompany the numerical lower bound of the categorical range of exposure of the unexposed subjects
	unexp_level_lower	Numerical	The relevant numerical lower bound of the categorical range of exposure of the unexposed subjects
	unexp_level_upper_sign	Categorical	A mathematical sign to accompany the lower or upper bound of categorical exposure for the unexposed subjects

Table S2. Data Extraction Template			
Category	Extraction Field Name	Field Type	Definition
	unexp_level_upper	Numerical	Enter the relevant numerical lower bound of a categorical range of exposure of the unexposed subjects
Effect size location	page_num	Free text	Page number (where you found effect size) from literature, or survey question where you found effect size.
	table_num	Free text	Table number where you find relevant effect sizes.
	in_supplement	Binary	Were the relevant effect sizes found in the supplemental section? 1 = yes, 0 = no
Effect Size & Uncertainty	measure	Categorical	The type of effect size being extracted
	effect_size_unit	Categorical	Mathematical space of the effect size.<br>• 'linear': Select this if the extracted effect size is in linear mathematical space. Effect sizes are most often reported in this form. For example, if the reported hazard ratio of exposure to secondhand smoke and developing lung cancer is 1.5, this value is in linear space.<br>• 'logit': Select this if the extracted effect size is in logit mathematical space. This will most likely occur if the model coefficient of a logistic regression is extracted, which is the model associated with case-control studies and produces odds ratios.<br>• 'log': Select this if the extracted effect size is in logarithmic mathematical space. This will most likely occur if the model coefficient of various statistical models (other than logistic regression) is extracted. Generally, relative risks and hazard ratios not in linear space are in log space.
	effect_size_measure	Categorical	Specify the estimate type of the effect size
	mean	Numerical	The effect size estimate
	lower	Numerical	Provide the lower limit of the confidence interval. Enter on a 'per 1' basis. (If the CI is reported as a percent, you must convert to a decimal.) These 3 fields must all be filled in if any of them are filled in: lower, upper, uncertainty_type_value.
	upper	Numerical	Provide the upper limit of the confidence interval. Enter on a 'per 1' basis. (If the CI is reported as a percent, you must convert to a decimal.) These 3 fields must all be filled in if any of them are filled in: lower, upper, uncertainty_type_value.
	uncertainty_type_value	Numerical	This field is required if lower and upper are entered. This column reports the confidence level of the confidence interval (e.g., 95, 90, 99). These 3 fields

Table S2. Data Extraction Template

Category	Extraction Field Name	Field Type	Definition
			must all be filled in if any of them are filled in: lower, upper, uncertainty_type_value.
	standard_error	Numerical	Required if upper and lower uncertainty bounds are not extracted and standard error is reported.
	uncertainty_issue	Binary	Is a measure of uncertainty not reported? 1=yes, 0=no
	effect_size_derived	Binary	Was the effect size calculated (e.g., adjusted to linear space, calculated using an online tool)? 1=yes, no=0
	effect_size_from_image	Binary	Was WebPlotDigitizer or another image processing tool used to extract the effect size? 1=yes, no=0
Effect Size Analysis Details	subgroup_analysis	Binary	Is the effect size from the main analysis (all participants)? (Examples of a sub-analysis include only males, among a specific age group, etc.) 1=yes, 0=no
	subgroup_analysis_free_text	Free text	If a sub-analysis, describe it (e.g., age, sex, etc.)
	effect_size_multi_location	Binary	Is the effect size from a multi-country study and only one effect size is reported for all locations? 1=yes, 0=no
	effect_size_multi_location_s pecify	Categorical	Which geography level is represented by the effect size?
	pooled_cohort	Binary	Is the effect size from a pooled analysis and only a pooled effect size is reported? 1=yes, 0=no
	dose_response	Binary	Does the study support a dose-response relationship between the exposure and the outcome? 1=yes, 0=no
	dose_response_detail	Free text	If '1' was specified in the dose_response field, please specify in this field the type of evidence supporting the dose-response relationship. For example, 'statistically significant p-value for linear trend'.
SHS-specific columns	shs_exp_location	Categorical	Select from the drop-down list the location of SHS exposure, as defined in the survey for that row of extraction.<br>• Values: 'home', 'home only', 'work', 'work only', 'home or work', 'home and work', 'public', 'multiple-or', 'multiple-and', 'other', 'any'.
	shs_exp_location_desc	Free text	Enter a brief description of the location of SHS exposure, if either 'multiple' or 'other' were selected in the shs_exp_location column.<br>Example: If the exposure was SHS at either work or in public, enter 'work or public'. If the exposure was a combination of work and public, enter 'work and public'
	shs_exp_source	Categorical	Select from the drop-down list the source of SHS exposure.<br>• If shs_exp_location is set to 'home', select one from among 'maternal', 'maternal only',

Table S2. Data Extraction Template			
Category	Extraction Field Name	Field Type	Definition
			'paternal', 'paternal only', 'parental', 'parental-one', 'parental-both', 'spouse', 'other family (not spouse)', or 'family (anyone in household)'.<br>• If shs_exp_location is set to a value other than 'home', select 'any' only.
	shs_exp_unit	Free text	Enter the unit of categorical or continuous exposure related to the values filled out in the exposure level columns as reported in the paper for exposed subjects.<br>• 'binary': Exposure is dichotomous, i.e., yes or no<br>• 'cigs/day': Exposure is recorded as equivalents to cigarettes/day smoked by the source of exposure
	shs_exp_temporality	Categorical	Select from the drop-down list the temporality of SHS exposure for the exposed subjects. That is, denote exposed subjects' SHS exposure as current, former, etc.<br>• Values: 'former', 'current', 'never', 'ever', 'adulthood', 'childhood'.<br>Example: If SHS exposure is from a former smoking spouse, select 'former'.
	shs_exp_smoking_status	Categorical	Select from the drop-down list the smoking status of the exposed subjects.<br>• Values: 'former smokers', 'never smokers', 'non-smokers (former and never)', 'unknown', 'any smoking status'.
	shs_unexp_unit	Free text	Enter the unit of categorical or continuous exposure related to the values filled out in the exposure level columns as reported in the paper for unexposed subjects.<br>• 'binary': Exposure is dichotomous, i.e., yes or no<br>• 'cigs/day': Exposure is recorded as equivalents to cigarettes/day smoked by the source of exposure
	shs_unexp_temporality	Categorical	Select from the drop-down list the temporality of SHS exposure for the unexposed subjects. That is, denote unexposed subjects' SHS exposure as current, former, etc.<br>• Values: 'former', 'current', 'never', 'ever', 'adulthood', 'childhood'.<br>Example: If SHS exposure is from a former smoking spouse, select 'former'.
	shs_unexp_smoking_status	Categorical	Select from the drop-down list the smoking status of the unexposed subjects.<br>• Values: 'former smokers', 'never smokers', 'non-smokers (former and never)', 'unknown', 'any smoking status'.
Cohort Sample Details	cohort_person_years_exp	Numerical	Please specify the person years of follow up in the exposed group
	cohort_person_years_unexp	Numerical	Please specify the person years of follow up in the unexposed group

Table S2. Data Extraction Template

Category	Extraction Field Name	Field Type	Definition
	cohort_person_years_total	Numerical	Enter the total person-years of follow-up if person-years of follow up in exposed and unexposed not reported
	cohort_number_events_exp	Numerical	The number of events in the exposed cohort group
	cohort_number_events_unexp	Numerical	Please specify the number of events in the unexposed group
	cohort_number_events_total	Numerical	Enter the total number of events/cases if number of events in exposed and unexposed not reported
	cohort_sample_size_exp	Numerical	Please specify the number of people in the exposed group if person-years of follow up in exposed not reported
	cohort_sample_size_unexp	Numerical	Please specify the number of people in the unexposed group if person-years of follow up in unexposed not reported
	cohort_sample_size_total	Numerical	Please specify the number of people included in the analysis if total person-years of follow up in not reported
	cohort_dropout_rate	Numerical	Dropout rate : Specify the dropout rate (%) at the end of the study. Enter on a "per 1" basis. For example: 23% is entered as .23.
	cohort_dropout_assess	Free text	Specify how dropout rate was defined in the study.
	cohort_exposed_def	Free text	Exposed group definition: Provide a brief description of the exposed group (i.e., the comparison group) as used in estimation of the relative risk
	cohort_exp_unit_rr	Free text	Exposure unit (for continuous risks): Specify the unit of exposure (e.g., grams/day).
	cohort_exp_level_rr	Numerical	Exposure level in the exposed group (for continuous risks): Specify the mean/median level of exposure in the exposed group.
	cohort_unexp_def	Free text	unexposed group definition: Provide a brief description of the unexposed group (i.e., the comparison group) as used in estimation of the relative risk (e.g., never smokers)
	cohort_unexp_unit_rr	Free text	Exposure unit (for continuous risks): Specify the unit of exposure (e.g., grams/day) for the unexposed group
	cohort_unexp_level_rr	Numerical	Exposure level in the unexposed group (for continuous risks): Specify the mean/median level of exposure in the unexposed group.
	cohort_exp_level_dr	Free text	Exposure level in for dose-repose RRs (for continuous risks): If the study reports dose-repose RR, please specify the level of exposure for the reported RR
Case-control Sample Details	cc_community	Binary	Were the controls selected from the community? 1 = yes, 0=no
	cc_cases_exp	Numerical	Number of exposed cases associated with the effect size

Table S2. Data Extraction Template

Category	Extraction Field Name	Field Type	Definition
	cc_controls_exp	Numerical	Number of exposed controls associated with the effect size
	cc_cases_unexp	Numerical	Number of cases in the unexposed group associated with the effect size.
	cc_controls_unexp	Numerical	Number of controls in the unexposed group associated with the effect size.
	cc_cases_total	Numerical	Total number of cases associated with the effect size
	cc_controls_total	Numerical	Total number of controls associated with the effect size
	cc_dropout_rate	Numerical	The dropout rate of the study sample, including non-responsiveness
	cc_dropout_assess	Free text	How the dropout rate was determined by the researchers
	cc_exposed_def	Free text	Exposed group definition: Provide a brief description of the exposed group for which the relative risk is reported
	cc_exp_unit_rr	Free text	For continuous risks, specify the unit of exposure used to define the exposed group.
	cc_exp_level_rr	Numerical	Exposure level in the exposed group (for continuous risks): Specify the mean/median level of exposure in the exposed group.
	cc_unexposed_def	Free text	Unexposed group definition: Provide a brief description of the unexposed group (i.e., the comparison group) as used in estimation of the relative risk
	cc_unexp_unit_rr	Free text	For continuous risks, specify the unit of exposure used to define the unexposed group
	cc_unexp_level_rr	Numerical	Exposure level in the unexposed group (for continuous risks): Specify the mean/median level of exposure in the unexposed group.
	cc_exp_level_dr	Free text	Exposure level in for dose-repose RRs (for continuous risks): If the study reports dose-repose RR, please specify the level of exposure for the reported RR.
Misc.	note_modeler	Free text	Any notes added by the modeler when reviewing the source.
	note_sr	Free text.	Any notes related to the extraction, including assumptions, data adjustments, problems with the source, any other notes that may be relevant, etc.

Section 2: Data Inputs

We extracted our data inputs from peer-reviewed studies identified through the systematic reviews. The data from studies included in our systematic reviews were extracted by one member of the research team using the extraction template described above (Section 1.5). At a minimum, the most and least adjusted relevant effect size and related uncertainty reported for each study exposure-outcome definition pairing and the analytical sample was extracted, along with the metadata for each effect size and the metadata for the study overall. Individual observations that did not match our inclusion criteria and matched our exclusion criteria were not extracted. In certain cases, where an effect size for an exact or preferred secondhand smoke definition was not reported but the number of exposed cases, unexposed cases, exposed controls, and unexposed controls were available, the extractor manually calculated the unadjusted effect size corresponding to the study design and its related uncertainty.

Section 2.1: Study characteristics

Table S3. Summary of study characteristics

Health outcome	Author-Year	Study name	Study design	Location	Sex	Age start	Age end	Exposure assessment	Outcome assessment	Endpoint	Sample size	Exposed	Events	Follow-up (years)	Person-years	Cases	Controls	Control pool	Exposure location	Exposure source	Smoking status
Asthma	Al-Kubaisy 2005	N/A	Prospective cohort	Iraq	Both	6	12	Self-report	Self-report; Physician diagnosis	Incidence	2,262	N/A	N/A	0.75	N/A	644	1,618	Community	Home	Maternal; Paternal	Unspecified
Asthma	Al-Qerem 2016	N/A	Prospective cohort	Egypt	Both	7	12	Self-report	Self-report	Incidence	2,183	N/A	N/A	2	N/A	N/A	N/A	N/A	Home	Family (anyone in household)	Never smokers
Asthma	Arif 2017	N/A	Case-control	Sindh	Both	2	14	Self-report	Physician diagnosis	Morbidity	N/A	N/A	N/A	N/A	N/A	200	200	Community	Home	Parental	Never smokers
Asthma	Aversa 2021	Rochester Epidemiology Project (REP)	Prospective cohort	Minnesota	Both	0	0	Self-report	Administrative medical records or disease registries	Incidence	14,572	1,608	N/A	8.8	N/A	N/A	N/A	N/A	Home	Maternal	Never smokers
Asthma	Azizi 1995	N/A	Case-control	Malaysia	Both	0.083	5	Self-report	Physician diagnosis; Administrative medical records or disease registries	Morbidity	N/A	N/A	N/A	N/A	N/A	158	201	Hospital	Home	Family (anyone in household)	Never smokers
Asthma	Balemans 2006	N/A	Prospective cohort	Netherlands	Both	2	21	Self-report	Self-report	Incidence	N/A	693	N/A	19	N/A	N/A	N/A	N/A	Home	Maternal	Any smoking status
Asthma	Beijsterveldt 2008	N/A	Prospective cohort	Netherlands	Both	0.25	0.999	Self-report	Self-report	Incidence	11,684	N/A	N/A	5	N/A	N/A	N/A	N/A	Home	Paternal	Never smokers
Asthma	Bener 2007	N/A	Case-control	Qatar	Both	6	13	Self-report	Self-report; Physician diagnosis	Morbidity	N/A	N/A	N/A	N/A	N/A	716	716	Community	Home	Paternal	Unspecified
Asthma	Bergmann 2000	N/A	Prospective cohort	Germany	Both	0	0	Self-report	Physician diagnosis; Self-report	Incidence	1,314	N/A	N/A	6	N/A	N/A	N/A	N/A	Home	Family (anyone in household)	Never smokers
Asthma	Boker 2019	N/A	Case-control	Saudi Arabia	Both	2	14	Self-report	Physician diagnosis	Morbidity	N/A	N/A	N/A	N/A	N/A	257	252	Hospital	Home	Parental	Never smokers
Asthma	Boneberger 2011	N/A	Case-control	Chile	Both	6	15	Self-report	Physician diagnosis	Morbidity	N/A	N/A	N/A	N/A	N/A	188	294	Hospital	Home	Family (anyone in household)	Never smokers

Table S3. Summary of study characteristics

Health outcome	Author-Year	Study name	Study design	Location	Sex	Age start	Age end	Exposure assessment	Outcome assessment	Endpoint	Sample size	Exposed	Events	Follow-up (years)	Person-years	Cases	Controls	Control pool	Exposure location	Exposure source	Smoking status
Asthma	Bozicević 2000	N/A	Case-control	Croatia	Both	18	73	Self-report	Self-report	Morbidity	N/A	N/A	N/A	N/A	N/A	175	108	Hospital	Home; Work	Family (anyone in household); Any	Any smoking status
Asthma	Butland 1997	N/A	Case-control	Greater London	Both	7.5	8.5	Self-report	Self-report	Morbidity	N/A	N/A	N/A	N/A	N/A	N/A	N/A	Community	Home	Paternal	Never smokers
Asthma	Carlsten 2012	N/A	Prospective cohort	Canada	Both	0	0	Self-report; Biomarker	Self-report	Incidence	380	33	76	7	N/A	N/A	N/A	N/A	Home	Family (anyone in household)	Never smokers
Asthma	Carr 2019	Tucson Children's Respiratory Study (TCRS)	Prospective cohort	Arizona	Both	0	0	Self-report	Self-report	Incidence	433	N/A	N/A	6	N/A	N/A	N/A	N/A	Home	Maternal	Never smokers
Asthma	Carrasco 2021	INMA (Environment and Childhood) Birth Cohort Study	Prospective cohort	Spain	Both	0	0	Self-report	Self-report	Incidence	1,885	1,265	397	4	N/A	N/A	N/A	N/A	Home	Family (anyone in household)	Never smokers
Asthma	Chan 2019	N/A	Case-crossover	Taiwan (Province of China)	Both	18	73	Self-report	Self-report	Incidence	132	N/A	N/A	N/A	N/A	21	111	Community	Home and work	Any	Never smokers
Asthma	Charoenca 2013	N/A	Case-control	Thailand	Both	0	4	Self-report	Physician diagnosis	Morbidity	N/A	N/A	N/A	N/A	N/A	462	462	Hospital	Home	Any	Never smokers
Asthma	Chen 2011	N/A	Nested case-control	Taiwan (Province of China)	Both	12	14	Self-report	Self-report; Physician diagnosis	Incidence	579	N/A	N/A	1	N/A	193	386	Community	Home	Family (anyone in household)	Non-smokers (former and never)
Asthma	Clark 1994	N/A	Case-control	South East England	Both	5	7	Self-report	Self-report	Morbidity	N/A	N/A	N/A	N/A	N/A	23	39	Hospital	Home	Family (anyone in household)	Never smokers
Asthma	Coogan 2015	Black Women's Health Study (BWHS)	Prospective cohort	United States of America	Female	21	69	Self-report	Self-report	Incidence	N/A	N/A	819	14.7	368,174	N/A	N/A	N/A	Home or work	Any	Never smokers
Asthma	Daigler 1991	N/A	Case-control	New York	Both	0	15	Self-report	Physician diagnosis	Morbidity	N/A	N/A	N/A	N/A	N/A	137	246	Hospital	Home	Maternal	Never smokers

Table S3. Summary of study characteristics

Health outcome	Author-Year	Study name	Study design	Location	Sex	Age start	Age end	Exposure assessment	Outcome assessment	Endpoint	Sample size	Exposed	Events	Follow-up (years)	Person-years	Cases	Controls	Control pool	Exposure location	Exposure source	Smoking status
Asthma	David 2005	Singapore Chinese Health Study	Prospective cohort	Singapore	Both	45	74	Self-report	Self-report; Physician diagnosis	Incidence	35,000	N/A	N/A	5.8	N/A	N/A	N/A	N/A	Home	Any	Never smokers
Asthma	Dekker 2015	Generation R Study	Prospective cohort	Netherlands	Both	0	0	Self-report	Self-report; Biomarker	Incidence	3,457	77	N/A	6	N/A	N/A	N/A	N/A	Home	Family (anyone in household)	Never smokers
Asthma	Ehrlich 1992	N/A	Case-control	New York	Both	3	14	Self-report; Biomarker	Physician diagnosis	Morbidity	N/A	N/A	N/A	N/A	N/A	107	121	Hospital	Home	Maternal	Unspecified
Asthma	Ehrlich 1996	N/A	Case-control	Western Cape	Both	7	8	Self-report	Self-report	Morbidity	N/A	N/A	N/A	N/A	N/A	325	250	N/A	Home	Maternal	Never smokers
Asthma	El-Sharif 2003	N/A	Case-control	Palestine	Both	6	12	Self-report	Self-report	Morbidity	N/A	N/A	N/A	N/A	N/A	N/A	N/A	Community	Home	Family (anyone in household)	Never smokers
Asthma	Elder 1996	N/A	Prospective cohort	Australia	Both	0	0	Self-report	Self-report; Physician diagnosis	Incidence	525	N/A	N/A	1	N/A	N/A	N/A	N/A	Home	Maternal	Never smokers
Asthma	Fagbule 1994	N/A	Case-control	Nigeria	Both	0	15	Self-report	Self-report	Morbidity	N/A	N/A	N/A	N/A	N/A	140	140	Community	Home	Parental	Never smokers
Asthma	Fernando 2009	N/A	Case-control	Sri Lanka	Both	5	12	Self-report	Physician diagnosis	Morbidity	N/A	N/A	N/A	N/A	N/A	100	96	Hospital	Home	Family (anyone in household)	Never smokers
Asthma	Flexeder 2019	European Community Respiratory Health Survey (ECRHS)	Prospective cohort	Belgium; Germany; Spain; France; Italy; United Kingdom; Iceland; Norway; Sweden; Switzerland; Australia; Estonia	Both	20	44	Self-report	Physician diagnosis	Incidence	3,011	142	N/A	20	60,220	N/A	N/A	N/A	Other	Any	Non-smokers (former and never)
Asthma	Flodin 1995	N/A	Case-control	Sweden	Both	20	65	Self-report	Physician diagnosis; Self-report	Morbidity	N/A	N/A	N/A	N/A	N/A	79	304	Community	Work	Any	Any smoking status
Asthma	Frassanito 2022	N/A	Case-control	Lazio	Both	6	6	Self-report	Self-report; Physician diagnosis	Morbidity	N/A	N/A	N/A	N/A	N/A	66	296	Community	Other	Any	Unspecified

Table S3. Summary of study characteristics

Health outcome	Author-Year	Study name	Study design	Location	Sex	Age start	Age end	Exposure assessment	Outcome assessment	Endpoint	Sample size	Exposed	Events	Follow-up (years)	Person-years	Cases	Controls	Control pool	Exposure location	Exposure source	Smoking status
Asthma	Galobardes 2015	Avon Longitudinal Study of Parents and Children (ALSPAC)	Prospective cohort	South West England	Both	0	0	Self-report	Self-report; Biomarker	Incidence	5,500	754	N/A	8	N/A	N/A	N/A	N/A	Home	Family (anyone in household)	Never smokers
Asthma	Genuneit 2006	International Study of Asthma and Allergies in Childhood (ISAAC II)	Prospective cohort	Germany	Both	9	11	Self-report	Self-report	Incidence	2,936	N/A	N/A	8	N/A	N/A	N/A	N/A	Home	Family (anyone in household)	Non-smokers (former and never)
Asthma	Goksör 2006	N/A	Prospective cohort	Sweden	Both	0	1	Self-report	Self-report	Incidence	383	N/A	N/A	20	N/A	N/A	N/A	N/A	Other	Any	Never smokers
Asthma	Grabenhenrich 2014	German MAS-90	Prospective cohort	Germany	Both	0	0	Self-report	Self-report; Physician diagnosis	Incidence	1,010	212	N/A	20	20,200	N/A	N/A	N/A	Home	Family (anyone in household)	Never smokers
Asthma	Guo 2019	N/A	Case-control	Shandong	Both	0	9	Self-report	Administrative medical records or disease registries	Morbidity	N/A	N/A	N/A	N/A	N/A	102	80	Community	Home	Family (anyone in household)	Never smokers
Asthma	Hadnadjev 2011	N/A	Case-control	Serbia	Both	7	14	Self-report	Administrative medical records or disease registries	Morbidity	N/A	N/A	N/A	N/A	N/A	252	252	Community	Home	Any	Unspecified
Asthma	Hagendorens 2005	Prospective study on the Influence of Perinatal factors on the Ocurrence of asthma and allergies (PIPO)	Prospective cohort	Belgium	Both	0	0	Self-report	Self-report	Incidence	810	N/A	N/A	1	N/A	N/A	N/A	N/A	Other	Any	Never smokers
Asthma	Hedman 2011	N/A	Prospective cohort	Sweden	Both	7	8	Self-report	Self-report	Incidence	2,805	N/A	N/A	9	N/A	N/A	N/A	N/A	Home	Maternal	Non-smokers (former and never)

Table S3. Summary of study characteristics

Health outcome	Author-Year	Study name	Study design	Location	Sex	Age start	Age end	Exposure assessment	Outcome assessment	Endpoint	Sample size	Exposed	Events	Follow-up (years)	Person-years	Cases	Controls	Control pool	Exposure location	Exposure source	Smoking status
Asthma	Horwood 1985	Christchurch Child Development Study	Prospective cohort	New Zealand	Both	0	0	Self-report	Self-report; Physician diagnosis	Incidence	1,056	N/A	N/A	6	N/A	N/A	N/A	N/A	Home	Parental	Never smokers
Asthma	Huang 2022	Childhood Environment and Allergic Diseases Study (CEAS)	Case-control	Taiwan (Province of China)	Both	3	18	Self-report	Physician diagnosis	Morbidity	N/A	N/A	N/A	N/A	N/A	41	106	Hospital	Home	Family (anyone in household)	Never smokers
Asthma	Hunt 2011	Syracuse AUDIT (Assessment of Urban Dwellings for Indoor Toxics) birth cohort study	Prospective cohort	New York	Both	0	0	Self-report; Biomarker	Physician diagnosis	Incidence	103	70	39	1	N/A	N/A	N/A	N/A	Home	Family (anyone in household)	Never smokers
Asthma	Hwang 2011	N/A	Case-control	Taiwan (Province of China)	Both	1	7	Self-report	Self-report	Morbidity	N/A	N/A	N/A	N/A	N/A	188	376	N/A	Home	Family (anyone in household)	Never smokers
Asthma	Håberg 2007	MoBa Study	Prospective cohort	Norway	Both	0	0	Self-report	Self-report	Incidence	22,390	N/A	N/A	1.5	N/A	N/A	N/A	N/A	Home	Maternal; Paternal	Never smokers
Asthma	Izuhara 2016	The Nagahama Study	Prospective cohort	Shiga	Both	30	75	Self-report	Self-report	Incidence	N/A	N/A	N/A	2	N/A	N/A	N/A	N/A	Other	Any	Unspecified
Asthma	Jaakkola 2001	N/A	Prospective cohort	Oslo	Both	0	0	Self-report	Self-report; Physician diagnosis	Incidence	2,531	N/A	N/A	4	N/A	N/A	N/A	N/A	Home	Family (anyone in household)	Never smokers
Asthma	Jaakkola 2003	N/A	Case-control	Finland	Both	21	63	Self-report	Physician diagnosis; Administrative medical records or disease registries	Morbidity	N/A	N/A	N/A	N/A	N/A	683	1,424	Community	Home and work; Work; Home	Any	Never smokers
Asthma	Jedrychowski 2008	N/A	Prospective cohort	Małopolskie	Both	0	0	Self-report	Self-report	Incidence	468	N/A	N/A	2	N/A	N/A	N/A	N/A	Home	Family (anyone in household)	Never smokers
Asthma	Kamran 2015	N/A	Case-control	Pakistan	Both	1	15	Self-report	Physician diagnosis	Morbidity	N/A	N/A	N/A	N/A	N/A	173	173	Community	Home	Parental	Unspecified

Table S3. Summary of study characteristics

Health outcome	Author-Year	Study name	Study design	Location	Sex	Age start	Age end	Exposure assessment	Outcome assessment	Endpoint	Sample size	Exposed	Events	Follow-up (years)	Person-years	Cases	Controls	Control pool	Exposure location	Exposure source	Smoking status
Asthma	Kanoh 2012	N/A	Prospective cohort	Japan	Both	0.5	0.5	Self-report	Self-report	Incidence	62,165	15,909	N/A	4.5	N/A	N/A	N/A	N/A	Home	Maternal; Paternal	Never smokers
Asthma	Karunasekera 2001	N/A	Case-control	Sri Lanka	Both	1	10	Self-report	Physician diagnosis	Morbidity	N/A	N/A	N/A	N/A	N/A	300	300	Hospital	Other	Any	Unspecified
Asthma	Khozime 2019	N/A	Case-control	Khorasan-e-Razavi	Both	5	70	Self-report	Physician diagnosis	Morbidity	N/A	N/A	N/A	N/A	N/A	50	50	Hospital	Home	Family (anyone in household)	Non-smokers (former and never)
Asthma	Kim 2018	Elementary School Student Cohort	Case-cohort	Republic of Korea	Both	5.68	12.16	Self-report	Self-report; Physician diagnosis	Incidence	3,770	881	N/A	4	13,480	N/A	N/A	N/A	Other	Any	Never smokers
Asthma	Klinnert 2001	N/A	Prospective cohort	United States of America	Both	0	0	Self-report	Administrative medical records or disease registries; Self-report	Incidence	145	N/A	N/A	8	N/A	N/A	N/A	N/A	Home	Maternal	Never smokers
Asthma	Kobyletzki 2012	Dampness in Building and Health study	Prospective cohort	Sweden	Both	1	2	Self-report	Self-report	Incidence	3,124	694	73	5	N/A	N/A	N/A	N/A	Home	Parental	Never smokers
Asthma	Kumar 2021	N/A	Case-control	Maharashtra	Both	6	12	Self-report	Physician diagnosis	Morbidity	N/A	N/A	N/A	N/A	N/A	180	180	Community	Home	Maternal	Unspecified
Asthma	Kurukulaaratchy 2006	N/A	Prospective cohort	South East England	Both	0	0	Self-report	Self-report	Incidence	1,373	N/A	367	10	N/A	N/A	N/A	N/A	Home	Parental	Never smokers
Asthma	Lawson 2014	National Population Health Survey	Prospective cohort	Canada	Both	12	18	Self-report	Self-report	Incidence	2,038,890	825,750	N/A	12	21,274,890	N/A	N/A	N/A	Home	Family (anyone in household)	Never smokers
Asthma	Leen 1994	N/A	Case-control	Ireland	Both	0	15	Self-report	Administrative medical records or disease registries	Morbidity	N/A	N/A	N/A	N/A	N/A	115	96	Community	Home	Family (anyone in household)	Unspecified
Asthma	Lemanske 2005	Childhood Origins of ASThma (COAST) study	Prospective cohort	United States of America	Both	0	0	Self-report	Self-report; Physician diagnosis	Incidence	275	N/A	N/A	3	N/A	N/A	N/A	N/A	Other	Any	Never smokers

Table S3. Summary of study characteristics

Health outcome	Author-Year	Study name	Study design	Location	Sex	Age start	Age end	Exposure assessment	Outcome assessment	Endpoint	Sample size	Exposed	Events	Follow-up (years)	Person-years	Cases	Controls	Control pool	Exposure location	Exposure source	Smoking status
Asthma	Li 2013	open cohort	Prospective cohort	Sweden	Both	2	11	Self-report	Administrative medical records or disease registries	Incidence	864,468	231,645	17,672	11	9,509,148	N/A	N/A	N/A	Home	Maternal	Never smokers
Asthma	Lilljeqvist 1997	Psychosocial Risks for Allergy Development (PRAD)-Project	Case-control	Oslo	Both	7	10	Self-report	Self-report	Morbidity	N/A	N/A	N/A	N/A	N/A	293	413	Community	Home	Parental	Never smokers
Asthma	Litonjua 2001	N/A	Prospective cohort	Massachusetts	Both	0	0.00548	Self-report	Self-report; Physician diagnosis	Incidence	222	N/A	N/A	2	N/A	N/A	N/A	N/A	Home	Family (anyone in household)	Never smokers
Asthma	Majeed 2008	N/A	Case-control	Sindh	Both	1	8	Self-report	Physician diagnosis; Administrative medical records or disease registries	Morbidity	N/A	N/A	N/A	N/A	N/A	200	199	Hospital	Home	Family (anyone in household)	Unspecified
Asthma	Marbury 1996	Indoor Air and Children's Health Study	Prospective cohort	Minnesota	Both	0	0.167	Self-report	Administrative medical records or disease registries; Self-report	Incidence	1,424	N/A	N/A	2	2,537	N/A	N/A	N/A	Home	Maternal	Never smokers
Asthma	McConnell 2002	Southern California Children's Health Study	Prospective cohort	California	Both	9	16	Self-report	Self-report; Physician diagnosis	Incidence	3,535	N/A	N/A	5	N/A	N/A	N/A	N/A	Home	Maternal	Any smoking status
Asthma	McKeever 2001	N/A	Prospective cohort	West Midlands	Both	0	0	Disease; Registry	Administrative medical records or disease registries	Incidence	29,238	N/A	N/A	2.9	N/A	N/A	N/A	N/A	Home	Maternal; Paternal	Unspecified
Asthma	Melsom 2001	N/A	Nested case-control	Nepal	Both	11	17	Self-report	Self-report	Incidence	247	N/A	N/A	0.167	N/A	121	126	Community	Home	Family (anyone in household)	Unspecified
Asthma	Midodzi 2010	N/A	Prospective cohort	Canada	Both	0	1	Self-report	Self-report	Incidence	8,499	3,429	N/A	4	N/A	N/A	N/A	N/A	Home	Parental	Never smokers

Table S3. Summary of study characteristics

Health outcome	Author-Year	Study name	Study design	Location	Sex	Age start	Age end	Exposure assessment	Outcome assessment	Endpoint	Sample size	Exposed	Events	Follow-up (years)	Person-years	Cases	Controls	Control pool	Exposure location	Exposure source	Smoking status
Asthma	Milanzi 2017	PIAMA	Prospective cohort	Netherlands	Both	0	0	Self-report	Self-report	Incidence	1,454	N/A	N/A	17	24,718	N/A	N/A	N/A	Other	Any	Never smokers
Asthma	Milner 2004	N/A	Prospective cohort	United States of America	Both	0	0	Self-report	Self-report; Physician diagnosis	Incidence	8,071	N/A	N/A	3	N/A	N/A	N/A	N/A	Home	Family (anyone in household)	Never smokers
Asthma	Mommers 2005	N/A	Nested case-control	Germany; Netherlands	Both	7	8	Self-report	Self-report	Incidence	1,191	N/A	N/A	1	N/A	590	601	Community	Other	Any	Unspecified
Asthma	Morfín-Maciel 2006	N/A	Case-control	Mexico City	Both	13	14	Self-report	Self-report	Morbidity	N/A	N/A	N/A	N/A	N/A	227	5,570	Community	Home	Maternal; Family (anyone in household)	Any smoking status; Unspecified
Asthma	Mpairwe 2019	N/A	Case-control	Uganda	Both	5	17	Self-report	Physician diagnosis	Morbidity	N/A	N/A	N/A	N/A	N/A	555	1,115	Community	Home	Family (anyone in household)	Never smokers
Asthma	Mumcuoglu 1994	N/A	Case-control	Palestine	Both	3	15	Self-report	Self-report; Administrative medical records or disease registries	Morbidity	N/A	N/A	N/A	N/A	N/A	300	100	Community	Home	Paternal; Other family (not spouse)	Unspecified
Asthma	Murray 2004	National Asthma Campaign Manchester Asthma and Allergy Study	Prospective cohort	United Kingdom	Both	0	3	Self-report	Physician diagnosis; Self-report	Incidence	369	70	198	3	N/A	N/A	N/A	N/A	Home	Maternal	Never smokers
Asthma	Muñoz 2012	N/A	Case-control	Coahuila	Both	6	9	Self-report; Biomarker	Self-report	Morbidity	N/A	N/A	N/A	N/A	N/A	90	111	Community	Home	Family (anyone in household)	Never smokers
Asthma	Neuman 2012	ALSPAC	Prospective cohort	South West England	Both	0	0	Self-report	Self-report	Incidence	5,867	407	N/A	6	N/A	N/A	N/A	N/A	Home	Maternal	Never smokers
Asthma	Neuman 2012	AMICS-Menorca	Prospective cohort	Spain	Both	0	0	Self-report	Self-report	Incidence	280	12	N/A	6	N/A	N/A	N/A	N/A	Home	Maternal	Never smokers
Asthma	Neuman 2012	DARC	Prospective cohort	Denmark	Both	0.083	0.167	Self-report	Self-report	Incidence	332	17	N/A	6	N/A	N/A	N/A	N/A	Home	Maternal	Never smokers
Asthma	Neuman 2012	GINIplus	Prospective cohort	Germany	Both	0	0	Self-report	Self-report	Incidence	3,296	137	N/A	6	N/A	N/A	N/A	N/A	Home	Maternal	Never smokers

Table S3. Summary of study characteristics

Health outcome	Author-Year	Study name	Study design	Location	Sex	Age start	Age end	Exposure assessment	Outcome assessment	Endpoint	Sample size	Exposed	Events	Follow-up (years)	Person-years	Cases	Controls	Control pool	Exposure location	Exposure source	Smoking status
Asthma	Neuman 2012	LISAplus	Prospective cohort	Germany	Both	0.00822	0.011	Self-report	Self-report	Incidence	1,488	67	N/A	6	N/A	N/A	N/A	N/A	Home	Maternal	Never smokers
Asthma	Neuman 2012	MAS	Prospective cohort	Germany	Both	0.083	0.167	Self-report	Self-report	Incidence	688	127	N/A	6	N/A	N/A	N/A	N/A	Home	Maternal	Never smokers
Asthma	Neuman 2012	PIAMA-NHS	Prospective cohort	Netherlands	Both	0	0	Self-report	Self-report	Incidence	2,347	56	N/A	6	N/A	N/A	N/A	N/A	Home	Maternal	Never smokers
Asthma	Nguyen 2010	N/A	Nested case-control	New York	Both	0	99	Self-report	Self-report	Incidence	4,727	N/A	N/A	1	N/A	1,555	3,172	Hospital	Home	Family (anyone in household)	Unspecified
Asthma	Norbäck 2018	China-Children-Homes-Health (CCHH) Study	Retrospective cohort	China	Both	0	0	Self-report	Self-report	Incidence	37,686	22,176	N/A	6	N/A	N/A	N/A	N/A	Home	Family (anyone in household)	Never smokers
Asthma	O'Connell 1974	N/A	Case-control	United States of America	Both	2	16	Self-report	Administrative medical records or disease registries	Morbidity	N/A	N/A	N/A	N/A	N/A	400	228	Hospital	Home	Parental	Unspecified
Asthma	Oddy 1999	Western Australian Pregnancy Cohort Study	Prospective cohort	Australia	Both	0	0	Self-report	Self-report	Incidence	2,187	N/A	N/A	6	N/A	N/A	N/A	N/A	Home	Family (anyone in household)	Never smokers
Asthma	Palvo 2008	N/A	Case-control	São Paulo	Both	6	7	Self-report	Self-report	Morbidity	N/A	N/A	N/A	N/A	N/A	N/A	N/A	Community	Home	Family (anyone in household)	Never smokers
Asthma	Patrick 2020	Canadian Healthy Infant Longitudinal Development (CHILD) prospective birth cohort study	Prospective cohort	Canada	Both	0	0	Self-report	Physician diagnosis; Self-report	Incidence	N/A	500	118	5	N/A	N/A	N/A	N/A	Home	Family (anyone in household)	Never smokers
Asthma	Pattemore 2018	The New Zealand Asthma and Allergy Cohort Study	Prospective cohort	New Zealand	Both	0	0	Self-report	Self-report	Incidence	2,167	N/A	N/A	4.8	9,208	N/A	N/A	N/A	Home	Maternal	Never smokers

Table S3. Summary of study characteristics

Health outcome	Author-Year	Study name	Study design	Location	Sex	Age start	Age end	Exposure assessment	Outcome assessment	Endpoint	Sample size	Exposed	Events	Follow-up (years)	Person-years	Cases	Controls	Control pool	Exposure location	Exposure source	Smoking status
Asthma	Pokharel 2001	N/A	Case-control	Haryana, Rural	Both	11	15	Self-report	Self-report	Morbidity	N/A	N/A	N/A	N/A	N/A	40	80	Community	Home	Family (anyone in household)	Unspecified
Asthma	Pokharel 2007	N/A	Case-control	Nepal	Both	11	15	Self-report	Self-report	Morbidity	N/A	N/A	N/A	N/A	N/A	120	240	Community	Home	Family (anyone in household)	Unspecified
Asthma	Polk 2004	Asthma Multicenter Infants Cohort Study	Prospective cohort	South East England; Spain	Both	0	4	Self-report	Self-report	Incidence	1,289	N/A	N/A	4	N/A	N/A	N/A	N/A	Home	Maternal	Never smokers
Asthma	Polosa 2005	N/A	Retrospective cohort	Sicilia	Both	18	40	Self-report	Physician diagnosis	Incidence	436	178	N/A	10	N/A	N/A	N/A	N/A	Home	Parental	Unspecified
Asthma	Ponsonby 2000	N/A	Prospective cohort	Australia	Both	0	0	Self-report	Self-report	Incidence	6,378	N/A	N/A	7	N/A	N/A	N/A	N/A	Home	Family (anyone in household)	Never smokers
Asthma	Ratageri 2000	N/A	Case-control	Delhi	Both	5	15	Self-report	Physician diagnosis	Morbidity	N/A	N/A	N/A	N/A	N/A	60	60	Hospital	Home	Family (anyone in household)	Any smoking status
Asthma	Rennie 2008	N/A	Case-control	Canada	Both	6	13	Self-report	Self-report; Physician diagnosis	Morbidity	N/A	N/A	N/A	N/A	N/A	89	108	Community	Home	Any	Unspecified
Asthma	Rosa 2011	N/A	Prospective cohort	New York	Both	0	0	Self-report; Biomarker	Self-report; Physician diagnosis	Incidence	290	N/A	N/A	6	N/A	N/A	N/A	N/A	Home	Family (anyone in household)	Never smokers
Asthma	Schroer 2009	Childhood Allergy and Air Pollution Study (CCAAPS)	Prospective cohort	Ohio	Both	0	0	Self-report	Self-report	Incidence	570	N/A	N/A	2	N/A	N/A	N/A	N/A	Home	Family (anyone in household)	Never smokers
Asthma	Sears 2002	Dunedin Multidisciplinary Health and Development Research Study	Prospective cohort	New Zealand	Both	0	0	Self-report	Self-report	Incidence	1,037	N/A	N/A	26	N/A	N/A	N/A	N/A	Home	Paternal	Unspecified
Asthma	Selby 2018	EuroPrevall	Prospective cohort	Iceland; South East England; Netherlands;	Both	0	0	Self-report	Self-report	Incidence	15,745	2,915	1,170	2.5	N/A	N/A	N/A	N/A	Home	Maternal; Other family (not spouse)	Never smokers

Table S3. Summary of study characteristics

Health outcome	Author-Year	Study name	Study design	Location	Sex	Age start	Age end	Exposure assessment	Outcome assessment	Endpoint	Sample size	Exposed	Events	Follow-up (years)	Person-years	Cases	Controls	Control pool	Exposure location	Exposure source	Smoking status
				Germany; Łódzkie; Lithuania; Spain; Lombardia; Greece																	
Asthma	Sherman 1990	N/A	Prospective cohort	Massachusetts	Both	5	9	Self-report	Self-report; Physician diagnosis	Incidence	770	N/A	N/A	11	N/A	N/A	N/A	N/A	Home	Maternal; Paternal	Unspecified
Asthma	Slob 2020	Netherlands Twin Register (NTR) study	Retrospective cohort	Netherlands	Both	3	3	Self-report	Self-report	Incidence	69,465	18,009	14,739	9	N/A	N/A	N/A	N/A	Home	Maternal; Paternal	Never smokers
Asthma	Snodgrass 2016	Growing Up in Singapore Towards healthy Outcomes (GUSTO)	Prospective cohort	Singapore	Both	0	0	Self-report	Self-report	Incidence	1,236	N/A	N/A	2	N/A	N/A	N/A	N/A	Home	Family (anyone in household)	Never smokers
Asthma	Strachan 1996	British National Child Development Study	Prospective cohort	United Kingdom	Both	0	0	Self-report	Self-report	Incidence	4,384	N/A	N/A	33	N/A	N/A	N/A	N/A	Home	Paternal; Maternal	Unspecified
Asthma	Sun 2015	Study of Prevention of Asthma in Children in Europe (SPACE)	Prospective cohort	Austria; England; Germany	Both	0	0	Self-report	Self-report	Incidence	549	119	26	3	N/A	N/A	N/A	N/A	Home	Parental	Never smokers
Asthma	Surdu 2006	N/A	Case-control	New York	Both	2	14	Self-report	Administrative medical records or disease registries	Morbidity	N/A	N/A	N/A	N/A	N/A	25	25	Hospital	Home	Family (anyone in household)	Unspecified
Asthma	Tadaki 2009	N/A	Prospective cohort	Gunma	Both	0	0	Self-report	Self-report; Physician diagnosis	Incidence	213	N/A	188	1	N/A	N/A	N/A	N/A	Home	Any	Never smokers
Asthma	Takemura 2001	The Tokorozawa Childhood	Case-control	Saitama	Both	6	15	Self-report	Self-report; Physician diagnosis	Morbidity	N/A	N/A	N/A	N/A	N/A	2,315	21,513	Community	Home	Parental	Unspecified

Table S3. Summary of study characteristics

Health outcome	Author-Year	Study name	Study design	Location	Sex	Age start	Age end	Exposure assessment	Outcome assessment	Endpoint	Sample size	Exposed	Events	Follow-up (years)	Person-years	Cases	Controls	Control pool	Exposure location	Exposure source	Smoking status
		Asthma and Pollinosis Study																			
Asthma	Tanaka 2008	Osaka Maternal and Child Health Study	Prospective cohort	Ōsaka	Both	0	0	Self-report	Self-report	Incidence	763	195	33	2	N/A	N/A	N/A	N/A	Home	Family (anyone in household)	Never smokers
Asthma	Tanaka 2017	Kyushu Okinawa Maternal and Child Health Study (KOMCHS)	Prospective cohort	Japan	Both	0	0	Self-report	Self-report	Incidence	1,354	601	373	2.4	N/A	N/A	N/A	N/A	Home	Family (anyone in household)	Never smokers
Asthma	Taveras 2006	Project Viva cohort study	Prospective cohort	Massachusetts	Both	0	0	Self-report	Self-report	Incidence	1,372	N/A	N/A	2	N/A	N/A	N/A	N/A	Home	Maternal	Never smokers
Asthma	Thacher 2014	Children, Allergy, Milieu, Stockholm, Epidemiolgic (BAMSE) study	Prospective cohort	Sweden	Both	0.167	0.167	Self-report	Self-report	Incidence	3,978	N/A	N/A	16	N/A	N/A	N/A	N/A	Home	Parental	Never smokers
Asthma	Thacher 2018	Mechanisms of the Development of Allergy (MeDALL) project [comprised of Children, Allergy, Milieu, Stockholm, Epidemiology (BAMSE); German Infant Nutritional Intervention (GINIplus); Influences of Lifestyle-Related Factors on	Prospective cohort	Germany	Both	0	0	Self-report	Self-report	Incidence	18,151	3,232	N/A	16	N/A	N/A	N/A	N/A	Home	Family (anyone in household)	Never smokers

Table S3. Summary of study characteristics

Health outcome	Author-Year	Study name	Study design	Location	Sex	Age start	Age end	Exposure assessment	Outcome assessment	Endpoint	Sample size	Exposed	Events	Follow-up (years)	Person-years	Cases	Controls	Control pool	Exposure location	Exposure source	Smoking status
		the Immune System and the Development of Allergies in Childhood (LISAplus)]																			
Asthma	Thorn 2001	N/A	Nested case-control	Sweden	Both	20	50	Self-report	Self-report; Physician diagnosis	Incidence	1,044	N/A	N/A	1	N/A	174	870	Community	Home	Family (anyone in household)	Never smokers
Asthma	Toizumi 2019	Nha Trang Birth Cohort Study	Prospective cohort	Viet Nam	Both	0	0	Self-report	Self-report	Incidence	1,202	685	N/A	6	N/A	N/A	N/A	N/A	Home	Family (anyone in household)	Never smokers
Asthma	Usemann 2018	Basel-Bern Infant Lung Development (BILD) birth-chort study	Prospective cohort	Switzerland	Both	0	0	Self-report	Self-report	Incidence	322	N/A	31	3	N/A	N/A	N/A	N/A	Home	Parental	Never smokers
Asthma	Valk 2012	Generation R cohort	Prospective cohort	Netherlands	Both	0	0	Self-report	Self-report	Incidence	1,342	117	N/A	4	N/A	N/A	N/A	N/A	Home	Family (anyone in household)	Never smokers
Asthma	Valk 2012	PIAMA cohort	Prospective cohort	Netherlands	Both	0	0	Self-report	Self-report	Incidence	1,570	445	N/A	4	N/A	N/A	N/A	N/A	Home	Family (anyone in household)	Never smokers
Asthma	Vázquez 2006	N/A	Case-control	Tamaulipas	Both	13	18	Self-report	Self-report	Morbidity	N/A	N/A	N/A	N/A	N/A	253	3,750	Community	Home	Family (anyone in household)	Any smoking status
Asthma	Wada 2021	N/A	Prospective cohort	Japan	Both	0	0	Self-report	Self-report	Incidence	42,136	1,905	901	1	N/A	N/A	N/A	N/A	Home	Family (anyone in household)	Never smokers
Asthma	Wang 2021	RHINE II/RHINE III	Prospective cohort	Denmark; Estonia; Norway; Sweden; Iceland	Both	27	55	Self-report	Self-report; Physician diagnosis	Incidence	11,506	1,023	N/A	11.3	N/A	N/A	N/A	N/A	Home	Family (anyone in household)	Never smokers
Asthma	Werff 2013	N/A	Prospective cohort	Cuba	Both	4	14	Self-report	Self-report	Incidence	770	364	31	3.5	N/A	N/A	N/A	N/A	Home	Family (anyone in household)	Never smokers

Table S3. Summary of study characteristics

Health outcome	Author-Year	Study name	Study design	Location	Sex	Age start	Age end	Exposure assessment	Outcome assessment	Endpoint	Sample size	Exposed	Events	Follow-up (years)	Person-years	Cases	Controls	Control pool	Exposure location	Exposure source	Smoking status
Asthma	Willers 1991	N/A	Case-control	Sweden	Both	3	15	Self-report	Administrative medical records or disease registries	Morbidity	N/A	N/A	N/A	N/A	N/A	35	59	Community	Home	Parental	Unspecified
Asthma	Yang 1998	N/A	Case-control	Taiwan (Province of China)	Both	3	15	Self-report	Physician diagnosis	Morbidity	N/A	N/A	N/A	N/A	N/A	86	86	Hospital	Home	Family (anyone in household)	Never smokers
Asthma	Yang 1998	N/A	Case-control	Taiwan (Province of China)	Both	6	12	Self-report	Self-report	Morbidity	N/A	N/A	N/A	N/A	N/A	165	165	Community	Home	Parental	Never smokers
Asthma	Youssef 2018	N/A	Case-control	Egypt	Both	3	8	Self-report	Self-report; Physician diagnosis	Morbidity	N/A	N/A	N/A	N/A	N/A	45	52	N/A	Home	Family (anyone in household)	Never smokers
Asthma	Zejda 2003	N/A	Prospective cohort	Śląskie	Both	7	9	Self-report	Self-report; Physician diagnosis	Incidence	663	N/A	N/A	7	N/A	N/A	N/A	N/A	Home	Parental	Unspecified
Asthma	Zheng 2002	N/A	Case-control	Beijing	Both	6	10	Self-report	Self-report; Physician diagnosis	Morbidity	N/A	N/A	N/A	N/A	N/A	403	806	Community	Home	Family (anyone in household)	Unspecified
Breast cancer	Ahern 2009	N/A	Case-control	Massachusetts	Female	28	75	Self-report	Administrative medical records or disease registries	Morbidity	N/A	N/A	N/A	N/A	N/A	215	173	Community	Home or work	Any	Never smokers
Breast cancer	Alberg 2004	CLUE II	Nested case-control	Maryland	Female	18	99	Self-report	Administrative medical records or disease registries; Death certificates	Incidence & Mortality	128	N/A	N/A	N/A	N/A	62	66	Community	Home	Spouse	Never smokers
Breast cancer	Anderson 2012	Ontario Women's Diet and Health Study	Case-control	Canada	Female	25	74	Self-report	Administrative medical records or disease registries	Morbidity	N/A	N/A	N/A	N/A	N/A	1,827	1,913	Community	Home; Work	Family (anyone in household); Any	Never smokers
Breast cancer	Bonner 2005	Western New York Exposures and Breast	Case-control	New York	Female	35	79	Self-report	Administrative medical records or disease	Morbidity	N/A	N/A	N/A	N/A	N/A	242	492	Community	Home or work	Any	Never smokers

Table S3. Summary of study characteristics

Health outcome	Author-Year	Study name	Study design	Location	Sex	Age start	Age end	Exposure assessment	Outcome assessment	Endpoint	Sample size	Exposed	Events	Follow-up (years)	Person-years	Cases	Controls	Control pool	Exposure location	Exposure source	Smoking status
		Cancer Study (WEB Study)							registries; Physician diagnosis												
Breast cancer	Chaveepojnkamjorn 2017	N/A	Case-control	Thailand	Female	25	44	Self-report	Physician diagnosis; Biomarker	Morbidity	N/A	N/A	N/A	N/A	N/A	257	257	Hospital	Other	Any	Any smoking status
Breast cancer	De 2010	N/A	Case-control	Sri Lanka	Female	30	64	Self-report	Biomarker; Physician diagnosis; Administrative medical records or disease registries	Morbidity	N/A	N/A	N/A	N/A	N/A	100	203	Hospital	Other	Any	Never smokers
Breast cancer	Dianatinasab 2017	N/A	Case-control	Khuzestan; Kohgiluyeh and Boyer-Ahmad; Bushehr; Fars; Hormozgan	Female	18	99	Self-report	Biomarker	Morbidity	N/A	N/A	N/A	N/A	N/A	526	526	Hospital	Home	Parental	Any smoking status
Breast cancer	Dossus 2014	European Prospective Investigation into Cancer and Nutrition (EPIC)	Prospective cohort	Sicilia; Toscana; Piemonte; Lombardia; France; Germany; Netherlands; Campania; Denmark; Sweden; Troms og Finnmark	Female	18	99	Self-report	Administrative medical records or disease registries; Self-report	Incidence & Mortality	183,608	N/A	3,597	11	N/A	N/A	N/A	N/A	Home or work	Any	Never smokers
Breast cancer	El-Sheikh 2021	N/A	Case-control	Egypt	Female	19	99	Self-report	Physician diagnosis; Biomarker	Morbidity	N/A	N/A	N/A	N/A	N/A	48	42	Hospital	Other	Any	Non-smokers (former and never)
Breast cancer	Fararouei 2019	N/A	Case-control	Fars	Female	20	50	Self-report	Physician diagnosis; Biomarker	Morbidity	N/A	N/A	N/A	N/A	N/A	505	505	Hospital	Other	Any	Any smoking status
Breast cancer	Fu 2015	N/A	Case-control	Guangdong	Female	18	99	Self-report	Administrative medical records or	Morbidity	N/A	N/A	N/A	N/A	N/A	367	419	Hospital	Other	Any	Any smoking status

Table S3. Summary of study characteristics

Health outcome	Author-Year	Study name	Study design	Location	Sex	Age start	Age end	Exposure assessment	Outcome assessment	Endpoint	Sample size	Exposed	Events	Follow-up (years)	Person-years	Cases	Controls	Control pool	Exposure location	Exposure source	Smoking status
									disease registries												
Breast cancer	Gao 2013	N/A	Case-control	Jiangsu	Female	18	99	Self-report	Biomarker; Physician diagnosis	Morbidity	N/A	N/A	N/A	N/A	N/A	630	657	Community	Home	Spouse	Any smoking status
Breast cancer	Hanaoka 2005	Japan Public Health Center (JPHC) based prospective study on cancer and cardiovascular diseases (JPHC Study Cohort I)	Prospective cohort	Japan	Female	40	59	Self-report	Administrative medical records or disease registries; Death certificates	Incidence & Mortality	N/A	N/A	154	9.2	177,971	N/A	N/A	N/A	Home	Family (anyone in household)	Never smokers
Breast cancer	Hirose 1995	N/A	Case-control	Aichi	Female	20	99	Self-report	Administrative medical records or disease registries	Morbidity	N/A	N/A	N/A	N/A	N/A	560	11,276	Hospital	Home	Spouse	Never smokers
Breast cancer	Hosseinzadeh 2014	N/A	Case-control	East Azarbayejan	Female	18	99	Self-report	Biomarker	Morbidity	N/A	N/A	N/A	N/A	N/A	140	280	Hospital	Other	Any	Any smoking status
Breast cancer	Hsieh 2014	N/A	Case-control	Taiwan (Province of China)	Female	18	99	Self-report	Administrative medical records or disease registries; Biomarker	Morbidity	N/A	N/A	N/A	N/A	N/A	737	719	Community	Other	Any	Any smoking status
Breast cancer	Hu 2013	N/A	Case-control	Hubei	Female	25	75	Self-report	Biomarker	Morbidity	N/A	N/A	N/A	N/A	N/A	211	196	Hospital	Home or work	Any	Any smoking status
Breast cancer	Ilic 2013	N/A	Case-control	Serbia	Female	18	99	Self-report	Biomarker; Physician diagnosis	Morbidity	N/A	N/A	N/A	N/A	N/A	130	124	Hospital	Home or work	Any	Never smokers
Breast cancer	Jee 1999	N/A	Prospective cohort	Republic of Korea	Female	40	88	Self-report	Administrative medical records or disease registries	Incidence	120,634	84,525	138	3.5	N/A	N/A	N/A	N/A	Home	Spouse	Never smokers

Table S3. Summary of study characteristics

Health outcome	Author-Year	Study name	Study design	Location	Sex	Age start	Age end	Exposure assessment	Outcome assessment	Endpoint	Sample size	Exposed	Events	Follow-up (years)	Person-years	Cases	Controls	Control pool	Exposure location	Exposure source	Smoking status
Breast cancer	Johnson 2000	National Enhanced Cancer Surveillance System	Case-control	Canada	Female	25	74	Self-report	Administrative medical records or disease registries	Morbidity	N/A	N/A	N/A	N/A	N/A	1,261	1,362	Community	Home or work	Any	Never smokers; Former smokers
Breast cancer	Kariri 2017	N/A	Case-control	Palestine	Female	18	60	Self-report	Administrative medical records or disease registries	Morbidity	N/A	N/A	N/A	N/A	N/A	105	209	Community	Other	Any	Unspecified
Breast cancer	Lash 1999	N/A	Case-control	Massachusetts	Female	18	99	Self-report	Administrative medical records or disease registries	Morbidity	N/A	N/A	N/A	N/A	N/A	120	406	Community	Home	Family (anyone in household)	Never smokers
Breast cancer	Lash 2002	N/A	Case-control	Massachusetts	Female	18	99	Self-report	Administrative medical records or disease registries	Morbidity & Mortality	N/A	N/A	N/A	N/A	N/A	305	249	Community	Home	Family (anyone in household)	Never smokers
Breast cancer	Li 2015	N/A	Case-control	Guangdong	Female	25	70	Self-report	Physician diagnosis; Biomarker	Morbidity	N/A	N/A	N/A	N/A	N/A	877	890	Hospital	Home or work	Any	Never smokers
Breast cancer	Lin 2008	Japan Collaborative Cohort Study for Evaluation of Cancer Risk (JACC)	Prospective cohort	Japan	Female	40	79	Self-report	Administrative medical records or disease registries; Death certificates	Incidence & Mortality	N/A	N/A	194	7.9	331,669	N/A	N/A	N/A	Home	Family (anyone in household)	Never smokers
Breast cancer	Liu 2000	N/A	Case-control	Chongqing	Female	24	55	Self-report	Biomarker; Physician diagnosis	Morbidity	N/A	N/A	N/A	N/A	N/A	186	186	Hospital	Home; Work	Family (anyone in household); Any	Never smokers
Breast cancer	Luo 2011	Women's Health Initiative Observational Study	Prospective cohort	United States of America	Female	50	79	Self-report	Self-report; Administrative medical records or disease registries	Incidence	45,035	33,773	958	10.3	N/A	N/A	N/A	N/A	Home and work; Home; Work	Any; Family (anyone in household)	Never smokers

Table S3. Summary of study characteristics

Health outcome	Author-Year	Study name	Study design	Location	Sex	Age start	Age end	Exposure assessment	Outcome assessment	Endpoint	Sample size	Exposed	Events	Follow-up (years)	Person-years	Cases	Controls	Control pool	Exposure location	Exposure source	Smoking status
Breast cancer	Marzouk 2009	N/A	Case-control	Egypt	Female	18	59	Self-report	Physician diagnosis; Biomarker	Morbidity	N/A	N/A	N/A	N/A	N/A	198	153	Hospital	Home	Family (anyone in household)	Any smoking status
Breast cancer	Metsola 2005	N/A	Case-control	Finland	Female	37.5	91.6	Self-report	Physician diagnosis; Biomarker	Morbidity	N/A	N/A	N/A	N/A	N/A	363	351	Community	Home or work	Any	Never smokers
Breast cancer	Millikan 1998	Carolina Breast Cancer Study (CBCS)	Case-control	North Carolina	Female	20	74	Self-report	Administrative medical records or disease registries	Morbidity	N/A	N/A	N/A	N/A	N/A	247	253	Community	Home	Family (anyone in household)	Never smokers
Breast cancer	Morabia 1996	N/A	Case-control	Switzerland	Female	30	74	Self-report	Administrative medical records or disease registries; Biomarker	Morbidity	N/A	N/A	N/A	N/A	N/A	90	455	Community	Home	Spouse	Never smokers
Breast cancer	Niehoff 2017	N/A	Case-control	New York	Female	20	98	Self-report	Administrative medical records or disease registries	Morbidity	N/A	N/A	N/A	N/A	N/A	975	956	N/A	Home	Spouse	Any smoking status
Breast cancer	Nishino 2001	N/A	Prospective cohort	Miyagi	Female	40	99	Self-report	Administrative medical records or disease registries	Incidence	N/A	N/A	45	7.6	38,112	N/A	N/A	N/A	Home	Family (anyone in household)	Never smokers
Breast cancer	Nishino 2014	N/A	Case-control	Miyagi	Female	30	99	Self-report	Administrative medical records or disease registries	Morbidity	N/A	N/A	N/A	N/A	N/A	581	1,570	Hospital	Home	Spouse	Never smokers
Breast cancer	Pimhanam 2014	N/A	Case-control	Thailand	Female	17	76	Self-report	Biomarker	Morbidity	N/A	N/A	N/A	N/A	N/A	427	429	Hospital	Other	Any	Never smokers
Breast cancer	Pirie 2008	Million Women Study	Prospective cohort	United Kingdom	Female	50	64	Self-report	Administrative medical records or disease registries; Death certificates	Incidence & Mortality	431,040	43,231	1,107	3.5	N/A	N/A	N/A	N/A	Home	Family (anyone in household); Spouse	Never smokers

Table S3. Summary of study characteristics

Health outcome	Author-Year	Study name	Study design	Location	Sex	Age start	Age end	Exposure assessment	Outcome assessment	Endpoint	Sample size	Exposed	Events	Follow-up (years)	Person-years	Cases	Controls	Control pool	Exposure location	Exposure source	Smoking status
Breast cancer	Reynolds 2009	California Teacher's Study	Prospective cohort	California	Female	20	99	Self-report	Administrative medical records or disease registries; Death certificates; Self-report	Incidence & Mortality	83,494	69,354	2,596	10	N/A	N/A	N/A	N/A	Home; Work	Family (anyone in household); Any	Never smokers
Breast cancer	Roddam 2007	N/A	Case-control	England	Female	36	45	Self-report	Administrative medical records or disease registries	Morbidity	N/A	N/A	N/A	N/A	N/A	570	577	Community	Home	Spouse	Never smokers; Former smokers
Breast cancer	Rollison 2008	N/A	Case-control	Delaware	Female	40	79	Self-report	Administrative medical records or disease registries	Morbidity	N/A	N/A	N/A	N/A	N/A	246	319	Community	Work; Home	Any; Family (anyone in household)	Never smokers
Breast cancer	Shrubsole 2004	Shanghai Breast Cancer Study (SBCS)	Case-control	Shanghai	Female	25	64	Self-report	Administrative medical records or disease registries	Morbidity	N/A	N/A	N/A	N/A	N/A	1,013	1,117	Community	Home	Spouse	Never smokers
Breast cancer	Smith 1994	The UK National Case-Control Study Group (UKNCCSG)	Case-control	England	Female	18	35	Self-report	Administrative medical records or disease registries	Morbidity	N/A	N/A	N/A	N/A	N/A	21	27	Community	Home or work	Any	Never smokers
Breast cancer	Strumylaite 2017	N/A	Case-control	Lithuania	Female	28	90	Self-report	Physician diagnosis; Biomarker	Morbidity	N/A	N/A	N/A	N/A	N/A	901	2,066	Hospital	Work; Home; Home and work	Any; Family (anyone in household)	Never smokers
Breast cancer	Tang 2013	N/A	Case-control	Guangdong	Female	18	99	Self-report	Administrative medical records or disease registries	Morbidity	N/A	N/A	N/A	N/A	N/A	765	818	Hospital	Home or work	Any	Never smokers
Breast cancer	Tong 2014	N/A	Case-control	Liaoning	Female	18	99	Self-report	Biomarker	Morbidity	N/A	N/A	N/A	N/A	N/A	312	312	Community	Home	Spouse	Never smokers
Breast cancer	Wartenberg 2000	American Cancer Society's	Prospective cohort	United States of	Female	30	99	Self-report	Self-report; Administrative medical	Mortality	98,693	37,883	439	5.6	N/A	N/A	N/A	N/A	Home	Spouse	Never smokers

Table S3. Summary of study characteristics

Health outcome	Author-Year	Study name	Study design	Location	Sex	Age start	Age end	Exposure assessment	Outcome assessment	Endpoint	Sample size	Exposed	Events	Follow-up (years)	Person-years	Cases	Controls	Control pool	Exposure location	Exposure source	Smoking status
		Cancer Prevention Study II (CPS-II)		America; Puerto Rico					records or disease registries; Death certificates												
Breast cancer	White 2017	National Institute of Environmental Health Sciences (NIEHS) Sister Study	Prospective cohort	United States of America	Female	35	74	Self-report	Administrative medical records or disease registries; Self-report	Incidence	N/A	N/A	979	6.4	182,457	N/A	N/A	N/A	Other	Any	Never smokers
Breast cancer	Woo 2000	N/A	Nested case-control	Maryland	Female	18	99	Self-report	Administrative medical records or disease registries	Incidence	2,132	N/A	N/A	17	N/A	706	1,426	Community	Home	Family (anyone in household)	Never smokers
Breast cancer	Xue 2011	Nurses' Health Study (NHS)	Prospective cohort	United States of America	Female	30	55	Self-report	Self-report; Administrative medical records or disease registries	Incidence & Mortality	N/A	N/A	3,456	24.3	1,067,588	N/A	N/A	N/A	Home or work	Any	Never smokers
Breast cancer	Yassin 2018	N/A	Case-control	Palestine	Female	18	99	Self-report	Administrative medical records or disease registries	Morbidity	N/A	N/A	N/A	N/A	N/A	105	210	Community	Other	Any	Any smoking status
Breast cancer	Zahali 2021	N/A	Case-control	Malaysia	Female	21	59	Self-report	Physician diagnosis; Biomarker	Morbidity	N/A	N/A	N/A	N/A	N/A	78	83	Hospital	Home; Work	Family (anyone in household); Any	Never smokers
Breast cancer	Zhao 1999	N/A	Case-control	Sichuan	Female	26	82	Self-report	Administrative medical records or disease registries; Physician diagnosis; Biomarker	Morbidity	N/A	N/A	N/A	N/A	N/A	265	265	Hospital	Other	Any	Unspecified

Table S3. Summary of study characteristics

Health outcome	Author-Year	Study name	Study design	Location	Sex	Age start	Age end	Exposure assessment	Outcome assessment	Endpoint	Sample size	Exposed	Events	Follow-up (years)	Person-years	Cases	Controls	Control pool	Exposure location	Exposure source	Smoking status
Chronic obstructive pulmonary disease	Behrendt 2005	Third National Health and Nutrition Examination Survey	Case-control	United States of America	Both	18	80	Self-report	Biomarker; Administrative medical records or disease registries; Physician diagnosis	Morbidity	N/A	N/A	N/A	N/A	N/A	397	6,922	Community	Home; Work	Family (anyone in household); Any	Non-smokers (former and never)
Chronic obstructive pulmonary disease	Chan-Yeung 2007	N/A	Case-control	Hong Kong Special Administrative Region of China	Both	18	99	Self-report	Physician diagnosis; Administrative medical records or disease registries; Biomarker	Morbidity	N/A	N/A	N/A	N/A	N/A	289	289	Community	Home or work	Any	Any smoking status
Chronic obstructive pulmonary disease	Chen 2013	N/A	Case-control	Fujian	Both	27	85	Self-report	Biomarker	Morbidity	N/A	N/A	N/A	N/A	N/A	216	432	Community	Other	Any	Any smoking status
Chronic obstructive pulmonary disease	David 2005	Singapore Chinese Health Study	Prospective cohort	Singapore	Both	45	74	Self-report	Self-report	Incidence	31,142	29,324	149	5.8	N/A	N/A	N/A	N/A	Home; Work	Family (anyone in household); Any	Never smokers
Chronic obstructive pulmonary disease	Dennis 1996	N/A	Case-control	Colombia	Female	35	99	Self-report	Physician diagnosis; Biomarker	Morbidity	N/A	N/A	N/A	N/A	N/A	96	88	Hospital	Home	Spouse	Any smoking status
Chronic obstructive pulmonary disease	Ding 2015	N/A	Case-control	Hainan	Both	32	98	Self-report	Biomarker	Morbidity	N/A	N/A	N/A	N/A	N/A	277	307	Community	Other	Any	Any smoking status
Chronic obstructive pulmonary disease	Diver 2018	The American Cancer Society's Cancer Prevention Study-II Nutrtition Cohort (CPS-II Nutrition)	Prospective cohort	United States of America	Both	50	74	Self-report	Administrative medical records or disease registries	Mortality	N/A	N/A	235	18.8	888,879	N/A	N/A	N/A	Home	Family (anyone in household)	Never smokers

Table S3. Summary of study characteristics

Health outcome	Author-Year	Study name	Study design	Location	Sex	Age start	Age end	Exposure assessment	Outcome assessment	Endpoint	Sample size	Exposed	Events	Follow-up (years)	Person-years	Cases	Controls	Control pool	Exposure location	Exposure source	Smoking status
Chronic obstructive pulmonary disease	Enstrom 2003	CPS I (California cancer prevention study; American Cancer Society cancer prevention study)	Prospective cohort	California	Both	30	96	Self-report	Administrative medical records or disease registries; Death certificates	Mortality	2,459	1,175	209	39	N/A	N/A	N/A	N/A	Home	Spouse	Never smokers
Chronic obstructive pulmonary disease	Gerbase 2006	Swiss Study on Air Pollution and Lung Diseases in Adults (SAPALDIA)	Prospective cohort	Switzerland	Both	18	60	Self-report	Self-report; Biomarker	Incidence	1,511	309	54	11	N/A	N/A	N/A	N/A	Other	Any	Never smokers
Chronic obstructive pulmonary disease	He 2012	N/A	Prospective cohort	Shaanxi	Both	51.5	87.8	Self-report	Death certificates; Administrative medical records or disease registries	Mortality	910	611	36	15.4	N/A	N/A	N/A	N/A	Home or work	Any	Never smokers
Chronic obstructive pulmonary disease	Huang 2019	N/A	Case-control	Taiwan (Province of China)	Both	18	99	Self-report	Administrative medical records or disease registries	Morbidity	N/A	N/A	N/A	N/A	N/A	791	3,150	Community	Other	Any	Never smokers
Chronic obstructive pulmonary disease	Johannessen 2012	Bergen COPD Cohort Study	Case-control	Vestland	Both	40	79	Self-report	Physician diagnosis; Administrative medical records or disease registries; Biomarker	Morbidity	N/A	N/A	N/A	N/A	N/A	N/A	N/A	Community	Work; Home	Any; Family (anyone in household)	Any smoking status
Chronic obstructive pulmonary disease	Lee 1986	N/A	Case-control	England	Both	35	74	Self-report	Physician diagnosis; Administrative medical records or	Morbidity	N/A	N/A	N/A	N/A	N/A	14	269	Hospital	Home	Spouse	Never smokers

Table S3. Summary of study characteristics

Health outcome	Author-Year	Study name	Study design	Location	Sex	Age start	Age end	Exposure assessment	Outcome assessment	Endpoint	Sample size	Exposed	Events	Follow-up (years)	Person-years	Cases	Controls	Control pool	Exposure location	Exposure source	Smoking status
									disease registries												
Chronic obstructive pulmonary disease	McGhee 2005	N/A	Case-control	Hong Kong Special Administrative Region of China	Both	60	99	Proxy respondent	Death certificates	Mortality	N/A	N/A	N/A	N/A	N/A	138	763	Community	Home	Family (anyone in household)	Never smokers
Chronic obstructive pulmonary disease	Pahwa 2019	Saskaatchewan Rural Health Study	Prospective cohort	Canada	Both	18	99	Self-report	Self-report	Incidence	8,109	1,228	479	4	N/A	N/A	N/A	N/A	Home	Family (anyone in household)	Any smoking status
Chronic obstructive pulmonary disease	Salama 2020	N/A	Case-control	Saudi Arabia	Both	19	99	Self-report	Physician diagnosis; Biomarker	Morbidity	N/A	N/A	N/A	N/A	N/A	157	157	Hospital	Other	Any	Any smoking status
Chronic obstructive pulmonary disease	Salameh 2012	N/A	Case-control	Lebanon	Both	40	99	Self-report	Physician diagnosis	Morbidity	N/A	N/A	N/A	N/A	N/A	248	348	Community	Work; Home	Any; Family (anyone in household)	Any smoking status
Chronic obstructive pulmonary disease	Sandler 1989	N/A	Prospective cohort	Maryland	Both	25	99	Self-report	Death certificates	Mortality	19,035	10,799	19	10	189,597	N/A	N/A	N/A	Home	Family (anyone in household)	Never smokers
Chronic obstructive pulmonary disease	Vineis 2005	European prospective investigation into cancer and nutrition (EPIC) - GenAir	Nested case-control	France; Italy; Denmark; Sweden; Netherlands; Germany	Both	35	74	Self-report	Administrative medical records or disease registries; Self-report	Mortality	300	N/A	N/A	N/A	N/A	14	286	Community	Home or work	Any	Non-smokers (former and never)
Chronic obstructive pulmonary disease	Wu 2010	N/A	Case-control	Taiwan (Province of China)	Female	40	99	Self-report	Administrative medical records or disease registries; Biomarker	Morbidity	N/A	N/A	N/A	N/A	N/A	127	133	Community	Home or work	Any	Non-smokers (former and never)
Chronic obstructive pulmonary disease	Xu 2007	N/A	Nested case-control	Jiangsu	Both	35	99	Self-report	Self-report; Administrative medical records or	Incidence	1,769	N/A	N/A	N/A	N/A	879	890	Community	Other	Any	Any smoking status

Table S3. Summary of study characteristics

Health outcome	Author-Year	Study name	Study design	Location	Sex	Age start	Age end	Exposure assessment	Outcome assessment	Endpoint	Sample size	Exposed	Events	Follow-up (years)	Person-years	Cases	Controls	Control pool	Exposure location	Exposure source	Smoking status
									disease registries												
Diabetes mellitus type 2	Hayashino 2008	High Risk and Population Strategy for Occupational Health Promotion Study (HIPOP-OHP)	Prospective cohort	Japan	Both	19	69	Self-report	Biomarker; Administrative medical records or disease registries; Self-report	Incidence	2,819	690	819	3.4	N/A	N/A	N/A	N/A	Work; Home	Any; Family (anyone in household)	Non-smokers (former and never)
Diabetes mellitus type 2	Huang 2020	China Kadoorie Biobank (CKB) cohort study	Prospective cohort	Jiangsu	Female	30	79	Self-report	Administrative medical records or disease registries; Biomarker; Self-report	Incidence	28,177	19,391	774	7.2	202,746	N/A	N/A	N/A	Other	Any	Never smokers
Diabetes mellitus type 2	Jiang 2019	California Teacher Study (CTS)	Prospective cohort	California	Female	20	79	Self-report	Self-report	Incidence	37,447	14,065	2,315	17	N/A	N/A	N/A	N/A	Home	Family (anyone in household)	Never smokers
Diabetes mellitus type 2	Kim 2021	Kangbuk Samsung Health Study	Retrospective cohort	Republic of Korea	Both	18	99	Self-report; Biomarker	Biomarker; Self-report	Incidence	131,724	30,223	2,115	14	N/A	N/A	N/A	N/A	Home or work	Any	Never smokers
Diabetes mellitus type 2	Kowall 2010	KORA (Cooperative Health Research in the Region of Augsburg) S4 survey	Prospective cohort	Germany	Both	55	74	Self-report	Self-report; Biomarker	Incidence	861	161	35	7	N/A	N/A	N/A	N/A	Home or work	Any	Never smokers; Former smokers
Diabetes mellitus type 2	Oba 2020	Japan Public Health Center-based Prospective Study (JPHC Study)	Prospective cohort	Japan	Female	40	69	Self-report	Self-report	Incidence	19,001	12,432	N/A	10	N/A	N/A	N/A	N/A	Home	Spouse	Never smokers
Diabetes mellitus type 2	Rias 2020	N/A	Case-control	East Java	Both	18	75	Self-report	Biomarker; Physician diagnosis	Morbidity	N/A	N/A	N/A	N/A	N/A	261	277	Community	Other	Any	Never smokers

Table S3. Summary of study characteristics

Health outcome	Author-Year	Study name	Study design	Location	Sex	Age start	Age end	Exposure assessment	Outcome assessment	Endpoint	Sample size	Exposed	Events	Follow-up (years)	Person-years	Cases	Controls	Control pool	Exposure location	Exposure source	Smoking status
Diabetes mellitus type 2	Zeng 2022	N/A	Case-control	Jiangxi	Female	25	95	Self-report	Self-report; Biomarker	Morbidity	N/A	N/A	N/A	N/A	N/A	79	81	Community	Other	Any	Non-smokers (former and never)
Diabetes mellitus type 2	Zhang 2011	Nurses' Health Study (NHS)	Prospective cohort	United States of America	Female	41	55	Self-report	Self-report	Incidence	N/A	N/A	2,161	15.3	663,594	N/A	N/A	N/A	Other	Any	Never smokers
Ischemic heart disease	Attard 2017	Maltese Acute Myocardial Infarction Study (MAMI)	Case-control	Malta	Both	20	77	Self-report	Administrative medical records or disease registries	Morbidity	N/A	N/A	N/A	N/A	N/A	12	10	Community	Home	Family (anyone in household)	Non-smokers (former and never)
Ischemic heart disease	Awawdi 2016	N/A	Case-control	Israel	Female	35	70	Self-report	Physician diagnosis	Morbidity	N/A	N/A	N/A	N/A	N/A	19	11	Community	Home	Spouse	Never smokers
Ischemic heart disease	Ciruzzi 1998	Factores de Riesgo Coronario en America del Sur (FRICAS)	Case-control	Argentina	Both	24	83	Self-report	Administrative medical records or disease registries; Biomarker	Morbidity	N/A	N/A	N/A	N/A	N/A	131	117	Hospital	Home	Family (anyone in household)	Never smokers
Ischemic heart disease	Clark 2013	Singapore Chinese Health Study	Prospective cohort	Singapore	Both	45	74	Self-report	Administrative medical records or disease registries	Mortality	N/A	N/A	311	5.5	161,425	N/A	N/A	N/A	Home or work	Any	Never smokers
Ischemic heart disease	Ding 2009	N/A	Case-control	Hong Kong Special Administrative Region of China	Female	18	99	Self-report	Administrative medical records or disease registries; Biomarker	Morbidity	N/A	N/A	N/A	N/A	N/A	95	65	Hospital	Home	Family (anyone in household)	Never smokers
Ischemic heart disease	Diver 2018	The American Cancer Society's Cancer Prevention Study-II Nutrtition Cohort (CPS-II Nutrition)	Prospective cohort	United States of America	Both	50	74	Self-report	Administrative medical records or disease registries	Mortality	N/A	N/A	2,939	18.8	888,879	N/A	N/A	N/A	Home	Family (anyone in household)	Never smokers

Table S3. Summary of study characteristics

Health outcome	Author-Year	Study name	Study design	Location	Sex	Age start	Age end	Exposure assessment	Outcome assessment	Endpoint	Sample size	Exposed	Events	Follow-up (years)	Person-years	Cases	Controls	Control pool	Exposure location	Exposure source	Smoking status
Ischemic heart disease	Dobson 1991	N/A	Case-control	Australia	Both	35	69	Self-report	Administrative medical records or disease registries	Morbidity & Mortality	N/A	N/A	N/A	N/A	N/A	960	1,944	Community	Home; Work	Family (anyone in household); Any	Never smokers; Former smokers
Ischemic heart disease	Enstrom 2003	CPS-I (California cancer prevention study)	Prospective cohort	California	Both	30	96	Self-report	Administrative medical records or disease registries; Death certificates	Mortality	28,079	13,222	4,747	38	N/A	N/A	N/A	N/A	Home	Spouse	Never smokers
Ischemic heart disease	Fatmi 2014	N/A	Case-control	Pakistan	Female	0	99	Self-report	Physician diagnosis	Morbidity	N/A	N/A	N/A	N/A	N/A	73	73	Hospital	Home	Any	Any smoking status
Ischemic heart disease	Gallo 2010	European Prospective Investigation into Cancer and Nutrition (EPIC)	Prospective cohort	France; Italy; Netherlands; Sweden; Denmark; Norway	Both	18	99	Self-report; Biomarker	Death certificates	Mortality	69,870	22,758	81	9.9	N/A	N/A	N/A	N/A	Home	Family (anyone in household)	Never smokers
Ischemic heart disease	Garland 1985	N/A	Prospective cohort	California	Female	50	79	Self-report	Death certificates; Administrative medical records or disease registries; Self-report	Mortality	300	97	4	10	N/A	N/A	N/A	N/A	Home	Spouse	Never smokers
Ischemic heart disease	He 1994	N/A	Case-control	Shaanxi	Female	37	67	Self-report	Physician diagnosis; Biomarker; Administrative medical records or disease registries	Morbidity	N/A	N/A	N/A	N/A	N/A	48	76	Hospital	Home or work	Any	Never smokers
Ischemic heart disease	He 2012	N/A	Prospective cohort	Shaanxi	Both	51.5	87.8	Self-report	Death certificates; Administrative medical records or disease	Mortality	910	611	41	15.4	N/A	N/A	N/A	N/A	Home or work	Any	Never smokers

Table S3. Summary of study characteristics

Health outcome	Author-Year	Study name	Study design	Location	Sex	Age start	Age end	Exposure assessment	Outcome assessment	Endpoint	Sample size	Exposed	Events	Follow-up (years)	Person-years	Cases	Controls	Control pool	Exposure location	Exposure source	Smoking status
									registries; Biomarker												
Ischemic heart disease	Hill 2007	New Zealand Census-Mortality Study	Prospective cohort	New Zealand	Both	45	74	Self-report	Death certificates	Mortality	668,259	121,812	4,251	3	1,983,184	N/A	N/A	N/A	Home	Family (anyone in household)	Never smokers
Ischemic heart disease	Hole 1989	N/A	Prospective cohort	Scotland	Both	45	64	Self-report	Administrative medical records or disease registries; Death certificates	Mortality	2,455	1,538	84	11.5	N/A	N/A	N/A	N/A	Home	Family (anyone in household)	Never smokers
Ischemic heart disease	Janghorbani 1997	N/A	Case-control	Kerman	Female	42	85	Self-report	Physician diagnosis	Morbidity	N/A	N/A	N/A	N/A	N/A	200	400	Hospital	Home	Family (anyone in household)	Never smokers
Ischemic heart disease	Kastorini 2013	N/A	Case-control	Greece	Both	18	99	Self-report	Administrative medical records or disease registries; Physician diagnosis	Morbidity	N/A	N/A	N/A	N/A	N/A	32	37	Community	Other	Any	Non-smokers (former and never)
Ischemic heart disease	Kawachi 1997	Nurses' Health Study cohort	Prospective cohort	United States of America	Female	30	55	Self-report	Administrative medical records or disease registries; Death certificates	Incidence & Mortality	N/A	N/A	152	9.4	302,325	N/A	N/A	N/A	Home or work	Any	Never smokers
Ischemic heart disease	Kobayashi 2022	N/A	Prospective cohort	Japan	Female	40	59	Self-report	Administrative medical records or disease registries	Incidence	22,445	11,239	62	18.1	431,721	N/A	N/A	N/A	Other	Spouse	Never smokers
Ischemic heart disease	La 1993	GISSI-2 (Gruppo Italiano per lo Studio della Sopravvivenz	Nested case-control	Italy	Both	29	74	Self-report	Physician diagnosis; Administrative medical records or	Incidence	61	N/A	N/A	N/A	N/A	24	37	Hospital	Home	Spouse	Never smokers

Table S3. Summary of study characteristics

Health outcome	Author-Year	Study name	Study design	Location	Sex	Age start	Age end	Exposure assessment	Outcome assessment	Endpoint	Sample size	Exposed	Events	Follow-up (years)	Person-years	Cases	Controls	Control pool	Exposure location	Exposure source	Smoking status
		a nell'Infarto Miocardico)							disease registries												
Ischemic heart disease	Lee 1986	N/A	Case-control	England	Both	35	74	Self-report	Physician diagnosis; Administrative medical records or disease registries	Morbidity	N/A	N/A	N/A	N/A	N/A	70	269	Hospital	Home	Spouse	Never smokers
Ischemic heart disease	Malinauskiene 2011	N/A	Case-control	Lithuania	Female	35	61	Self-report	Administrative medical records or disease registries	Morbidity	N/A	N/A	N/A	N/A	N/A	58	100	Community	Other	Any	Never smokers
Ischemic heart disease	McElduff 1998	WHO MONICA study	Case-control	Australia; New Zealand	Both	35	69	Self-report	Administrative medical records or disease registries; Self-report; Death certificates	Morbidity & Mortality	N/A	N/A	N/A	N/A	N/A	186	844	Community	Home or work	Any	Non-smokers (former and never)
Ischemic heart disease	McGhee 2005	N/A	Case-control	Hong Kong Special Administrative Region of China	Both	60	99	Proxy respondent	Death certificates	Mortality	N/A	N/A	N/A	N/A	N/A	584	763	Community	Home	Family (anyone in household)	Never smokers
Ischemic heart disease	Muscat 1995	N/A	Case-control	New York; Pennsylvania; Michigan; Illinois	Both	18	74	Self-report	Administrative medical records or disease registries; Physician diagnosis; Biomarker	Morbidity	N/A	N/A	N/A	N/A	N/A	64	88	Hospital	Work	Any	Never smokers
Ischemic heart disease	Nishtar 2004	N/A	Case-control	Pakistan	Both	39	67	Self-report	Physician diagnosis	Morbidity	N/A	N/A	N/A	N/A	N/A	200	200	Hospital	Home	Family (anyone in household)	Any smoking status
Ischemic heart disease	Notara 2015	GREECS (GREEk acute	Prospective cohort	Greece	Both	18	99	Self-report	Physician diagnosis	Incidence & Mortality	N/A	N/A	N/A	10	N/A	N/A	N/A	N/A	Work; Home	Any; Family (anyone in household)	Unspecified

Table S3. Summary of study characteristics

Health outcome	Author-Year	Study name	Study design	Location	Sex	Age start	Age end	Exposure assessment	Outcome assessment	Endpoint	Sample size	Exposed	Events	Follow-up (years)	Person-years	Cases	Controls	Control pool	Exposure location	Exposure source	Smoking status
		Coronary Syndrome)																			
Ischemic heart disease	Pitsavos 2002	Cardio2000	Case-control	Greece	Both	18	99	Self-report	Biomarker; Administrative medical records or disease registries	Morbidity	N/A	N/A	N/A	N/A	N/A	N/A	N/A	Community	Home or work	Any	Never smokers
Ischemic heart disease	Rashid 2019	N/A	Case-control	Malaysia	Female	30	65	Self-report	Physician diagnosis; Administrative medical records or disease registries	Morbidity	N/A	N/A	N/A	N/A	N/A	91	53	Community	Home or work	Any	Unspecified
Ischemic heart disease	Rosenlund 2001	Stockholm Heart Epidemiology Program (SHEEP)	Case-control	Sweden	Both	45	70	Self-report	Administrative medical records or disease registries	Morbidity	N/A	N/A	N/A	N/A	N/A	113	196	Community	Home; Work	Spouse; Any	Never smokers
Ischemic heart disease	Rossi 2011	N/A	Case-control	Costa Rica	Both	18	99	Self-report	Physician diagnosis; Biomarker	Morbidity	N/A	N/A	N/A	N/A	N/A	N/A	N/A	Community	Home	Family (anyone in household)	Non-smokers (former and never)
Ischemic heart disease	Sadeghi 2020	Tehran Lipid and Glucose Study (TLGS)	Prospective cohort	Tehran	Both	30	99	Self-report	Self-report; Administrative medical records or disease registries	Incidence & Mortality	6,003	1,791	720	14.7	N/A	N/A	N/A	N/A	Home or work	Any	Never smokers
Ischemic heart disease	Sandler 1989	N/A	Prospective cohort	Maryland	Both	25	99	Self-report	Death certificates	Mortality	19,035	10,799	1,358	10	189,597	N/A	N/A	N/A	Home	Family (anyone in household)	Never smokers
Ischemic heart disease	Spencer 1999	N/A	Case-control	Australia	Male	25	69	Self-report	Administrative medical records or disease registries	Morbidity	N/A	N/A	N/A	N/A	N/A	310	816	Community	Home; Work	Family (anyone in household); Any	Any smoking status
Ischemic heart disease	Steenland 1996	American Cancer Society's Cancer	Prospective cohort	United States of America	Both	30	99	Self-report	Administrative medical records or disease	Mortality	135,217	N/A	1,606	7	N/A	N/A	N/A	N/A	Home	Spouse	Never smokers

Table S3. Summary of study characteristics

Health outcome	Author-Year	Study name	Study design	Location	Sex	Age start	Age end	Exposure assessment	Outcome assessment	Endpoint	Sample size	Exposed	Events	Follow-up (years)	Person-years	Cases	Controls	Control pool	Exposure location	Exposure source	Smoking status
		Prevention Study II (CPS-II)							registries; Death certificates												
Ischemic heart disease	Sulo 2008	N/A	Case-control	Albania	Both	35	74	Self-report	Biomarker; Administrative medical records or disease registries	Morbidity	N/A	N/A	N/A	N/A	N/A	141	333	Community	Home	Spouse	Never smokers
Ischemic heart disease	Svendsen 1987	Multiple Risk Factor Intervention Trial (MRFIT)	Prospective cohort	United States of America	Male	35	57	Self-report	Administrative medical records or disease registries; Physician diagnosis; Death certificates	Incidence & Mortality	1,245	286	69	7	N/A	N/A	N/A	N/A	Home	Spouse	Never smokers
Ischemic stroke	Kastorini 2013	N/A	Case-control	Greece	Both	18	99	Self-report	Administrative medical records or disease registries; Physician diagnosis	Morbidity	N/A	N/A	N/A	N/A	N/A	38	51	Community	Other	Any	Non-smokers (former and never)
Ischemic stroke	You 1999	N/A	Case-control	Australia	Both	18	99	Self-report	Physician diagnosis; Biomarker	Morbidity	N/A	N/A	N/A	N/A	N/A	73	70	Community	Home	Spouse	Never smokers
Lower respiratory infections	Almirall 2014	Community-acquired pneumonia in Health Care Centres (PACAP) study	Case-control	Spain	Both	14	100	Self-report	Physician diagnosis; Biomarker	Morbidity	N/A	N/A	N/A	N/A	N/A	471	532	Community	Home	Family (anyone in household)	Never smokers
Lower respiratory infections	Arlington 2019	Global Network for Women's and Children's Health Research MNH	Prospective cohort	Maharashtra	Both	0	0	Self-report	Self-report	Incidence	1,586	302	302	0.5	N/A	N/A	N/A	N/A	Home	Family (anyone in household)	Never smokers

Table S3. Summary of study characteristics

Health outcome	Author-Year	Study name	Study design	Location	Sex	Age start	Age end	Exposure assessment	Outcome assessment	Endpoint	Sample size	Exposed	Events	Follow-up (years)	Person-years	Cases	Controls	Control pool	Exposure location	Exposure source	Smoking status
		Registry Study																			
Lower respiratory infections	Baker 2006	immune biomarker study (IBS)	Prospective cohort	Czechia	Both	0	0	Self-report	Administrative medical records or disease registries	Incidence	N/A	N/A	890	1.7	1,295	N/A	N/A	N/A	Home	Other family (not spouse)	Never smokers
Lower respiratory infections	Barsam 2013	N/A	Case-control	Minas Gerais	Both	0.5	13	Self-report	Physician diagnosis; Biomarker	Morbidity	N/A	N/A	N/A	N/A	N/A	126	126	Hospital	Home	Family (anyone in household)	Never smokers
Lower respiratory infections	Behrooz 2018	N/A	Case-control	Massachusetts	Both	0	0.999	Self-report	Administrative medical records or disease registries	Morbidity	N/A	N/A	N/A	N/A	N/A	550	391	Hospital	Home	Family (anyone in household)	Never smokers
Lower respiratory infections	Bermúdez 2021	N/A	Prospective cohort	Spain	Both	0	0	Self-report	Self-report; Administrative medical records or disease registries	Incidence	231	76	89	2	N/A	N/A	N/A	N/A	Home	Parental	Never smokers
Lower respiratory infections	Broor 2001	N/A	Case-control	India	Both	0	4	Self-report	Administrative medical records or disease registries	Morbidity	N/A	N/A	N/A	N/A	N/A	386	311	Hospital	Home	Paternal; Other family (not spouse)	Never smokers
Lower respiratory infections	Broughton 2005	N/A	Prospective cohort	United Kingdom	Both	0	0	Self-report	Self-report; Biomarker	Incidence	126	N/A	N/A	1	N/A	94	32	Hospital	Home	Parental	Never smokers
Lower respiratory infections	Charoenca 2013	N/A	Case-control	Thailand	Both	0.08	4	Self-report	Physician diagnosis	Morbidity	N/A	N/A	N/A	N/A	N/A	462	462	Hospital	Home	Parental	Never smokers
Lower respiratory infections	Colley 1974	N/A	Prospective cohort	Greater London	Both	0	0	Self-report	Self-report; Administrative medical records or disease registries	Incidence	1,774	1,030	205	1	N/A	N/A	N/A	N/A	Home	Parental	Never smokers

Table S3. Summary of study characteristics

Health outcome	Author-Year	Study name	Study design	Location	Sex	Age start	Age end	Exposure assessment	Outcome assessment	Endpoint	Sample size	Exposed	Events	Follow-up (years)	Person-years	Cases	Controls	Control pool	Exposure location	Exposure source	Smoking status
Lower respiratory infections	Dina 2021	N/A	Case-control	West Java	Both	1	5	Self-report	Physician diagnosis	Morbidity	N/A	N/A	N/A	N/A	N/A	107	107	Hospital	Home	Family (anyone in household)	Never smokers
Lower respiratory infections	Duijts 2008	Generation R Study	Prospective cohort	Netherlands	Both	0	0	Self-report	Self-report	Incidence	3,264	534	N/A	0.5	N/A	N/A	N/A	N/A	Home	Maternal	Never smokers
Lower respiratory infections	Dwedar 2018	N/A	Case-control	Egypt	Both	18	99	Self-report	Administrative medical records or disease registries	Morbidity	N/A	N/A	N/A	N/A	N/A	20	20	Hospital	Home	Family (anyone in household)	Non-smokers (former and never)
Lower respiratory infections	Farr 2000	N/A	Case-control	England	Both	18	75	Self-report	Administrative medical records or disease registries	Morbidity	N/A	N/A	N/A	N/A	N/A	178	769	Community	Home	Spouse; Other family (not spouse)	Non-smokers (former and never)
Lower respiratory infections	Farr 2000	N/A	Case-control	East Midlands	Both	15	79	Self-report	Administrative medical records or disease registries	Morbidity	N/A	N/A	N/A	N/A	N/A	65	421	Community	Home	Family (anyone in household)	Any smoking status
Lower respiratory infections	Farzana 2017	N/A	Case-control	Bangladesh	Both	0.164	1	Self-report	Physician diagnosis; Administrative medical records or disease registries; Biomarker	Morbidity	N/A	N/A	N/A	N/A	N/A	64	64	Hospital	Home	Parental	Never smokers
Lower respiratory infections	Fergusson 1985	Christchurch Child Development Study	Prospective cohort	New Zealand	Both	0	0	Proxy respondent	Physician diagnosis; Self-report	Incidence	2,294	569	242	2	N/A	N/A	N/A	N/A	Home	Maternal only; Paternal only; Parental-both	Never smokers
Lower respiratory infections	Frassanito 2022	N/A	Case-control	Lazio	Both	0	0.999	Self-report	Administrative medical records or disease registries	Morbidity	N/A	N/A	N/A	N/A	N/A	645	183	Community	Home	Family (anyone in household)	Never smokers

Table S3. Summary of study characteristics

Health outcome	Author-Year	Study name	Study design	Location	Sex	Age start	Age end	Exposure assessment	Outcome assessment	Endpoint	Sample size	Exposed	Events	Follow-up (years)	Person-years	Cases	Controls	Control pool	Exposure location	Exposure source	Smoking status
Lower respiratory infections	Fuentes-Leonarte 2015	N/A	Prospective cohort	Spain	Both	0	0	Self-report	Self-report	Incidence	1,309	N/A	749	2.5	N/A	N/A	N/A	N/A	Home	Maternal; Paternal	Never smokers
Lower respiratory infections	Goetghebuer 2004	N/A	Case-control	England	Both	0	1	Self-report	Physician diagnosis	Morbidity	N/A	N/A	N/A	N/A	N/A	140	356	Hospital	Home	Parental	Never smokers
Lower respiratory infections	Grant 2012	N/A	Case-control	New Zealand	Both	0	4	Self-report	Administrative medical records or disease registries	Morbidity	N/A	N/A	N/A	N/A	N/A	288	351	Community	Home	Family (anyone in household)	Never smokers
Lower respiratory infections	Hassan 2001	N/A	Case-control	Iraq	Both	0.167	5	Self-report	Physician diagnosis	Morbidity	N/A	N/A	N/A	N/A	N/A	148	250	Hospital	Home	Family (anyone in household)	Never smokers
Lower respiratory infections	Håberg 2007	Norwegian Mother and Child Cohort (MoBa) study	Prospective cohort	Norway	Both	0	0	Self-report	Self-report	Incidence	17,945	3,488	2,978	1.5	N/A	N/A	N/A	N/A	Home	Parental	Never smokers
Lower respiratory infections	Islam 2021	N/A	Case-control	Bangladesh	Both	0.5	5	Self-report	Physician diagnosis	Morbidity	N/A	N/A	N/A	N/A	N/A	174	174	Hospital	Home	Family (anyone in household)	Never smokers
Lower respiratory infections	Johnson 1992	N/A	Case-control	Oyo	Both	0.04	4	Self-report	Physician diagnosis; Administrative medical records or disease registries	Morbidity	N/A	N/A	N/A	N/A	N/A	100	100	Hospital	Home	Family (anyone in household)	Never smokers
Lower respiratory infections	Keskinoglu 2007	N/A	Case-control	Türkiye	Both	2	12	Self-report	Administrative medical records or disease registries	Morbidity	N/A	N/A	N/A	N/A	N/A	150	150	Community	Home	Family (anyone in household)	Never smokers
Lower respiratory infections	Koch 2003	N/A	Prospective cohort	Greenland	Both	0.115	2	Self-report	Self-report; Physician diagnosis	Incidence	287	233	N/A	2	N/A	N/A	N/A	N/A	Home	Family (anyone in household)	Never smokers
Lower respiratory infections	Lanari 2015	N/A	Prospective cohort	Italy	Both	0	0	Self-report	Self-report; Administrative medical records or	Incidence & Mortality	3,518	902	185	1	N/A	N/A	N/A	N/A	Other; Home	Family (anyone in household)	Never smokers

Table S3. Summary of study characteristics

Health outcome	Author-Year	Study name	Study design	Location	Sex	Age start	Age end	Exposure assessment	Outcome assessment	Endpoint	Sample size	Exposed	Events	Follow-up (years)	Person-years	Cases	Controls	Control pool	Exposure location	Exposure source	Smoking status
									disease registries												
Lower respiratory infections	Liu 2022	N/A	Retrospective cohort	Guangdong	Both	3	6	Self-report	Physician diagnosis	Incidence	3,226	1,862	733	1	N/A	N/A	N/A	N/A	Home	Family (anyone in household)	Never smokers
Lower respiratory infections	Liyanage 2021	N/A	Case-control	Sri Lanka	Both	0.167	5.083	Self-report	Physician diagnosis	Morbidity	N/A	N/A	N/A	N/A	N/A	37	37	Hospital	Home	Family (anyone in household)	Never smokers
Lower respiratory infections	Loeb 2009	N/A	Case-control	Canada	Both	65	99	Self-report	Physician diagnosis; Biomarker	Morbidity	N/A	N/A	N/A	N/A	N/A	693	867	Community	Home	Family (anyone in household)	Any smoking status
Lower respiratory infections	Marbury 1996	N/A	Prospective cohort	Minnesota	Both	0	0	Self-report	Administrative medical records or disease registries	Incidence	1,424	107	30	2	N/A	N/A	N/A	N/A	Home	Maternal	Never smokers
Lower respiratory infections	McConnochie 1986	N/A	Case-control	New York	Both	0	1	Self-report	Physician diagnosis; Administrative medical records or disease registries	Morbidity	N/A	N/A	N/A	N/A	N/A	53	106	Hospital	Home	Family (anyone in household)	Never smokers
Lower respiratory infections	Miyahara 2017	Nha Trang Birth Cohort study	Prospective cohort	Viet Nam	Both	0	0	Self-report	Physician diagnosis; Administrative medical records or disease registries; Biomarker	Incidence	1,624	930	180	2	N/A	N/A	N/A	N/A	Home	Paternal	Never smokers
Lower respiratory infections	Nenna 2017	N/A	Case-control	Lazio	Both	0.5	2	Self-report	Administrative medical records or disease registries	Morbidity	N/A	N/A	N/A	N/A	N/A	213	213	Hospital	Home	Family (anyone in household)	Never smokers
Lower respiratory infections	Nuesslein 1999	N/A	Prospective cohort	Germany	Both	0	0	Self-report	Self-report	Incidence	91	47	N/A	1	N/A	N/A	N/A	N/A	Home	Family (anyone in household)	Never smokers

Table S3. Summary of study characteristics

Health outcome	Author-Year	Study name	Study design	Location	Sex	Age start	Age end	Exposure assessment	Outcome assessment	Endpoint	Sample size	Exposed	Events	Follow-up (years)	Person-years	Cases	Controls	Control pool	Exposure location	Exposure source	Smoking status
Lower respiratory infections	Pullan 1982	N/A	Case-control	North East England	Both	0	0.999	Self-report	Administrative medical records or disease registries	Morbidity	N/A	N/A	N/A	N/A	N/A	130	111	Community	Home	Maternal; Paternal	Never smokers
Lower respiratory infections	Ramesh 2012	N/A	Case-control	Karnataka	Both	0.08	4	Self-report	Physician diagnosis	Morbidity	N/A	N/A	N/A	N/A	N/A	101	101	Hospital	Other	Any	Never smokers
Lower respiratory infections	Robledo-Aceves 2018	N/A	Case-control	Jalisco	Both	0.082	1	Self-report	Physician diagnosis; Administrative medical records or disease registries; Biomarker	Morbidity	N/A	N/A	N/A	N/A	N/A	134	134	Hospital	Home	Family (anyone in household)	Never smokers
Lower respiratory infections	Roda 2011	PARIS cohort study	Prospective cohort	France	Both	0	1	Self-report	Self-report	Incidence	2,940	592	N/A	1	N/A	N/A	N/A	N/A	Home	Family (anyone in household)	Never smokers
Lower respiratory infections	Roux 2015	N/A	Prospective cohort	Western Cape	Both	0	0	Self-report	Physician diagnosis; Biomarker	Incidence	N/A	N/A	141	0.7	511	N/A	N/A	N/A	Home	Maternal	Never smokers
Lower respiratory infections	Rylander 1995	N/A	Case-control	Sweden	Both	0.329	4	Self-report	Physician diagnosis	Morbidity	N/A	N/A	N/A	N/A	N/A	347	719	Community	Home	Paternal; Maternal; Maternal and paternal	Never smokers
Lower respiratory infections	Schulte-Hobein 1992	N/A	Prospective cohort	Germany	Both	0	0	Self-report; Biomarker	Self-report	Incidence	138	69	50	1	N/A	N/A	N/A	N/A	Home	Maternal	Never smokers
Lower respiratory infections	Taylor 1987	Child Health and Education Study (CHES)	Prospective cohort	United Kingdom	Both	0	0	Self-report	Self-report	Incidence	5,982	353	889	5	N/A	N/A	N/A	N/A	Home	Maternal	Never smokers
Lower respiratory infections	Tupasi 1990	N/A	Prospective cohort	National Capital Region	Both	0	4	Self-report	Physician diagnosis	Incidence	N/A	N/A	6,542	2	1,080	N/A	N/A	N/A	Home	Maternal and paternal	Never smokers
Lower respiratory infections	Törmänen 2018	N/A	Case-control	Finland	Both	0	0.5	Self-report	Physician diagnosis; Administrative medical records or	Morbidity	N/A	N/A	N/A	N/A	N/A	138	112	Community	Home	Maternal; Paternal	Never smokers

Table S3. Summary of study characteristics

Health outcome	Author-Year	Study name	Study design	Location	Sex	Age start	Age end	Exposure assessment	Outcome assessment	Endpoint	Sample size	Exposed	Events	Follow-up (years)	Person-years	Cases	Controls	Control pool	Exposure location	Exposure source	Smoking status
									disease registries; Biomarker												
Lower respiratory infections	Verani 2016	N/A	Case-control	Gauteng	Both	0.153	4	Self-report	Physician diagnosis; Biomarker	Morbidity	N/A	N/A	N/A	N/A	N/A	889	2,628	Community	Home	Parental	Never smokers
Lower respiratory infections	Victora 1994	N/A	Case-control	Rio Grande do Sul	Both	0	1	Self-report	Biomarker; Physician diagnosis	Morbidity	N/A	N/A	N/A	N/A	N/A	864	850	Community	Home	Paternal; Maternal; Maternal and paternal	Never smokers
Lower respiratory infections	Wenten 2005	Air pollution and absence study (APAS)	Prospective cohort	California	Both	9	11	Self-report	Self-report	Incidence	1,313	223	N/A	0.5	N/A	223	1,090	Community	Home	Family (anyone in household)	Never smokers
Lower respiratory infections	Wright 1991	N/A	Prospective cohort	Arizona	Both	0	0	Self-report	Physician diagnosis; Administrative medical records or disease registries	Incidence	797	207	N/A	1.7	N/A	N/A	N/A	N/A	Home	Maternal	Never smokers
Otitis media	Adair-Bischoff 1998	N/A	Case-control	Canada	Both	0	3.083	Self-report; Biomarker	Self-report; Administrative medical records or disease registries	Morbidity	N/A	N/A	N/A	N/A	N/A	N/A	N/A	Community	Home	Family (anyone in household)	Never smokers
Otitis media	Alho 1993	N/A	Prospective cohort	Finland	Both	0.08	0.08	Self-report	Administrative medical records or disease registries; Physician diagnosis	Incidence	2,512	316	1,956	1.8	N/A	N/A	N/A	N/A	Home	Parental	Never smokers
Otitis media	Bentdal 2007	Oslo Birth Cohort	Prospective cohort	Oslo	Both	0	0	Self-report	Self-report	Incidence	2,522	1,015	336	10	N/A	N/A	N/A	N/A	Home	Parental	Never smokers
Otitis media	Clamp 2020	Avon Longitudinal Study of Parents and Children (Children of	Prospective cohort	South West England	Both	1	1	Self-report	Biomarker; Self-report	Incidence	6,560	2,920	746	8	N/A	N/A	N/A	N/A	Home	Family (anyone in household)	Never smokers

Table S3. Summary of study characteristics

Health outcome	Author-Year	Study name	Study design	Location	Sex	Age start	Age end	Exposure assessment	Outcome assessment	Endpoint	Sample size	Exposed	Events	Follow-up (years)	Person-years	Cases	Controls	Control pool	Exposure location	Exposure source	Smoking status
		the 90s study)																			
Otitis media	Collet 1995	N/A	Prospective cohort	Canada	Both	0	0	Self-report	Self-report	Incidence	918	325	601	4	N/A	N/A	N/A	N/A	Home	Maternal	Never smokers
Otitis media	Costa 2004	N/A	Case-control	Mozambique	Both	0.5	5	Self-report	Physician diagnosis; Administrative medical records or disease registries; Biomarker	Morbidity	N/A	N/A	N/A	N/A	N/A	187	118	Community	Home	Family (anyone in household)	Never smokers
Otitis media	Daigler 1991	N/A	Case-control	New York	Both	0	21	Self-report	Physician diagnosis	Morbidity	N/A	N/A	N/A	N/A	N/A	125	246	Community	Home	Maternal; Paternal	Never smokers
Otitis media	Daly 1999	Early Otitis Media Study	Prospective cohort	Minnesota	Both	0	0	Self-report	Administrative medical records or disease registries	Incidence	511	22	204	0.5	N/A	N/A	N/A	N/A	Home	Parental	Never smokers
Otitis media	Daly 2007	Little Ears Study	Prospective cohort	Minnesota	Both	0	0	Self-report	Physician diagnosis	Incidence	200	160	125	0.5	N/A	N/A	N/A	N/A	Home	Family (anyone in household)	Never smokers
Otitis media	Ey 1995	Tucson Children's Respiratory Study (CRS)	Prospective cohort	Arizona	Both	0	0	Self-report	Administrative medical records or disease registries; Physician diagnosis	Incidence	949	232	609	3	N/A	N/A	N/A	N/A	Home	Maternal	Never smokers
Otitis media	Fuentes-Leonarte 2015	INMA (Childhood and the environment)	Prospective cohort	Spain	Both	0	0	Self-report	Self-report; Physician diagnosis	Incidence	4,274	1,308	646	3	N/A	N/A	N/A	N/A	Home	Maternal; Paternal	Never smokers
Otitis media	Håberg 2010	Norwegian Mother and Child Cohort Study (MoBa)	Prospective cohort	Norway	Both	0	1.5	Self-report	Self-report	Incidence	49,704	7,470	22,671	1.5	N/A	N/A	N/A	N/A	Home	Parental	Never smokers

Table S3. Summary of study characteristics

Health outcome	Author-Year	Study name	Study design	Location	Sex	Age start	Age end	Exposure assessment	Outcome assessment	Endpoint	Sample size	Exposed	Events	Follow-up (years)	Person-years	Cases	Controls	Control pool	Exposure location	Exposure source	Smoking status
Otitis media	Jensen 2013	N/A	Prospective cohort	Greenland	Both	0	0	Self-report	Administrative medical records or disease registries	Incidence	133	91	234	11	N/A	N/A	N/A	N/A	Home	Maternal	Never smokers
Otitis media	Koch 2011	N/A	Prospective cohort	Greenland	Both	0	4	Self-report	Self-report; Physician diagnosis	Incidence	465	372	55	2.6	N/A	N/A	N/A	N/A	Home	Family (anyone in household)	Never smokers
Otitis media	Niclasen 2016	Aarhus Birth Cohort (ABC)	Prospective cohort	Denmark	Both	0	0	Self-report	Self-report	Incidence	7,578	3,993	3,159	12	N/A	N/A	N/A	N/A	Home	Maternal	Never smokers
Otitis media	Prins-van 2017	Wheezing and Illnesses Study Leidsche Rijn (WHISTLER) cohort study	Prospective cohort	Netherlands	Both	0.115	0.115	Self-report	Self-report	Incidence	1,012	26	338	0.9	N/A	N/A	N/A	N/A	Other	Any	Never smokers
Otitis media	Pukander 1985	N/A	Case-control	Finland	Both	2	3	Self-report	Physician diagnosis	Morbidity	N/A	N/A	N/A	N/A	N/A	264	207	Community	Home	Parental	Never smokers
Otitis media	Samson 2020	N/A	Prospective cohort	Tamil Nadu, Rural	Both	0	0	Self-report	Physician diagnosis	Incidence	107	18	15	8	N/A	N/A	N/A	N/A	Home	Parental	Never smokers
Otitis media	Stenstrom 1993	N/A	Case-control	Canada	Both	0	4	Self-report	Self-report; Administrative medical records or disease registries; Physician diagnosis	Morbidity	N/A	N/A	N/A	N/A	N/A	45	26	Hospital	Home	Family (anyone in household)	Never smokers
Otitis media	Ståhlberg 1986	N/A	Case-control	Finland	Both	0.833	3.75	Self-report	Physician diagnosis	Morbidity	N/A	N/A	N/A	N/A	N/A	159	210	Community	Home	Parental-one; Parental-both	Never smokers
Otitis media	Tainio 1988	N/A	Prospective cohort	Finland	Both	0	0	Self-report	Physician diagnosis	Incidence	183	66	N/A	2	N/A	N/A	N/A	N/A	Home	Parental	Never smokers
Otitis media	Teele 1989	N/A	Prospective cohort	Massachusetts	Both	0	0.25	Self-report	Physician diagnosis; Administrative medical records or	Incidence	498	298	465	7	N/A	N/A	N/A	N/A	Home	Parental	Never smokers

Table S3. Summary of study characteristics

Health outcome	Author-Year	Study name	Study design	Location	Sex	Age start	Age end	Exposure assessment	Outcome assessment	Endpoint	Sample size	Exposed	Events	Follow-up (years)	Person-years	Cases	Controls	Control pool	Exposure location	Exposure source	Smoking status
									disease registries												
Otitis media	Wijayanti 2021	N/A	Case-control	Central Java	Both	6	12	Self-report	Physician diagnosis	Morbidity	N/A	N/A	N/A	N/A	N/A	125	125	Community	Home	Family (anyone in household)	Never smokers
Otitis media	Yang 1999	N/A	Case-control	Taiwan (Province of China)	Both	6	12	Self-report	Self-report	Morbidity	N/A	N/A	N/A	N/A	N/A	219	219	Community	Home	Family (anyone in household)	Never smokers
Stroke	Bonita 1999	N/A	Case-control	New Zealand	Both	35	74	Self-report	Administrative medical records or disease registries	Morbidity & Mortality	N/A	N/A	N/A	N/A	N/A	155	676	Community	Home or work	Any	Non-smokers (former and never)
Stroke	Diver 2018	The American Cancer Society's Cancer Prevention Study-II Nutrtition Cohort (CPS-II Nutrition)	Prospective cohort	United States of America	Both	50	74	Self-report	Administrative medical records or disease registries	Mortality	N/A	N/A	1,336	18.8	888,879	N/A	N/A	N/A	Home	Family (anyone in household)	Never smokers
Stroke	Gallo 2010	European Prospective Investigation into Cancer and Nutrition (EPIC)	Prospective cohort	France; Italy; Netherlands; Germany; Sweden; Denmark; Norway	Both	18	99	Self-report	Administrative medical records or disease registries	Mortality	127,404	71,837	203	9.9	N/A	N/A	N/A	N/A	Home; Work	Family (anyone in household); Any	Never smokers
Stroke	Glymour 2008	Heatlh and Retirement Study (HRS)	Prospective cohort	United States of America	Both	50	99	Self-report	Self-report	Incidence & Mortality	16,225	N/A	688	9.1	85,551	N/A	N/A	N/A	Home	Spouse	Never smokers; Former smokers
Stroke	He 2012	N/A	Prospective cohort	Shaanxi	Both	51.5	87.8	Self-report	Death certificates; Administrative medical records or disease	Mortality	910	611	60	15.4	N/A	N/A	N/A	N/A	Home or work	Any	Never smokers

Table S3. Summary of study characteristics

Health outcome	Author-Year	Study name	Study design	Location	Sex	Age start	Age end	Exposure assessment	Outcome assessment	Endpoint	Sample size	Exposed	Events	Follow-up (years)	Person-years	Cases	Controls	Control pool	Exposure location	Exposure source	Smoking status
									registries; Biomarker												
Stroke	Hill 2007	New Zealand Census Mortality Study	Prospective cohort	New Zealand	Both	45	74	Self-report	Death certificates	Mortality	668,259	121,812	1,152	3	1,983,184	N/A	N/A	N/A	Home	Family (anyone in household)	Never smokers
Stroke	Hou 2017	China Nationwide Retrospective Mortality Survey	Case-control	China	Both	30	98	Self-report	Administrative medical records or disease registries	Mortality	N/A	N/A	N/A	N/A	N/A	N/A	N/A	Community	Home	Spouse	Never smokers
Stroke	Kobayashi 2022	Japan Public Health Center–based prospective study	Prospective cohort	Japan	Female	40	69	Self-report	Administrative medical records or disease registries	Incidence	22,445	11,239	N/A	18.9	431,721	N/A	N/A	N/A	Home	Spouse	Never smokers
Stroke	Lee 1986	N/A	Case-control	England	Both	35	74	Self-report	Physician diagnosis; Administrative medical records or disease registries	Morbidity	N/A	N/A	N/A	N/A	N/A	55	269	Hospital	Home	Spouse	Never smokers
Stroke	Malek 2015	Reasons for Geographic and Racial Differences in Stroke (REGARDS) Study	Prospective cohort	United States of America	Both	45	99	Self-report	Administrative medical records or disease registries; Self-report	Incidence & Mortality	21,743	5,081	428	5.6	121,231	N/A	N/A	N/A	Other	Any	Non-smokers (former and never)
Stroke	McGhee 2005	N/A	Case-control	Hong Kong Special Administrative Region of China	Both	60	99	Self-report	Death certificates	Mortality	N/A	N/A	N/A	N/A	N/A	223	221	Community	Home	Family (anyone in household)	Never smokers
Stroke	Nishino 2014	Three-Prefecture Cohort Study	Prospective cohort	Aichi; Miyagi; Ōsaka	Female	40	99	Self-report	Administrative medical records or disease registries;	Mortality	36,021	22,187	906	12.2	437,715	N/A	N/A	N/A	Home	Family (anyone in household)	Never smokers

Table S3. Summary of study characteristics

Health outcome	Author-Year	Study name	Study design	Location	Sex	Age start	Age end	Exposure assessment	Outcome assessment	Endpoint	Sample size	Exposed	Events	Follow-up (years)	Person-years	Cases	Controls	Control pool	Exposure location	Exposure source	Smoking status
									Death certificates												
Stroke	Poulsen 2021	Danish Diet Cancer and Health (DCH) cohort	Case-cohort	Denmark	Both	50	64	Self-report	Administrative medical records or disease registries	Incidence & Mortality	1,734	N/A	534	13.5	N/A	N/A	N/A	N/A	Home or work	Any	Never smokers
Stroke	Qureshi 2005	First National Health and Nutrition Examination Survey Epidemiologic Follow-Up Study (NHEFS)	Prospective cohort	United States of America	Female	25	74	Self-report	Administrative medical records or disease registries; Self-report	Incidence & Mortality	3,032	1,823	109	8.5	N/A	N/A	N/A	N/A	Home	Spouse	Never smokers
Stroke	Sandler 1989	N/A	Prospective cohort	Maryland	Both	25	99	Self-report	Death certificates	Mortality	N/A	N/A	655	10	189,597	N/A	N/A	N/A	Home	Family (anyone in household)	Never smokers
Stroke	Wen 2006	Shanghai Women's health study	Prospective cohort	Shanghai	Female	40	70	Self-report	Administrative medical records or disease registries; Death certificates	Mortality	65,180	39,795	157	5.7	N/A	N/A	N/A	N/A	Home	Spouse	Never smokers
Subarachnoid hemorrhage	Anderson 2004	Australasian Cooperative Research on Subarachnoid Hemorrhage Study (ACROSS)	Case-control	Australia; New Zealand	Both	15	99	Self-report	Administrative medical records or disease registries	Morbidity & Mortality	N/A	N/A	N/A	N/A	N/A	8	18	Community	Home	Family (anyone in household)	Never smokers
Subarachnoid hemorrhage	Yamada 2003	Japan Collaborative Cohort Study (JACC)	Prospective cohort	Japan	Both	40	79	Self-report	Death certificates	Mortality	N/A	N/A	72	9.9	386,840	N/A	N/A	N/A	Other	Any	Unspecified
Tracheal, bronchus,	Abdel-Rahman 2020	Prostate, Lung, Colorectal,	Prospective cohort	United States of America	Both	55	74	Self-report	Administrative medical records or	Incidence	146,981	50,061	N/A	8	N/A	N/A	N/A	N/A	Work; Home	Any; Family (anyone in household)	Never smokers

Table S3. Summary of study characteristics

Health outcome	Author-Year	Study name	Study design	Location	Sex	Age start	Age end	Exposure assessment	Outcome assessment	Endpoint	Sample size	Exposed	Events	Follow-up (years)	Person-years	Cases	Controls	Control pool	Exposure location	Exposure source	Smoking status
and lung cancer		and Ovary (PLCO) trial							disease registries												
Tracheal, bronchus, and lung cancer	Al-Zoughool 2013	N/A	Case-control	Canada	Both	35	75	Self-report	Administrative medical records or disease registries; Biomarker	Morbidity	N/A	N/A	N/A	N/A	N/A	32	410	Community	Home	Family (anyone in household)	Never smokers
Tracheal, bronchus, and lung cancer	Asomaning 2008	N/A	Case-control	Massachusetts	Both	18	99	Self-report	Physician diagnosis	Morbidity	N/A	N/A	N/A	N/A	N/A	34	128	Community	Home; Work	Family (anyone in household); Any	Never smokers
Tracheal, bronchus, and lung cancer	Behera 2005	N/A	Case-control	Other Union Territories	Female	30	80	Self-report	Physician diagnosis; Administrative medical records or disease registries; Biomarker	Morbidity	N/A	N/A	N/A	N/A	N/A	67	46	Hospital	Other	Any	Any smoking status
Tracheal, bronchus, and lung cancer	Boffetta 1998	N/A	Case-control	Germany; Sweden; France; Portugal; Spain; South West England; Veneto; Piemonte; Lazio	Both	18	74	Self-report	Administrative medical records or disease registries	Morbidity	N/A	N/A	N/A	N/A	N/A	649	1,540	Hospital	Home and work	Any	Never smokers
Tracheal, bronchus, and lung cancer	Boffetta 1999	N/A	Case-control	Sweden; France; Germany; Veneto; Lazio; Wielkopolskie; Moscow City; Romania	Both	18	99	Self-report	Administrative medical records or disease registries; Biomarker	Morbidity	N/A	N/A	N/A	N/A	N/A	70	178	Hospital	Home or work	Any	Never smokers
Tracheal, bronchus,	Brownson 1992	N/A	Case-control	Missouri	Female	30	84	Proxy respondent; Self-report	Administrative medical records or disease	Morbidity & Mortality	N/A	N/A	N/A	N/A	N/A	615	1,400	Community	Home	Family (anyone in household)	Non-smokers (former and never)

Table S3. Summary of study characteristics

Health outcome	Author-Year	Study name	Study design	Location	Sex	Age start	Age end	Exposure assessment	Outcome assessment	Endpoint	Sample size	Exposed	Events	Follow-up (years)	Person-years	Cases	Controls	Control pool	Exposure location	Exposure source	Smoking status
and lung cancer									registries; Biomarker												
Tracheal, bronchus, and lung cancer	Bräuner 2012	Diet, Cancer, and Health Study	Prospective cohort	Denmark	Both	50	64	Self-report	Administrative medical records or disease registries	Incidence & Mortality	52,692	33,865	589	9.6	N/A	N/A	N/A	N/A	Home or work	Any	Any smoking status
Tracheal, bronchus, and lung cancer	Cardenas 1997	Cancer Prevention Study II (CPS-II)	Prospective cohort	United States of America; Puerto Rico	Both	30	99	Self-report	Self-report; Death certificates; Administrative medical records or disease registries	Mortality	217,572	62,631	185	7	N/A	N/A	N/A	N/A	Home	Spouse	Never smokers
Tracheal, bronchus, and lung cancer	Cassidy 2006	The Liverpool Lung Project	Case-control	North West England	Both	18	99	Self-report	Administrative medical records or disease registries	Morbidity	N/A	N/A	N/A	N/A	N/A	779	2,224	Community	Home; Work	Family (anyone in household); Any	Any smoking status
Tracheal, bronchus, and lung cancer	Chan-Yeung 2003	N/A	Case-control	Hong Kong Special Administrative Region of China	Both	18	99	Self-report	Physician diagnosis; Biomarker	Morbidity	N/A	N/A	N/A	N/A	N/A	171	215	Hospital	Home or work	Any	Never smokers
Tracheal, bronchus, and lung cancer	Chen 2008	N/A	Case-control	Taiwan (Province of China)	Both	18	99	Self-report	Physician diagnosis; Biomarker	Morbidity	N/A	N/A	N/A	N/A	N/A	147	400	Hospital	Home or work	Any	Any smoking status
Tracheal, bronchus, and lung cancer	Cheng 2022	China Kadoorie Biobank	Prospective cohort	China	Both	30	79	Self-report	Administrative medical records or disease registries	Mortality	243,539	177,583	695	10.2	N/A	N/A	N/A	N/A	Home	Family (anyone in household)	Never smokers
Tracheal, bronchus, and lung cancer	Consonni 2018	Environment And Genetics in Lung cancer Etiology (EAGLE) study	Case-control	Lombardia	Both	35	79	Self-report	Administrative medical records or disease registries; Biomarker	Morbidity	N/A	N/A	N/A	N/A	N/A	1,318	1,368	Community	Home or work	Any	Any smoking status

Table S3. Summary of study characteristics

Health outcome	Author-Year	Study name	Study design	Location	Sex	Age start	Age end	Exposure assessment	Outcome assessment	Endpoint	Sample size	Exposed	Events	Follow-up (years)	Person-years	Cases	Controls	Control pool	Exposure location	Exposure source	Smoking status
Tracheal, bronchus, and lung cancer	Dalager 1986	N/A	Case-control	Louisiana; New Jersey; Texas	Both	18	99	Self-report	Administrative medical records or disease registries	Morbidity	N/A	N/A	N/A	N/A	N/A	48	466	Hospital	Home	Spouse	Never smokers
Tracheal, bronchus, and lung cancer	Davis 2018	N/A	Case-control	Czechia; Hungary; Mazowieckie; Romania; Russian Federation; Slovenia	Female	20	75	Self-report	Administrative medical records or disease registries; Biomarker	Morbidity	N/A	N/A	N/A	N/A	N/A	86	181	Hospital	Home or work	Any	Never smokers
Tracheal, bronchus, and lung cancer	Du 1995	N/A	Case-control	Guangdong	Female	18	99	Self-report	Administrative medical records or disease registries	Mortality	N/A	N/A	N/A	N/A	N/A	75	128	Community	Home	Spouse	Never smokers
Tracheal, bronchus, and lung cancer	Enstrom 2003	American Cancer Society Cancer Prevention Study (CPS I); California Cancer Prevention Study (CPS I)	Prospective cohort	California	Both	35	84	Self-report	Death certificates; Administrative medical records or disease registries	Mortality	28,079	13,222	200	38	N/A	N/A	N/A	N/A	Home	Spouse	Never smokers
Tracheal, bronchus, and lung cancer	Ferreccio 2013	N/A	Case-control	Chile	Both	30	99	Self-report	Physician diagnosis; Administrative medical records or disease registries; Biomarker	Morbidity	N/A	N/A	N/A	N/A	N/A	59	242	Community	Home	Family (anyone in household)	Never smokers
Tracheal, bronchus, and lung cancer	Fontham 1994	N/A	Case-control	United States of America	Female	20	79	Self-report; Proxy respondent	Administrative medical records or disease registries	Morbidity & Mortality	N/A	N/A	N/A	N/A	N/A	1,262	2,500	Community	Home; Work	Family (anyone in household); Any	Never smokers

Table S3. Summary of study characteristics

Health outcome	Author-Year	Study name	Study design	Location	Sex	Age start	Age end	Exposure assessment	Outcome assessment	Endpoint	Sample size	Exposed	Events	Follow-up (years)	Person-years	Cases	Controls	Control pool	Exposure location	Exposure source	Smoking status
Tracheal, bronchus, and lung cancer	Franco-Marina 2006	N/A	Case-control	Mexico City; México	Both	47	102	Self-report	Administrative medical records or disease registries	Morbidity	N/A	N/A	N/A	N/A	N/A	385	898	Community	Home	Family (anyone in household)	Any smoking status
Tracheal, bronchus, and lung cancer	Galeone 2008	N/A	Case-control	Heilongjiang	Both	18	99	Self-report	Administrative medical records or disease registries	Morbidity	N/A	N/A	N/A	N/A	N/A	60	216	Hospital	Home and work	Any	Never smokers
Tracheal, bronchus, and lung cancer	Gallegos-Arreola 2008	N/A	Case-control	Jalisco	Both	18	88	Self-report	Administrative medical records or disease registries	Morbidity	N/A	N/A	N/A	N/A	N/A	32	138	Community	Other	Any	Never smokers
Tracheal, bronchus, and lung cancer	Gao 1987	N/A	Case-control	Shanghai	Female	35	69	Self-report	Administrative medical records or disease registries	Morbidity	N/A	N/A	N/A	N/A	N/A	672	735	Community	Home	Family (anyone in household)	Any smoking status
Tracheal, bronchus, and lung cancer	Garfinkel 1985	N/A	Case-control	United States of America	Female	40	99	Self-report; Proxy respondent	Administrative medical records or disease registries	Morbidity & Mortality	N/A	N/A	N/A	N/A	N/A	134	402	Hospital	Home	Spouse	Never smokers
Tracheal, bronchus, and lung cancer	Ger 1993	N/A	Case-control	Taiwan (Province of China)	Both	18	99	Self-report	Biomarker	Morbidity	N/A	N/A	N/A	N/A	N/A	131	262	Hospital	Home; Work	Spouse; Other family (not spouse); Any	Any smoking status
Tracheal, bronchus, and lung cancer	Gorlova 2008	N/A	Case-control	Texas	Both	18	99	Self-report	Administrative medical records or disease registries	Morbidity	N/A	N/A	N/A	N/A	N/A	266	428	Hospital	Home or work	Any	Never smokers
Tracheal, bronchus, and lung cancer	Han 2017	N/A	Case-control	Hong Kong Special Administrative Region of China; Macao Special Administrati	Both	19	99	Self-report	Administrative medical records or disease registries; Biomarker	Morbidity	N/A	N/A	N/A	N/A	N/A	99	99	Community	Home; Work	Family (anyone in household); Any	Never smokers

Table S3. Summary of study characteristics

Health outcome	Author-Year	Study name	Study design	Location	Sex	Age start	Age end	Exposure assessment	Outcome assessment	Endpoint	Sample size	Exposed	Events	Follow-up (years)	Person-years	Cases	Controls	Control pool	Exposure location	Exposure source	Smoking status
				ve Region of China																	
Tracheal, bronchus, and lung cancer	Hansen 2021	Norwegian Women and Cancer (NOWAC) Study	Prospective cohort	Norway	Female	31	70	Self-report	Administrative medical records or disease registries	Incidence	43,713	25,082	96	15.9	708,075	N/A	N/A	N/A	Home	Family (anyone in household)	Never smokers
Tracheal, bronchus, and lung cancer	He 2012	N/A	Prospective cohort	Shaanxi	Both	51.5	87.8	Self-report	Death certificates; Administrative medical records or disease registries; Biomarker	Mortality	910	611	16	15.4	N/A	N/A	N/A	N/A	Home or work	Any	Never smokers
Tracheal, bronchus, and lung cancer	He 2013	N/A	Case-control	Fujian	Both	18	99	Self-report	Physician diagnosis	Morbidity	N/A	N/A	N/A	N/A	N/A	507	662	Community	Home or work	Any	Any smoking status
Tracheal, bronchus, and lung cancer	He 2017	N/A	Case-control	Fujian	Female	40	99	Self-report	Administrative medical records or disease registries; Biomarker	Morbidity	N/A	N/A	N/A	N/A	N/A	292	202	Hospital	Home or work	Any	Any smoking status
Tracheal, bronchus, and lung cancer	Hernández-Garduño 2004	N/A	Case-control	Mexico	Female	44	93	Self-report	Administrative medical records or disease registries	Morbidity	N/A	N/A	N/A	N/A	N/A	113	273	Hospital	Home	Family (anyone in household)	Never smokers
Tracheal, bronchus, and lung cancer	Hill 2007	New Zealand Census Mortality Study	Prospective cohort	New Zealand	Both	45	74	Self-report	Death certificates	Mortality	320,931	53,469	207	3	952,726	N/A	N/A	N/A	Home	Family (anyone in household)	Never smokers
Tracheal, bronchus, and lung cancer	Hirayama 1984	N/A	Prospective cohort	Japan	Both	40	99	Self-report	Death certificates	Mortality	80,156	38,982	183	16	N/A	N/A	N/A	N/A	Home	Spouse	Never smokers
Tracheal, bronchus,	Hole 1989	N/A	Prospective cohort	Scotland	Both	45	64	Self-report	Administrative medical records or	Mortality	1,784	1,295	6	11.5	N/A	N/A	N/A	N/A	Home	Family (anyone in household)	Never smokers

Table S3. Summary of study characteristics

Health outcome	Author-Year	Study name	Study design	Location	Sex	Age start	Age end	Exposure assessment	Outcome assessment	Endpoint	Sample size	Exposed	Events	Follow-up (years)	Person-years	Cases	Controls	Control pool	Exposure location	Exposure source	Smoking status
and lung cancer									disease registries; Death certificates												
Tracheal, bronchus, and lung cancer	Humble 1987	N/A	Case-control	New Mexico	Both	18	84	Self-report; Proxy respondent	Administrative medical records or disease registries	Morbidity & Mortality	N/A	N/A	N/A	N/A	N/A	28	292	Community	Home	Spouse	Never smokers
Tracheal, bronchus, and lung cancer	Janerich 1990	N/A	Case-control	New York	Both	20	80	Self-report	Administrative medical records or disease registries	Morbidity	N/A	N/A	N/A	N/A	N/A	129	129	Community	Home	Spouse	Never smokers
Tracheal, bronchus, and lung cancer	Jee 1999	N/A	Prospective cohort	Republic of Korea	Female	40	88	Self-report	Administrative medical records or disease registries	Incidence	120,634	84,525	63	3.5	N/A	N/A	N/A	N/A	Home	Spouse	Never smokers
Tracheal, bronchus, and lung cancer	Jin 2014	N/A	Case-control	Jiangsu	Both	18	99	Self-report	Administrative medical records or disease registries	Morbidity	N/A	N/A	N/A	N/A	N/A	1,424	4,543	Community	Home	Family (anyone in household)	Any smoking status
Tracheal, bronchus, and lung cancer	Johnson 2001	National Enhanced Cancer Surveillance System	Case-control	Canada	Female	20	75	Self-report	Administrative medical records or disease registries	Morbidity	N/A	N/A	N/A	N/A	N/A	79	840	Community	Home and work	Any	Never smokers
Tracheal, bronchus, and lung cancer	Jöckel 1998	N/A	Case-control	Germany	Both	31	42	Self-report	Physician diagnosis; Biomarker	Morbidity	N/A	N/A	N/A	N/A	N/A	71	276	Community	Home	Spouse	Non-smokers (former and never)
Tracheal, bronchus, and lung cancer	Kabat 1984	N/A	Case-control	United States of America	Both	18	99	Self-report	Administrative medical records or disease registries	Morbidity	N/A	N/A	N/A	N/A	N/A	78	78	Hospital	Home; Work	Family (anyone in household); Any	Never smokers
Tracheal, bronchus,	Kabat 1995	N/A	Case-control	New York; Illinois;	Both	20	80	Self-report	Administrative medical records or	Morbidity	N/A	N/A	N/A	N/A	N/A	106	271	Hospital	Home	Spouse	Never smokers

Table S3. Summary of study characteristics

Health outcome	Author-Year	Study name	Study design	Location	Sex	Age start	Age end	Exposure assessment	Outcome assessment	Endpoint	Sample size	Exposed	Events	Follow-up (years)	Person-years	Cases	Controls	Control pool	Exposure location	Exposure source	Smoking status
and lung cancer				Michigan; Pennsylvania					disease registries; Biomarker												
Tracheal, bronchus, and lung cancer	Kabat 1996	N/A	Case-control	United States of America	Both	20	80	Self-report	Physician diagnosis; Biomarker	Morbidity	N/A	N/A	N/A	N/A	N/A	106	271	Hospital	Home	Spouse	Never smokers
Tracheal, bronchus, and lung cancer	Kalandidi 1990	N/A	Case-control	Greece	Female	35	99	Self-report	Biomarker	Morbidity	N/A	N/A	N/A	N/A	N/A	91	120	Hospital	Home	Spouse	Never smokers
Tracheal, bronchus, and lung cancer	Koo 1987	N/A	Case-control	Hong Kong Special Administrative Region of China	Female	18	99	Self-report	Administrative medical records or disease registries	Morbidity	N/A	N/A	N/A	N/A	N/A	113	183	Community	Home	Family (anyone in household)	Never smokers
Tracheal, bronchus, and lung cancer	Kurahashi 2008	Japan Public Health Center-based Prospective Study (JPHC Study) cohorts 1 and 2	Prospective cohort	Akita; Iwate; Nagano; Okinawa; Ibaraki; Niigata; Kōchi; Nagasaki	Female	40	69	Self-report	Administrative medical records or disease registries; Death certificates	Incidence & Mortality	N/A	N/A	106	13.3	380,049	N/A	N/A	N/A	Home; Work	Spouse; Any	Never smokers
Tracheal, bronchus, and lung cancer	Lam 1987	N/A	Case-control	Hong Kong Special Administrative Region of China	Female	18	99	Self-report	Physician diagnosis; Biomarker	Morbidity	N/A	N/A	N/A	N/A	N/A	199	335	Community	Home	Spouse	Never smokers
Tracheal, bronchus, and lung cancer	Lan 2008	N/A	Case-control	Yunnan	Both	18	85	Self-report	Physician diagnosis; Biomarker	Morbidity	N/A	N/A	N/A	N/A	N/A	498	498	Community	Home	Family (anyone in household)	Any smoking status
Tracheal, bronchus, and lung cancer	Lee 1986	N/A	Case-control	England	Both	35	74	Self-report	Physician diagnosis; Administrative medical records or disease registries	Morbidity	N/A	N/A	N/A	N/A	N/A	47	96	Hospital	Home	Spouse	Never smokers

Table S3. Summary of study characteristics

Health outcome	Author-Year	Study name	Study design	Location	Sex	Age start	Age end	Exposure assessment	Outcome assessment	Endpoint	Sample size	Exposed	Events	Follow-up (years)	Person-years	Cases	Controls	Control pool	Exposure location	Exposure source	Smoking status
Tracheal, bronchus, and lung cancer	Lee 2000	N/A	Case-control	Taiwan (Province of China)	Female	18	99	Self-report	Administrative medical records or disease registries; Biomarker	Morbidity	N/A	N/A	N/A	N/A	N/A	931	1,496	Hospital	Home; Work	Spouse; Other family (not spouse); Any	Never smokers
Tracheal, bronchus, and lung cancer	Li 2002	N/A	Case-cohort	Shanghai	Both	24	78	Self-report	Administrative medical records or disease registries	Mortality	88	37	9	24	N/A	N/A	N/A	N/A	Work	Any	Never smokers
Tracheal, bronchus, and lung cancer	Li 2020	N/A	Prospective cohort	Shanghai	Both	23	79	Self-report	Administrative medical records or disease registries	Incidence	17,789	9,110	152	9.1	156,566	N/A	N/A	N/A	Home or work	Any	Never smokers
Tracheal, bronchus, and lung cancer	Liang 2009	N/A	Case-control	Liaoning	Female	20	80	Self-report	Physician diagnosis	Morbidity	N/A	N/A	N/A	N/A	N/A	226	279	Community	Other	Any	Never smokers
Tracheal, bronchus, and lung cancer	Liang 2019	N/A	Case-control	Hebei	Both	15	99	Self-report	Administrative medical records or disease registries; Biomarker	Morbidity	N/A	N/A	N/A	N/A	N/A	1,013	1,340	Hospital	Work; Home	Any; Family (anyone in household)	Never smokers
Tracheal, bronchus, and lung cancer	Liu 1991	N/A	Case-control	Yunnan	Female	18	99	Self-report	Biomarker; Administrative medical records or disease registries	Morbidity	N/A	N/A	N/A	N/A	N/A	54	202	Community	Home	Family (anyone in household)	Any smoking status
Tracheal, bronchus, and lung cancer	Masjedi 2013	N/A	Case-control	Tehran	Both	18	99	Self-report	Physician diagnosis; Administrative medical records or disease registries; Biomarker	Morbidity	N/A	N/A	N/A	N/A	N/A	81	289	Hospital	Other	Any	Unspecified

Table S3. Summary of study characteristics

Health outcome	Author-Year	Study name	Study design	Location	Sex	Age start	Age end	Exposure assessment	Outcome assessment	Endpoint	Sample size	Exposed	Events	Follow-up (years)	Person-years	Cases	Controls	Control pool	Exposure location	Exposure source	Smoking status
Tracheal, bronchus, and lung cancer	Mbeje 2022	N/A	Case-control	KwaZulu-Natal	Both	21	99	Self-report	Self-report	Morbidity	N/A	N/A	N/A	N/A	N/A	75	159	Hospital	Home or work	Any	Any smoking status
Tracheal, bronchus, and lung cancer	McGhee 2005	N/A	Case-control	Hong Kong Special Administrative Region of China	Both	60	99	Proxy respondent	Death certificates	Mortality	N/A	N/A	N/A	N/A	N/A	524	763	Community	Home	Family (anyone in household)	Never smokers
Tracheal, bronchus, and lung cancer	Miller 2003	N/A	Case-control	Massachusetts	Both	27	96	Self-report	Physician diagnosis; Biomarker	Morbidity	N/A	N/A	N/A	N/A	N/A	56	408	Hospital	Other	Any	Never smokers
Tracheal, bronchus, and lung cancer	Minichilli 2022	PROTOS (Pulmonary cancer and Risk factors for Tumors, Observational Study) Case-Control Study	Case-control	Puglia	Both	18	99	Self-report	Biomarker	Morbidity	N/A	N/A	N/A	N/A	N/A	351	1,053	Community	Other	Any	Any smoking status
Tracheal, bronchus, and lung cancer	Ng 2005	N/A	Case-control	Singapore	Female	18	99	Self-report	Physician diagnosis; Administrative medical records or disease registries; Biomarker	Morbidity	N/A	N/A	N/A	N/A	N/A	190	237	Hospital	Home	Family (anyone in household)	Never smokers
Tracheal, bronchus, and lung cancer	Nishino 2001	N/A	Prospective cohort	Miyagi	Female	40	99	Self-report	Administrative medical records or disease registries	Incidence	N/A	N/A	14	7.6	38,112	N/A	N/A	N/A	Home	Family (anyone in household)	Never smokers
Tracheal, bronchus, and lung cancer	Phukan 2014	N/A	Case-control	Mizoram	Female	18	99	Self-report	Administrative medical records or disease registries; Biomarker	Morbidity	N/A	N/A	N/A	N/A	N/A	230	460	Community	Home	Family (anyone in household)	Any smoking status

Table S3. Summary of study characteristics

Health outcome	Author-Year	Study name	Study design	Location	Sex	Age start	Age end	Exposure assessment	Outcome assessment	Endpoint	Sample size	Exposed	Events	Follow-up (years)	Person-years	Cases	Controls	Control pool	Exposure location	Exposure source	Smoking status
Tracheal, bronchus, and lung cancer	Pirie 2016	Million Women Study	Prospective cohort	United Kingdom	Female	50	84	Self-report	Administrative medical records or disease registries	Incidence	342,217	42,940	125	14	N/A	N/A	N/A	N/A	Home	Family (anyone in household)	Never smokers
Tracheal, bronchus, and lung cancer	Rapiti 1999	N/A	Case-control	Other Union Territories	Both	18	99	Self-report	Physician diagnosis; Biomarker	Morbidity	N/A	N/A	N/A	N/A	N/A	56	106	Hospital	Home	Spouse	Never smokers
Tracheal, bronchus, and lung cancer	Raspanti 2016	N/A	Case-control	Nepal	Both	18	99	Self-report	Administrative medical records or disease registries; Biomarker	Morbidity	N/A	N/A	N/A	N/A	N/A	174	150	Hospital	Home or work	Any	Any smoking status
Tracheal, bronchus, and lung cancer	Ren 2013	N/A	Case-control	Liaoning	Female	18	99	Self-report	Physician diagnosis; Biomarker	Morbidity	N/A	N/A	N/A	N/A	N/A	764	983	Hospital	Other	Any	Never smokers
Tracheal, bronchus, and lung cancer	Robles 2014	Mayo Clinic study	Case-control	Minnesota	Both	18	99	Self-report	Self-report; Administrative medical records or disease registries	Morbidity	N/A	N/A	N/A	N/A	N/A	321	179	Community	Other	Any	Never smokers
Tracheal, bronchus, and lung cancer	Rylander 2006	N/A	Case-control	Sweden	Both	18	75	Self-report	Administrative medical records or disease registries	Morbidity	N/A	N/A	N/A	N/A	N/A	152	652	Community	Work	Any	Non-smokers (former and never)
Tracheal, bronchus, and lung cancer	Sasco 2002	N/A	Case-control	Morocco	Both	34	82	Self-report	Physician diagnosis; Biomarker	Morbidity	N/A	N/A	N/A	N/A	N/A	118	235	Hospital	Home or work	Any	Any smoking status
Tracheal, bronchus, and lung cancer	Schwartz 2007	N/A	Case-control	Michigan	Female	18	74	Self-report	Administrative medical records or disease registries	Morbidity	N/A	N/A	N/A	N/A	N/A	487	993	Community	Home; Work	Family (anyone in household); Any	Any smoking status
Tracheal, bronchus,	Seki 2013	N/A	Case-control	Miyagi	Both	30	99	Self-report	Administrative medical	Morbidity	N/A	N/A	N/A	N/A	N/A	362	2,410	Hospital	Home	Spouse	Never smokers

Table S3. Summary of study characteristics

Health outcome	Author-Year	Study name	Study design	Location	Sex	Age start	Age end	Exposure assessment	Outcome assessment	Endpoint	Sample size	Exposed	Events	Follow-up (years)	Person-years	Cases	Controls	Control pool	Exposure location	Exposure source	Smoking status
and lung cancer									records or disease registries												
Tracheal, bronchus, and lung cancer	Shen 1998	N/A	Case-control	Jiangsu	Female	18	99	Self-report	Administrative medical records or disease registries	Morbidity	N/A	N/A	N/A	N/A	N/A	70	70	Community	Home	Family (anyone in household)	Never smokers
Tracheal, bronchus, and lung cancer	Sloan 2012	New England Lung Cancer Study (NELCS)	Case-control	New Hampshire; Vermont	Both	30	74	Self-report	Administrative medical records or disease registries	Morbidity	N/A	N/A	N/A	N/A	N/A	441	300	Community	Home	Family (anyone in household)	Any smoking status
Tracheal, bronchus, and lung cancer	Sobue 1990	N/A	Case-control	Ōsaka	Female	40	79	Self-report	Administrative medical records or disease registries	Morbidity	N/A	N/A	N/A	N/A	N/A	144	731	Hospital	Home	Other family (not spouse)	Never smokers
Tracheal, bronchus, and lung cancer	Speizer 1999	Nurses' Health Study	Prospective cohort	United States of America	Female	30	55	Self-report	Self-report; Death certificates; Administrative medical records or disease registries	Incidence & Mortality	N/A	N/A	35	15.2	776,300	N/A	N/A	N/A	Other	Any	Never smokers
Tracheal, bronchus, and lung cancer	Spitz 2011	N/A	Case-control	Texas	Both	18	99	Self-report	Administrative medical records or disease registries	Morbidity	N/A	N/A	N/A	N/A	N/A	335	368	Hospital	Other	Any	Never smokers
Tracheal, bronchus, and lung cancer	Stockwell 1992	N/A	Case-control	Florida	Female	18	99	Self-report; Proxy respondent	Administrative medical records or disease registries	Morbidity & Mortality	N/A	N/A	N/A	N/A	N/A	108	174	Community	Home	Spouse	Never smokers
Tracheal, bronchus, and lung cancer	Sun 2017	Nord-Trondelag Health Survey 2 (HUNT 2)	Prospective cohort	Trøndelag	Both	20	99	Self-report	Administrative medical records or disease registries	Incidence	N/A	N/A	670	16.8	N/A	N/A	N/A	N/A	Home	Family (anyone in household)	Any smoking status

Table S3. Summary of study characteristics

Health outcome	Author-Year	Study name	Study design	Location	Sex	Age start	Age end	Exposure assessment	Outcome assessment	Endpoint	Sample size	Exposed	Events	Follow-up (years)	Person-years	Cases	Controls	Control pool	Exposure location	Exposure source	Smoking status
Tracheal, bronchus, and lung cancer	Svensson 1989	N/A	Case-control	Sweden	Female	18	99	Self-report	Administrative medical records or disease registries; Physician diagnosis; Biomarker	Morbidity	N/A	N/A	N/A	N/A	N/A	27	150	Hospital	Home or work	Any	Never smokers
Tracheal, bronchus, and lung cancer	Torres-Durán 2015	N/A	Case-control	Spain	Both	34	90	Self-report	Physician diagnosis	Morbidity	N/A	N/A	N/A	N/A	N/A	212	317	Hospital	Home	Family (anyone in household)	Never smokers
Tracheal, bronchus, and lung cancer	Tubío-Pérez 2022	N/A	Case-control	Spain	Both	36	99	Self-report	Biomarker	Morbidity	N/A	N/A	N/A	N/A	N/A	382	505	Hospital	Home	Family (anyone in household)	Never smokers
Tracheal, bronchus, and lung cancer	Veglia 2007	European Prospective Investigation into Cancer and Nutrition (EPIC)	Prospective cohort	Spain; Italy; Denmark; United Kingdom; Greece; Germany	Both	35	74	Self-report	Administrative medical records or disease registries; Death certificates	Incidence & Mortality	217,055	N/A	809	6.1	N/A	N/A	N/A	N/A	Work	Any	Unspecified
Tracheal, bronchus, and lung cancer	Villeneuve 2014	N/A	Case-control	Canada	Both	20	84	Self-report	Physician diagnosis; Administrative medical records or disease registries; Biomarker	Morbidity	N/A	N/A	N/A	N/A	N/A	409	374	Community	Home; Work; Home and work	Family (anyone in household); Any	Any smoking status
Tracheal, bronchus, and lung cancer	Wang 1994	N/A	Case-control	Heilongjiang	Female	30	69	Self-report	Administrative medical records or disease registries	Morbidity	N/A	N/A	N/A	N/A	N/A	55	55	Hospital	Home	Family (anyone in household)	Non-smokers (former and never)
Tracheal, bronchus, and lung cancer	Wang 1996	N/A	Case-control	Guangdong	Both	32	78	Self-report	Physician diagnosis; Biomarker	Morbidity	N/A	N/A	N/A	N/A	N/A	390	390	Hospital	Home; Work	Family (anyone in household); Any	Any smoking status

Table S3. Summary of study characteristics

Health outcome	Author-Year	Study name	Study design	Location	Sex	Age start	Age end	Exposure assessment	Outcome assessment	Endpoint	Sample size	Exposed	Events	Follow-up (years)	Person-years	Cases	Controls	Control pool	Exposure location	Exposure source	Smoking status
Tracheal, bronchus, and lung cancer	Wang 1996	N/A	Case-control	Liaoning	Female	35	69	Self-report	Administrative medical records or disease registries; Biomarker; Physician diagnosis	Morbidity	N/A	N/A	N/A	N/A	N/A	135	135	Community	Work	Any	Never smokers
Tracheal, bronchus, and lung cancer	Wang 2009	N/A	Case-control	Hong Kong Special Administrative Region of China	Female	30	79	Self-report	Physician diagnosis; Biomarker	Morbidity	N/A	N/A	N/A	N/A	N/A	243	338	Community	Home or work; Home and work	Any	Never smokers
Tracheal, bronchus, and lung cancer	Wang 2015	Women's Health Initiative Observational Study (WHI-OS)	Prospective cohort	United States of America	Female	50	79	Self-report	Self-report; Administrative medical records or disease registries	Incidence	39,771	51,524	152	10.5	N/A	N/A	N/A	N/A	Home; Work	Family (anyone in household); Any	Never smokers
Tracheal, bronchus, and lung cancer	Weiss 2008	Shanghai Women's Health Study	Prospective cohort	Shanghai	Female	40	70	Self-report	Administrative medical records or disease registries	Incidence	N/A	N/A	198	4.1	506,522	N/A	N/A	N/A	Home or work	Family (anyone in household)	Non-smokers (former and never)
Tracheal, bronchus, and lung cancer	Wen 2006	Shanghai Women's Health Study	Prospective cohort	Shanghai	Female	40	70	Self-report	Administrative medical records or disease registries; Death certificates	Mortality	65,180	39,795	157	5.7	N/A	N/A	N/A	N/A	Home	Spouse	Never smokers
Tracheal, bronchus, and lung cancer	Wenzlaff 2005	N/A	Case-control	Michigan	Both	18	99	Self-report	Administrative medical records or disease registries	Morbidity	N/A	N/A	N/A	N/A	N/A	155	181	Hospital	Home	Family (anyone in household)	Never smokers
Tracheal, bronchus, and lung cancer	Wu 1985	N/A	Case-control	California	Female	18	75	Self-report	Administrative medical records or disease registries	Morbidity	N/A	N/A	N/A	N/A	N/A	N/A	N/A	Community	Home; Work	Spouse; Any	Any smoking status

Table S3. Summary of study characteristics

Health outcome	Author-Year	Study name	Study design	Location	Sex	Age start	Age end	Exposure assessment	Outcome assessment	Endpoint	Sample size	Exposed	Events	Follow-up (years)	Person-years	Cases	Controls	Control pool	Exposure location	Exposure source	Smoking status
Tracheal, bronchus, and lung cancer	Xu 2020	N/A	Case-control	Fujian	Both	18	99	Self-report	Biomarker	Morbidity	N/A	N/A	N/A	N/A	N/A	449	512	Community	Other	Any	Any smoking status
Tracheal, bronchus, and lung cancer	Yang 2015	N/A	Case-control	Guangdong; Jiangsu	Both	18	99	Self-report	Physician diagnosis; Administrative medical records or disease registries; Biomarker	Morbidity	N/A	N/A	N/A	N/A	N/A	354	364	Community	Home	Family (anyone in household)	Never smokers
Tracheal, bronchus, and lung cancer	Yang 2016	Genetic Epidemiological Study of Lung Adenocarcinoma (GELAC)	Case-control	Taiwan (Province of China)	Both	18	99	Self-report	Administrative medical records or disease registries; Biomarker	Morbidity	N/A	N/A	N/A	N/A	N/A	709	726	Hospital	Other	Any	Any smoking status
Tracheal, bronchus, and lung cancer	Yin 2014	N/A	Case-control	Liaoning	Female	18	99	Self-report	Physician diagnosis; Biomarker	Morbidity	N/A	N/A	N/A	N/A	N/A	306	318	Hospital	Other	Any	Never smokers
Tracheal, bronchus, and lung cancer	Yoon 2008	N/A	Case-control	Republic of Korea	Female	30	71	Self-report	Biomarker	Morbidity	N/A	N/A	N/A	N/A	N/A	213	213	Hospital	Home; Work	Spouse; Paternal; Maternal; Any	Never smokers
Tracheal, bronchus, and lung cancer	Zaridze 1998	N/A	Case-control	Moscow City	Female	18	99	Self-report	Administrative medical records or disease registries; Biomarker	Morbidity	N/A	N/A	N/A	N/A	N/A	189	358	Hospital	Home; Work	Spouse; Other family (not spouse); Any	Never smokers
Tracheal, bronchus, and lung cancer	Zatloukal 2003	N/A	Case-control	Czechia	Female	25	89	Self-report	Administrative medical records or disease registries; Biomarker	Morbidity	N/A	N/A	N/A	N/A	N/A	74	889	Hospital	Other	Any	Never smokers
Tracheal, bronchus,	Zhong 1999	N/A	Case-control	Shanghai	Female	35	69	Self-report	Administrative medical records or	Morbidity	N/A	N/A	N/A	N/A	N/A	545	651	Community	Home or work	Any	Never smokers

Table S3. Summary of study characteristics

Health outcome	Author-Year	Study name	Study design	Location	Sex	Age start	Age end	Exposure assessment	Outcome assessment	Endpoint	Sample size	Exposed	Events	Follow-up (years)	Person-years	Cases	Controls	Control pool	Exposure location	Exposure source	Smoking status
and lung cancer									disease registries												
Tracheal, bronchus, and lung cancer	Zhuang 2022	N/A	Case-control	Fujian	Both	18	99	Self-report	Biomarker	Morbidity	N/A	N/A	N/A	N/A	N/A	623	985	Community	Other	Any	Non-smokers (former and never)

N/A, not available

Section 2.2: Effect size data details

From the extracted data points, we identified those that were eligible for inclusion in our models in order to capture the observation(s) from each included study that would best inform our estimates. This process was undertaken because studies often reported several effect sizes estimated from the same populations for the same health outcome but with slightly different parameters or definitions. For example, a study might report the risk associated with being exposed to SHS at different settings and/or from different sources; the risk associated with current exposure and with ever exposure; health risks for both specific age groups and for all ages combined; different disease endpoints (e.g.; mortality or incidence); models with different degrees of adjustment for potential confounders; slightly different outcome definitions; etc. Including all extracted data points in the models would introduce unnecessary noise and over-represent studies that happened to report more observations.

In order to address this challenge, we applied a data point selection process in which each study's extracted data points were evaluated using the stepwise criteria described below to identify the most adjusted effect sizes with the closest match to our exposure case definition, our outcome definition, and largest sample size for each analytic sample. This approach allowed us to select the preferred data points for inclusion without entirely dropping any of the included studies.

1. <u>Best match exposure definition:</u>

   From each study, we selected the observations that best matched the SHS exposure definition used in the GBD in the following order: First, we selected data points based on the smoking status of the assessed population. Therefore, when available within each study, we selected data points reporting the risk for non-smokers (former and never smokers combined); in the absence of that, we selected data points for former and/or never smokers; finally, if the previous smoking status were not available, we accepted observations with unspecified/any smoking status. Second, we selected observations based on the setting of exposure, where we prioritized those reporting an effect size for home or work exposure combined, followed by home and/or work separately, and finally, those reporting on a broad/unspecified location definition. Third, from the remaining data points, we further selected the ones for inclusion in our models based on the source of exposure, always prioritizing more aggregated definitions (e.g., any or parental) over more specific ones (e.g., maternal or paternal). Finally, if a study reported an effect size for both ever and current SHS exposure, we preferred the observation of the risk associated with current exposure.

2. <u>Non-age- or sex-specific observations:</u>

   Because we don't compute age or sex-specific pooled effect sizes associated with exposure to SHS in in the GBD, we selected the observations reporting the risk for both sexes and all age-groups combined. In case either of both of these were not available, all available sex-specific and/or age-specific observations from a study were retained in this phase.

3. <u>Combined sub-groups:</u>

   Following, if multiple observations met the above criteria, these observations were frequently from studies that reported effect sizes for other aggregated and disaggregated sub-groups. Examples of these included results from sensitivity analysis results (e.g., separate analyses for pre- and post-menopause women). When possible, we preferred the effect sizes reported for combined sub-

groups over those that were disaggregated so our models could be informed by the largest possible sample sizes.

4. Best match outcome definition:

We selected the observations from each study that best matched the GBD preferred outcome definitions. For stroke, studies that reported effect sizes for both the aggregate outcome and each (or any) of the sub-types, we selected the aggregated outcome definition, since this one was the most common case across the included stroke studies. In the absence of the aggregated definition, we include the effect sizes associated with all reported sub-types.

5. Best match our disease endpoints:

In this next step, we checked the remaining data points for multiple disease endpoints reported within each study. When possible, we selected effect sizes matched to mortality and morbidity combined over those for specific endpoints.

6. Most adjusted effect size reported:

Finally, if multiple observations from the same study met the above criteria, we selected the most adjusted effect size reported.

In the cases where a study still had multiple eligible observations selected after this stepwise process, we manually examined the differences in the observations to determine whether or not multiple rows should still be included in our models. After the selection phase, to avoid overrepresentation of the studies with multiple observations associated with non-mutually exclusive populations, we down-weighted the data points based on the number of overlapping observations within each sex-age-smoking status combination from that study using the following equation:

$$standard\ error\ used\ for\ modeling = \sqrt{number\ of\ overlapping\ observations} \times observed\ standard\ error$$

In Tables S4-S12, we display the effect sizes from each study that were selected for inclusion in our models. For information purposes, we show both the original standard error and the adjusted one (based on the number of overlapping observations, as explained above). The outlier column indicates if the observation was trimmed as part of the Burden of Proof methodology. Before running our models, to best adjust for the smoking status of the studied population, we reassigned all “unknown” smoking status to “adjusted never-smokers” if the study only included kids aged 15 years or less. We also considered those cases “controlled for smoking”, since we assume all of the individuals are never smokers. Those observations were treated as “never-smokers” in our models, but we assign them a separate smoking status category for transparency.

Table S4. Summary of data inputs ischemic heart disease

Author	Study design	Health outcome	Sex	Age	Exposure location	Exposure source	Smoking status	Log effect size	Original SE of the log effect size	SE of the log effect size used in the model	Outlier
Attard 2017	Case-control	Ischemic heart disease	Both	20 - 77	Home	Family (anyone in household)	Non-smokers (former and never)	0.693	0.530	0.530	No
Awawdi 2016	Case-control	Ischemic heart disease	Female	35 - 70	Home	Spouse	Never smokers	1.300	0.652	0.652	Yes
Ciruzzi 1998	Case-control	Ischemic heart disease	Both	24 - 83	Home	Family (anyone in household)	Never smokers	0.519	0.174	0.174	No
Clark 2013	Prospective cohort	Ischemic heart disease	Both	45 - 74	Home or work	Any	Never smokers	0.000	0.153	0.153	No
Ding 2009	Case-control	Ischemic heart disease	Female	18 - 99	Home	Family (anyone in household)	Never smokers	0.419	0.207	0.207	No
Diver 2018	Prospective cohort	Ischemic heart disease	Both	50 - 74	Home	Family (anyone in household)	Never smokers	0.270	0.081	0.081	No
Dobson 1991	Case-control	Ischemic heart disease	Male	35 - 69	Home	Family (anyone in household)	Never smokers	-0.030	0.335	0.474	No
Dobson 1991	Case-control	Ischemic heart disease	Male	35 - 69	Home	Family (anyone in household)	Former smokers	0.577	0.231	0.326	No
Dobson 1991	Case-control	Ischemic heart disease	Female	35 - 69	Home	Family (anyone in household)	Never smokers	0.900	0.264	0.373	No
Dobson 1991	Case-control	Ischemic heart disease	Female	35 - 69	Home	Family (anyone in household)	Former smokers	0.392	0.407	0.575	No

Table S4. Summary of data inputs ischemic heart disease

Author	Study design	Health outcome	Sex	Age	Exposure location	Exposure source	Smoking status	Log effect size	Original SE of the log effect size	SE of the log effect size used in the model	Outlier
Dobson 1991	Case-control	Ischemic heart disease	Male	35 - 69	Work	Any	Never smokers	-0.051	0.319	0.451	No
Dobson 1991	Case-control	Ischemic heart disease	Male	35 - 69	Work	Any	Former smokers	-0.128	0.300	0.425	No
Dobson 1991	Case-control	Ischemic heart disease	Female	35 - 69	Work	Any	Never smokers	-0.416	0.698	0.987	No
Dobson 1991	Case-control	Ischemic heart disease	Female	35 - 69	Work	Any	Former smokers	0.793	0.973	1.376	No
Enstrom 2003	Prospective cohort	Ischemic heart disease	Male	30 - 96	Home	Spouse	Never smokers	-0.083	0.069	0.069	Yes
Enstrom 2003	Prospective cohort	Ischemic heart disease	Female	30 - 96	Home	Spouse	Never smokers	-0.030	0.045	0.045	Yes
Fatmi 2014	Case-control	Ischemic heart disease	Female	0 - 99	Home	Any	Any smoking status	0.405	0.380	0.380	No
Gallo 2010	Prospective cohort	Ischemic heart disease	Both	18 - 99	Home	Family (anyone in household)	Never smokers	0.270	0.234	0.234	No
Garland 1985	Prospective cohort	Ischemic heart disease	Female	50 - 79	Home	Spouse	Never smokers	0.737	0.991	0.991	No
He 1994	Case-control	Ischemic heart disease	Female	37 - 67	Home or work	Any	Never smokers	0.859	0.435	0.435	No
He 2012	Prospective cohort	Ischemic heart disease	Both	51.5 - 87.8	Home or work	Any	Never smokers	0.765	0.390	0.390	No
Hill 2007	Prospective cohort	Ischemic heart disease	Male	45 - 74	Home	Family (anyone in household)	Never smokers	0.039	0.085	0.121	No

Table S4. Summary of data inputs ischemic heart disease

Author	Study design	Health outcome	Sex	Age	Exposure location	Exposure source	Smoking status	Log effect size	Original SE of the log effect size	SE of the log effect size used in the model	Outlier
Hill 2007	Prospective cohort	Ischemic heart disease	Male	45 - 74	Home	Family (anyone in household)	Never smokers	0.166	0.103	0.146	No
Hill 2007	Prospective cohort	Ischemic heart disease	Female	45 - 74	Home	Family (anyone in household)	Never smokers	-0.020	0.088	0.124	No
Hill 2007	Prospective cohort	Ischemic heart disease	Female	45 - 74	Home	Family (anyone in household)	Never smokers	0.239	0.134	0.190	No
Hole 1989	Prospective cohort	Ischemic heart disease	Both	45 - 64	Home	Family (anyone in household)	Never smokers	0.698	0.260	0.260	No
Janghorbani 1997	Case-control	Ischemic heart disease	Female	42 - 85	Home	Family (anyone in household)	Never smokers	0.104	0.290	0.290	No
Kastorini 2013	Case-control	Ischemic heart disease	Both	18 - 99	Other	Any	Non-smokers (former and never)	1.466	0.535	0.535	Yes
Kawachi 1997	Prospective cohort	Ischemic heart disease	Female	30 - 55	Home or work	Any	Never smokers	0.536	0.259	0.259	No
Kobayashi 2022	Prospective cohort	Ischemic heart disease	Female	40 - 59	Other	Spouse	Never smokers	0.703	0.272	0.272	No
La 1993	Nested case-control	Ischemic heart disease	Both	29 - 74	Home	Spouse	Never smokers	0.191	0.379	0.379	No
Lee 1986	Case-control	Ischemic heart disease	Both	35 - 74	Home	Spouse	Never smokers	-0.010	0.212	0.212	No

Table S4. Summary of data inputs ischemic heart disease

Author	Study design	Health outcome	Sex	Age	Exposure location	Exposure source	Smoking status	Log effect size	Original SE of the log effect size	SE of the log effect size used in the model	Outlier
Malinauskiene 2011	Case-control	Ischemic heart disease	Female	35 - 61	Other	Any	Never smokers	0.247	0.217	0.217	No
McElduff 1998	Case-control	Ischemic heart disease	Both	35 - 69	Home or work	Any	Non-smokers (former and never)	-0.094	0.135	0.191	No
McElduff 1998	Case-control	Ischemic heart disease	Both	35 - 69	Home or work	Any	Non-smokers (former and never)	0.122	0.145	0.205	No
McElduff 1998	Case-control	Ischemic heart disease	Both	35 - 64	Home or work	Any	Non-smokers (former and never)	0.322	0.149	0.149	No
McGhee 2005	Case-control	Ischemic heart disease	Both	60 - 99	Home	Family (anyone in household)	Never smokers	0.300	0.137	0.137	No
Muscat 1995	Case-control	Ischemic heart disease	Male	18 - 74	Work	Any	Never smokers	0.182	0.331	0.331	No
Muscat 1995	Case-control	Ischemic heart disease	Female	18 - 74	Work	Any	Never smokers	0.000	0.467	0.467	No
Nishtar 2004	Case-control	Ischemic heart disease	Both	39 - 67	Home	Family (anyone in household)	Any smoking status	1.054	0.411	0.411	Yes
Notara 2015	Prospective cohort	Ischemic heart disease	Both	18 - 99	Work	Any	Unknown	-0.041	0.110	0.155	No
Notara 2015	Prospective cohort	Ischemic heart disease	Both	18 - 99	Home	Family (anyone in household)	Unknown	0.122	0.121	0.171	No

Table S4. Summary of data inputs ischemic heart disease

Author	Study design	Health outcome	Sex	Age	Exposure location	Exposure source	Smoking status	Log effect size	Original SE of the log effect size	SE of the log effect size used in the model	Outlier
Pitsavos 2002	Case-control	Ischemic heart disease	Male	18 - 99	Home or work	Any	Never smokers	0.223	0.089	0.126	No
Pitsavos 2002	Case-control	Ischemic heart disease	Male	18 - 99	Home or work	Any	Never smokers	0.385	0.091	0.129	No
Pitsavos 2002	Case-control	Ischemic heart disease	Female	18 - 99	Home or work	Any	Never smokers	0.255	0.111	0.158	No
Pitsavos 2002	Case-control	Ischemic heart disease	Female	18 - 99	Home or work	Any	Never smokers	0.445	0.141	0.199	No
Rashid 2019	Case-control	Ischemic heart disease	Female	30 - 65	Home or work	Any	Unknown	1.095	0.256	0.256	Yes
Rosenlund 2001	Case-control	Ischemic heart disease	Both	45 - 70	Home	Spouse	Never smokers	0.315	0.215	0.304	No
Rosenlund 2001	Case-control	Ischemic heart disease	Both	45 - 70	Work	Any	Never smokers	0.270	0.200	0.283	No
Rossi 2011	Case-control	Ischemic heart disease	Both	18 - 99	Home	Family (anyone in household)	Non-smokers (former and never)	0.157	0.080	0.080	No
Sadeghi 2020	Prospective cohort	Ischemic heart disease	Both	30 - 99	Home or work	Any	Never smokers	0.239	0.088	0.088	No
Sandler 1989	Prospective cohort	Ischemic heart disease	Male	25 - 99	Home	Family (anyone in household)	Never smokers	0.270	0.114	0.114	No
Sandler 1989	Prospective cohort	Ischemic heart disease	Female	25 - 99	Home	Family (anyone in household)	Never smokers	0.174	0.068	0.068	No

Table S4. Summary of data inputs ischemic heart disease

Author	Study design	Health outcome	Sex	Age	Exposure location	Exposure source	Smoking status	Log effect size	Original SE of the log effect size	SE of the log effect size used in the model	Outlier
Spencer 1999	Case-control	Ischemic heart disease	Male	25 - 69	Home	Family (anyone in household)	Any smoking status	0.357	0.150	0.212	No
Spencer 1999	Case-control	Ischemic heart disease	Male	25 - 69	Work	Any	Any smoking status	0.511	0.150	0.212	No
Steenland 1996	Prospective cohort	Ischemic heart disease	Male	30 - 99	Home	Spouse	Never smokers	0.207	0.091	0.091	No
Steenland 1996	Prospective cohort	Ischemic heart disease	Female	30 - 99	Home	Spouse	Never smokers	0.174	0.103	0.103	No
Sulo 2008	Case-control	Ischemic heart disease	Both	35 - 74	Home	Spouse	Never smokers	0.495	0.276	0.276	No
Svendsen 1987	Prospective cohort	Ischemic heart disease	Male	35 - 57	Home	Spouse	Never smokers	0.476	0.265	0.265	No

Table S5. Summary of data inputs for stroke

Author	Study design	Health outcome	Sex	Age	Exposure location	Exposure source	Smoking status	Log effect size	Original SE of the log effect size	SE of the log effect size used in the model	Outlier
Anderson 2004	Case-control	Stroke	Both	15 - 99	Home	Family (anyone in household)	Never smokers	-0.357	0.427	0.427	No
Bonita 1999	Case-control	Stroke	Both	35 - 74	Home or work	Any	Non-smokers (former and never)	0.599	0.158	0.158	Yes
Diver 2018	Prospective cohort	Stroke	Both	50 - 74	Home	Family (anyone in household)	Never smokers	0.215	0.120	0.120	No
Gallo 2010	Prospective cohort	Stroke	Both	18 - 99	Home	Family (anyone in household)	Never smokers	0.020	0.275	0.388	No
Gallo 2010	Prospective cohort	Stroke	Both	18 - 99	Work	Any	Never smokers	0.199	0.333	0.471	No
Glymour 2008	Prospective cohort	Stroke	Both	50 - 99	Home	Spouse	Never smokers	0.351	0.161	0.161	No
Glymour 2008	Prospective cohort	Stroke	Both	50 - 99	Home	Spouse	Former smokers	0.542	0.137	0.137	Yes
He 2012	Prospective cohort	Stroke	Both	51.5 - 87.8	Home or work	Any	Never smokers	0.798	0.311	0.311	Yes
Hill 2007	Prospective cohort	Stroke	Male	45 - 74	Home	Family (anyone in household)	Never smokers	0.464	0.169	0.239	No
Hill 2007	Prospective cohort	Stroke	Female	45 - 74	Home	Family (anyone in household)	Never smokers	-0.105	0.151	0.213	No

Table S5. Summary of data inputs for stroke

Author	Study design	Health outcome	Sex	Age	Exposure location	Exposure source	Smoking status	Log effect size	Original SE of the log effect size	SE of the log effect size used in the model	Outlier
Hill 2007	Prospective cohort	Stroke	Male	45 - 74	Home	Family (anyone in household)	Never smokers	0.599	0.213	0.302	No
Hill 2007	Prospective cohort	Stroke	Female	45 - 74	Home	Family (anyone in household)	Never smokers	0.157	0.223	0.315	No
Hou 2017	Case-control	Stroke	Both	30 - 98	Home	Spouse	Never smokers	0.131	0.036	0.036	No
Kastorini 2013	Case-control	Stroke	Both	18 - 99	Other	Any	Non-smokers (former and never)	0.278	0.448	0.448	No
Kobayashi 2022	Prospective cohort	Stroke	Female	40 - 69	Home	Spouse	Never smokers	-0.117	0.125	0.125	No
Lee 1986	Case-control	Stroke	Both	35 - 74	Home	Spouse	Never smokers	0.006	0.233	0.233	No
Malek 2015	Prospective cohort	Stroke	Both	45 - 99	Other	Any	Non-smokers (former and never)	0.239	0.131	0.131	No
McGhee 2005	Case-control	Stroke	Both	60 - 99	Home	Family (anyone in household)	Never smokers	0.399	0.133	0.133	No
Nishino 2014	Prospective cohort	Stroke	Female	40 - 99	Home	Family (anyone in household)	Never smokers	0.131	0.071	0.071	No
Poulsen 2021	Case-cohort	Stroke	Both	50 - 64	Home or work	Any	Never smokers	0.052	0.364	0.364	No

Table S5. Summary of data inputs for stroke

Author	Study design	Health outcome	Sex	Age	Exposure location	Exposure source	Smoking status	Log effect size	Original SE of the log effect size	SE of the log effect size used in the model	Outlier
Qureshi 2005	Prospective cohort	Stroke	Female	25 - 74	Home	Spouse	Never smokers	-0.105	0.197	0.197	No
Sandler 1989	Prospective cohort	Stroke	Male	25 - 99	Home	Family (anyone in household)	Never smokers	-0.030	0.206	0.206	No
Sandler 1989	Prospective cohort	Stroke	Female	25 - 99	Home	Family (anyone in household)	Never smokers	0.215	0.094	0.094	No
Wen 2006	Prospective cohort	Stroke	Female	40 - 70	Home	Spouse	Never smokers	0.419	0.176	0.176	No
Yamada 2003	Prospective cohort	Stroke	Both	40 - 79	Other	Any	Unknown	-0.051	0.249	0.249	No
You 1999	Case-control	Stroke	Both	18 - 99	Home	Spouse	Never smokers	0.531	0.279	0.279	No

Table S6. Summary of data inputs for tracheal, bronchus, and lung cancer

Author	Study design	Health outcome	Sex	Age	Exposure location	Exposure source	Smoking status	Log effect size	Original SE of the log effect size	SE of the log effect size used in the model	Outlier
Abdel-Rahman 2020	Prospective cohort	Tracheal, bronchus, and lung cancer	Both	55 - 74	Work	Any	Never smokers	0.712	0.224	0.449	No
Abdel-Rahman 2020	Prospective cohort	Tracheal, bronchus, and lung cancer	Both	55 - 74	Work	Any	Never smokers	-0.081	0.200	0.400	No
Abdel-Rahman 2020	Prospective cohort	Tracheal, bronchus, and lung cancer	Both	55 - 74	Home	Family (anyone in household)	Never smokers	0.593	0.226	0.453	No
Abdel-Rahman 2020	Prospective cohort	Tracheal, bronchus, and lung cancer	Both	55 - 74	Home	Family (anyone in household)	Never smokers	0.050	0.201	0.401	No
Al-Zoughool 2013	Case-control	Tracheal, bronchus, and lung cancer	Both	35 - 75	Home	Family (anyone in household)	Never smokers	-0.416	0.404	0.404	No
Asomaning 2008	Case-control	Tracheal, bronchus, and lung cancer	Both	18 - 99	Home	Family (anyone in household)	Never smokers	-0.077	0.439	0.621	No
Asomaning 2008	Case-control	Tracheal, bronchus, and lung cancer	Both	18 - 99	Work	Any	Never smokers	-0.122	0.435	0.615	No
Behera 2005	Case-control	Tracheal, bronchus, and lung cancer	Female	30 - 80	Other	Any	Any smoking status	0.698	0.454	0.454	No
Boffetta 1998	Case-control	Tracheal, bronchus, and lung cancer	Both	18 - 74	Home and work	Any	Never smokers	0.131	0.131	0.131	No

Table S6. Summary of data inputs for tracheal, bronchus, and lung cancer

Author	Study design	Health outcome	Sex	Age	Exposure location	Exposure source	Smoking status	Log effect size	Original SE of the log effect size	SE of the log effect size used in the model	Outlier
Boffetta 1999	Case-control	Tracheal, bronchus, and lung cancer	Both	18 - 99	Home or work	Any	Never smokers	0.182	0.364	0.364	No
Brownson 1992	Case-control	Tracheal, bronchus, and lung cancer	Female	30 - 84	Home	Family (anyone in household)	Non-smokers (former and never)	0.000	0.103	0.103	Yes
Bräuner 2012	Prospective cohort	Tracheal, bronchus, and lung cancer	Both	50 - 64	Home or work	Any	Any smoking status	1.769	0.146	0.146	Yes
Cardenas 1997	Prospective cohort	Tracheal, bronchus, and lung cancer	Female	30 - 99	Home	Spouse	Never smokers	0.182	0.207	0.207	No
Cardenas 1997	Prospective cohort	Tracheal, bronchus, and lung cancer	Male	30 - 99	Home	Spouse	Never smokers	0.000	0.354	0.354	No
Cassidy 2006	Case-control	Tracheal, bronchus, and lung cancer	Both	18 - 99	Home	Family (anyone in household)	Any smoking status	0.392	0.133	0.188	No
Cassidy 2006	Case-control	Tracheal, bronchus, and lung cancer	Both	18 - 99	Work	Any	Any smoking status	-0.223	0.120	0.170	Yes
Chan-Yeung 2003	Case-control	Tracheal, bronchus, and lung cancer	Male	18 - 99	Home or work	Any	Never smokers	0.888	0.343	0.343	No
Chan-Yeung 2003	Case-control	Tracheal, bronchus, and lung cancer	Female	18 - 99	Home or work	Any	Never smokers	1.281	0.439	0.439	No

Table S6. Summary of data inputs for tracheal, bronchus, and lung cancer

Author	Study design	Health outcome	Sex	Age	Exposure location	Exposure source	Smoking status	Log effect size	Original SE of the log effect size	SE of the log effect size used in the model	Outlier
Chen 2008	Case-control	Tracheal, bronchus, and lung cancer	Both	18 - 99	Home or work	Any	Any smoking status	0.264	0.217	0.217	No
Cheng 2022	Prospective cohort	Tracheal, bronchus, and lung cancer	Both	30 - 79	Home	Family (anyone in household)	Never smokers	-0.041	0.087	0.087	Yes
Consonni 2018	Case-control	Tracheal, bronchus, and lung cancer	Male	35 - 79	Home or work	Any	Any smoking status	0.519	0.151	0.151	No
Consonni 2018	Case-control	Tracheal, bronchus, and lung cancer	Female	35 - 79	Home or work	Any	Any smoking status	0.255	0.310	0.310	No
Dalager 1986	Case-control	Tracheal, bronchus, and lung cancer	Both	18 - 99	Home	Spouse	Never smokers	0.020	0.576	0.998	No
Dalager 1986	Case-control	Tracheal, bronchus, and lung cancer	Both	18 - 99	Home	Spouse	Never smokers	1.058	0.587	1.017	No
Dalager 1986	Case-control	Tracheal, bronchus, and lung cancer	Both	18 - 99	Home	Spouse	Never smokers	0.270	0.512	0.887	No
Davis 2018	Case-control	Tracheal, bronchus, and lung cancer	Female	20 - 75	Home or work	Any	Never smokers	-0.041	0.288	0.288	No
Du 1995	Case-control	Tracheal, bronchus, and lung cancer	Female	18 - 99	Home	Spouse	Never smokers	0.174	0.302	0.302	No

Table S6. Summary of data inputs for tracheal, bronchus, and lung cancer

Author	Study design	Health outcome	Sex	Age	Exposure location	Exposure source	Smoking status	Log effect size	Original SE of the log effect size	SE of the log effect size used in the model	Outlier
Enstrom 2003	Prospective cohort	Tracheal, bronchus, and lung cancer	Male	35 - 84	Home	Spouse	Never smokers	-0.562	0.403	0.403	No
Enstrom 2003	Prospective cohort	Tracheal, bronchus, and lung cancer	Female	35 - 84	Home	Spouse	Never smokers	-0.128	0.193	0.193	No
Ferreccio 2013	Case-control	Tracheal, bronchus, and lung cancer	Both	30 - 99	Home	Family (anyone in household)	Never smokers	0.358	0.310	0.310	No
Fontham 1994	Case-control	Tracheal, bronchus, and lung cancer	Female	20 - 79	Home	Family (anyone in household)	Never smokers	0.207	0.125	0.177	No
Fontham 1994	Case-control	Tracheal, bronchus, and lung cancer	Female	20 - 79	Work	Any	Never smokers	0.329	0.115	0.162	No
Franco-Marina 2006	Case-control	Tracheal, bronchus, and lung cancer	Both	47 - 102	Home	Family (anyone in household)	Any smoking status	0.588	0.187	0.187	No
Galeone 2008	Case-control	Tracheal, bronchus, and lung cancer	Both	18 - 99	Home and work	Any	Never smokers	0.604	0.347	0.347	No
Gallegos-Arreola 2008	Case-control	Tracheal, bronchus, and lung cancer	Both	18 - 88	Other	Any	Never smokers	2.079	0.752	0.752	Yes
Gao 1987	Case-control	Tracheal, bronchus, and lung cancer	Female	35 - 69	Home	Family (anyone in household)	Any smoking status	-0.105	0.216	0.216	No

Table S6. Summary of data inputs for tracheal, bronchus, and lung cancer

Author	Study design	Health outcome	Sex	Age	Exposure location	Exposure source	Smoking status	Log effect size	Original SE of the log effect size	SE of the log effect size used in the model	Outlier
Garfinkel 1985	Case-control	Tracheal, bronchus, and lung cancer	Female	40 - 99	Home	Spouse	Never smokers	0.207	0.136	0.136	No
Ger 1993	Case-control	Tracheal, bronchus, and lung cancer	Both	18 - 99	Home	Spouse	Any smoking status	-0.261	0.400	0.980	No
Ger 1993	Case-control	Tracheal, bronchus, and lung cancer	Both	18 - 99	Home	Other family (not spouse)	Any smoking status	-0.416	0.336	0.823	No
Ger 1993	Case-control	Tracheal, bronchus, and lung cancer	Both	18 - 99	Work	Any	Any smoking status	0.293	0.319	0.780	No
Ger 1993	Case-control	Tracheal, bronchus, and lung cancer	Both	18 - 99	Home	Spouse	Any smoking status	1.044	0.513	1.257	No
Ger 1993	Case-control	Tracheal, bronchus, and lung cancer	Both	18 - 99	Home	Other family (not spouse)	Any smoking status	-0.041	0.352	0.863	No
Ger 1993	Case-control	Tracheal, bronchus, and lung cancer	Both	18 - 99	Work	Any	Any smoking status	0.554	0.386	0.945	No
Gorlova 2008	Case-control	Tracheal, bronchus, and lung cancer	Both	18 - 99	Home or work	Any	Never smokers	0.610	0.193	0.193	No
Han 2017	Case-control	Tracheal, bronchus, and lung cancer	Both	19 - 99	Home	Family (anyone in household)	Never smokers	0.507	0.307	0.614	No

Table S6. Summary of data inputs for tracheal, bronchus, and lung cancer

Author	Study design	Health outcome	Sex	Age	Exposure location	Exposure source	Smoking status	Log effect size	Original SE of the log effect size	SE of the log effect size used in the model	Outlier
Han 2017	Case-control	Tracheal, bronchus, and lung cancer	Both	19 - 99	Work	Any	Never smokers	1.562	0.382	0.764	No
Han 2017	Case-control	Tracheal, bronchus, and lung cancer	Both	19 - 99	Home	Family (anyone in household)	Never smokers	0.438	0.350	0.699	No
Han 2017	Case-control	Tracheal, bronchus, and lung cancer	Both	19 - 99	Work	Any	Never smokers	0.536	0.357	0.713	No
Hansen 2021	Prospective cohort	Tracheal, bronchus, and lung cancer	Female	31 - 70	Home	Family (anyone in household)	Never smokers	0.293	0.208	0.208	No
He 2012	Prospective cohort	Tracheal, bronchus, and lung cancer	Both	51.5 - 87.8	Home or work	Any	Never smokers	0.693	0.595	0.595	No
He 2013	Case-control	Tracheal, bronchus, and lung cancer	Both	18 - 99	Home or work	Any	Any smoking status	1.795	0.132	0.132	Yes
He 2017	Case-control	Tracheal, bronchus, and lung cancer	Female	40 - 99	Home or work	Any	Any smoking status	0.770	0.132	0.132	Yes
Hernández-Garduño 2004	Case-control	Tracheal, bronchus, and lung cancer	Female	44 - 93	Home	Family (anyone in household)	Never smokers	0.405	0.261	0.261	No
Hill 2007	Prospective cohort	Tracheal, bronchus, and lung cancer	Male	45 - 74	Home	Family (anyone in household)	Never smokers	0.077	0.336	0.336	No

Table S6. Summary of data inputs for tracheal, bronchus, and lung cancer

Author	Study design	Health outcome	Sex	Age	Exposure location	Exposure source	Smoking status	Log effect size	Original SE of the log effect size	SE of the log effect size used in the model	Outlier
Hill 2007	Prospective cohort	Tracheal, bronchus, and lung cancer	Female	45 - 74	Home	Family (anyone in household)	Never smokers	0.322	0.288	0.288	No
Hirayama 1984	Prospective cohort	Tracheal, bronchus, and lung cancer	Female	40 - 99	Home	Spouse	Never smokers	0.372	0.202	0.202	No
Hirayama 1984	Prospective cohort	Tracheal, bronchus, and lung cancer	Male	40 - 99	Home	Spouse	Never smokers	0.811	0.385	0.385	No
Hole 1989	Prospective cohort	Tracheal, bronchus, and lung cancer	Both	45 - 64	Home	Family (anyone in household)	Never smokers	0.880	0.855	0.855	No
Humble 1987	Case-control	Tracheal, bronchus, and lung cancer	Both	18 - 84	Home	Spouse	Never smokers	1.065	0.498	0.498	No
Janerich 1990	Case-control	Tracheal, bronchus, and lung cancer	Both	20 - 80	Home	Spouse	Never smokers	-0.073	0.268	0.268	No
Jee 1999	Prospective cohort	Tracheal, bronchus, and lung cancer	Female	40 - 88	Home	Spouse	Never smokers	0.642	0.320	0.320	No
Jin 2014	Case-control	Tracheal, bronchus, and lung cancer	Both	18 - 99	Home	Family (anyone in household)	Any smoking status	0.344	0.065	0.065	No
Johnson 2001	Case-control	Tracheal, bronchus, and lung cancer	Female	20 - 75	Home and work	Any	Never smokers	0.182	0.457	0.646	No

Table S6. Summary of data inputs for tracheal, bronchus, and lung cancer

Author	Study design	Health outcome	Sex	Age	Exposure location	Exposure source	Smoking status	Log effect size	Original SE of the log effect size	SE of the log effect size used in the model	Outlier
Johnson 2001	Case-control	Tracheal, bronchus, and lung cancer	Female	20 - 75	Home and work	Any	Never smokers	0.489	0.377	0.532	No
Jöckel 1998	Case-control	Tracheal, bronchus, and lung cancer	Both	31 - 42	Home	Spouse	Non-smokers (former and never)	0.457	0.326	0.326	No
Kabat 1984	Case-control	Tracheal, bronchus, and lung cancer	Both	18 - 99	Home	Family (anyone in household)	Never smokers	0.000	0.356	0.503	No
Kabat 1984	Case-control	Tracheal, bronchus, and lung cancer	Both	18 - 99	Work	Any	Never smokers	0.104	0.322	0.455	No
Kabat 1995	Case-control	Tracheal, bronchus, and lung cancer	Male	20 - 80	Home	Spouse	Never smokers	0.470	0.444	0.444	No
Kabat 1995	Case-control	Tracheal, bronchus, and lung cancer	Female	20 - 80	Home	Spouse	Never smokers	0.077	0.299	0.299	No
Kabat 1996	Case-control	Tracheal, bronchus, and lung cancer	Male	20 - 80	Home	Spouse	Never smokers	0.470	0.432	0.432	No
Kabat 1996	Case-control	Tracheal, bronchus, and lung cancer	Female	20 - 80	Home	Spouse	Never smokers	0.095	0.294	0.294	No
Kalandidi 1990	Case-control	Tracheal, bronchus, and lung cancer	Female	35 - 99	Home	Spouse	Never smokers	0.747	0.337	0.337	No

Table S6. Summary of data inputs for tracheal, bronchus, and lung cancer

Author	Study design	Health outcome	Sex	Age	Exposure location	Exposure source	Smoking status	Log effect size	Original SE of the log effect size	SE of the log effect size used in the model	Outlier
Koo 1987	Case-control	Tracheal, bronchus, and lung cancer	Female	18 - 99	Home	Family (anyone in household)	Never smokers	0.519	0.555	0.784	No
Koo 1987	Case-control	Tracheal, bronchus, and lung cancer	Female	18 - 99	Home	Family (anyone in household)	Never smokers	-0.446	0.594	0.840	No
Kurahashi 2008	Prospective cohort	Tracheal, bronchus, and lung cancer	Female	40 - 69	Home	Spouse	Never smokers	0.293	0.256	0.362	No
Kurahashi 2008	Prospective cohort	Tracheal, bronchus, and lung cancer	Female	40 - 69	Work	Any	Never smokers	1.008	0.461	0.652	No
Lam 1987	Case-control	Tracheal, bronchus, and lung cancer	Female	18 - 99	Home	Spouse	Never smokers	0.501	0.180	0.180	No
Lan 2008	Case-control	Tracheal, bronchus, and lung cancer	Both	18 - 85	Home	Family (anyone in household)	Any smoking status	0.474	0.247	0.247	No
Lee 1986	Case-control	Tracheal, bronchus, and lung cancer	Both	35 - 74	Home	Spouse	Never smokers	0.104	0.394	0.394	No
Lee 2000	Case-control	Tracheal, bronchus, and lung cancer	Female	18 - 99	Home	Spouse	Never smokers	0.788	0.201	0.402	No
Lee 2000	Case-control	Tracheal, bronchus, and lung cancer	Female	18 - 99	Home	Other family (not spouse)	Never smokers	0.182	0.292	0.584	No

Table S6. Summary of data inputs for tracheal, bronchus, and lung cancer

Author	Study design	Health outcome	Sex	Age	Exposure location	Exposure source	Smoking status	Log effect size	Original SE of the log effect size	SE of the log effect size used in the model	Outlier
Lee 2000	Case-control	Tracheal, bronchus, and lung cancer	Female	18 - 99	Home	Other family (not spouse)	Never smokers	0.405	0.239	0.479	No
Lee 2000	Case-control	Tracheal, bronchus, and lung cancer	Female	18 - 99	Work	Any	Never smokers	0.182	0.400	0.800	No
Li 2002	Case-cohort	Tracheal, bronchus, and lung cancer	Both	24 - 78	Work	Any	Never smokers	1.044	0.800	0.800	No
Li 2020	Prospective cohort	Tracheal, bronchus, and lung cancer	Both	23 - 79	Home or work	Any	Never smokers	0.239	0.170	0.170	No
Liang 2009	Case-control	Tracheal, bronchus, and lung cancer	Female	20 - 80	Other	Any	Never smokers	0.049	0.215	0.303	No
Liang 2009	Case-control	Tracheal, bronchus, and lung cancer	Female	20 - 80	Other	Any	Never smokers	0.358	0.186	0.262	No
Liang 2019	Case-control	Tracheal, bronchus, and lung cancer	Both	15 - 99	Work	Any	Never smokers	0.658	0.117	0.165	No
Liang 2019	Case-control	Tracheal, bronchus, and lung cancer	Both	15 - 99	Home	Family (anyone in household)	Never smokers	0.658	0.091	0.128	No
Liu 1991	Case-control	Tracheal, bronchus, and lung cancer	Female	18 - 99	Home	Family (anyone in household)	Any smoking status	-0.261	0.479	0.479	No

Table S6. Summary of data inputs for tracheal, bronchus, and lung cancer

Author	Study design	Health outcome	Sex	Age	Exposure location	Exposure source	Smoking status	Log effect size	Original SE of the log effect size	SE of the log effect size used in the model	Outlier
Masjedi 2013	Case-control	Tracheal, bronchus, and lung cancer	Male	18 - 99	Other	Any	Unknown	0.531	0.463	0.463	No
Masjedi 2013	Case-control	Tracheal, bronchus, and lung cancer	Female	18 - 99	Other	Any	Unknown	0.336	0.363	0.363	No
Mbeje 2022	Case-control	Tracheal, bronchus, and lung cancer	Both	21 - 99	Home or work	Any	Any smoking status	1.188	0.407	0.407	No
McGhee 2005	Case-control	Tracheal, bronchus, and lung cancer	Both	60 - 99	Home	Family (anyone in household)	Never smokers	0.329	0.153	0.153	No
Miller 2003	Case-control	Tracheal, bronchus, and lung cancer	Both	27 - 96	Other	Any	Never smokers	0.322	0.290	0.290	No
Minichilli 2022	Case-control	Tracheal, bronchus, and lung cancer	Both	18 - 99	Other	Any	Any smoking status	0.304	0.112	0.112	No
Ng 2005	Case-control	Tracheal, bronchus, and lung cancer	Female	18 - 99	Home	Family (anyone in household)	Never smokers	-0.288	0.251	0.355	No
Ng 2005	Case-control	Tracheal, bronchus, and lung cancer	Female	18 - 99	Home	Family (anyone in household)	Never smokers	0.223	0.400	0.565	No
Nishino 2001	Prospective cohort	Tracheal, bronchus, and lung cancer	Female	40 - 99	Home	Family (anyone in household)	Never smokers	0.182	0.607	0.607	No

Table S6. Summary of data inputs for tracheal, bronchus, and lung cancer

Author	Study design	Health outcome	Sex	Age	Exposure location	Exposure source	Smoking status	Log effect size	Original SE of the log effect size	SE of the log effect size used in the model	Outlier
Phukan 2014	Case-control	Tracheal, bronchus, and lung cancer	Female	18 - 99	Home	Family (anyone in household)	Any smoking status	0.445	0.217	0.217	No
Pirie 2016	Prospective cohort	Tracheal, bronchus, and lung cancer	Female	50 - 84	Home	Family (anyone in household)	Never smokers	-0.041	0.167	0.167	No
Rapiti 1999	Case-control	Tracheal, bronchus, and lung cancer	Both	18 - 99	Home	Spouse	Never smokers	1.629	0.619	0.619	Yes
Raspanti 2016	Case-control	Tracheal, bronchus, and lung cancer	Both	18 - 99	Home or work	Any	Any smoking status	0.255	0.140	0.140	No
Ren 2013	Case-control	Tracheal, bronchus, and lung cancer	Female	18 - 99	Other	Any	Never smokers	0.122	0.105	0.105	No
Robles 2014	Case-control	Tracheal, bronchus, and lung cancer	Both	18 - 99	Other	Any	Never smokers	0.810	0.164	0.164	Yes
Rylander 2006	Case-control	Tracheal, bronchus, and lung cancer	Both	18 - 75	Work	Any	Non-smokers (former and never)	0.815	0.452	0.452	No
Sasco 2002	Case-control	Tracheal, bronchus, and lung cancer	Both	34 - 82	Home or work	Any	Any smoking status	0.307	0.333	0.333	No
Schwartz 2007	Case-control	Tracheal, bronchus, and lung cancer	Female	18 - 74	Home	Family (anyone in household)	Any smoking status	0.772	0.148	0.210	No

Table S6. Summary of data inputs for tracheal, bronchus, and lung cancer

Author	Study design	Health outcome	Sex	Age	Exposure location	Exposure source	Smoking status	Log effect size	Original SE of the log effect size	SE of the log effect size used in the model	Outlier
Schwartz 2007	Case-control	Tracheal, bronchus, and lung cancer	Female	18 - 74	Work	Any	Any smoking status	0.674	0.146	0.206	No
Seki 2013	Case-control	Tracheal, bronchus, and lung cancer	Male	30 - 99	Home	Spouse	Never smokers	0.255	0.681	0.681	No
Seki 2013	Case-control	Tracheal, bronchus, and lung cancer	Female	30 - 99	Home	Spouse	Never smokers	0.270	0.141	0.141	No
Shen 1998	Case-control	Tracheal, bronchus, and lung cancer	Female	18 - 99	Home	Family (anyone in household)	Never smokers	0.322	0.466	0.466	No
Sloan 2012	Case-control	Tracheal, bronchus, and lung cancer	Both	30 - 74	Home	Family (anyone in household)	Any smoking status	0.723	0.220	0.311	No
Sloan 2012	Case-control	Tracheal, bronchus, and lung cancer	Both	30 - 74	Home	Family (anyone in household)	Any smoking status	0.658	0.192	0.271	No
Sobue 1990	Case-control	Tracheal, bronchus, and lung cancer	Female	40 - 79	Home	Other family (not spouse)	Never smokers	0.405	0.201	0.201	No
Speizer 1999	Prospective cohort	Tracheal, bronchus, and lung cancer	Female	30 - 55	Other	Any	Never smokers	0.405	0.777	0.777	No
Spitz 2011	Case-control	Tracheal, bronchus, and lung cancer	Both	18 - 99	Other	Any	Never smokers	0.464	0.179	0.179	No

Table S6. Summary of data inputs for tracheal, bronchus, and lung cancer

Author	Study design	Health outcome	Sex	Age	Exposure location	Exposure source	Smoking status	Log effect size	Original SE of the log effect size	SE of the log effect size used in the model	Outlier
Stockwell 1992	Case-control	Tracheal, bronchus, and lung cancer	Female	18 - 99	Home	Spouse	Never smokers	0.470	0.337	0.337	No
Sun 2017	Prospective cohort	Tracheal, bronchus, and lung cancer	Both	20 - 99	Home	Family (anyone in household)	Any smoking status	0.405	0.166	0.235	No
Sun 2017	Prospective cohort	Tracheal, bronchus, and lung cancer	Both	20 - 99	Home	Family (anyone in household)	Any smoking status	0.412	0.163	0.230	No
Svensson 1989	Case-control	Tracheal, bronchus, and lung cancer	Female	18 - 99	Home or work	Any	Never smokers	0.182	0.505	0.505	No
Torres-Durán 2015	Case-control	Tracheal, bronchus, and lung cancer	Both	34 - 90	Home	Family (anyone in household)	Never smokers	-0.248	0.209	0.209	Yes
Tubío-Pérez 2022	Case-control	Tracheal, bronchus, and lung cancer	Both	36 - 99	Home	Family (anyone in household)	Never smokers	-0.128	0.146	0.146	Yes
Veglia 2007	Prospective cohort	Tracheal, bronchus, and lung cancer	Both	35 - 74	Work	Any	Unknown	0.464	0.143	0.143	No
Villeneuve 2014	Case-control	Tracheal, bronchus, and lung cancer	Both	20 - 84	Home	Family (anyone in household)	Any smoking status	0.113	0.271	0.470	No
Villeneuve 2014	Case-control	Tracheal, bronchus, and lung cancer	Both	20 - 84	Work	Any	Any smoking status	0.577	0.361	0.625	No

Table S6. Summary of data inputs for tracheal, bronchus, and lung cancer

Author	Study design	Health outcome	Sex	Age	Exposure location	Exposure source	Smoking status	Log effect size	Original SE of the log effect size	SE of the log effect size used in the model	Outlier
Villeneuve 2014	Case-control	Tracheal, bronchus, and lung cancer	Both	20 - 84	Home and work	Any	Any smoking status	0.525	0.267	0.463	No
Wang 1994	Case-control	Tracheal, bronchus, and lung cancer	Female	30 - 69	Home	Family (anyone in household)	Non-smokers (former and never)	-0.094	0.527	0.527	No
Wang 1996	Case-control	Tracheal, bronchus, and lung cancer	Both	32 - 78	Home	Family (anyone in household)	Any smoking status	0.582	0.258	0.365	No
Wang 1996	Case-control	Tracheal, bronchus, and lung cancer	Both	32 - 78	Work	Any	Any smoking status	0.519	0.201	0.284	No
Wang 1996	Case-control	Tracheal, bronchus, and lung cancer	Female	35 - 69	Work	Any	Never smokers	-0.117	0.349	0.349	No
Wang 2009	Case-control	Tracheal, bronchus, and lung cancer	Female	30 - 79	Home or work	Any	Never smokers	-0.010	0.307	0.434	No
Wang 2009	Case-control	Tracheal, bronchus, and lung cancer	Female	30 - 79	Home and work	Any	Never smokers	0.049	0.338	0.478	No
Wang 2015	Prospective cohort	Tracheal, bronchus, and lung cancer	Female	50 - 79	Home	Family (anyone in household)	Never smokers	0.191	0.180	0.254	No
Wang 2015	Prospective cohort	Tracheal, bronchus, and lung cancer	Female	50 - 79	Work	Any	Never smokers	0.039	0.179	0.253	No

Table S6. Summary of data inputs for tracheal, bronchus, and lung cancer

Author	Study design	Health outcome	Sex	Age	Exposure location	Exposure source	Smoking status	Log effect size	Original SE of the log effect size	SE of the log effect size used in the model	Outlier
Weiss 2008	Prospective cohort	Tracheal, bronchus, and lung cancer	Female	40 - 70	Home or work	Family (anyone in household)	Non-smokers (former and never)	-0.062	0.186	0.186	No
Wen 2006	Prospective cohort	Tracheal, bronchus, and lung cancer	Female	40 - 70	Home	Spouse	Never smokers	0.058	0.219	0.219	No
Wenzlaff 2005	Case-control	Tracheal, bronchus, and lung cancer	Both	18 - 99	Home	Family (anyone in household)	Never smokers	-0.110	0.238	0.238	No
Wu 1985	Case-control	Tracheal, bronchus, and lung cancer	Female	18 - 75	Home	Spouse	Any smoking status	0.182	0.364	0.728	No
Wu 1985	Case-control	Tracheal, bronchus, and lung cancer	Female	18 - 75	Home	Spouse	Any smoking status	0.000	1.105	2.210	No
Wu 1985	Case-control	Tracheal, bronchus, and lung cancer	Female	18 - 75	Work	Any	Any smoking status	0.182	0.258	0.516	No
Wu 1985	Case-control	Tracheal, bronchus, and lung cancer	Female	18 - 75	Work	Any	Any smoking status	0.833	0.618	1.236	No
Xu 2020	Case-control	Tracheal, bronchus, and lung cancer	Both	18 - 99	Other	Any	Any smoking status	0.942	0.136	0.136	Yes
Yang 2015	Case-control	Tracheal, bronchus, and lung cancer	Both	18 - 99	Home	Family (anyone in household)	Never smokers	0.300	0.136	0.192	No

Table S6. Summary of data inputs for tracheal, bronchus, and lung cancer

Author	Study design	Health outcome	Sex	Age	Exposure location	Exposure source	Smoking status	Log effect size	Original SE of the log effect size	SE of the log effect size used in the model	Outlier
Yang 2015	Case-control	Tracheal, bronchus, and lung cancer	Both	18 - 99	Home	Family (anyone in household)	Never smokers	0.344	0.163	0.230	No
Yang 2016	Case-control	Tracheal, bronchus, and lung cancer	Male	18 - 99	Other	Any	Any smoking status	0.986	0.206	0.206	Yes
Yang 2016	Case-control	Tracheal, bronchus, and lung cancer	Female	18 - 99	Other	Any	Any smoking status	0.756	0.167	0.167	No
Yin 2014	Case-control	Tracheal, bronchus, and lung cancer	Female	18 - 99	Other	Any	Never smokers	0.247	0.163	0.163	No
Yoon 2008	Case-control	Tracheal, bronchus, and lung cancer	Female	30 - 71	Home	Spouse	Never smokers	-1.323	0.346	0.692	Yes
Yoon 2008	Case-control	Tracheal, bronchus, and lung cancer	Female	30 - 71	Home	Paternal	Never smokers	-0.252	0.225	0.450	No
Yoon 2008	Case-control	Tracheal, bronchus, and lung cancer	Female	30 - 71	Home	Maternal	Never smokers	-0.478	0.274	0.548	No
Yoon 2008	Case-control	Tracheal, bronchus, and lung cancer	Female	30 - 71	Work	Any	Never smokers	0.075	0.194	0.388	No
Zaridze 1998	Case-control	Tracheal, bronchus, and lung cancer	Female	18 - 99	Home	Spouse	Never smokers	0.425	0.187	0.325	No

Table S6. Summary of data inputs for tracheal, bronchus, and lung cancer

Author	Study design	Health outcome	Sex	Age	Exposure location	Exposure source	Smoking status	Log effect size	Original SE of the log effect size	SE of the log effect size used in the model	Outlier
Zaridze 1998	Case-control	Tracheal, bronchus, and lung cancer	Female	18 - 99	Home	Other family (not spouse)	Never smokers	-0.094	0.228	0.396	No
Zaridze 1998	Case-control	Tracheal, bronchus, and lung cancer	Female	18 - 99	Work	Any	Never smokers	-0.128	0.240	0.416	No
Zatloukal 2003	Case-control	Tracheal, bronchus, and lung cancer	Female	25 - 89	Other	Any	Never smokers	-0.844	0.397	0.397	Yes
Zhong 1999	Case-control	Tracheal, bronchus, and lung cancer	Female	35 - 69	Home or work	Any	Never smokers	0.336	0.204	0.288	No
Zhong 1999	Case-control	Tracheal, bronchus, and lung cancer	Female	35 - 69	Home or work	Any	Never smokers	0.182	0.207	0.293	No
Zhuang 2022	Case-control	Tracheal, bronchus, and lung cancer	Both	18 - 99	Other	Any	Non-smokers (former and never)	0.802	0.115	0.115	Yes

Table S7. Summary of data inputs for breast cancer

Author	Study design	Health outcome	Sex	Age	Exposure location	Exposure source	Smoking status	Log effect size	Original SE of the log effect size	SE of the log effect size used in the model	Outlier
Ahern 2009	Case-control	Breast cancer	Female	28 - 75	Home or work	Any	Never smokers	0.000	0.312	0.441	No
Ahern 2009	Case-control	Breast cancer	Female	28 - 75	Home or work	Any	Never smokers	-0.357	0.337	0.477	No
Alberg 2004	Nested case-control	Breast cancer	Female	18 - 99	Home	Spouse	Never smokers	0.182	0.358	0.358	No
Anderson 2012	Case-control	Breast cancer	Female	25 - 74	Home	Family (anyone in household)	Never smokers	0.129	0.093	0.132	No
Anderson 2012	Case-control	Breast cancer	Female	25 - 74	Work	Any	Never smokers	0.023	0.094	0.133	No
Bonner 2005	Case-control	Breast cancer	Female	35 - 79	Home or work	Any	Never smokers	0.104	0.325	0.460	No
Bonner 2005	Case-control	Breast cancer	Female	35 - 79	Home or work	Any	Never smokers	-0.223	0.204	0.288	No
Chaveepojnkamjorn 2017	Case-control	Breast cancer	Female	25 - 44	Other	Any	Any smoking status	0.798	0.213	0.213	Yes
De 2010	Case-control	Breast cancer	Female	30 - 64	Other	Any	Never smokers	1.085	0.338	0.338	No
Dianatinasab 2017	Case-control	Breast cancer	Female	18 - 99	Home	Parental	Any smoking status	0.451	0.162	0.162	No
Dossus 2014	Prospective cohort	Breast cancer	Female	18 - 99	Home or work	Any	Never smokers	0.095	0.044	0.044	No

Table S7. Summary of data inputs for breast cancer

Author	Study design	Health outcome	Sex	Age	Exposure location	Exposure source	Smoking status	Log effect size	Original SE of the log effect size	SE of the log effect size used in the model	Outlier
El-Sheikh 2021	Case-control	Breast cancer	Female	19 - 99	Other	Any	Non-smokers (former and never)	1.636	0.477	0.477	Yes
Fararouei 2019	Case-control	Breast cancer	Female	20 - 50	Other	Any	Any smoking status	0.536	0.146	0.146	No
Fu 2015	Case-control	Breast cancer	Female	18 - 99	Other	Any	Any smoking status	1.573	0.310	0.310	Yes
Gao 2013	Case-control	Breast cancer	Female	18 - 99	Home	Spouse	Any smoking status	0.372	0.119	0.119	No
Hanaoka 2005	Prospective cohort	Breast cancer	Female	40 - 59	Home	Family (anyone in household)	Never smokers	0.000	0.177	0.177	No
Hirose 1995	Case-control	Breast cancer	Female	20 - 99	Home	Spouse	Never smokers	0.140	0.121	0.171	No
Hirose 1995	Case-control	Breast cancer	Female	20 - 99	Home	Spouse	Never smokers	0.329	0.147	0.208	No
Hosseinzadeh 2014	Case-control	Breast cancer	Female	18 - 99	Other	Any	Any smoking status	1.015	0.307	0.307	Yes
Hsieh 2014	Case-control	Breast cancer	Female	18 - 99	Other	Any	Any smoking status	1.224	0.182	0.182	Yes
Hu 2013	Case-control	Breast cancer	Female	25 - 75	Home or work	Any	Any smoking status	0.432	0.252	0.252	No
Ilic 2013	Case-control	Breast cancer	Female	18 - 99	Home or work	Any	Never smokers	0.451	0.337	0.337	No
Jee 1999	Prospective cohort	Breast cancer	Female	40 - 88	Home	Spouse	Never smokers	0.262	0.177	0.177	No

Table S7. Summary of data inputs for breast cancer

Author	Study design	Health outcome	Sex	Age	Exposure location	Exposure source	Smoking status	Log effect size	Original SE of the log effect size	SE of the log effect size used in the model	Outlier
Johnson 2000	Case-control	Breast cancer	Female	25 - 74	Home or work	Any	Never smokers	0.956	0.433	0.866	No
Johnson 2000	Case-control	Breast cancer	Female	25 - 74	Home or work	Any	Never smokers	0.956	0.388	0.777	No
Johnson 2000	Case-control	Breast cancer	Female	25 - 74	Home or work	Any	Former smokers	0.470	0.496	0.993	No
Johnson 2000	Case-control	Breast cancer	Female	25 - 74	Home or work	Any	Former smokers	0.956	0.359	0.717	No
Johnson 2000	Case-control	Breast cancer	Female	25 - 74	Home or work	Any	Never smokers	0.095	0.280	0.561	No
Johnson 2000	Case-control	Breast cancer	Female	25 - 74	Home or work	Any	Never smokers	0.262	0.234	0.467	No
Johnson 2000	Case-control	Breast cancer	Female	25 - 74	Home or work	Any	Former smokers	0.588	0.312	0.624	No
Johnson 2000	Case-control	Breast cancer	Female	25 - 74	Home or work	Any	Former smokers	0.262	0.234	0.467	No
Kariri 2017	Case-control	Breast cancer	Female	18 - 60	Other	Any	Unknown	0.435	0.241	0.241	No
Lash 1999	Case-control	Breast cancer	Female	18 - 99	Home	Family (anyone in household)	Never smokers	0.693	0.309	0.309	No
Lash 2002	Case-control	Breast cancer	Female	18 - 99	Home	Family (anyone in household)	Never smokers	-0.163	0.142	0.142	No
Li 2015	Case-control	Breast cancer	Female	25 - 70	Home or work	Any	Never smokers	0.300	0.101	0.101	No

Table S7. Summary of data inputs for breast cancer

Author	Study design	Health outcome	Sex	Age	Exposure location	Exposure source	Smoking status	Log effect size	Original SE of the log effect size	SE of the log effect size used in the model	Outlier
Lin 2008	Prospective cohort	Breast cancer	Female	40 - 79	Home	Family (anyone in household)	Never smokers	-0.528	0.295	0.418	No
Lin 2008	Prospective cohort	Breast cancer	Female	40 - 79	Home	Family (anyone in household)	Never smokers	-0.342	0.200	0.282	No
Liu 2000	Case-control	Breast cancer	Female	24 - 55	Home	Family (anyone in household)	Never smokers	1.404	0.312	0.441	Yes
Liu 2000	Case-control	Breast cancer	Female	24 - 55	Work	Any	Never smokers	0.239	0.102	0.144	No
Luo 2011	Prospective cohort	Breast cancer	Female	50 - 79	Home and work	Any	Never smokers	0.140	0.106	0.184	No
Luo 2011	Prospective cohort	Breast cancer	Female	50 - 79	Home	Family (anyone in household)	Never smokers	-0.094	0.135	0.234	No
Luo 2011	Prospective cohort	Breast cancer	Female	50 - 79	Work	Any	Never smokers	0.010	0.110	0.190	No
Marzouk 2009	Case-control	Breast cancer	Female	18 - 59	Home	Family (anyone in household)	Any smoking status	0.642	0.234	0.234	No
Metsola 2005	Case-control	Breast cancer	Female	37.5 - 91.6	Home or work	Any	Never smokers	-0.248	0.154	0.154	No
Millikan 1998	Case-control	Breast cancer	Female	20 - 74	Home	Family (anyone in household)	Never smokers	0.262	0.191	0.191	No

Table S7. Summary of data inputs for breast cancer

Author	Study design	Health outcome	Sex	Age	Exposure location	Exposure source	Smoking status	Log effect size	Original SE of the log effect size	SE of the log effect size used in the model	Outlier
Morabia 1996	Case-control	Breast cancer	Female	30 - 74	Home	Spouse	Never smokers	1.131	0.341	0.341	Yes
Niehoff 2017	Case-control	Breast cancer	Female	20 - 98	Home	Spouse	Any smoking status	0.278	0.098	0.098	No
Nishino 2001	Prospective cohort	Breast cancer	Female	40 - 99	Home	Family (anyone in household)	Never smokers	-0.562	0.381	0.381	No
Nishino 2014	Case-control	Breast cancer	Female	30 - 99	Home	Spouse	Never smokers	0.122	0.131	0.262	No
Nishino 2014	Case-control	Breast cancer	Female	30 - 99	Home	Spouse	Never smokers	-0.010	0.252	0.504	No
Nishino 2014	Case-control	Breast cancer	Female	30 - 99	Home	Spouse	Never smokers	0.542	0.572	1.144	No
Nishino 2014	Case-control	Breast cancer	Female	30 - 99	Home	Spouse	Never smokers	0.157	0.172	0.344	No
Pimhanam 2014	Case-control	Breast cancer	Female	17 - 76	Other	Any	Never smokers	0.820	0.285	0.285	No
Pirie 2008	Prospective cohort	Breast cancer	Female	50 - 64	Home	Family (anyone in household)	Never smokers	0.030	0.071	0.101	No
Pirie 2008	Prospective cohort	Breast cancer	Female	50 - 64	Home	Spouse	Never smokers	0.020	0.068	0.096	No
Reynolds 2009	Prospective cohort	Breast cancer	Female	20 - 99	Home	Family (anyone in household)	Never smokers	0.039	0.059	0.084	No

Table S7. Summary of data inputs for breast cancer

Author	Study design	Health outcome	Sex	Age	Exposure location	Exposure source	Smoking status	Log effect size	Original SE of the log effect size	SE of the log effect size used in the model	Outlier
Reynolds 2009	Prospective cohort	Breast cancer	Female	20 - 99	Work	Any	Never smokers	0.020	0.050	0.070	No
Roddam 2007	Case-control	Breast cancer	Female	36 - 45	Home	Spouse	Never smokers	-0.117	0.171	0.171	No
Roddam 2007	Case-control	Breast cancer	Female	36 - 45	Home	Spouse	Former smokers	0.086	0.187	0.187	No
Rollison 2008	Case-control	Breast cancer	Female	40 - 79	Work	Any	Never smokers	-0.223	0.253	0.358	No
Rollison 2008	Case-control	Breast cancer	Female	40 - 79	Home	Family (anyone in household)	Never smokers	0.058	0.327	0.463	No
Shrubsole 2004	Case-control	Breast cancer	Female	25 - 64	Home	Spouse	Never smokers	0.000	0.103	0.103	No
Smith 1994	Case-control	Breast cancer	Female	18 - 35	Home or work	Any	Never smokers	1.141	0.741	0.741	No
Strumylaite 2017	Case-control	Breast cancer	Female	28 - 90	Work	Any	Never smokers	0.010	0.171	0.297	No
Strumylaite 2017	Case-control	Breast cancer	Female	28 - 90	Home	Family (anyone in household)	Never smokers	0.631	0.157	0.271	No
Strumylaite 2017	Case-control	Breast cancer	Female	28 - 90	Home and work	Any	Never smokers	1.030	0.214	0.370	No
Tang 2013	Case-control	Breast cancer	Female	18 - 99	Home or work	Any	Never smokers	0.385	0.112	0.112	No
Tong 2014	Case-control	Breast cancer	Female	18 - 99	Home	Spouse	Never smokers	0.378	0.168	0.168	No

Table S7. Summary of data inputs for breast cancer

Author	Study design	Health outcome	Sex	Age	Exposure location	Exposure source	Smoking status	Log effect size	Original SE of the log effect size	SE of the log effect size used in the model	Outlier
Wartenberg 2000	Prospective cohort	Breast cancer	Female	30 - 99	Home	Spouse	Never smokers	0.000	0.103	0.103	No
White 2017	Prospective cohort	Breast cancer	Female	35 - 74	Other	Any	Never smokers	-0.151	0.077	0.077	Yes
Woo 2000	Nested case-control	Breast cancer	Female	18 - 99	Home	Family (anyone in household)	Never smokers	0.039	0.120	0.120	No
Xue 2011	Prospective cohort	Breast cancer	Female	30 - 55	Home or work	Any	Never smokers	0.086	0.058	0.117	No
Xue 2011	Prospective cohort	Breast cancer	Female	30 - 55	Home or work	Any	Never smokers	0.000	0.076	0.153	No
Xue 2011	Prospective cohort	Breast cancer	Female	30 - 55	Home or work	Any	Never smokers	-0.010	0.079	0.159	No
Xue 2011	Prospective cohort	Breast cancer	Female	30 - 55	Home or work	Any	Never smokers	-0.030	0.092	0.183	No
Yassin 2018	Case-control	Breast cancer	Female	18 - 99	Other	Any	Any smoking status	0.470	0.234	0.234	No
Zahali 2021	Case-control	Breast cancer	Female	21 - 59	Home	Family (anyone in household)	Never smokers	0.775	0.323	0.457	No
Zahali 2021	Case-control	Breast cancer	Female	21 - 59	Work	Any	Never smokers	-0.062	0.516	0.730	No
Zhao 1999	Case-control	Breast cancer	Female	26 - 82	Other	Any	Unknown	0.793	0.222	0.222	No

Table S8. Summary of data inputs for asthma

Author	Study design	Health outcome	Sex	Age	Exposure location	Exposure source	Smoking status	Log effect size	Original SE of the log effect size	SE of the log effect size used in the model	Outlier
Al-Kubaisy 2005	Prospective cohort	Asthma	Both	6 - 12	Home	Maternal	Adj never smokers	0.747	0.314	0.444	No
Al-Kubaisy 2005	Prospective cohort	Asthma	Both	6 - 12	Home	Paternal	Adj never smokers	0.698	0.106	0.150	Yes
Al-Qerem 2016	Prospective cohort	Asthma	Both	7 - 12	Home	Family (anyone in household)	Never smokers	0.854	0.180	0.180	Yes
Arif 2017	Case-control	Asthma	Both	2 - 14	Home	Parental	Never smokers	0.243	0.285	0.285	No
Aversa 2021	Prospective cohort	Asthma	Both	0 - 0	Home	Maternal	Never smokers	0.329	0.082	0.082	No
Azizi 1995	Case-control	Asthma	Both	0.083 - 5	Home	Family (anyone in household)	Never smokers	0.647	0.266	0.266	No
Balemans 2006	Prospective cohort	Asthma	Both	2 - 21	Home	Maternal	Any smoking status	0.470	0.244	0.244	No
Beijsterveldt 2008	Prospective cohort	Asthma	Both	0.25 - 0.999	Home	Paternal	Never smokers	0.077	0.059	0.059	No
Bener 2007	Case-control	Asthma	Both	6 - 13	Home	Paternal	Adj never smokers	0.928	0.137	0.137	Yes
Bergmann 2000	Prospective cohort	Asthma	Both	0 - 0	Home	Family (anyone in household)	Never smokers	0.276	0.204	0.204	No
Boker 2019	Case-control	Asthma	Both	2 - 14	Home	Parental	Never smokers	-0.288	0.226	0.226	No

Table S8. Summary of data inputs for asthma

Author	Study design	Health outcome	Sex	Age	Exposure location	Exposure source	Smoking status	Log effect size	Original SE of the log effect size	SE of the log effect size used in the model	Outlier
Boneberger 2011	Case-control	Asthma	Both	6 - 15	Home	Family (anyone in household)	Never smokers	0.086	0.225	0.225	No
Bozicević 2000	Case-control	Asthma	Male	18 - 73	Home	Family (anyone in household)	Any smoking status	0.182	0.419	0.593	No
Bozicević 2000	Case-control	Asthma	Female	18 - 73	Home	Family (anyone in household)	Any smoking status	0.713	0.374	0.528	No
Bozicević 2000	Case-control	Asthma	Male	18 - 73	Work	Any	Any smoking status	1.004	0.486	0.688	No
Bozicević 2000	Case-control	Asthma	Female	18 - 73	Work	Any	Any smoking status	1.256	0.382	0.541	No
Butland 1997	Case-control	Asthma	Both	7.5 - 8.5	Home	Paternal	Never smokers	0.039	0.182	0.182	No
Carlsten 2012	Prospective cohort	Asthma	Both	0 - 0	Home	Family (anyone in household)	Never smokers	0.531	0.397	0.397	No
Carr 2019	Prospective cohort	Asthma	Both	0 - 0	Home	Maternal	Never smokers	0.405	0.261	0.261	No
Carrasco 2021	Prospective cohort	Asthma	Both	0 - 0	Home	Family (anyone in household)	Never smokers	0.385	0.123	0.123	No
Chan 2019	Case-crossover	Asthma	Both	18 - 73	Home and work	Any	Never smokers	0.806	0.167	0.167	Yes
Charoenca 2013	Case-control	Asthma	Both	0 - 4	Home	Any	Never smokers	1.340	0.222	0.222	Yes

Table S8. Summary of data inputs for asthma

Author	Study design	Health outcome	Sex	Age	Exposure location	Exposure source	Smoking status	Log effect size	Original SE of the log effect size	SE of the log effect size used in the model	Outlier
Chen 2011	Nested case-control	Asthma	Both	12 - 14	Home	Family (anyone in household)	Non-smokers (former and never)	0.020	0.203	0.203	No
Clark 1994	Case-control	Asthma	Both	5 - 7	Home	Family (anyone in household)	Never smokers	-0.348	0.584	0.584	No
Coogan 2015	Prospective cohort	Asthma	Female	21 - 69	Home or work	Any	Never smokers	0.157	0.111	0.111	No
Daigler 1991	Case-control	Asthma	Both	0 - 15	Home	Maternal	Never smokers	0.673	0.293	0.293	No
David 2005	Prospective cohort	Asthma	Both	45 - 74	Home	Any	Never smokers	0.148	0.087	0.087	No
Dekker 2015	Prospective cohort	Asthma	Both	0 - 0	Home	Family (anyone in household)	Never smokers	0.058	0.258	0.258	No
Ehrlich 1992	Case-control	Asthma	Both	3 - 14	Home	Maternal	Adj never smokers	0.693	0.288	0.288	No
Ehrlich 1996	Case-control	Asthma	Both	7 - 8	Home	Maternal	Never smokers	0.285	0.218	0.218	No
El-Sharif 2003	Case-control	Asthma	Both	6 - 12	Home	Family (anyone in household)	Never smokers	0.269	0.280	0.280	No
Elder 1996	Prospective cohort	Asthma	Both	0 - 0	Home	Maternal	Never smokers	0.322	0.441	0.441	No
Fagbule 1994	Case-control	Asthma	Both	0 - 15	Home	Parental	Never smokers	0.344	0.264	0.264	No

Table S8. Summary of data inputs for asthma

Author	Study design	Health outcome	Sex	Age	Exposure location	Exposure source	Smoking status	Log effect size	Original SE of the log effect size	SE of the log effect size used in the model	Outlier
Fernando 2009	Case-control	Asthma	Both	5 - 12	Home	Family (anyone in household)	Never smokers	1.605	0.445	0.445	Yes
Flexeder 2019	Prospective cohort	Asthma	Both	20 - 44	Other	Any	Non-smokers (former and never)	0.993	0.406	0.406	No
Flodin 1995	Case-control	Asthma	Both	20 - 65	Work	Any	Any smoking status	0.405	0.291	0.291	No
Frassanito 2022	Case-control	Asthma	Both	6 - 6	Other	Any	Adj never smokers	-0.223	0.384	0.384	No
Galobardes 2015	Prospective cohort	Asthma	Both	0 - 0	Home	Family (anyone in household)	Never smokers	0.285	0.117	0.117	No
Genuneit 2006	Prospective cohort	Asthma	Both	9 - 11	Home	Family (anyone in household)	Non-smokers (former and never)	0.693	0.443	0.443	No
Goksör 2006	Prospective cohort	Asthma	Both	0 - 1	Other	Any	Never smokers	1.131	0.566	0.566	No
Grabenhenrich 2014	Prospective cohort	Asthma	Both	0 - 0	Home	Family (anyone in household)	Never smokers	-0.400	0.247	0.247	No
Guo 2019	Case-control	Asthma	Both	0 - 9	Home	Family (anyone in household)	Never smokers	-0.300	0.381	0.381	No
Hadnadjev 2011	Case-control	Asthma	Both	7 - 14	Home	Any	Adj never smokers	-0.236	0.200	0.200	No

Table S8. Summary of data inputs for asthma

Author	Study design	Health outcome	Sex	Age	Exposure location	Exposure source	Smoking status	Log effect size	Original SE of the log effect size	SE of the log effect size used in the model	Outlier
Hagendorens 2005	Prospective cohort	Asthma	Both	0 - 0	Other	Any	Never smokers	1.197	0.312	0.312	Yes
Hedman 2011	Prospective cohort	Asthma	Both	7 - 8	Home	Maternal	Non-smokers (former and never)	0.351	0.142	0.142	No
Horwood 1985	Prospective cohort	Asthma	Both	0 - 0	Home	Parental	Never smokers	-0.151	0.237	0.237	No
Huang 2022	Case-control	Asthma	Both	3 - 18	Home	Family (anyone in household)	Never smokers	0.207	0.379	0.379	No
Hunt 2011	Prospective cohort	Asthma	Both	0 - 0	Home	Family (anyone in household)	Never smokers	0.489	0.451	0.451	No
Hwang 2011	Case-control	Asthma	Both	1 - 7	Home	Family (anyone in household)	Never smokers	0.000	0.182	0.182	No
Håberg 2007	Prospective cohort	Asthma	Both	0 - 0	Home	Maternal	Never smokers	-0.083	0.069	0.098	No
Håberg 2007	Prospective cohort	Asthma	Both	0 - 0	Home	Paternal	Never smokers	0.174	0.049	0.070	No
Izuhara 2016	Prospective cohort	Asthma	Both	30 - 75	Other	Any	Unknown	0.293	0.129	0.129	No
Jaakkola 2001	Prospective cohort	Asthma	Both	0 - 0	Home	Family (anyone in household)	Never smokers	0.095	0.169	0.169	No
Jaakkola 2003	Case-control	Asthma	Both	21 - 63	Home and work	Any	Never smokers	0.507	0.263	0.456	No

Table S8. Summary of data inputs for asthma

Author	Study design	Health outcome	Sex	Age	Exposure location	Exposure source	Smoking status	Log effect size	Original SE of the log effect size	SE of the log effect size used in the model	Outlier
Jaakkola 2003	Case-control	Asthma	Both	21 - 63	Work	Any	Never smokers	0.604	0.285	0.494	No
Jaakkola 2003	Case-control	Asthma	Both	21 - 63	Home	Any	Never smokers	1.343	0.690	1.195	No
Jedrychowski 2008	Prospective cohort	Asthma	Both	0 - 0	Home	Family (anyone in household)	Never smokers	-0.094	0.555	0.961	No
Jedrychowski 2008	Prospective cohort	Asthma	Both	0 - 0	Home	Family (anyone in household)	Never smokers	0.220	0.566	0.981	No
Jedrychowski 2008	Prospective cohort	Asthma	Both	0 - 0	Home	Family (anyone in household)	Never smokers	-0.216	0.653	1.131	No
Kamran 2015	Case-control	Asthma	Both	1 - 15	Home	Parental	Unknown	1.504	0.274	0.274	Yes
Kanoh 2012	Prospective cohort	Asthma	Both	0.5 - 0.5	Home	Maternal	Never smokers	0.215	0.056	0.079	No
Kanoh 2012	Prospective cohort	Asthma	Both	0.5 - 0.5	Home	Paternal	Never smokers	0.039	0.042	0.059	No
Karunasekera 2001	Case-control	Asthma	Both	1 - 10	Other	Any	Adj never smokers	0.095	0.177	0.177	No
Khozime 2019	Case-control	Asthma	Both	5 - 70	Home	Family (anyone in household)	Non-smokers (former and never)	1.728	0.443	0.443	Yes
Kim 2018	Case-cohort	Asthma	Both	5.68 - 12.16	Other	Any	Never smokers	0.491	0.118	0.118	No

Table S8. Summary of data inputs for asthma

Author	Study design	Health outcome	Sex	Age	Exposure location	Exposure source	Smoking status	Log effect size	Original SE of the log effect size	SE of the log effect size used in the model	Outlier
Klinnert 2001	Prospective cohort	Asthma	Both	0 - 0	Home	Maternal	Never smokers	0.432	0.469	0.469	No
Kobyletzki 2012	Prospective cohort	Asthma	Both	1 - 2	Home	Parental	Never smokers	-0.083	0.341	0.341	No
Kumar 2021	Case-control	Asthma	Both	6 - 12	Home	Maternal	Adj never smokers	1.637	0.540	0.540	No
Kurukulaaratchy 2006	Prospective cohort	Asthma	Both	0 - 0	Home	Parental	Never smokers	0.751	0.335	0.474	No
Kurukulaaratchy 2006	Prospective cohort	Asthma	Both	0 - 0	Home	Parental	Never smokers	1.082	0.531	0.751	No
Lawson 2014	Prospective cohort	Asthma	Both	12 - 18	Home	Family (anyone in household)	Never smokers	0.723	0.247	0.247	No
Leen 1994	Case-control	Asthma	Both	0 - 15	Home	Family (anyone in household)	Unknown	-0.280	0.279	0.279	No
Lemanske 2005	Prospective cohort	Asthma	Both	0 - 0	Other	Any	Never smokers	0.262	0.406	0.406	No
Li 2013	Prospective cohort	Asthma	Both	2 - 11	Home	Maternal	Never smokers	0.425	0.017	0.017	Yes
Lilljeqvist 1997	Case-control	Asthma	Both	7 - 10	Home	Parental	Never smokers	0.085	0.154	0.154	No
Litonjua 2001	Prospective cohort	Asthma	Both	0 - 0.00548	Home	Family (anyone in household)	Never smokers	1.292	0.707	0.707	No

Table S8. Summary of data inputs for asthma

Author	Study design	Health outcome	Sex	Age	Exposure location	Exposure source	Smoking status	Log effect size	Original SE of the log effect size	SE of the log effect size used in the model	Outlier
Majeed 2008	Case-control	Asthma	Both	1 - 8	Home	Family (anyone in household)	Adj never smokers	0.788	0.348	0.348	No
Marbury 1996	Prospective cohort	Asthma	Both	0 - 0.167	Home	Maternal	Never smokers	0.742	0.177	0.177	Yes
McConnell 2002	Prospective cohort	Asthma	Both	9 - 16	Home	Maternal	Any smoking status	-0.105	0.280	0.280	No
McKeever 2001	Prospective cohort	Asthma	Both	0 - 0	Home	Maternal	Adj never smokers	0.307	0.034	0.048	No
McKeever 2001	Prospective cohort	Asthma	Both	0 - 0	Home	Paternal	Adj never smokers	0.293	0.042	0.059	No
Melsom 2001	Nested case-control	Asthma	Both	11 - 17	Home	Family (anyone in household)	Unknown	0.642	0.347	0.347	No
Midodzi 2010	Prospective cohort	Asthma	Both	0 - 1	Home	Parental	Never smokers	0.199	0.058	0.058	No
Milanzi 2017	Prospective cohort	Asthma	Both	0 - 0	Other	Any	Never smokers	0.000	0.211	0.211	No
Milner 2004	Prospective cohort	Asthma	Both	0 - 0	Home	Family (anyone in household)	Never smokers	0.438	0.077	0.077	Yes
Mommers 2005	Nested case-control	Asthma	Both	7 - 8	Other	Any	Adj never smokers	0.166	0.229	0.229	No
Morfín-Maciel 2006	Case-control	Asthma	Both	13 - 14	Home	Maternal	Any smoking status	0.884	0.212	0.212	Yes

Table S8. Summary of data inputs for asthma

Author	Study design	Health outcome	Sex	Age	Exposure location	Exposure source	Smoking status	Log effect size	Original SE of the log effect size	SE of the log effect size used in the model	Outlier
Morfín-Maciel 2006	Case-control	Asthma	Both	13 - 14	Home	Family (anyone in household)	Adj never smokers	0.519	0.181	0.181	No
Mpairwe 2019	Case-control	Asthma	Both	5 - 17	Home	Family (anyone in household)	Never smokers	0.231	0.171	0.171	No
Mumcuoglu 1994	Case-control	Asthma	Both	3 - 15	Home	Paternal	Unknown	-0.056	0.235	0.332	No
Mumcuoglu 1994	Case-control	Asthma	Both	3 - 15	Home	Other family (not spouse)	Unknown	0.289	0.393	0.556	No
Murray 2004	Prospective cohort	Asthma	Both	0 - 3	Home	Maternal	Never smokers	0.582	0.275	0.275	No
Muñoz 2012	Case-control	Asthma	Both	6 - 9	Home	Family (anyone in household)	Never smokers	-0.004	0.425	0.425	No
Neuman 2012	Prospective cohort	Asthma	Both	0 - 0	Home	Maternal	Never smokers	-0.288	0.229	0.459	No
Neuman 2012	Prospective cohort	Asthma	Both	0 - 0	Home	Maternal	Never smokers	0.223	1.084	2.168	No
Neuman 2012	Prospective cohort	Asthma	Both	0.083 - 0.167	Home	Maternal	Never smokers	0.372	1.092	1.544	No
Neuman 2012	Prospective cohort	Asthma	Both	0 - 0	Home	Maternal	Never smokers	0.378	0.405	0.810	No
Neuman 2012	Prospective cohort	Asthma	Both	0.00822 - 0.011	Home	Maternal	Never smokers	0.718	0.549	0.549	No

Table S8. Summary of data inputs for asthma

Author	Study design	Health outcome	Sex	Age	Exposure location	Exposure source	Smoking status	Log effect size	Original SE of the log effect size	SE of the log effect size used in the model	Outlier
Neuman 2012	Prospective cohort	Asthma	Both	0.083 - 0.167	Home	Maternal	Never smokers	-0.174	0.659	0.931	No
Neuman 2012	Prospective cohort	Asthma	Both	0 - 0	Home	Maternal	Never smokers	0.507	0.536	1.072	No
Nguyen 2010	Nested case-control	Asthma	Both	0 - 17	Home	Family (anyone in household)	Unknown	-0.357	0.201	0.201	No
Nguyen 2010	Nested case-control	Asthma	Both	18 - 99	Home	Family (anyone in household)	Unknown	-0.105	0.115	0.115	No
Norbäck 2018	Retrospectiv e cohort	Asthma	Both	0 - 0	Home	Family (anyone in household)	Never smokers	0.030	0.052	0.052	No
O'Connell 1974	Case-control	Asthma	Both	2 - 16	Home	Parental	Unknown	0.265	0.172	0.172	No
Oddy 1999	Prospective cohort	Asthma	Both	0 - 0	Home	Family (anyone in household)	Never smokers	0.239	0.102	0.102	No
Palvo 2008	Case-control	Asthma	Both	6 - 7	Home	Family (anyone in household)	Never smokers	0.358	0.125	0.125	No
Patrick 2020	Prospective cohort	Asthma	Both	0 - 0	Home	Family (anyone in household)	Never smokers	0.207	0.224	0.224	No
Pattemore 2018	Prospective cohort	Asthma	Both	0 - 0	Home	Maternal	Never smokers	0.698	0.609	1.492	No

Table S8. Summary of data inputs for asthma

Author	Study design	Health outcome	Sex	Age	Exposure location	Exposure source	Smoking status	Log effect size	Original SE of the log effect size	SE of the log effect size used in the model	Outlier
Pattemore 2018	Prospective cohort	Asthma	Both	0 - 0	Home	Maternal	Never smokers	0.451	0.535	1.311	No
Pattemore 2018	Prospective cohort	Asthma	Both	0 - 0	Home	Maternal	Never smokers	0.412	0.507	1.241	No
Pattemore 2018	Prospective cohort	Asthma	Both	0 - 0	Home	Maternal	Never smokers	-0.105	0.500	1.224	No
Pattemore 2018	Prospective cohort	Asthma	Both	0 - 0	Home	Maternal	Never smokers	-0.635	0.502	1.229	No
Pattemore 2018	Prospective cohort	Asthma	Both	0 - 0	Home	Maternal	Never smokers	-0.041	0.515	1.263	No
Pokharel 2001	Case-control	Asthma	Both	11 - 15	Home	Family (anyone in household)	Unknown	1.203	0.362	0.362	Yes
Pokharel 2007	Case-control	Asthma	Both	11 - 15	Home	Family (anyone in household)	Unknown	1.203	0.359	0.359	Yes
Polk 2004	Prospective cohort	Asthma	Both	0 - 4	Home	Maternal	Never smokers	0.157	0.061	0.122	No
Polk 2004	Prospective cohort	Asthma	Both	0 - 4	Home	Maternal	Never smokers	0.307	0.062	0.123	No
Polk 2004	Prospective cohort	Asthma	Both	0 - 4	Home	Maternal	Never smokers	0.300	0.064	0.128	No
Polk 2004	Prospective cohort	Asthma	Both	0 - 4	Home	Maternal	Never smokers	0.215	0.070	0.140	No
Polosa 2005	Retrospective cohort	Asthma	Both	18 - 40	Home	Parental	Unknown	0.278	0.206	0.206	No

Table S8. Summary of data inputs for asthma

Author	Study design	Health outcome	Sex	Age	Exposure location	Exposure source	Smoking status	Log effect size	Original SE of the log effect size	SE of the log effect size used in the model	Outlier
Ponsonby 2000	Prospective cohort	Asthma	Both	0 - 0	Home	Family (anyone in household)	Never smokers	0.030	0.106	0.106	No
Ratageri 2000	Case-control	Asthma	Both	5 - 15	Home	Family (anyone in household)	Any smoking status	-0.128	0.364	0.515	No
Ratageri 2000	Case-control	Asthma	Both	5 - 15	Home	Family (anyone in household)	Any smoking status	0.270	0.365	0.517	No
Rennie 2008	Case-control	Asthma	Both	6 - 13	Home	Any	Adj never smokers	0.191	0.355	0.355	No
Rosa 2011	Prospective cohort	Asthma	Both	0 - 0	Home	Family (anyone in household)	Never smokers	-0.020	0.318	0.318	No
Schroer 2009	Prospective cohort	Asthma	Both	0 - 0	Home	Family (anyone in household)	Never smokers	0.548	0.227	0.321	No
Schroer 2009	Prospective cohort	Asthma	Both	0 - 0	Home	Family (anyone in household)	Never smokers	0.642	0.264	0.373	No
Sears 2002	Prospective cohort	Asthma	Both	0 - 0	Home	Paternal	Adj never smokers	0.470	0.278	0.278	No
Selby 2018	Prospective cohort	Asthma	Both	0 - 0	Home	Maternal	Never smokers	0.482	0.203	0.288	No
Selby 2018	Prospective cohort	Asthma	Both	0 - 0	Home	Other family (not spouse)	Never smokers	0.223	0.198	0.281	No

Table S8. Summary of data inputs for asthma

Author	Study design	Health outcome	Sex	Age	Exposure location	Exposure source	Smoking status	Log effect size	Original SE of the log effect size	SE of the log effect size used in the model	Outlier
Sherman 1990	Prospective cohort	Asthma	Both	5 - 9	Home	Maternal	Adj never smokers	0.166	0.224	0.317	No
Sherman 1990	Prospective cohort	Asthma	Both	5 - 9	Home	Paternal	Adj never smokers	0.131	0.302	0.427	No
Slob 2020	Retrospective cohort	Asthma	Both	3 - 3	Home	Maternal	Never smokers	0.117	0.030	0.042	No
Slob 2020	Retrospective cohort	Asthma	Both	3 - 3	Home	Paternal	Never smokers	0.084	0.028	0.040	No
Snodgrass 2016	Prospective cohort	Asthma	Both	0 - 0	Home	Family (anyone in household)	Never smokers	0.536	0.108	0.108	Yes
Strachan 1996	Prospective cohort	Asthma	Both	0 - 0	Home	Paternal	Adj never smokers	-0.083	0.116	0.164	No
Strachan 1996	Prospective cohort	Asthma	Both	0 - 0	Home	Maternal	Adj never smokers	0.174	0.166	0.235	No
Sun 2015	Prospective cohort	Asthma	Both	0 - 0	Home	Parental	Never smokers	-0.117	0.359	0.359	No
Surdu 2006	Case-control	Asthma	Both	2 - 14	Home	Family (anyone in household)	Adj never smokers	0.399	0.535	0.535	No
Tadaki 2009	Prospective cohort	Asthma	Both	0 - 0	Home	Any	Never smokers	0.039	0.450	0.450	No
Takemura 2001	Case-control	Asthma	Both	6 - 15	Home	Parental	Unknown	-0.051	0.043	0.043	Yes

Table S8. Summary of data inputs for asthma

Author	Study design	Health outcome	Sex	Age	Exposure location	Exposure source	Smoking status	Log effect size	Original SE of the log effect size	SE of the log effect size used in the model	Outlier
Tanaka 2008	Prospective cohort	Asthma	Both	0 - 0	Home	Family (anyone in household)	Never smokers	-0.288	0.448	0.448	No
Tanaka 2017	Prospective cohort	Asthma	Both	0 - 0	Home	Family (anyone in household)	Never smokers	0.157	0.129	0.129	No
Taveras 2006	Prospective cohort	Asthma	Both	0 - 0	Home	Maternal	Never smokers	0.039	0.051	0.051	No
Thacher 2014	Prospective cohort	Asthma	Both	0.167 - 0.167	Home	Parental	Never smokers	0.095	0.144	0.144	No
Thacher 2018	Prospective cohort	Asthma	Both	0 - 0	Home	Family (anyone in household)	Never smokers	0.049	0.078	0.078	No
Thorn 2001	Nested case-control	Asthma	Both	20 - 50	Home	Family (anyone in household)	Never smokers	0.875	0.274	0.274	No
Toizumi 2019	Prospective cohort	Asthma	Both	0 - 0	Home	Family (anyone in household)	Never smokers	-0.117	0.274	0.274	No
Usemann 2018	Prospective cohort	Asthma	Both	0 - 0	Home	Parental	Never smokers	0.223	0.446	0.446	No
Valk 2012	Prospective cohort	Asthma	Both	0 - 0	Home	Family (anyone in household)	Never smokers	0.198	0.196	0.277	No
Valk 2012	Prospective cohort	Asthma	Both	0 - 0	Home	Family (anyone in household)	Never smokers	-0.092	0.122	0.173	No

Table S8. Summary of data inputs for asthma

Author	Study design	Health outcome	Sex	Age	Exposure location	Exposure source	Smoking status	Log effect size	Original SE of the log effect size	SE of the log effect size used in the model	Outlier
Vázquez 2006	Case-control	Asthma	Both	13 - 18	Home	Family (anyone in household)	Any smoking status	0.425	0.133	0.133	No
Wada 2021	Prospective cohort	Asthma	Both	0 - 0	Home	Family (anyone in household)	Never smokers	0.149	0.145	0.145	No
Wang 2021	Prospective cohort	Asthma	Both	27 - 55	Home	Family (anyone in household)	Never smokers	-0.223	0.168	0.237	No
Wang 2021	Prospective cohort	Asthma	Both	27 - 55	Home	Family (anyone in household)	Never smokers	-0.198	0.161	0.228	No
Werff 2013	Prospective cohort	Asthma	Both	4 - 14	Home	Family (anyone in household)	Never smokers	0.239	0.398	0.398	No
Willers 1991	Case-control	Asthma	Both	3 - 15	Home	Parental	Unknown	0.685	0.441	0.441	No
Yang 1998	Case-control	Asthma	Both	3 - 15	Home	Family (anyone in household)	Never smokers	-0.186	0.303	0.303	No
Yang 1998	Case-control	Asthma	Both	6 - 12	Home	Parental	Never smokers	-0.186	0.218	0.218	No
Youssef 2018	Case-control	Asthma	Both	3 - 8	Home	Family (anyone in household)	Never smokers	1.883	0.955	0.955	No
Zejda 2003	Prospective cohort	Asthma	Both	7 - 9	Home	Parental	Adj never smokers	0.846	0.596	0.596	No

Table S8. Summary of data inputs for asthma

Author	Study design	Health outcome	Sex	Age	Exposure location	Exposure source	Smoking status	Log effect size	Original SE of the log effect size	SE of the log effect size used in the model	Outlier
Zheng 2002	Case-control	Asthma	Both	6 - 10	Home	Family (anyone in household)	Adj never smokers	0.336	0.139	0.139	No

Table S9. Summary of data inputs for lower respiratory infections

Author	Study design	Health outcome	Sex	Age	Exposure location	Exposure source	Smoking status	Log effect size	Original SE of the log effect size	SE of the log effect size used in the model	Outlier
Almirall 2014	Case-control	Lower respiratory infections	Both	14 - 100	Home	Family (anyone in household)	Never smokers	0.166	0.142	0.142	No
Arlington 2019	Prospective cohort	Lower respiratory infections	Both	0 - 0	Home	Family (anyone in household)	Never smokers	0.262	0.150	0.150	No
Baker 2006	Prospective cohort	Lower respiratory infections	Both	0 - 0	Home	Other family (not spouse)	Never smokers	0.255	0.125	0.125	No
Barsam 2013	Case-control	Lower respiratory infections	Both	0.5 - 13	Home	Family (anyone in household)	Never smokers	0.400	0.276	0.276	No
Behrooz 2018	Case-control	Lower respiratory infections	Both	0 - 0.999	Home	Family (anyone in household)	Never smokers	1.433	0.261	0.261	Yes
Bermúdez 2021	Prospective cohort	Lower respiratory infections	Both	0 - 0	Home	Parental	Never smokers	0.329	0.436	0.436	No
Broor 2001	Case-control	Lower respiratory infections	Both	0 - 4	Home	Paternal	Never smokers	0.215	0.206	0.291	No
Broor 2001	Case-control	Lower respiratory infections	Both	0 - 4	Home	Other family (not spouse)	Never smokers	-1.715	1.244	1.759	No
Broughton 2005	Prospective cohort	Lower respiratory infections	Both	0 - 0	Home	Parental	Never smokers	0.560	0.544	0.544	No

Table S9. Summary of data inputs for lower respiratory infections

Author	Study design	Health outcome	Sex	Age	Exposure location	Exposure source	Smoking status	Log effect size	Original SE of the log effect size	SE of the log effect size used in the model	Outlier
Charoenca 2013	Case-control	Lower respiratory infections	Both	0.08 - 4	Home	Parental	Never smokers	1.340	0.222	0.222	Yes
Colley 1974	Prospective cohort	Lower respiratory infections	Both	0 - 0	Home	Parental	Never smokers	0.381	0.214	0.303	No
Colley 1974	Prospective cohort	Lower respiratory infections	Both	0 - 0	Home	Parental	Never smokers	0.813	0.204	0.288	No
Dina 2021	Case-control	Lower respiratory infections	Both	1 - 5	Home	Family (anyone in household)	Never smokers	1.483	0.487	0.487	Yes
Duijts 2008	Prospective cohort	Lower respiratory infections	Both	0 - 0	Home	Maternal	Never smokers	0.344	0.187	0.187	No
Dwedar 2018	Case-control	Lower respiratory infections	Both	18 - 99	Home	Family (anyone in household)	Non-smokers (former and never)	0.166	0.142	0.142	No
Farr 2000	Case-control	Lower respiratory infections	Both	18 - 75	Home	Spouse	Non-smokers (former and never)	-0.211	0.219	0.310	No
Farr 2000	Case-control	Lower respiratory infections	Both	18 - 75	Home	Other family (not spouse)	Non-smokers (former and never)	0.086	0.193	0.272	No
Farr 2000	Case-control	Lower respiratory infections	Both	15 - 79	Home	Family (anyone in household)	Any smoking status	-0.248	0.337	0.337	No

Table S9. Summary of data inputs for lower respiratory infections

Author	Study design	Health outcome	Sex	Age	Exposure location	Exposure source	Smoking status	Log effect size	Original SE of the log effect size	SE of the log effect size used in the model	Outlier
Farzana 2017	Case-control	Lower respiratory infections	Both	0.164 - 1	Home	Parental	Never smokers	1.030	0.366	0.366	No
Fergusson 1985	Prospective cohort	Lower respiratory infections	Both	0 - 0	Home	Maternal only	Never smokers	0.505	0.163	0.283	No
Fergusson 1985	Prospective cohort	Lower respiratory infections	Both	0 - 0	Home	Paternal only	Never smokers	0.030	0.201	0.349	No
Fergusson 1985	Prospective cohort	Lower respiratory infections	Both	0 - 0	Home	Parental-both	Never smokers	0.489	0.163	0.283	No
Frassanito 2022	Case-control	Lower respiratory infections	Both	0 - 0.999	Home	Family (anyone in household)	Never smokers	0.742	0.203	0.203	No
Fuentes-Leonarte 2015	Prospective cohort	Lower respiratory infections	Both	0 - 0	Home	Maternal	Never smokers	-0.020	0.151	0.214	No
Fuentes-Leonarte 2015	Prospective cohort	Lower respiratory infections	Both	0 - 0	Home	Paternal	Never smokers	0.010	0.108	0.152	No
Goetghebuer 2004	Case-control	Lower respiratory infections	Both	0 - 1	Home	Parental	Never smokers	0.513	0.257	0.257	No
Grant 2012	Case-control	Lower respiratory infections	Both	0 - 4	Home	Family (anyone in household)	Never smokers	-0.151	0.154	0.154	No

Table S9. Summary of data inputs for lower respiratory infections

Author	Study design	Health outcome	Sex	Age	Exposure location	Exposure source	Smoking status	Log effect size	Original SE of the log effect size	SE of the log effect size used in the model	Outlier
Hassan 2001	Case-control	Lower respiratory infections	Both	0.167 - 5	Home	Family (anyone in household)	Never smokers	0.770	0.212	0.212	Yes
Håberg 2007	Prospective cohort	Lower respiratory infections	Both	0 - 0	Home	Parental	Never smokers	0.086	0.042	0.042	No
Islam 2021	Case-control	Lower respiratory infections	Both	0.5 - 5	Home	Family (anyone in household)	Never smokers	0.476	0.246	0.246	No
Johnson 1992	Case-control	Lower respiratory infections	Both	0.04 - 4	Home	Family (anyone in household)	Never smokers	0.405	0.406	0.406	No
Keskinoglu 2007	Case-control	Lower respiratory infections	Both	2 - 12	Home	Family (anyone in household)	Never smokers	-0.033	0.256	0.256	No
Koch 2003	Prospective cohort	Lower respiratory infections	Both	0.115 - 2	Home	Family (anyone in household)	Never smokers	0.756	0.250	0.250	No
Lanari 2015	Prospective cohort	Lower respiratory infections	Both	0 - 0	Other	Family (anyone in household)	Never smokers	0.000	0.234	0.331	No
Lanari 2015	Prospective cohort	Lower respiratory infections	Both	0 - 0	Home	Family (anyone in household)	Never smokers	-0.105	0.397	0.562	No
Liu 2022	Retrospective cohort	Lower respiratory infections	Both	3 - 6	Home	Family (anyone in household)	Never smokers	0.048	0.088	0.088	No

Table S9. Summary of data inputs for lower respiratory infections

Author	Study design	Health outcome	Sex	Age	Exposure location	Exposure source	Smoking status	Log effect size	Original SE of the log effect size	SE of the log effect size used in the model	Outlier
Liyanage 2021	Case-control	Lower respiratory infections	Both	0.167 - 5.083	Home	Family (anyone in household)	Never smokers	0.290	0.668	0.668	No
Loeb 2009	Case-control	Lower respiratory infections	Both	65 - 99	Home	Family (anyone in household)	Any smoking status	0.567	0.262	0.262	No
Marbury 1996	Prospective cohort	Lower respiratory infections	Both	0 - 0	Home	Maternal	Never smokers	0.405	0.103	0.103	No
McConnochie 1986	Case-control	Lower respiratory infections	Both	0 - 1	Home	Family (anyone in household)	Never smokers	1.166	0.416	0.416	No
Miyahara 2017	Prospective cohort	Lower respiratory infections	Both	0 - 0	Home	Paternal	Never smokers	0.565	0.180	0.180	No
Nenna 2017	Case-control	Lower respiratory infections	Both	0.5 - 2	Home	Family (anyone in household)	Never smokers	-0.653	0.292	0.292	Yes
Nuesslein 1999	Prospective cohort	Lower respiratory infections	Both	0 - 0	Home	Family (anyone in household)	Never smokers	0.077	0.941	0.941	No
Pullan 1982	Case-control	Lower respiratory infections	Both	0 - 0.999	Home	Maternal	Never smokers	0.902	0.267	0.378	No
Pullan 1982	Case-control	Lower respiratory infections	Both	0 - 0.999	Home	Paternal	Never smokers	0.653	0.276	0.390	No

Table S9. Summary of data inputs for lower respiratory infections

Author	Study design	Health outcome	Sex	Age	Exposure location	Exposure source	Smoking status	Log effect size	Original SE of the log effect size	SE of the log effect size used in the model	Outlier
Ramesh 2012	Case-control	Lower respiratory infections	Both	0.08 - 4	Other	Any	Never smokers	0.451	0.391	0.391	No
Robledo-Aceves 2018	Case-control	Lower respiratory infections	Both	0.082 - 1	Home	Family (anyone in household)	Never smokers	1.253	0.289	0.289	Yes
Roda 2011	Prospective cohort	Lower respiratory infections	Both	0 - 1	Home	Family (anyone in household)	Never smokers	0.049	0.106	0.106	No
Roux 2015	Prospective cohort	Lower respiratory infections	Both	0 - 0	Home	Maternal	Never smokers	0.663	0.195	0.195	No
Rylander 1995	Case-control	Lower respiratory infections	Both	0.329 - 1	Home	Paternal	Never smokers	0.531	0.384	0.665	No
Rylander 1995	Case-control	Lower respiratory infections	Both	0.329 - 1	Home	Maternal	Never smokers	0.693	0.354	0.613	No
Rylander 1995	Case-control	Lower respiratory infections	Both	0.329 - 1	Home	Maternal and paternal	Never smokers	0.693	0.309	0.536	No
Rylander 1995	Case-control	Lower respiratory infections	Both	1.5 - 4	Home	Paternal	Never smokers	0.095	0.439	0.761	No
Rylander 1995	Case-control	Lower respiratory infections	Both	1.5 - 4	Home	Maternal	Never smokers	0.833	0.421	0.728	No

Table S9. Summary of data inputs for lower respiratory infections

Author	Study design	Health outcome	Sex	Age	Exposure location	Exposure source	Smoking status	Log effect size	Original SE of the log effect size	SE of the log effect size used in the model	Outlier
Rylander 1995	Case-control	Lower respiratory infections	Both	1.5 - 4	Home	Maternal and paternal	Never smokers	0.262	0.344	0.596	No
Schulte-Hobein 1992	Prospective cohort	Lower respiratory infections	Both	0 - 0	Home	Maternal	Never smokers	0.241	0.228	0.228	No
Taylor 1987	Prospective cohort	Lower respiratory infections	Both	0 - 0	Home	Maternal	Never smokers	0.252	0.118	0.118	No
Tupasi 1990	Prospective cohort	Lower respiratory infections	Both	0 - 4	Home	Maternal and paternal	Never smokers	0.113	0.032	0.032	No
Törmänen 2018	Case-control	Lower respiratory infections	Both	0 - 0.5	Home	Maternal	Never smokers	1.591	0.468	0.661	No
Törmänen 2018	Case-control	Lower respiratory infections	Both	0 - 0.5	Home	Paternal	Never smokers	0.631	0.285	0.404	No
Verani 2016	Case-control	Lower respiratory infections	Both	0.153 - 4	Home	Parental	Never smokers	1.639	0.286	0.286	Yes
Victora 1994	Case-control	Lower respiratory infections	Both	0 - 1	Home	Paternal	Never smokers	-0.117	0.169	0.292	No
Victora 1994	Case-control	Lower respiratory infections	Both	0 - 1	Home	Maternal	Never smokers	0.020	0.193	0.334	No

Table S9. Summary of data inputs for lower respiratory infections

Author	Study design	Health outcome	Sex	Age	Exposure location	Exposure source	Smoking status	Log effect size	Original SE of the log effect size	SE of the log effect size used in the model	Outlier
Victora 1994	Case-control	Lower respiratory infections	Both	0 - 1	Home	Maternal and paternal	Never smokers	-0.062	0.160	0.276	No
Wenten 2005	Prospective cohort	Lower respiratory infections	Both	9 - 11	Home	Family (anyone in household)	Never smokers	0.412	0.153	0.153	No
Wright 1991	Prospective cohort	Lower respiratory infections	Both	0 - 0	Home	Maternal	Never smokers	0.554	0.277	0.277	No

Table S10. Summary of data inputs for chronic obstructive pulmonary disease

Author	Study design	Health outcome	Sex	Age	Exposure location	Exposure source	Smoking status	Log effect size	Original SE of the log effect size	SE of the log effect size used in the model	Outlier
Behrendt 2005	Case-control	Chronic obstructive pulmonary disease	Both	18 - 80	Home	Family (anyone in household)	Non-smokers (former and never)	-0.580	0.241	0.341	Yes
Behrendt 2005	Case-control	Chronic obstructive pulmonary disease	Both	18 - 80	Work	Any	Non-smokers (former and never)	-1.347	0.594	0.840	Yes
Chan-Yeung 2007	Case-control	Chronic obstructive pulmonary disease	Both	18 - 99	Home or work	Any	Any smoking status	0.916	0.494	0.494	No
Chen 2013	Case-control	Chronic obstructive pulmonary disease	Both	27 - 85	Other	Any	Any smoking status	0.429	0.189	0.189	No
David 2005	Prospective cohort	Chronic obstructive pulmonary disease	Both	45 - 74	Home	Family (anyone in household)	Never smokers	0.833	0.269	0.381	No
David 2005	Prospective cohort	Chronic obstructive pulmonary disease	Both	45 - 74	Work	Any	Never smokers	0.412	0.185	0.262	No
Dennis 1996	Case-control	Chronic obstructive pulmonary disease	Female	35 - 99	Home	Spouse	Any smoking status	0.713	0.323	0.323	No

Table S10. Summary of data inputs for chronic obstructive pulmonary disease

Author	Study design	Health outcome	Sex	Age	Exposure location	Exposure source	Smoking status	Log effect size	Original SE of the log effect size	SE of the log effect size used in the model	Outlier
Ding 2015	Case-control	Chronic obstructive pulmonary disease	Both	32 - 98	Other	Any	Any smoking status	0.495	0.231	0.231	No
Diver 2018	Prospective cohort	Chronic obstructive pulmonary disease	Both	50 - 74	Home	Family (anyone in household)	Never smokers	-0.010	0.311	0.311	No
Enstrom 2003	Prospective cohort	Chronic obstructive pulmonary disease	Male	30 - 96	Home	Spouse	Never smokers	0.247	0.293	0.293	No
Enstrom 2003	Prospective cohort	Chronic obstructive pulmonary disease	Female	30 - 96	Home	Spouse	Never smokers	0.113	0.211	0.211	No
Gerbase 2006	Prospective cohort	Chronic obstructive pulmonary disease	Both	18 - 60	Other	Any	Never smokers	-0.236	0.331	0.331	No
He 2012	Prospective cohort	Chronic obstructive pulmonary disease	Both	51.5 - 87.8	Home or work	Any	Never smokers	0.833	0.396	0.396	No
Huang 2019	Case-control	Chronic obstructive pulmonary disease	Both	18 - 99	Other	Any	Never smokers	-0.010	0.148	0.148	No

Table S10. Summary of data inputs for chronic obstructive pulmonary disease

Author	Study design	Health outcome	Sex	Age	Exposure location	Exposure source	Smoking status	Log effect size	Original SE of the log effect size	SE of the log effect size used in the model	Outlier
Johannessen 2012	Case-control	Chronic obstructive pulmonary disease	Female	40 - 79	Work	Any	Any smoking status	0.329	0.453	0.453	No
Johannessen 2012	Case-control	Chronic obstructive pulmonary disease	Male	40 - 79	Home	Family (anyone in household)	Any smoking status	-0.051	0.220	0.311	No
Johannessen 2012	Case-control	Chronic obstructive pulmonary disease	Male	40 - 79	Work	Any	Any smoking status	-0.083	0.223	0.316	No
Lee 1986	Case-control	Chronic obstructive pulmonary disease	Both	35 - 74	Home	Spouse	Never smokers	-0.237	0.405	0.405	No
McGhee 2005	Case-control	Chronic obstructive pulmonary disease	Both	60 - 99	Home	Family (anyone in household)	Never smokers	0.688	0.226	0.226	No
Pahwa 2019	Prospective cohort	Chronic obstructive pulmonary disease	Both	18 - 99	Home	Family (anyone in household)	Any smoking status	-0.968	0.628	0.628	No
Salama 2020	Case-control	Chronic obstructive pulmonary disease	Both	19 - 99	Other	Any	Any smoking status	0.470	0.603	0.603	No

Table S10. Summary of data inputs for chronic obstructive pulmonary disease

Author	Study design	Health outcome	Sex	Age	Exposure location	Exposure source	Smoking status	Log effect size	Original SE of the log effect size	SE of the log effect size used in the model	Outlier
Salameh 2012	Case-control	Chronic obstructive pulmonary disease	Both	40 - 99	Work	Any	Any smoking status	0.637	0.263	0.372	No
Salameh 2012	Case-control	Chronic obstructive pulmonary disease	Both	40 - 99	Home	Family (anyone in household)	Any smoking status	0.940	0.201	0.284	No
Sandler 1989	Prospective cohort	Chronic obstructive pulmonary disease	Male	25 - 99	Home	Family (anyone in household)	Never smokers	-0.073	0.894	0.894	No
Sandler 1989	Prospective cohort	Chronic obstructive pulmonary disease	Female	25 - 99	Home	Family (anyone in household)	Never smokers	1.732	0.795	0.795	No
Vineis 2005	Nested case-control	Chronic obstructive pulmonary disease	Both	35 - 74	Home or work	Any	Non-smokers (former and never)	0.776	0.603	0.603	No
Wu 2010	Case-control	Chronic obstructive pulmonary disease	Female	40 - 99	Home or work	Any	Non-smokers (former and never)	0.565	0.259	0.367	No
Wu 2010	Case-control	Chronic obstructive pulmonary disease	Female	40 - 99	Home or work	Any	Non-smokers (former and never)	1.345	0.411	0.581	No

Table S10. Summary of data inputs for chronic obstructive pulmonary disease

Author	Study design	Health outcome	Sex	Age	Exposure location	Exposure source	Smoking status	Log effect size	Original SE of the log effect size	SE of the log effect size used in the model	Outlier
Xu 2007	Nested case-control	Chronic obstructive pulmonary disease	Both	35 - 99	Other	Any	Any smoking status	-0.174	0.086	0.086	Yes

Table S12. Summary of data inputs for type 2 diabetes mellitus

Author	Study design	Health outcome	Sex	Age	Exposure location	Exposure source	Smoking status	Log effect size	Original SE of the log effect size	SE of the log effect size used in the model	Outlier
Hayashino 2008	Prospective cohort	Diabetes mellitus type 2	Both	19 - 69	Work	Any	Non-smokers (former and never)	0.593	0.281	0.397	No
Hayashino 2008	Prospective cohort	Diabetes mellitus type 2	Both	19 - 69	Home	Family (anyone in household)	Non-smokers (former and never)	-0.223	0.284	0.402	No
Huang 2020	Prospective cohort	Diabetes mellitus type 2	Female	30 - 79	Other	Any	Never smokers	0.157	0.080	0.080	No
Jiang 2019	Prospective cohort	Diabetes mellitus type 2	Female	20 - 79	Home	Family (anyone in household)	Never smokers	0.086	0.072	0.102	No
Jiang 2019	Prospective cohort	Diabetes mellitus type 2	Female	20 - 79	Home	Family (anyone in household)	Never smokers	0.223	0.061	0.086	No
Kim 2021	Retrospective cohort	Diabetes mellitus type 2	Both	18 - 99	Home or work	Any	Never smokers	0.174	0.060	0.060	No
Kowall 2010	Prospective cohort	Diabetes mellitus type 2	Both	55 - 74	Home or work	Any	Never smokers	0.875	0.454	0.454	No
Kowall 2010	Prospective cohort	Diabetes mellitus type 2	Both	55 - 74	Home or work	Any	Former smokers	0.956	0.492	0.492	No
Oba 2020	Prospective cohort	Diabetes mellitus type 2	Female	40 - 69	Home	Spouse	Never smokers	0.030	0.095	0.095	No
Rias 2020	Case-control	Diabetes mellitus type 2	Both	18 - 75	Other	Any	Never smokers	0.990	0.486	0.486	Yes
Zeng 2022	Case-control	Diabetes mellitus type 2	Female	25 - 95	Other	Any	Non-smokers (former and never)	-0.673	0.328	0.328	Yes

Table S12. Summary of data inputs for type 2 diabetes mellitus

Author	Study design	Health outcome	Sex	Age	Exposure location	Exposure source	Smoking status	Log effect size	Original SE of the log effect size	SE of the log effect size used in the model	Outlier
Zhang 2011	Prospective cohort	Diabetes mellitus type 2	Female	41 - 55	Other	Any	Never smokers	0.095	0.069	0.097	No
Zhang 2011	Prospective cohort	Diabetes mellitus type 2	Female	41 - 55	Other	Any	Never smokers	0.148	0.077	0.108	No

Table S12. Summary of data inputs for otitis media

Author	Study design	Health outcome	Sex	Age	Exposure location	Exposure source	Smoking status	Log effect size	Original SE of the log effect size	SE of the log effect size used in the model	Outlier
Adair-Bischoff 1998	Case-control	Otitis media	Both	0 - 3.083	Home	Family (anyone in household)	Never smokers	0.104	0.178	0.178	No
Alho 1993	Prospective cohort	Otitis media	Both	0.08 - 0.08	Home	Parental	Never smokers	0.157	0.052	0.052	No
Bentdal 2007	Prospective cohort	Otitis media	Both	0 - 0	Home	Parental	Never smokers	0.262	0.120	0.120	No
Clamp 2020	Prospective cohort	Otitis media	Both	1 - 1	Home	Family (anyone in household)	Never smokers	0.095	0.094	0.094	No
Collet 1995	Prospective cohort	Otitis media	Both	0 - 0	Home	Maternal	Never smokers	0.262	0.150	0.150	No
Costa 2004	Case-control	Otitis media	Both	0.5 - 5	Home	Family (anyone in household)	Never smokers	0.412	0.057	0.057	Yes
Daigler 1991	Case-control	Otitis media	Both	0 - 21	Home	Maternal	Never smokers	-0.102	0.245	0.347	No
Daigler 1991	Case-control	Otitis media	Both	0 - 21	Home	Paternal	Never smokers	-0.182	0.246	0.348	No
Daly 1999	Prospective cohort	Otitis media	Both	0 - 0	Home	Parental	Never smokers	0.262	0.150	0.150	No
Daly 2007	Prospective cohort	Otitis media	Both	0 - 0	Home	Family (anyone in household)	Never smokers	-0.051	0.130	0.130	No
Ey 1995	Prospective cohort	Otitis media	Both	0 - 0	Home	Maternal	Never smokers	0.231	0.161	0.161	No

Table S12. Summary of data inputs for otitis media

Author	Study design	Health outcome	Sex	Age	Exposure location	Exposure source	Smoking status	Log effect size	Original SE of the log effect size	SE of the log effect size used in the model	Outlier
Fuentes-Leonarte 2015	Prospective cohort	Otitis media	Both	0 - 0	Home	Maternal	Never smokers	0.157	0.151	0.213	No
Fuentes-Leonarte 2015	Prospective cohort	Otitis media	Both	0 - 0	Home	Paternal	Never smokers	0.223	0.108	0.152	No
Håberg 2010	Prospective cohort	Otitis media	Both	0 - 0.5	Home	Parental	Never smokers	0.010	0.073	0.103	No
Håberg 2010	Prospective cohort	Otitis media	Both	0 - 0.5	Home	Parental	Never smokers	0.174	0.089	0.127	No
Håberg 2010	Prospective cohort	Otitis media	Both	0.5 - 1	Home	Parental	Never smokers	0.039	0.037	0.052	No
Håberg 2010	Prospective cohort	Otitis media	Both	0.5 - 1	Home	Parental	Never smokers	0.077	0.045	0.063	No
Håberg 2010	Prospective cohort	Otitis media	Both	1 - 1.5	Home	Parental	Never smokers	0.000	0.031	0.043	No
Håberg 2010	Prospective cohort	Otitis media	Both	1 - 1.5	Home	Parental	Never smokers	0.030	0.040	0.056	No
Jensen 2013	Prospective cohort	Otitis media	Both	0 - 0	Home	Maternal	Never smokers	0.932	0.313	0.313	Yes
Koch 2011	Prospective cohort	Otitis media	Both	0 - 4	Home	Family (anyone in household)	Never smokers	0.262	0.363	0.363	No
Niclasen 2016	Prospective cohort	Otitis media	Both	0 - 0	Home	Maternal	Never smokers	0.048	0.022	0.022	No

Table S12. Summary of data inputs for otitis media

Author	Study design	Health outcome	Sex	Age	Exposure location	Exposure source	Smoking status	Log effect size	Original SE of the log effect size	SE of the log effect size used in the model	Outlier
Prins-van 2017	Prospective cohort	Otitis media	Both	0.115 - 0.115	Other	Any	Never smokers	0.457	0.311	0.311	No
Pukander 1985	Case-control	Otitis media	Both	2 - 3	Home	Parental	Never smokers	0.635	0.191	0.191	Yes
Samson 2020	Prospective cohort	Otitis media	Both	0 - 0	Home	Parental	Never smokers	1.430	0.653	0.653	No
Stenstrom 1993	Case-control	Otitis media	Both	0 - 4	Home	Family (anyone in household)	Never smokers	0.986	0.381	0.381	Yes
Ståhlberg 1986	Case-control	Otitis media	Both	0.833 - 3.75	Home	Parental-one	Never smokers	1.065	0.308	0.435	No
Ståhlberg 1986	Case-control	Otitis media	Both	0.833 - 3.75	Home	Parental-both	Never smokers	0.914	0.354	0.501	No
Tainio 1988	Prospective cohort	Otitis media	Both	0 - 0	Home	Parental	Never smokers	0.531	0.229	0.229	No
Teele 1989	Prospective cohort	Otitis media	Both	0 - 0.25	Home	Parental	Never smokers	0.043	0.026	0.026	No
Wijayanti 2021	Case-control	Otitis media	Both	6 - 12	Home	Family (anyone in household)	Never smokers	0.549	0.256	0.256	No
Yang 1999	Case-control	Otitis media	Both	6 - 12	Home	Family (anyone in household)	Never smokers	0.049	0.209	0.209	No

Section 3: Sensitivity Analyses

We conducted three sensitivity analyses to evaluate the robustness of our primary results with regard to changes in the included data. In the first analysis, we restricted the data to studies with a prospective cohort design, which is considered the gold standard of observational research. In the second one, we subset the data to observations from never-smokers only, removing observations associated with former smokers or those with any or unknown smoking status, in an attempt to further avoid the confounding effect of active smoking. In this analysis, observations that were reclassified as "never-smokers" due to the age of the participants (age < 15) (see Section 2.2), were included in the models. Finally, in the last analysis, we applied both of these restrictions concurrently. Specifically for asthma, we had an additional analysis where we included childhood (age 16 or less) studies only. Each sensitivity analysis with more than 10 observations was conducted with 10% trimming, while models with fewer than 10 observations were conducted without trimming, following the BPRF methodology. All other model parameters remained the same. For more details on each of the studies, see Tables S3-S12. Results from each of the sensitivity analyses are presented in section 3.1 below.

Table S13. Strength of the evidence for the relationship between secondhand smoking when including prospective cohort studies only

Health outcome	RR (95% UI without gamma)	RR (95% UI with gamma)	BPRF	ROS	Star rating	Publication bias	No. of studies	Selected bias covariates	Risk-Outcome pair included in GBD 2021
Ischemic heart disease	1.21 (1.14, 1.28)	1.21 (1.03, 1.42)	1.06	0.03	☆☆	No	15	Baseline exposure assessment; Not representative population	Y
Stroke	1.21 (1.12, 1.31)	1.21 (0.98, 1.5)	1.01	0.01	☆☆	No	12	Self-reported outcome	Y
Asthma	1.23 (1.18, 1.29)	1.23 (0.97, 1.57)	1.01	0.004	☆☆	No	68	adj_L1; self-reported outcome; children population	N
Otitis media	1.06 (1.03, 1.09)	1.06 (0.99, 1.13)	1	0.002	☆☆	Yes	16	None	Y
Type 2 diabetes mellitus	1.14 (1.06, 1.23)	1.14 (0.95, 1.38)	0.98	-0.01	☆	No	6	None	Y
Lower respiratory infections	1.25 (1.15, 1.36)	1.25 (0.89, 1.75)	0.94	-0.03	☆	No	21	None	Y
Tracheal, bronchus, and lung cancer	1.29 (1.16, 1.43)	1.29 (0.89, 1.86)	0.95	-0.03	☆	No	21	adj_L2	Y
Breast cancer	1.05 (1, 1.1)	1.05 (0.92, 1.2)	N/A	N/A		No	11	None	Y
Chronic obstructive pulmonary disease	1.21 (0.93, 1.57)	1.21 (0.55, 2.64)	N/A	N/A		No	7	None	Y

The reported mean relative risk (RR) and its 95% uncertainty interval (UI) reflect the risk an individual who has been exposed to secondhand smoking has of developing the outcome of interest relative to that of someone who has not been exposed to secondhand smoking. Gamma is the estimated between-study heterogeneity. We report the 95% UI when not incorporating between-study heterogeneity − "95% UI without gamma" − and when accounting for between-study heterogeneity − "95% UI with gamma." The Burden of Proof Risk Function (BPRF) is calculated for risk-outcome pairs that were found to have significant relationships at an 0.05 level of significance when not incorporating between-study heterogeneity (i.e., the lower bound of the 95% UI without gamma does not cross the null RR value of one). The BPRF corresponds to the 5th quantile estimate of relative risk accounting for between-study heterogeneity closest to the null for each risk–outcome pair, and it reflects the most conservative estimate of excess risk associated with secondhand smoking that is consistent with the available data. Since we define secondhand smoking exposure as dichotomous risk factors, i.e., an individual either has been exposed or has not, the risk-outcome score (ROS) is calculated as the signed value of log(BPRF) divided by two. Negative ROSs indicate that the evidence of the association is very weak and inconsistent. For ease of interpretation, we have transformed the ROS and BPRF into a star rating (1-5) with a higher rating representing a larger effect with stronger evidence. The potential existence of publication bias, which, if present, would affect the validity of the results, was tested using Egger's Regression. Included studies represent all available relevant data identified through our systematic reviews from January 1970 through July 2022. The selected bias covariates were chosen for inclusion in the model using algorithm that systematically detects bias covariates that correspond to significant sources of bias in the observations included. If selected, the observations were adjusted to better reflect the gold standard values of the covariate.

Table S14. Strength of the evidence for the relationship between secondhand smoking when including observations from never-smokers only

Health outcome	RR (95% UI without gamma)	RR (95% UI with gamma)	BPRF	ROS	Star rating	Publication bias	No. of studies	Selected bias covariates	Risk-Outcome pair included in GBD 2021
Ischemic heart disease	1.26 (1.19, 1.33)	1.26 (1.05, 1.52)	1.08	0.04	☆☆	No	28	Baseline exposure assessment; not representative population	Y
Stroke	1.18 (1.12, 1.24)	1.18 (1.04, 1.34)	1.06	0.03	☆☆	No	16	None	Y
Type 2 diabetes mellitus	1.16 (1.09, 1.24)	1.16 (0.98, 1.37)	1.01	0.004	☆☆	No	7	None	Y
Tracheal, bronchus, and lung cancer	1.24 (1.17, 1.31)	1.24 (0.97, 1.6)	1	0.002	☆☆	No	70	None	Y
Asthma	1.18 (1.14, 1.23)	1.18 (0.91, 1.53)	0.95	-0.02	☆	No	102	adj_L1; self-reported outcome; children population	N
Otitis media	1.12 (1.06, 1.18)	1.12 (0.92, 1.36)	0.95	-0.03	☆	No	24	Study design (not prospective cohort); self-reported outcome	Y
Lower respiratory infections	1.43 (1.3, 1.58)	1.43 (0.81, 2.54)	0.89	-0.06	☆	No	46	Not representative population; ever SHS exposure	Y
Breast cancer	1.09 (1.03, 1.15)	1.09 (0.85, 1.4)	0.89	-0.06	☆	No	37	Study design (not prospective cohort)	Y
Chronic obstructive pulmonary disease	1.15 (0.95, 1.4)	1.15 (0.65, 2.05)	N/A	N/A		No	9	adj_L1, adj_L0	Y

The reported mean relative risk (RR) and its 95% uncertainty interval (UI) reflect the risk an individual who has been exposed to secondhand smoking has of developing the outcome of interest relative to that of someone who has not been exposed to secondhand smoking. Gamma is the estimated between-study heterogeneity. We report the 95% UI when not incorporating between-study heterogeneity − "95% UI without gamma" − and when accounting for between-study heterogeneity − "95% UI with gamma." The Burden of Proof Risk Function (BPRF) is calculated for risk-outcome pairs that were found to have significant relationships at an 0.05 level of significance when not incorporating between-study heterogeneity (i.e., the lower bound of the 95% UI without gamma does not cross the null RR value of one). The BPRF corresponds to the 5th quantile estimate of relative risk accounting for between-study heterogeneity closest to the null for each risk–outcome pair, and it reflects the most conservative estimate of excess risk associated with secondhand smoking that is consistent with the available data. Since we define secondhand smoking exposure as dichotomous risk factors, i.e., an individual either has been exposed or has not, the risk-outcome score (ROS) is calculated as the signed value of log(BPRF) divided by two. Negative ROSs indicate that the evidence of the association is very weak and inconsistent. For ease of interpretation, we have transformed the ROS and BPRF into a star rating (1-5) with a higher rating representing a larger effect with stronger evidence. The potential existence of publication bias, which, if present, would affect the validity of the results, was tested using Egger's Regression. Included studies represent all available relevant data identified through our systematic reviews from January 1970 through July 2022. The selected bias covariates were chosen for inclusion in the model using algorithm that systematically detects bias covariates that correspond to significant sources of bias in the observations included. If selected, the observations were adjusted to better reflect the gold standard values of the covariate.

Table S15. Strength of the evidence for the relationship between secondhand smoking when including prospective cohorts and observations from never-smokers only

Health outcome	RR (95% UI without gamma)	RR (95% UI with gamma)	BPRF	ROS	Star rating	Publication bias	No. of studies	Selected bias covariates	Risk-Outcome pair included in GBD 2021
Ischemic heart disease	1.23 (1.16, 1.3)	1.23 (1.04, 1.45)	1.07	0.04	☆☆	No	14	Baseline exposure assessment; not representative population	Y
Otitis media	1.06 (1.03, 1.09)	1.06 (0.99, 1.13)	1	0.002	☆☆	Yes	16	None	Y
Stroke	1.2 (1.1, 1.3)	1.2 (0.96, 1.49)	1	-0.001	☆	No	10	None	Y
Asthma	1.19 (1.14, 1.24)	1.19 (0.95, 1.5)	0.98	-0.01	☆	No	62	adj_L1; self-reported outcome; children population	N
Type 2 diabetes mellitus	1.14 (1.06, 1.23)	1.14 (0.95, 1.38)	0.97	-0.01	☆	No	5	None	Y
Lower respiratory infections	1.25 (1.15, 1.36)	1.25 (0.89, 1.75)	0.94	-0.03	☆	No	21	None	Y
Tracheal, bronchus, and lung cancer	1.13 (1.02, 1.26)	1.13 (0.8, 1.61)	0.84	-0.08	☆	No	17	adj_L0	Y
Chronic obstructive pulmonary disease	1.32 (1.04, 1.68)	1.32 (0.67, 2.59)	0.75	-0.14	☆	No	6	None	Y
Breast cancer	1.05 (1, 1.1)	1.05 (0.92, 1.2)	N/A	N/A		No	11	None	Y

The reported mean relative risk (RR) and its 95% uncertainty interval (UI) reflect the risk an individual who has been exposed to secondhand smoking has of developing the outcome of interest relative to that of someone who has not been exposed to secondhand smoking. Gamma is the estimated between-study heterogeneity. We report the 95% UI when not incorporating between-study heterogeneity − "95% UI without gamma" − and when accounting for between-study heterogeneity − "95% UI with gamma." The Burden of Proof Risk Function (BPRF) is calculated for risk-outcome pairs that were found to have significant relationships at an 0.05 level of significance when not incorporating between-study heterogeneity (i.e., the lower bound of the 95% UI without gamma does not cross the null RR value of one). The BPRF corresponds to the 5th quantile estimate of relative risk accounting for between-study heterogeneity closest to the null for each risk–outcome pair, and it reflects the most conservative estimate of excess risk associated with secondhand smoking that is consistent with the available data. Since we define secondhand smoking exposure as dichotomous risk factors, i.e., an individual either has been exposed or has not, the risk-outcome score (ROS) is calculated as the signed value of log(BPRF) divided by two. Negative ROSs indicate that the evidence of the association is very weak and inconsistent. For ease of interpretation, we have transformed the ROS and BPRF into a star rating (1-5) with a higher rating representing a larger effect with stronger evidence. The potential existence of publication bias, which, if present, would affect the validity of the results, was tested using Egger's Regression. Included studies represent all available relevant data identified through our systematic reviews from January 1970 through July 2022. The selected bias covariates were chosen for inclusion in the model using algorithm that systematically detects bias covariates that correspond to significant sources of bias in the observations included. If selected, the observations were adjusted to better reflect the gold standard values of the covariate.

Table S16. Strength of the evidence for the relationship between secondhand smoking and asthma when including data from children aged 16 or less only

Health outcome	RR (95% UI without gamma)	RR (95% UI with gamma)	BPRF	ROS	Star rating	Publication bias	No. of studies	Selected bias covariates	Risk-Outcome pair included in GBD 2021
Asthma	1.2 (1.15, 1.25)	1.2 (0.91, 1.57)	0.95	-0.02	☆	No	96	Selection bias	N

The reported mean relative risk (RR) and its 95% uncertainty interval (UI) reflect the risk an individual who has been exposed to secondhand smoking has of developing the outcome of interest relative to that of someone who has not been exposed to secondhand smoking. Gamma is the estimated between-study heterogeneity. We report the 95% UI when not incorporating between-study heterogeneity − "95% UI without gamma" − and when accounting for between-study heterogeneity − "95% UI with gamma." The Burden of Proof Risk Function (BPRF) is calculated for risk-outcome pairs that were found to have significant relationships at an 0.05 level of significance when not incorporating between-study heterogeneity (i.e., the lower bound of the 95% UI without gamma does not cross the null RR value of one). The BPRF corresponds to the 5th quantile estimate of relative risk accounting for between-study heterogeneity closest to the null for each risk–outcome pair, and it reflects the most conservative estimate of excess risk associated with secondhand smoking that is consistent with the available data. Since we define secondhand smoking exposure as dichotomous risk factors, i.e., an individual either has been exposed or has not, the risk-outcome score (ROS) is calculated as the signed value of log(BPRF) divided by two. Negative ROSs indicate that the evidence of the association is very weak and inconsistent. For ease of interpretation, we have transformed the ROS and BPRF into a star rating (1-5) with a higher rating representing a larger effect with stronger evidence. The potential existence of publication bias, which, if present, would affect the validity of the results, was tested using Egger's Regression. Included studies represent all available relevant data identified through our systematic reviews from January 1970 through July 2022. The selected bias covariates were chosen for inclusion in the model using algorithm that systematically detects bias covariates that correspond to significant sources of bias in the observations included. If selected, the observations were adjusted to better reflect the gold standard values of the covariate.

Section 4: GATHER and PRISMA checklists

Section 4.1: PRISMA

Table S17. PRISMA 2020 Abstract Checklist

Section and Topic	Item #	Checklist item	Reported (Yes/No)
**TITLE**			
Title	1	Identify the report as a systematic review.	Identified in the abstract but not in the title. The title reflects the review and meta-analysis methodology used.
**BACKGROUND**			
Objectives	2	Provide an explicit statement of the main objective(s) or question(s) the review addresses.	Yes
**METHODS**			
Eligibility criteria	3	Specify the inclusion and exclusion criteria for the review.	Due to space constrains, information is provided in the main text and supplementary information.
Information sources	4	Specify the information sources (e.g. databases, registers) used to identify studies and the date when each was last searched.	Space limitation required us to describe search details in the main text and supplementary information.
Risk of bias	5	Specify the methods used to assess risk of bias in the included studies.	Yes, briefly as incorporated in the burden of proof meta-analytic approach; More detail provided in the main text and supplementary information.
Synthesis of results	6	Specify the methods used to present and synthesise results.	Yes, briefly as incorporated in the burden of proof meta-analytic approach; More detail provided in the main text and supplementary information.
**RESULTS**			
Included studies	7	Give the total number of included studies and participants and summarise relevant characteristics of studies.	Space limitation required us to describe these details in the main text and supplementary information.
Synthesis of results	8	Present results for main outcomes, preferably indicating the number of included studies and participants for each. If meta-analysis was done, report the summary estimate and confidence/credible interval. If comparing groups, indicate the direction of the effect (i.e. which group is favoured).	Briefly, interpretation of star rating is reported, which is the culmination of the primary results. Further details and summary estimates are provided in the main text.
**DISCUSSION**			
Limitations of evidence	9	Provide a brief summary of the limitations of the evidence included in the review (e.g. study risk of bias, inconsistency and imprecision).	Not in abstract. Covered in detail in the main text and supplementary information.
Interpretation	10	Provide a general interpretation of the results and important implications.	Yes
**OTHER**			
Funding	11	Specify the primary source of funding for the review.	Not in abstract due to space constraints but described in the main text.
Registration	12	Provide the register name and registration number.	N/A

Table S18. PRISMA 2020 Manuscript Checklist

Section and Topic	Item #	Checklist item	Location where item is reported
**TITLE**			
Title	1	Identify the report as a systematic review.	Not in the title due to focus on analytical methodology. Instead, this is explicitly stated in the Methods section of the manuscript.
**ABSTRACT**			
Abstract	2	See the PRISMA 2020 for Abstracts checklist.	Table S17
**INTRODUCTION**			
Rationale	3	Describe the rationale for the review in the context of existing knowledge.	Main - Introduction
Objectives	4	Provide an explicit statement of the objective(s) or question(s) the review addresses.	Main – Introduction
**METHODS**			
Eligibility criteria	5	Specify the inclusion and exclusion criteria for the review and how studies were grouped for the syntheses.	Methods; Supplementary Information section 1.2
Information sources	6	Specify all databases, registers, websites, organisations, reference lists and other sources searched or consulted to identify studies. Specify the date when each source was last searched or consulted.	Methods; Results; Supplementary Information section 1
Search strategy	7	Present the full search strategies for all databases, registers and websites, including any filters and limits used.	Supplementary information section 1.1
Selection process	8	Specify the methods used to decide whether a study met the inclusion criteria of the review, including how many reviewers screened each record and each report retrieved, whether they worked independently, and if applicable, details of automation tools used in the process.	Methods
Data collection process	9	Specify the methods used to collect data from reports, including how many reviewers collected data from each report, whether they worked independently, any processes for obtaining or confirming data from study investigators, and if applicable, details of automation tools used in the process.	Supplementary information section 1.5
Data items	10a	List and define all outcomes for which data were sought. Specify whether all results that were compatible with each outcome domain in each study were sought (e.g. for all measures, time points, analyses), and if not, the methods used to decide which results to collect.	Methods; Supplementary Information 1.3
	10b	List and define all other variables for which data were sought (e.g. participant and intervention characteristics, funding sources). Describe any assumptions made about any missing or unclear information.	Methods; Supplementary Information table S2-S3
Study risk of bias assessment	11	Specify the methods used to assess risk of bias in the included studies, including details of the tool(s) used, how many reviewers assessed each study and whether they worked independently, and if applicable, details of automation tools used in the process.	Methods; Supplementary Information section 5
Effect measures	12	Specify for each outcome the effect measure(s) (e.g. risk ratio, mean difference) used in the synthesis or presentation of results.	Methods
Synthesis methods	13a	Describe the processes used to decide which studies were eligible for each synthesis (e.g. tabulating the study intervention characteristics and comparing against the planned groups for each synthesis (item #5)).	Methods; Supplementary information section 2
	13b	Describe any methods required to prepare the data for presentation or synthesis, such as handling of missing summary statistics, or data conversions.	Methods; Supplementary information section 2
	13c	Describe any methods used to tabulate or visually display results of individual studies and syntheses.	Captions of figures
	13d	Describe any methods used to synthesize results and provide a rationale for the choice(s). If meta-analysis was performed, describe the model(s), method(s) to identify the presence and extent of statistical heterogeneity, and software package(s) used.	Methods overview; Code availability statement.
	13e	Describe any methods used to explore possible causes of heterogeneity among study results (e.g. subgroup analysis, meta-regression).	Methods;
	13f	Describe any sensitivity analyses conducted to assess robustness of the synthesized results.	Methods; Supplementary information section 3; Supplementary Tables S13-16.
Reporting bias assessment	14	Describe any methods used to assess risk of bias due to missing results in a synthesis (arising from reporting biases).	Methods; Table 2; Supplementary tables S13-16.

Certainty assessment	15	Describe any methods used to assess certainty (or confidence) in the body of evidence for an outcome.	Methods
**RESULTS**			
Study selection	16a	Describe the results of the search and selection process, from the number of records identified in the search to the number of studies included in the review, ideally using a flow diagram.	PRISMA flow diagrams (Supplementary Information Figs. S1-S9)
	16b	Cite studies that might appear to meet the inclusion criteria, but which were excluded, and explain why they were excluded.	N/A
Study characteristics	17	Cite each included study and present its characteristics.	Supplementary Information Table S3
Risk of bias in studies	18	Present assessments of risk of bias for each included study.	Supplementary Information Table S20
Results of individual studies	19	For all outcomes, present, for each study: (a) summary statistics for each group (where appropriate) and (b) an effect estimate and its precision (e.g. confidence/credible interval), ideally using structured tables or plots.	Results section; Table 2.
Results of syntheses	20a	For each synthesis, briefly summarise the characteristics and risk of bias among contributing studies.	Results section; Table 2.
	20b	Present results of all statistical syntheses conducted. If meta-analysis was done, present for each the summary estimate and its precision (e.g. confidence/credible interval) and measures of statistical heterogeneity. If comparing groups, describe the direction of the effect.	Results section; Table 2. Between-study heterogeneity (Supplementary Information Table S21)
	20c	Present results of all investigations of possible causes of heterogeneity among study results.	Results section
	20d	Present results of all sensitivity analyses conducted to assess the robustness of the synthesized results.	Results section; Supplementary information Tables S13-S16.
Reporting biases	21	Present assessments of risk of bias due to missing results (arising from reporting biases) for each synthesis assessed.	Results section; Figures 1-4
Certainty of evidence	22	Present assessments of certainty (or confidence) in the body of evidence for each outcome assessed.	Results section; Table 2; Figures 1-4
**DISCUSSION**			
Discussion	23a	Provide a general interpretation of the results in the context of other evidence.	Discussion paragraphs 1-3
	23b	Discuss any limitations of the evidence included in the review.	Discussion paragraph 6
	23c	Discuss any limitations of the review processes used.	Discussion paragraph 6
	23d	Discuss implications of the results for practice, policy, and future research.	Discussion paragraphs 4-5
**OTHER INFORMATION**			
Registration and protocol	24a	Provide registration information for the review, including register name and registration number, or state that the review was not registered.	The entirety of the Global Burden of Diseases, Injuries, and Risk Factors Study has been registered and approved through the UW IRB, as detailed in Methods section (“Overview”). The systematic review was not registered separately.
	24b	Indicate where the review protocol can be accessed, or state that a protocol was not prepared.	N/A
	24c	Describe and explain any amendments to information provided at registration or in the protocol.	N/A
Support	25	Describe sources of financial or non-financial support for the review, and the role of the funders or sponsors in the review.	Main text “Acknowledgments”
Competing interests	26	Declare any competing interests of review authors.	Main text “Competing interests”
Availability of data, code and other materials	27	Report which of the following are publicly available and where they can be found: template data collection forms; data extracted from included studies; data used for all analyses; analytic code; any other materials used in the review.	Main text “Data availability” and “Code availability” sections; data collection form template in SI Table S2

Section 4.2: GATHER

Table S19. GATHER Checklist

Item #	Checklist item	Reported on page #
**Objectives and funding**		
**1**	Define the indicator(s), populations (including age, sex, and geographic entities), and time period(s) for which estimates were made.	Methods (18-21)
**2**	List the funding sources for the work.	72
**Data Inputs**		
For all data inputs from multiple sources that are synthesized as part of the study:		
**3**	Describe how the data were identified and how the data were accessed.	Methods (20-21)
**4**	Specify the inclusion and exclusion criteria. Identify all ad-hoc exclusions.	Methods (20-21); Supplementary Information (14-15)
**5**	Provide information on all included data sources and their main characteristics. For each data source used, report reference information or contact name/institution, population represented, data collection method, year(s) of data collection, sex and age range, diagnostic criteria or measurement method, and sample size, as relevant.	Supplementary Information Table S3 (37-92)
**6**	Identify and describe any categories of input data that have potentially important biases (e.g., based on characteristics listed in item 5).	Methods (23-24); Supplementary Tables S4-S12
For data inputs that contribute to the analysis but were not synthesized as part of the study:		
**7**	Describe and give sources for any other data inputs.	NA
For all data inputs:		
**8**	Provide all data inputs in a file format from which data can be efficiently extracted (e.g., a spreadsheet rather than a PDF), including all relevant meta-data listed in item 5. For any data inputs that cannot be shared because of ethical or legal reasons, such as third-party ownership, provide a contact name or the name of the institution that retains the right to the data.	Supplementary Information (95-162)
**Data analysis**		
**9**	Provide a conceptual overview of the data analysis method. A diagram may be helpful.	Methods (18-19)
**10**	Provide a detailed description of all steps of the analysis, including mathematical formulae. This description should cover, as relevant, data cleaning, data pre-processing, data adjustments and weighting of data sources, and mathematical or statistical model(s).	Methods (22-26)
**11**	Describe how candidate models were evaluated and how the final model(s) were selected.	Methods (26)
**12**	Provide the results of an evaluation of model performance, if done, as well as the results of any relevant sensitivity analysis.	Supplementary Tables S13-S16
**13**	Describe methods for calculating uncertainty of the estimates. State which sources of uncertainty were, and were not, accounted for in the uncertainty analysis.	Methods (23-25)
**14**	State how analytic or statistical source code used to generate estimates can be accessed.	Code availability (27)
**Results and Discussion**		
**15**	Provide published estimates in a file format from which data can be efficiently extracted.	Table 2; Burden of Proof visualization tool (https://vizhub.healthdata.org/burden-of-proof/)
**16**	Report a quantitative measure of the uncertainty of the estimates (e.g. uncertainty intervals).	Table 2; Results (5-12)
**17**	Interpret results in light of existing evidence. If updating a previous set of estimates, describe the reasons for changes in estimates.	Discussion (12-15)
**18**	Discuss limitations of the estimates. Include a discussion of any modelling assumptions or data limitations that affect interpretation of the estimates.	Discussion (15)

Section 5: Study quality & bias assessment

As outlined in more detail in Zheng et al. and in the main manuscript, we used a covariate selection algorithm that systematically detects eligible covariates that reflected a significant source of bias in the included observations. The bias covariates created mainly followed the Grading of Recommendations, Assessment, Development and Evaluations (GRADE) approach, but we created additional ones that were relevant to the secondhand smoke case. In total, we created 13 potential bias covariates that were evaluated for their eligibility to be tested in the algorithm and potentially included in the model. The 'gold standard' value was coded as 0, while the alternatives were coded as 1. The covariates were defined as follows:

1. Level of adjustment for potential confounding variables

Three bias covariates were generated as a cascading dummy reflecting different levels of adjustment of the effect sizes included in our models. The categories were created after reviewing the overall availability and distribution of confounders across our data. Gold standard **maximally adjusted** (adj_L0) observations correspond to estimates that were maximally adjusted for age, sex, smoking, and at minimum 3 other potential confounders. Gold standard **adjusted for smoking, age, sex and 1-2 more** (adj_L1) observations are those that at minimum account for participants' smoking status, age, sex, and 1 or 2 more confounders. Finally, gold standard **adjusted for age, sex, and smoking** (adj_L2) observations are any that at least control for the age, sex, and smoking status of participants, regardless of their controlling of other potential confounders.

Bias covariates to evaluate the level of adjustment of included data points

Level of adjustment	Definition	Cascading dummy		
		L0	L1	L2
Insufficient	Does not control for age, sex, and smoking status regardless of whether it controls for other variables	1	1	1
Minimal	Controls only for age, sex, and smoking	1	1	0
Moderate	Controls for age, sex, smoking, and 1-2 additional confounders	1	0	0
Maximal	Controls for age, sex, smoking, and 3 or more confounders	0	0	0

2. Representativeness of study population

Indicates whether the study sample is (0) or is not (1) geographically representative of underlying location

3. Prospective-cohort design

Indicates if the study has a prospective cohort design (0) or any other (1).

4. Outcome assessment

Reflects how the cases for the study were ascertained. The gold standard is that outcomes were ascertained through physician diagnosis, disease registry, medical record review, or biomarker examination (0). The alternate for this bias covariate is that the outcome was self-reported by the study participants (1).

5. Selection bias

Captures if study is at risk for selection bias. Study determined to be at risk for selection bias (1) if loss to follow-up (cohorts) or percent for whom data not ascertained (case-controls) > 20%. Studies that did not report sufficient information for assessment were classified as at risk. Gold-standard cases are those not at risk for selection bias (0).

6. Exposure assessment

Indicates whether the exposure to secondhand smoke in cohort studies was assessed at baseline only (1) or multiple times during follow-up (0). Observations from case-control and nested-case control studies were classified as alternate (1).

7. Controlled for smoking

Differentiates effect sizes that were controlled for smoking (0) from those that were not (1), regardless of the adjustment for other confounders. Observations associated with never-smokers only, former-smokers only, or non-smokers only were considered controlled for smoking status (0).

8. Females only

Flag studies that were done among females only (1) and not with both sexes (0).

9. Broad secondhand smoke exposure location

Captures if the study reports on the exposure locations that best match our exposure definition (home, work) (0) or if it reports a broader unspecified location (any location) (1).

10. Non-current exposure to secondhand smoke

Indicates if the reported effect size is for current exposure (0) or ever/unspecified exposure (1). Gold standard observations were those that used current exposure to more closely aligns with the exposure definition in the Global Burden of Disease study.

11. Aggregate stroke definition – Hemorrhagic stroke:

Specifically for stroke, we created two bias covariates to account for possible differences between studies reporting sub-type-specific effect size only and those reporting stroke as an aggregated outcome. In the hemorrhagic stroke covariate, studies reporting on aggregated stroke or both sub-types (hemorrhagic and ischemic) are considered gold-standard cases (0), while those reporting hemorrhagic stroke only are the alternate (1).

12. Aggregate stroke definition – Ischemic stroke:

Similar to the previous covariate, studies reporting on aggregated stroke or both sub-types (hemorrhagic and ischemic) are considered gold-standard cases (0), while those reporting ischemic stroke only are the alternate (1).

13. Childhood asthma:

Studies were assigned a zero if performed among those aged 16 or less only (if cohort, age at enrollment plus follow-up time is equal or less than 16 years old). Studies including a broader age group were considered alternate. The age cutoff was decided based on data availability (most childhood studies would follow children up to 16 years or less).

We did not create bias covariates for evaluating the form of exposure assessment and reverse causation. All of the studies identified used self-reported exposure data. Unlike for other risk factors, reverse causation for secondhand smoke is unlikely since individuals are not likely to become exposed when they are already sick with the assessed health outcomes, so it was deemed to not be relevant for testing.

Bias covariates were eligible for testing in a model if there were two or more observations for each coded value of the covariate. If two or more bias covariates had the same values across all the observations, we randomly selected one for inclusion in our models. We present the covariates tested for each outcome in Table S20 (N/A indicates covariates that were not tested for the respective model). The selected bias covariates for each model are presented in Table 2 in the main text.

Table S20. Bias covariates tested in the primary model for each health outcome, by study

Author	Study design	Health outcome	Subpopulation	Not prosp. cohort	Out. self-reported	Exp. assessment	Potential selection bias	Adj. level 0	Adj. level 1	Adj. level 2	Not adj. smoking	Females only	Broad location exp.	Not current exp.	Asthma children	Hem. stroke	Isch. stroke
Al-Kubaisy 2005	Prospective cohort	Asthma	0	0	0	1	0	1	1	1	0	1	1	0	0	N/A	N/A
Al-Qerem 2016	Prospective cohort	Asthma	0	0	1	0	0	1	1	0	0	0	1	0	0	N/A	N/A
Arif 2017	Case-control	Asthma	0	1	0	1	1	1	1	1	0	1	1	0	0	N/A	N/A
Aversa 2021	Prospective cohort	Asthma	0	0	0	1	0	1	1	1	0	1	1	1	0	N/A	N/A
Azizi 1995	Case-control	Asthma	0	1	0	1	0	1	1	1	0	1	1	1	0	N/A	N/A
Balemans 2006	Prospective cohort	Asthma	0	0	1	1	1	1	1	1	0	1	1	1	1	N/A	N/A
Beijsterveldt 2008	Prospective cohort	Asthma	0	0	1	1	0	1	1	1	0	1	1	0	0	N/A	N/A
Bener 2007	Case-control	Asthma	0	1	0	1	0	1	1	1	0	1	1	1	0	N/A	N/A
Bergmann 2000	Prospective cohort	Asthma	0	0	0	1	0	1	1	0	0	0	1	1	0	N/A	N/A
Boker 2019	Case-control	Asthma	0	1	0	1	1	1	1	1	0	1	1	0	0	N/A	N/A
Boneberger 2011	Case-control	Asthma	0	1	0	1	0	1	1	0	0	0	1	1	0	N/A	N/A
Bozicević 2000	Case-control	Asthma	0	1	1	1	0	1	1	1	1	0	0	1	1	N/A	N/A
Butland 1997	Case-control	Asthma	0	1	1	1	1	1	1	0	0	0	1	1	0	N/A	N/A
Carlsten 2012	Prospective cohort	Asthma	0	0	1	0	1	1	1	0	0	0	1	1	0	N/A	N/A

Table S20. Bias covariates tested in the primary model for each health outcome, by study

Author	Study design	Health outcome	Subpopulation	Not prosp. cohort	Out. self-reported	Exp. assessment	Potential selection bias	Adj. level 0	Adj. level 1	Adj. level 2	Not adj. smoking	Females only	Broad location exp.	Not current exp.	Asthma children	Hem. stroke	Isch. stroke
Carr 2019	Prospective cohort	Asthma	0	0	1	1	1	1	1	0	0	0	1	0	0	N/A	N/A
Carrasco 2021	Prospective cohort	Asthma	0	0	1	1	0	1	1	0	0	0	1	0	0	N/A	N/A
Chan 2019	Case-crossover	Asthma	0	1	1	1	1	1	1	0	0	0	0	0	1	N/A	N/A
Charoenca 2013	Case-control	Asthma	0	1	0	1	0	1	1	1	0	1	1	1	0	N/A	N/A
Chen 2011	Nested case-control	Asthma	1	1	0	1	0	1	1	1	0	1	1	1	0	N/A	N/A
Clark 1994	Case-control	Asthma	0	1	1	1	0	1	1	1	0	1	1	1	0	N/A	N/A
Coogan 2015	Prospective cohort	Asthma	0	0	1	1	0	0	0	0	0	0	0	0	1	N/A	N/A
Daigler 1991	Case-control	Asthma	0	1	0	1	1	1	1	0	0	0	1	1	0	N/A	N/A
David 2005	Prospective cohort	Asthma	0	0	0	1	1	1	1	0	0	0	1	0	1	N/A	N/A
Dekker 2015	Prospective cohort	Asthma	0	0	0	1	0	1	1	0	0	0	1	0	0	N/A	N/A
Ehrlich 1992	Case-control	Asthma	0	1	0	1	0	1	1	0	0	0	1	1	0	N/A	N/A
Ehrlich 1996	Case-control	Asthma	1	1	1	1	1	1	1	1	0	1	1	0	0	N/A	N/A
El-Sharif 2003	Case-control	Asthma	0	1	1	1	1	1	1	0	0	0	1	1	0	N/A	N/A
Elder 1996	Prospective cohort	Asthma	0	0	0	1	0	1	1	1	0	1	1	1	0	N/A	N/A

Table S20. Bias covariates tested in the primary model for each health outcome, by study

Author	Study design	Health outcome	Subpopulation	Not prosp. cohort	Out. self-reported	Exp. assessment	Potential selection bias	Adj. level 0	Adj. level 1	Adj. level 2	Not adj. smoking	Females only	Broad location exp.	Not current exp.	Asthma children	Hem. stroke	Isch. stroke
Fagbule 1994	Case-control	Asthma	0	1	1	1	0	1	1	1	0	1	1	1	0	N/A	N/A
Fernando 2009	Case-control	Asthma	0	1	0	1	0	1	1	0	0	0	1	0	0	N/A	N/A
Flexeder 2019	Prospective cohort	Asthma	0	0	0	0	1	1	0	0	0	0	1	0	1	N/A	N/A
Flodin 1995	Case-control	Asthma	1	1	0	1	1	1	1	1	1	0	1	1	1	N/A	N/A
Frassanito 2022	Case-control	Asthma	0	1	0	1	1	1	1	1	0	1	1	1	0	N/A	N/A
Galobardes 2015	Prospective cohort	Asthma	0	0	0	1	1	1	1	0	0	0	1	0	0	N/A	N/A
Genuneit 2006	Prospective cohort	Asthma	1	0	1	1	1	1	1	0	0	0	1	0	1	N/A	N/A
Goksör 2006	Prospective cohort	Asthma	0	0	1	1	1	1	1	1	0	1	1	1	1	N/A	N/A
Grabenhenrich 2014	Prospective cohort	Asthma	0	0	0	0	1	1	1	0	0	0	1	0	1	N/A	N/A
Guo 2019	Case-control	Asthma	0	1	0	1	0	1	1	0	0	0	1	0	0	N/A	N/A
Hadnadjev 2011	Case-control	Asthma	0	1	0	1	0	1	1	1	0	1	1	0	0	N/A	N/A
Hagendorens 2005	Prospective cohort	Asthma	0	0	1	1	1	1	1	0	0	0	1	1	0	N/A	N/A
Hedman 2011	Prospective cohort	Asthma	0	0	1	0	0	1	1	0	0	0	1	0	1	N/A	N/A

Table S20. Bias covariates tested in the primary model for each health outcome, by study

Author	Study design	Health outcome	Subpopulation	Not prosp. cohort	Out. self-reported	Exp. assessment	Potential selection bias	Adj. level 0	Adj. level 1	Adj. level 2	Not adj. smoking	Females only	Broad location exp.	Not current exp.	Asthma children	Hem. stroke	Isch. stroke
Horwood 1985	Prospective cohort	Asthma	0	0	0	0	0	1	1	1	0	1	1	1	0	N/A	N/A
Huang 2022	Case-control	Asthma	0	1	0	1	0	1	1	0	0	0	1	0	1	N/A	N/A
Hunt 2011	Prospective cohort	Asthma	0	0	0	0	0	1	1	1	0	1	1	0	0	N/A	N/A
Hwang 2011	Case-control	Asthma	1	1	1	1	1	1	1	1	0	1	1	1	0	N/A	N/A
Håberg 2007	Prospective cohort	Asthma	1	0	1	1	0	1	1	0	0	0	1	1	0	N/A	N/A
Izuhara 2016	Prospective cohort	Asthma	0	0	1	1	0	1	1	0	0	0	1	1	1	N/A	N/A
Jaakkola 2001	Prospective cohort	Asthma	0	0	0	1	0	1	1	0	0	0	1	1	0	N/A	N/A
Jaakkola 2003	Case-control	Asthma	0	1	0	1	1	1	1	0	0	0	0	1	1	N/A	N/A
Jedrychowski 2008	Prospective cohort	Asthma	0	0	1	1	0	1	1	1	0	1	1	1	0	N/A	N/A
Kamran 2015	Case-control	Asthma	0	1	0	1	0	1	1	1	1	0	1	1	0	N/A	N/A
Kanoh 2012	Prospective cohort	Asthma	0	0	1	1	1	1	1	0	0	0	1	0	0	N/A	N/A
Karunasekera 2001	Case-control	Asthma	0	1	0	1	0	1	1	1	0	1	1	0	0	N/A	N/A
Khozime 2019	Case-control	Asthma	0	1	0	1	1	1	1	1	0	1	1	0	1	N/A	N/A
Kim 2018	Case-cohort	Asthma	0	1	0	1	1	1	0	0	0	0	1	0	0	N/A	N/A

Table S20. Bias covariates tested in the primary model for each health outcome, by study

Author	Study design	Health outcome	Subpopulation	Not prosp. cohort	Out. self-reported	Exp. assessment	Potential selection bias	Adj. level 0	Adj. level 1	Adj. level 2	Not adj. smoking	Females only	Broad location exp.	Not current exp.	Asthma children	Hem. stroke	Isch. stroke
Klinnert 2001	Prospective cohort	Asthma	0	0	0	1	0	1	1	1	0	1	1	1	0	N/A	N/A
Kobyletzki 2012	Prospective cohort	Asthma	0	0	1	1	1	1	1	0	0	0	1	1	0	N/A	N/A
Kumar 2021	Case-control	Asthma	0	1	0	1	1	1	1	1	0	1	1	1	0	N/A	N/A
Kurukulaaratchy 2006	Prospective cohort	Asthma	1	0	1	0	0	1	1	0	0	0	1	0	0	N/A	N/A
Lawson 2014	Prospective cohort	Asthma	1	0	1	0	1	0	0	0	0	0	1	0	1	N/A	N/A
Leen 1994	Case-control	Asthma	0	1	0	1	0	1	1	1	1	1	1	1	0	N/A	N/A
Lemanske 2005	Prospective cohort	Asthma	0	0	0	1	0	1	1	1	0	1	1	1	0	N/A	N/A
Li 2013	Prospective cohort	Asthma	0	0	0	1	1	1	0	0	0	0	1	1	1	N/A	N/A
Lilljeqvist 1997	Case-control	Asthma	0	1	1	1	1	1	1	1	0	1	1	0	0	N/A	N/A
Litonjua 2001	Prospective cohort	Asthma	0	0	0	1	0	1	1	1	0	1	1	0	0	N/A	N/A
Majeed 2008	Case-control	Asthma	0	1	0	1	0	1	1	1	0	1	1	1	0	N/A	N/A
Marbury 1996	Prospective cohort	Asthma	0	0	0	1	1	1	1	1	0	1	1	1	0	N/A	N/A
McConnell 2002	Prospective cohort	Asthma	0	0	0	1	0	1	1	1	1	0	1	0	1	N/A	N/A

Table S20. Bias covariates tested in the primary model for each health outcome, by study

Author	Study design	Health outcome	Subpopulation	Not prosp. cohort	Out. self-reported	Exp. assessment	Potential selection bias	Adj. level 0	Adj. level 1	Adj. level 2	Not adj. smoking	Females only	Broad location exp.	Not current exp.	Asthma children	Hem. stroke	Isch. stroke
McKeever 2001	Prospective cohort	Asthma	0	0	0	1	0	1	1	1	0	1	1	0	0	N/A	N/A
Melsom 2001	Nested case-control	Asthma	1	1	1	1	0	1	1	1	1	1	1	0	1	N/A	N/A
Midodzi 2010	Prospective cohort	Asthma	1	0	1	1	0	1	1	1	0	1	1	0	0	N/A	N/A
Milanzi 2017	Prospective cohort	Asthma	1	0	1	0	1	1	1	0	0	0	1	1	1	N/A	N/A
Milner 2004	Prospective cohort	Asthma	0	0	0	1	0	1	1	1	0	1	1	1	0	N/A	N/A
Mommers 2005	Nested case-control	Asthma	0	1	1	1	1	1	1	0	0	0	1	1	0	N/A	N/A
Morfín-Maciel 2006	Case-control	Asthma	1	1	1	1	0	1	1	1	1	1	1	0	0	N/A	N/A
Morfín-Maciel 2006	Case-control	Asthma	1	1	1	1	0	1	1	1	0	1	1	0	0	N/A	N/A
Mpairwe 2019	Case-control	Asthma	0	1	0	1	0	1	1	0	0	0	1	0	1	N/A	N/A
Mumcuoglu 1994	Case-control	Asthma	0	1	0	1	0	1	1	1	1	1	1	1	0	N/A	N/A
Murray 2004	Prospective cohort	Asthma	0	0	0	0	0	1	1	0	0	0	1	0	0	N/A	N/A
Muñoz 2012	Case-control	Asthma	0	1	1	1	0	1	1	1	0	1	1	1	0	N/A	N/A
Neuman 2012	Prospective cohort	Asthma	0	0	1	1	1	1	1	0	0	0	1	1	0	N/A	N/A

Table S20. Bias covariates tested in the primary model for each health outcome, by study

Author	Study design	Health outcome	Subpopulation	Not prosp. cohort	Out. self-reported	Exp. assessment	Potential selection bias	Adj. level 0	Adj. level 1	Adj. level 2	Not adj. smoking	Females only	Broad location exp.	Not current exp.	Asthma children	Hem. stroke	Isch. stroke
Nguyen 2010	Nested case-control	Asthma	1	1	1	1	1	1	1	1	1	0	1	0	1	N/A	N/A
Norbäck 2018	Retrospective cohort	Asthma	1	1	1	1	1	1	1	0	0	0	1	1	0	N/A	N/A
O'Connell 1974	Case-control	Asthma	0	1	0	1	0	1	1	1	1	1	1	1	0	N/A	N/A
Oddy 1999	Prospective cohort	Asthma	0	0	1	1	1	1	1	1	0	1	1	1	0	N/A	N/A
Palvo 2008	Case-control	Asthma	1	1	1	1	1	1	1	1	0	1	1	0	0	N/A	N/A
Patrick 2020	Prospective cohort	Asthma	0	0	0	0	0	1	0	0	0	0	1	1	0	N/A	N/A
Pattemore 2018	Prospective cohort	Asthma	0	0	1	0	0	1	1	1	0	1	1	1	0	N/A	N/A
Pokharel 2001	Case-control	Asthma	0	1	1	1	0	1	1	1	1	1	1	1	0	N/A	N/A
Pokharel 2007	Case-control	Asthma	0	1	1	1	0	1	1	1	1	1	1	1	0	N/A	N/A
Polk 2004	Prospective cohort	Asthma	0	0	1	1	0	1	1	1	0	1	1	1	0	N/A	N/A
Polosa 2005	Retrospective cohort	Asthma	0	1	0	1	1	1	1	1	1	1	1	1	1	N/A	N/A
Ponsonby 2000	Prospective cohort	Asthma	0	0	1	1	1	1	1	0	0	0	1	1	0	N/A	N/A
Ratageri 2000	Case-control	Asthma	0	1	0	1	0	1	1	1	1	1	1	1	0	N/A	N/A
Rennie 2008	Case-control	Asthma	0	1	0	1	1	1	1	1	0	1	1	1	0	N/A	N/A

Table S20. Bias covariates tested in the primary model for each health outcome, by study

Author	Study design	Health outcome	Subpopulation	Not prosp. cohort	Out. self-reported	Exp. assessment	Potential selection bias	Adj. level 0	Adj. level 1	Adj. level 2	Not adj. smoking	Females only	Broad location exp.	Not current exp.	Asthma children	Hem. stroke	Isch. stroke
Rosa 2011	Prospective cohort	Asthma	0	0	0	0	0	1	1	0	0	0	1	1	0	N/A	N/A
Schroer 2009	Prospective cohort	Asthma	0	0	1	1	0	1	1	0	0	0	1	0	0	N/A	N/A
Sears 2002	Prospective cohort	Asthma	0	0	1	0	0	1	1	1	0	1	1	1	0	N/A	N/A
Selby 2018	Prospective cohort	Asthma	0	0	1	1	1	1	1	0	0	0	1	0	0	N/A	N/A
Sherman 1990	Prospective cohort	Asthma	0	0	0	0	0	1	1	0	0	0	1	1	1	N/A	N/A
Slob 2020	Retrospective cohort	Asthma	0	1	1	0	1	1	1	1	0	1	1	1	0	N/A	N/A
Snodgrass 2016	Prospective cohort	Asthma	0	0	1	0	0	1	1	1	0	1	1	0	0	N/A	N/A
Strachan 1996	Prospective cohort	Asthma	0	0	1	0	1	1	1	0	0	0	1	1	1	N/A	N/A
Sun 2015	Prospective cohort	Asthma	0	0	1	0	0	1	0	0	0	0	1	0	0	N/A	N/A
Surdu 2006	Case-control	Asthma	0	1	0	1	0	1	1	1	0	1	1	0	0	N/A	N/A
Tadaki 2009	Prospective cohort	Asthma	0	0	0	1	1	1	1	1	0	1	1	1	0	N/A	N/A
Takemura 2001	Case-control	Asthma	0	1	0	1	0	1	1	1	1	0	1	1	0	N/A	N/A

Table S20. Bias covariates tested in the primary model for each health outcome, by study

Author	Study design	Health outcome	Subpopulation	Not prosp. cohort	Out. self-reported	Exp. assessment	Potential selection bias	Adj. level 0	Adj. level 1	Adj. level 2	Not adj. smoking	Females only	Broad location exp.	Not current exp.	Asthma children	Hem. stroke	Isch. stroke
Tanaka 2008	Prospective cohort	Asthma	0	0	1	1	1	1	1	0	0	0	1	1	0	N/A	N/A
Tanaka 2017	Prospective cohort	Asthma	1	0	1	0	1	1	0	0	0	0	1	1	0	N/A	N/A
Taveras 2006	Prospective cohort	Asthma	0	0	1	0	1	1	1	0	0	0	1	1	0	N/A	N/A
Thacher 2014	Prospective cohort	Asthma	0	0	1	0	0	1	1	0	0	0	1	1	0	N/A	N/A
Thacher 2018	Prospective cohort	Asthma	0	0	1	0	1	1	1	0	0	0	1	1	0	N/A	N/A
Thorn 2001	Nested case-control	Asthma	0	1	0	1	1	1	1	0	0	0	1	1	1	N/A	N/A
Toizumi 2019	Prospective cohort	Asthma	0	0	1	1	1	1	0	0	0	0	1	0	0	N/A	N/A
Usemann 2018	Prospective cohort	Asthma	0	0	1	0	0	1	1	0	0	0	1	0	0	N/A	N/A
Valk 2012	Prospective cohort	Asthma	0	0	1	1	0	1	1	0	0	0	1	0	0	N/A	N/A
Vázquez 2006	Case-control	Asthma	0	1	1	1	0	1	1	1	1	1	1	1	1	N/A	N/A
Wada 2021	Prospective cohort	Asthma	0	0	1	1	0	1	1	0	0	0	1	1	0	N/A	N/A
Wang 2021	Prospective cohort	Asthma	0	0	0	1	1	1	0	0	0	0	1	0	1	N/A	N/A

Table S20. Bias covariates tested in the primary model for each health outcome, by study

Author	Study design	Health outcome	Subpopulation	Not prosp. cohort	Out. self-reported	Exp. assessment	Potential selection bias	Adj. level 0	Adj. level 1	Adj. level 2	Not adj. smoking	Females only	Broad location exp.	Not current exp.	Asthma children	Hem. stroke	Isch. stroke
Werff 2013	Prospective cohort	Asthma	1	0	1	1	0	1	1	1	0	1	1	0	1	N/A	N/A
Willers 1991	Case-control	Asthma	0	1	0	1	0	1	1	1	1	1	1	1	0	N/A	N/A
Yang 1998	Case-control	Asthma	0	1	0	1	1	1	1	0	0	0	1	0	0	N/A	N/A
Yang 1998	Case-control	Asthma	0	1	1	1	0	1	1	0	0	0	1	0	0	N/A	N/A
Youssef 2018	Case-control	Asthma	0	1	0	1	1	1	1	0	0	0	1	0	0	N/A	N/A
Zejda 2003	Prospective cohort	Asthma	0	0	0	1	1	1	1	1	0	1	1	1	0	N/A	N/A
Zheng 2002	Case-control	Asthma	1	1	0	1	0	1	1	0	0	0	1	1	0	N/A	N/A
Ahern 2009	Case-control	Breast cancer	0	1	N/A	N/A	0	1	0	0	0	N/A	0	1	N/A	N/A	N/A
Alberg 2004	Nested case-control	Breast cancer	0	1	N/A	N/A	0	1	0	0	0	N/A	1	1	N/A	N/A	N/A
Anderson 2012	Case-control	Breast cancer	0	1	N/A	N/A	1	1	1	0	0	N/A	0	1	N/A	N/A	N/A
Bonner 2005	Case-control	Breast cancer	1	1	N/A	N/A	1	0	0	0	0	N/A	0	1	N/A	N/A	N/A
Chaveepojnkamjorn 2017	Case-control	Breast cancer	0	1	N/A	N/A	1	1	1	1	1	N/A	1	0	N/A	N/A	N/A
De 2010	Case-control	Breast cancer	0	1	N/A	N/A	0	1	1	0	0	N/A	1	0	N/A	N/A	N/A
Dianatinasab 2017	Case-control	Breast cancer	0	1	N/A	N/A	0	1	0	0	0	N/A	1	0	N/A	N/A	N/A
Dossus 2014	Prospective cohort	Breast cancer	0	0	N/A	N/A	0	1	1	1	0	N/A	0	1	N/A	N/A	N/A

Table S20. Bias covariates tested in the primary model for each health outcome, by study

Author	Study design	Health outcome	Subpopulation	Not prosp. cohort	Out. self-reported	Exp. assessment	Potential selection bias	Adj. level 0	Adj. level 1	Adj. level 2	Not adj. smoking	Females only	Broad location exp.	Not current exp.	Asthma children	Hem. stroke	Isch. stroke
El-Sheikh 2021	Case-control	Breast cancer	0	1	N/A	N/A	1	1	1	0	0	N/A	1	0	N/A	N/A	N/A
Fararouei 2019	Case-control	Breast cancer	0	1	N/A	N/A	0	0	0	0	0	N/A	1	0	N/A	N/A	N/A
Fu 2015	Case-control	Breast cancer	0	1	N/A	N/A	1	1	1	1	1	N/A	1	0	N/A	N/A	N/A
Gao 2013	Case-control	Breast cancer	0	1	N/A	N/A	0	1	1	1	1	N/A	1	0	N/A	N/A	N/A
Hanaoka 2005	Prospective cohort	Breast cancer	1	0	N/A	N/A	0	1	0	0	0	N/A	1	1	N/A	N/A	N/A
Hirose 1995	Case-control	Breast cancer	0	1	N/A	N/A	0	1	1	0	0	N/A	1	1	N/A	N/A	N/A
Hosseinzadeh 2014	Case-control	Breast cancer	0	1	N/A	N/A	1	1	1	1	1	N/A	1	0	N/A	N/A	N/A
Hsieh 2014	Case-control	Breast cancer	0	1	N/A	N/A	1	1	1	1	1	N/A	1	1	N/A	N/A	N/A
Hu 2013	Case-control	Breast cancer	0	1	N/A	N/A	0	1	1	1	1	N/A	0	1	N/A	N/A	N/A
Ilic 2013	Case-control	Breast cancer	0	1	N/A	N/A	1	0	0	0	0	N/A	0	0	N/A	N/A	N/A
Jee 1999	Prospective cohort	Breast cancer	0	0	N/A	N/A	0	1	0	0	0	N/A	1	0	N/A	N/A	N/A
Johnson 2000	Case-control	Breast cancer	1	1	N/A	N/A	1	0	0	0	0	N/A	0	1	N/A	N/A	N/A
Kariri 2017	Case-control	Breast cancer	0	1	N/A	N/A	1	1	1	1	1	N/A	1	0	N/A	N/A	N/A
Lash 1999	Case-control	Breast cancer	0	1	N/A	N/A	1	1	0	0	0	N/A	1	1	N/A	N/A	N/A
Lash 2002	Case-control	Breast cancer	1	1	N/A	N/A	1	1	0	0	0	N/A	1	1	N/A	N/A	N/A
Li 2015	Case-control	Breast cancer	0	1	N/A	N/A	0	1	0	0	0	N/A	0	1	N/A	N/A	N/A

Table S20. Bias covariates tested in the primary model for each health outcome, by study

Author	Study design	Health outcome	Subpopulation	Not prosp. cohort	Out. self-reported	Exp. assessment	Potential selection bias	Adj. level 0	Adj. level 1	Adj. level 2	Not adj. smoking	Females only	Broad location exp.	Not current exp.	Asthma children	Hem. stroke	Isch. stroke
Lin 2008	Prospective cohort	Breast cancer	0	0	N/A	N/A	0	1	0	0	0	N/A	1	1	N/A	N/A	N/A
Liu 2000	Case-control	Breast cancer	0	1	N/A	N/A	1	1	1	0	0	N/A	0	1	N/A	N/A	N/A
Luo 2011	Prospective cohort	Breast cancer	0	0	N/A	N/A	0	0	0	0	0	N/A	0	1	N/A	N/A	N/A
Marzouk 2009	Case-control	Breast cancer	0	1	N/A	N/A	1	1	1	1	1	N/A	1	0	N/A	N/A	N/A
Metsola 2005	Case-control	Breast cancer	0	1	N/A	N/A	1	1	1	0	0	N/A	0	1	N/A	N/A	N/A
Millikan 1998	Case-control	Breast cancer	0	1	N/A	N/A	1	1	0	0	0	N/A	1	1	N/A	N/A	N/A
Morabia 1996	Case-control	Breast cancer	1	1	N/A	N/A	1	0	0	0	0	N/A	1	1	N/A	N/A	N/A
Niehoff 2017	Case-control	Breast cancer	0	1	N/A	N/A	1	1	1	1	1	N/A	1	1	N/A	N/A	N/A
Nishino 2001	Prospective cohort	Breast cancer	0	0	N/A	N/A	0	1	1	0	0	N/A	1	0	N/A	N/A	N/A
Nishino 2014	Case-control	Breast cancer	0	1	N/A	N/A	0	0	0	0	0	N/A	1	0	N/A	N/A	N/A
Pimhanam 2014	Case-control	Breast cancer	0	1	N/A	N/A	1	1	0	0	0	N/A	1	1	N/A	N/A	N/A
Pirie 2008	Prospective cohort	Breast cancer	0	0	N/A	N/A	1	1	0	0	0	N/A	1	1	N/A	N/A	N/A
Reynolds 2009	Prospective cohort	Breast cancer	0	0	N/A	N/A	0	0	0	0	0	N/A	0	1	N/A	N/A	N/A
Roddam 2007	Case-control	Breast cancer	0	1	N/A	N/A	0	1	0	0	0	N/A	1	1	N/A	N/A	N/A
Rollison 2008	Case-control	Breast cancer	1	1	N/A	N/A	1	1	0	0	0	N/A	0	1	N/A	N/A	N/A

Table S20. Bias covariates tested in the primary model for each health outcome, by study

Author	Study design	Health outcome	Subpopulation	Not prosp. cohort	Out. self-reported	Exp. assessment	Potential selection bias	Adj. level 0	Adj. level 1	Adj. level 2	Not adj. smoking	Females only	Broad location exp.	Not current exp.	Asthma children	Hem. stroke	Isch. stroke
Shrubsole 2004	Case-control	Breast cancer	0	1	N/A	N/A	0	1	0	0	0	N/A	1	1	N/A	N/A	N/A
Smith 1994	Case-control	Breast cancer	0	1	N/A	N/A	1	1	0	0	0	N/A	0	1	N/A	N/A	N/A
Strumylaite 2017	Case-control	Breast cancer	0	1	N/A	N/A	0	0	0	0	0	N/A	0	1	N/A	N/A	N/A
Tang 2013	Case-control	Breast cancer	0	1	N/A	N/A	1	0	0	0	0	N/A	0	1	N/A	N/A	N/A
Tong 2014	Case-control	Breast cancer	0	1	N/A	N/A	0	1	0	0	0	N/A	1	1	N/A	N/A	N/A
Wartenberg 2000	Prospective cohort	Breast cancer	0	0	N/A	N/A	0	0	0	0	0	N/A	1	0	N/A	N/A	N/A
White 2017	Prospective cohort	Breast cancer	0	0	N/A	N/A	0	0	0	0	0	N/A	1	1	N/A	N/A	N/A
Woo 2000	Nested case-control	Breast cancer	1	1	N/A	N/A	1	1	1	0	0	N/A	1	0	N/A	N/A	N/A
Xue 2011	Prospective cohort	Breast cancer	0	0	N/A	N/A	0	1	0	0	0	N/A	0	1	N/A	N/A	N/A
Yassin 2018	Case-control	Breast cancer	0	1	N/A	N/A	1	1	1	1	1	N/A	1	0	N/A	N/A	N/A
Zahali 2021	Case-control	Breast cancer	0	1	N/A	N/A	1	1	1	0	0	N/A	0	0	N/A	N/A	N/A
Zhao 1999	Case-control	Breast cancer	0	1	N/A	N/A	1	1	1	1	1	N/A	1	0	N/A	N/A	N/A
Behrendt 2005	Case-control	Chronic obstructive pulmonary disease	0	1	0	1	0	1	1	0	0	0	0	0	N/A	N/A	N/A

Table S20. Bias covariates tested in the primary model for each health outcome, by study

Author	Study design	Health outcome	Subpopulation	Not prosp. cohort	Out. self-reported	Exp. assessment	Potential selection bias	Adj. level 0	Adj. level 1	Adj. level 2	Not adj. smoking	Females only	Broad location exp.	Not current exp.	Asthma children	Hem. stroke	Isch. stroke
Chan-Yeung 2007	Case-control	Chronic obstructive pulmonary disease	0	1	0	1	1	1	1	1	1	0	0	0	N/A	N/A	N/A
Chen 2013	Case-control	Chronic obstructive pulmonary disease	1	1	0	1	0	1	0	0	0	0	1	0	N/A	N/A	N/A
David 2005	Prospective cohort	Chronic obstructive pulmonary disease	0	0	1	1	0	1	1	0	0	0	0	1	N/A	N/A	N/A
Dennis 1996	Case-control	Chronic obstructive pulmonary disease	0	1	0	1	1	1	1	1	1	0	1	0	N/A	N/A	N/A
Ding 2015	Case-control	Chronic obstructive pulmonary disease	1	1	0	1	0	1	1	1	1	1	1	0	N/A	N/A	N/A
Diver 2018	Prospective cohort	Chronic obstructive pulmonary disease	1	0	0	0	0	0	0	0	0	0	1	0	N/A	N/A	N/A
Enstrom 2003	Prospective cohort	Chronic obstructive pulmonary disease	0	0	0	0	0	0	0	0	0	0	1	0	N/A	N/A	N/A

Table S20. Bias covariates tested in the primary model for each health outcome, by study

Author	Study design	Health outcome	Subpopulation	Not prosp. cohort	Out. self-reported	Exp. assessment	Potential selection bias	Adj. level 0	Adj. level 1	Adj. level 2	Not adj. smoking	Females only	Broad location exp.	Not current exp.	Asthma children	Hem. stroke	Isch. stroke
Gerbase 2006	Prospective cohort	Chronic obstructive pulmonary disease	0	0	0	1	0	1	0	0	0	0	1	0	N/A	N/A	N/A
He 2012	Prospective cohort	Chronic obstructive pulmonary disease	0	0	0	1	0	0	0	0	0	0	0	0	N/A	N/A	N/A
Huang 2019	Case-control	Chronic obstructive pulmonary disease	0	1	0	1	0	0	0	0	0	0	1	0	N/A	N/A	N/A
Johannessen 2012	Case-control	Chronic obstructive pulmonary disease	0	1	0	1	0	1	0	0	0	0	0	0	N/A	N/A	N/A
Johannessen 2012	Case-control	Chronic obstructive pulmonary disease	0	1	0	1	0	1	1	1	1	0	0	0	N/A	N/A	N/A
Lee 1986	Case-control	Chronic obstructive pulmonary disease	0	1	0	1	0	1	1	0	0	0	1	1	N/A	N/A	N/A
McGhee 2005	Case-control	Chronic obstructive pulmonary disease	0	1	0	1	0	1	1	0	0	0	1	1	N/A	N/A	N/A

Table S20. Bias covariates tested in the primary model for each health outcome, by study

Author	Study design	Health outcome	Subpopulation	Not prosp. cohort	Out. self-reported	Exp. assessment	Potential selection bias	Adj. level 0	Adj. level 1	Adj. level 2	Not adj. smoking	Females only	Broad location exp.	Not current exp.	Asthma children	Hem. stroke	Isch. stroke
Pahwa 2019	Prospective cohort	Chronic obstructive pulmonary disease	0	0	1	1	1	1	0	0	0	0	1	0	N/A	N/A	N/A
Salama 2020	Case-control	Chronic obstructive pulmonary disease	0	1	0	1	1	1	1	0	0	0	1	1	N/A	N/A	N/A
Salameh 2012	Case-control	Chronic obstructive pulmonary disease	0	1	0	1	1	1	1	1	0	1	0	0	N/A	N/A	N/A
Sandler 1989	Prospective cohort	Chronic obstructive pulmonary disease	1	0	0	1	0	1	0	0	0	0	1	0	N/A	N/A	N/A
Vineis 2005	Nested case-control	Chronic obstructive pulmonary disease	0	1	0	1	0	1	1	0	0	0	0	1	N/A	N/A	N/A
Wu 2010	Case-control	Chronic obstructive pulmonary disease	1	1	0	1	1	1	0	0	0	0	0	1	N/A	N/A	N/A
Xu 2007	Nested case-control	Chronic obstructive pulmonary disease	1	1	0	1	0	1	1	1	1	0	1	1	N/A	N/A	N/A

Table S20. Bias covariates tested in the primary model for each health outcome, by study

Author	Study design	Health outcome	Subpopulation	Not prosp. cohort	Out. self-reported	Exp. assessment	Potential selection bias	Adj. level 0	Adj. level 1	Adj. level 2	Not adj. smoking	Females only	Broad location exp.	Not current exp.	Asthma children	Hem. stroke	Isch. stroke
Hayashino 2008	Prospective cohort	Diabetes mellitus type 2	0	0	0	0	1	N/A	N/A	N/A	N/A	N/A	0	0	N/A	N/A	N/A
Huang 2020	Prospective cohort	Diabetes mellitus type 2	0	0	0	1	0	N/A	N/A	N/A	N/A	N/A	1	0	N/A	N/A	N/A
Jiang 2019	Prospective cohort	Diabetes mellitus type 2	0	0	1	1	1	N/A	N/A	N/A	N/A	N/A	1	1	N/A	N/A	N/A
Kim 2021	Retrospective cohort	Diabetes mellitus type 2	0	1	0	1	0	N/A	N/A	N/A	N/A	N/A	0	0	N/A	N/A	N/A
Kowall 2010	Prospective cohort	Diabetes mellitus type 2	1	0	0	1	1	N/A	N/A	N/A	N/A	N/A	0	0	N/A	N/A	N/A
Oba 2020	Prospective cohort	Diabetes mellitus type 2	0	0	1	0	0	N/A	N/A	N/A	N/A	N/A	1	0	N/A	N/A	N/A
Rias 2020	Case-control	Diabetes mellitus type 2	0	1	0	1	0	N/A	N/A	N/A	N/A	N/A	1	1	N/A	N/A	N/A
Zeng 2022	Case-control	Diabetes mellitus type 2	1	1	0	1	0	N/A	N/A	N/A	N/A	N/A	1	0	N/A	N/A	N/A
Zhang 2011	Prospective cohort	Diabetes mellitus type 2	0	0	1	1	0	N/A	N/A	N/A	N/A	N/A	1	0	N/A	N/A	N/A

Table S20. Bias covariates tested in the primary model for each health outcome, by study

Author	Study design	Health outcome	Subpopulation	Not prosp. cohort	Out. self-reported	Exp. assessment	Potential selection bias	Adj. level 0	Adj. level 1	Adj. level 2	Not adj. smoking	Females only	Broad location exp.	Not current exp.	Asthma children	Hem. stroke	Isch. stroke
Attard 2017	Case-control	Ischemic heart disease	1	1	N/A	1	0	0	0	0	0	N/A	1	1	N/A	N/A	N/A
Awawdi 2016	Case-control	Ischemic heart disease	0	1	N/A	1	0	1	1	0	0	N/A	1	1	N/A	N/A	N/A
Ciruzzi 1998	Case-control	Ischemic heart disease	0	1	N/A	1	0	0	0	0	0	N/A	1	0	N/A	N/A	N/A
Clark 2013	Prospective cohort	Ischemic heart disease	0	0	N/A	1	0	1	0	0	0	N/A	0	1	N/A	N/A	N/A
Ding 2009	Case-control	Ischemic heart disease	0	1	N/A	1	0	0	0	0	0	N/A	1	1	N/A	N/A	N/A
Diver 2018	Prospective cohort	Ischemic heart disease	1	0	N/A	0	0	0	0	0	0	N/A	1	0	N/A	N/A	N/A
Dobson 1991	Case-control	Ischemic heart disease	1	1	N/A	1	1	1	1	0	0	N/A	0	0	N/A	N/A	N/A
Enstrom 2003	Prospective cohort	Ischemic heart disease	0	0	N/A	0	0	0	0	0	0	N/A	1	0	N/A	N/A	N/A
Fatmi 2014	Case-control	Ischemic heart disease	0	1	N/A	1	0	1	1	1	1	N/A	1	1	N/A	N/A	N/A
Gallo 2010	Prospective cohort	Ischemic heart disease	0	0	N/A	1	1	1	0	0	0	N/A	1	0	N/A	N/A	N/A
Garland 1985	Prospective cohort	Ischemic heart disease	0	0	N/A	1	0	1	1	1	0	N/A	1	0	N/A	N/A	N/A
He 1994	Case-control	Ischemic heart disease	0	1	N/A	1	0	0	0	0	0	N/A	0	1	N/A	N/A	N/A

Table S20. Bias covariates tested in the primary model for each health outcome, by study

Author	Study design	Health outcome	Subpopulation	Not prosp. cohort	Out. self-reported	Exp. assessment	Potential selection bias	Adj. level 0	Adj. level 1	Adj. level 2	Not adj. smoking	Females only	Broad location exp.	Not current exp.	Asthma children	Hem. stroke	Isch. stroke
He 2012	Prospective cohort	Ischemic heart disease	0	0	N/A	1	0	0	0	0	0	N/A	0	1	N/A	N/A	N/A
Hill 2007	Prospective cohort	Ischemic heart disease	0	0	N/A	1	0	1	0	0	0	N/A	1	0	N/A	N/A	N/A
Hole 1989	Prospective cohort	Ischemic heart disease	1	0	N/A	1	0	1	1	0	0	N/A	1	0	N/A	N/A	N/A
Janghorbani 1997	Case-control	Ischemic heart disease	0	1	N/A	1	0	1	1	0	0	N/A	1	1	N/A	N/A	N/A
Kastorini 2013	Case-control	Ischemic heart disease	0	1	N/A	1	0	0	0	0	0	N/A	1	0	N/A	N/A	N/A
Kawachi 1997	Prospective cohort	Ischemic heart disease	0	0	N/A	1	0	0	0	0	0	N/A	0	0	N/A	N/A	N/A
Kobayashi 2022	Prospective cohort	Ischemic heart disease	0	0	N/A	1	1	0	0	0	0	N/A	1	0	N/A	N/A	N/A
La 1993	Nested case-control	Ischemic heart disease	0	1	N/A	1	0	0	0	0	0	N/A	1	0	N/A	N/A	N/A
Lee 1986	Case-control	Ischemic heart disease	0	1	N/A	1	0	1	1	1	0	N/A	1	1	N/A	N/A	N/A
Malinauskiene 2011	Case-control	Ischemic heart disease	0	1	N/A	1	1	1	0	0	0	N/A	1	0	N/A	N/A	N/A
McElduff 1998	Case-control	Ischemic heart disease	1	1	N/A	1	1	1	1	1	0	N/A	0	0	N/A	N/A	N/A
McElduff 1998	Case-control	Ischemic heart disease	1	1	N/A	1	0	1	1	1	0	N/A	0	0	N/A	N/A	N/A

Table S20. Bias covariates tested in the primary model for each health outcome, by study

Author	Study design	Health outcome	Subpopulation	Not prosp. cohort	Out. self-reported	Exp. assessment	Potential selection bias	Adj. level 0	Adj. level 1	Adj. level 2	Not adj. smoking	Females only	Broad location exp.	Not current exp.	Asthma children	Hem. stroke	Isch. stroke
McGhee 2005	Case-control	Ischemic heart disease	0	1	N/A	1	0	1	1	0	0	N/A	1	1	N/A	N/A	N/A
Muscat 1995	Case-control	Ischemic heart disease	0	1	N/A	1	0	1	0	0	0	N/A	1	0	N/A	N/A	N/A
Nishtar 2004	Case-control	Ischemic heart disease	0	1	N/A	1	1	1	1	1	1	N/A	1	0	N/A	N/A	N/A
Notara 2015	Prospective cohort	Ischemic heart disease	0	0	N/A	1	0	0	0	0	0	N/A	0	0	N/A	N/A	N/A
Pitsavos 2002	Case-control	Ischemic heart disease	1	1	N/A	1	0	0	0	0	0	N/A	0	1	N/A	N/A	N/A
Rashid 2019	Case-control	Ischemic heart disease	0	1	N/A	1	1	1	1	1	1	N/A	0	0	N/A	N/A	N/A
Rosenlund 2001	Case-control	Ischemic heart disease	1	1	N/A	1	1	0	0	0	0	N/A	0	0	N/A	N/A	N/A
Rossi 2011	Case-control	Ischemic heart disease	0	1	N/A	1	0	1	1	0	0	N/A	1	0	N/A	N/A	N/A
Sadeghi 2020	Prospective cohort	Ischemic heart disease	0	0	N/A	1	0	1	0	0	0	N/A	0	1	N/A	N/A	N/A
Sandler 1989	Prospective cohort	Ischemic heart disease	1	0	N/A	1	0	1	0	0	0	N/A	1	0	N/A	N/A	N/A
Spencer 1999	Case-control	Ischemic heart disease	1	1	N/A	1	1	1	1	1	1	N/A	0	1	N/A	N/A	N/A
Steenland 1996	Prospective cohort	Ischemic heart disease	0	0	N/A	1	0	0	0	0	0	N/A	1	0	N/A	N/A	N/A

Table S20. Bias covariates tested in the primary model for each health outcome, by study

Author	Study design	Health outcome	Subpopulation	Not prosp. cohort	Out. self-reported	Exp. assessment	Potential selection bias	Adj. level 0	Adj. level 1	Adj. level 2	Not adj. smoking	Females only	Broad location exp.	Not current exp.	Asthma children	Hem. stroke	Isch. stroke
Sulo 2008	Case-control	Ischemic heart disease	1	1	N/A	1	1	0	0	0	0	N/A	1	0	N/A	N/A	N/A
Svendsen 1987	Prospective cohort	Ischemic heart disease	0	0	N/A	1	0	0	0	0	0	N/A	1	0	N/A	N/A	N/A
Almirall 2014	Case-control	Lower respiratory infections	1	1	0	1	1	N/A	1	0	N/A	N/A	N/A	0	1	N/A	N/A
Arlington 2019	Prospective cohort	Lower respiratory infections	0	0	1	1	0	N/A	1	0	N/A	N/A	N/A	0	0	N/A	N/A
Baker 2006	Prospective cohort	Lower respiratory infections	0	0	0	1	0	N/A	1	0	N/A	N/A	N/A	0	0	N/A	N/A
Barsam 2013	Case-control	Lower respiratory infections	0	1	0	1	1	N/A	1	1	N/A	N/A	N/A	0	0	N/A	N/A
Behrooz 2018	Case-control	Lower respiratory infections	0	1	0	1	0	N/A	1	0	N/A	N/A	N/A	1	0	N/A	N/A
Bermúdez 2021	Prospective cohort	Lower respiratory infections	0	0	0	1	0	N/A	1	1	N/A	N/A	N/A	1	0	N/A	N/A
Broor 2001	Case-control	Lower respiratory infections	0	1	0	1	1	N/A	1	1	N/A	N/A	N/A	0	0	N/A	N/A

Table S20. Bias covariates tested in the primary model for each health outcome, by study

Author	Study design	Health outcome	Subpopulation	Not prosp. cohort	Out. self-reported	Exp. assessment	Potential selection bias	Adj. level 0	Adj. level 1	Adj. level 2	Not adj. smoking	Females only	Broad location exp.	Not current exp.	Asthma children	Hem. stroke	Isch. stroke
Broughton 2005	Prospective cohort	Lower respiratory infections	0	0	0	1	1	N/A	1	1	N/A	N/A	N/A	0	0	N/A	N/A
Charoenca 2013	Case-control	Lower respiratory infections	1	1	0	1	1	N/A	0	0	N/A	N/A	N/A	1	0	N/A	N/A
Colley 1974	Prospective cohort	Lower respiratory infections	0	0	0	0	0	N/A	1	1	N/A	N/A	N/A	0	0	N/A	N/A
Dina 2021	Case-control	Lower respiratory infections	0	1	0	1	1	N/A	1	0	N/A	N/A	N/A	0	0	N/A	N/A
Duijts 2008	Prospective cohort	Lower respiratory infections	1	0	1	1	1	N/A	1	1	N/A	N/A	N/A	0	0	N/A	N/A
Dwedar 2018	Case-control	Lower respiratory infections	0	1	0	1	1	N/A	1	0	N/A	N/A	N/A	0	1	N/A	N/A
Farr 2000	Case-control	Lower respiratory infections	1	1	0	1	1	N/A	1	1	N/A	N/A	N/A	1	1	N/A	N/A
Farzana 2017	Case-control	Lower respiratory infections	0	1	0	1	1	N/A	1	0	N/A	N/A	N/A	0	0	N/A	N/A
Fergusson 1985	Prospective cohort	Lower respiratory infections	1	0	0	0	0	N/A	1	1	N/A	N/A	N/A	0	0	N/A	N/A

Table S20. Bias covariates tested in the primary model for each health outcome, by study

Author	Study design	Health outcome	Subpopulation	Not prosp. cohort	Out. self-reported	Exp. assessment	Potential selection bias	Adj. level 0	Adj. level 1	Adj. level 2	Not adj. smoking	Females only	Broad location exp.	Not current exp.	Asthma children	Hem. stroke	Isch. stroke
Frassanito 2022	Case-control	Lower respiratory infections	0	1	0	1	1	N/A	1	0	N/A	N/A	N/A	1	0	N/A	N/A
Fuentes-Leonarte 2015	Prospective cohort	Lower respiratory infections	0	0	1	1	0	N/A	1	0	N/A	N/A	N/A	0	0	N/A	N/A
Goetghebuer 2004	Case-control	Lower respiratory infections	0	1	0	1	1	N/A	1	1	N/A	N/A	N/A	0	0	N/A	N/A
Grant 2012	Case-control	Lower respiratory infections	1	1	0	1	0	N/A	1	1	N/A	N/A	N/A	0	0	N/A	N/A
Hassan 2001	Case-control	Lower respiratory infections	0	1	0	1	1	N/A	1	1	N/A	N/A	N/A	0	0	N/A	N/A
Håberg 2007	Prospective cohort	Lower respiratory infections	1	0	1	0	1	N/A	1	0	N/A	N/A	N/A	0	0	N/A	N/A
Islam 2021	Case-control	Lower respiratory infections	0	1	0	1	0	N/A	1	1	N/A	N/A	N/A	0	0	N/A	N/A
Johnson 1992	Case-control	Lower respiratory infections	0	1	0	1	1	N/A	1	1	N/A	N/A	N/A	0	0	N/A	N/A
Keskinoglu 2007	Case-control	Lower respiratory infections	0	1	0	1	1	N/A	1	0	N/A	N/A	N/A	0	0	N/A	N/A

Table S20. Bias covariates tested in the primary model for each health outcome, by study

Author	Study design	Health outcome	Subpopulation	Not prosp. cohort	Out. self-reported	Exp. assessment	Potential selection bias	Adj. level 0	Adj. level 1	Adj. level 2	Not adj. smoking	Females only	Broad location exp.	Not current exp.	Asthma children	Hem. stroke	Isch. stroke
Koch 2003	Prospective cohort	Lower respiratory infections	1	0	0	1	0	N/A	1	0	N/A	N/A	N/A	0	0	N/A	N/A
Lanari 2015	Prospective cohort	Lower respiratory infections	1	0	0	0	0	N/A	1	0	N/A	N/A	N/A	1	0	N/A	N/A
Liu 2022	Retrospective cohort	Lower respiratory infections	1	1	0	1	1	N/A	1	1	N/A	N/A	N/A	0	0	N/A	N/A
Liyanage 2021	Case-control	Lower respiratory infections	0	1	0	1	0	N/A	1	0	N/A	N/A	N/A	0	0	N/A	N/A
Loeb 2009	Case-control	Lower respiratory infections	0	1	0	1	1	N/A	1	1	N/A	N/A	N/A	0	1	N/A	N/A
Marbury 1996	Prospective cohort	Lower respiratory infections	0	0	0	0	1	N/A	1	1	N/A	N/A	N/A	1	0	N/A	N/A
McConnochie 1986	Case-control	Lower respiratory infections	0	1	0	1	0	N/A	1	0	N/A	N/A	N/A	1	0	N/A	N/A
Miyahara 2017	Prospective cohort	Lower respiratory infections	0	0	0	1	0	N/A	0	0	N/A	N/A	N/A	0	0	N/A	N/A
Nenna 2017	Case-control	Lower respiratory infections	0	1	0	1	0	N/A	1	1	N/A	N/A	N/A	0	0	N/A	N/A

Table S20. Bias covariates tested in the primary model for each health outcome, by study

Author	Study design	Health outcome	Subpopulation	Not prosp. cohort	Out. self-reported	Exp. assessment	Potential selection bias	Adj. level 0	Adj. level 1	Adj. level 2	Not adj. smoking	Females only	Broad location exp.	Not current exp.	Asthma children	Hem. stroke	Isch. stroke
Nuesslein 1999	Prospective cohort	Lower respiratory infections	1	0	1	0	0	N/A	1	0	N/A	N/A	N/A	1	0	N/A	N/A
Pullan 1982	Case-control	Lower respiratory infections	0	1	0	1	0	N/A	1	0	N/A	N/A	N/A	0	0	N/A	N/A
Ramesh 2012	Case-control	Lower respiratory infections	1	1	0	1	1	N/A	1	1	N/A	N/A	N/A	0	0	N/A	N/A
Robledo-Aceves 2018	Case-control	Lower respiratory infections	0	1	0	1	1	N/A	1	0	N/A	N/A	N/A	1	0	N/A	N/A
Roda 2011	Prospective cohort	Lower respiratory infections	0	0	1	0	1	N/A	1	1	N/A	N/A	N/A	0	0	N/A	N/A
Roux 2015	Prospective cohort	Lower respiratory infections	0	0	0	1	0	N/A	1	0	N/A	N/A	N/A	0	0	N/A	N/A
Rylander 1995	Case-control	Lower respiratory infections	1	1	0	1	0	N/A	1	1	N/A	N/A	N/A	0	0	N/A	N/A
Schulte-Hobein 1992	Prospective cohort	Lower respiratory infections	0	0	1	0	1	N/A	1	1	N/A	N/A	N/A	0	0	N/A	N/A
Taylor 1987	Prospective cohort	Lower respiratory infections	1	0	1	1	1	N/A	1	1	N/A	N/A	N/A	0	0	N/A	N/A

Table S20. Bias covariates tested in the primary model for each health outcome, by study

Author	Study design	Health outcome	Subpopulation	Not prosp. cohort	Out. self-reported	Exp. assessment	Potential selection bias	Adj. level 0	Adj. level 1	Adj. level 2	Not adj. smoking	Females only	Broad location exp.	Not current exp.	Asthma children	Hem. stroke	Isch. stroke
Tupasi 1990	Prospective cohort	Lower respiratory infections	1	0	0	1	1	N/A	1	0	N/A	N/A	N/A	0	0	N/A	N/A
Törmänen 2018	Case-control	Lower respiratory infections	0	1	0	1	0	N/A	1	0	N/A	N/A	N/A	1	0	N/A	N/A
Verani 2016	Case-control	Lower respiratory infections	0	1	0	1	1	N/A	1	0	N/A	N/A	N/A	0	0	N/A	N/A
Victora 1994	Case-control	Lower respiratory infections	1	1	0	1	0	N/A	1	1	N/A	N/A	N/A	0	0	N/A	N/A
Wenten 2005	Prospective cohort	Lower respiratory infections	0	0	1	1	1	N/A	0	0	N/A	N/A	N/A	0	0	N/A	N/A
Wright 1991	Prospective cohort	Lower respiratory infections	0	0	0	1	0	N/A	1	1	N/A	N/A	N/A	1	0	N/A	N/A
Adair-Bischoff 1998	Case-control	Otitis media	1	1	0	1	1	N/A	N/A	1	N/A	N/A	N/A	N/A	0	N/A	N/A
Alho 1993	Prospective cohort	Otitis media	1	0	0	1	0	N/A	N/A	1	N/A	N/A	N/A	N/A	0	N/A	N/A
Bentdal 2007	Prospective cohort	Otitis media	1	0	1	0	1	N/A	N/A	0	N/A	N/A	N/A	N/A	0	N/A	N/A
Clamp 2020	Prospective cohort	Otitis media	0	0	0	0	1	N/A	N/A	1	N/A	N/A	N/A	N/A	0	N/A	N/A

Table S20. Bias covariates tested in the primary model for each health outcome, by study

Author	Study design	Health outcome	Subpopulation	Not prosp. cohort	Out. self-reported	Exp. assessment	Potential selection bias	Adj. level 0	Adj. level 1	Adj. level 2	Not adj. smoking	Females only	Broad location exp.	Not current exp.	Asthma children	Hem. stroke	Isch. stroke
Collet 1995	Prospective cohort	Otitis media	1	0	1	0	1	N/A	N/A	0	N/A	N/A	N/A	N/A	0	N/A	N/A
Costa 2004	Case-control	Otitis media	1	1	0	1	0	N/A	N/A	0	N/A	N/A	N/A	N/A	0	N/A	N/A
Daigler 1991	Case-control	Otitis media	0	1	0	1	1	N/A	N/A	1	N/A	N/A	N/A	N/A	1	N/A	N/A
Daly 1999	Prospective cohort	Otitis media	0	0	0	0	0	N/A	N/A	1	N/A	N/A	N/A	N/A	0	N/A	N/A
Daly 2007	Prospective cohort	Otitis media	0	0	0	0	0	N/A	N/A	1	N/A	N/A	N/A	N/A	0	N/A	N/A
Ey 1995	Prospective cohort	Otitis media	1	0	0	1	0	N/A	N/A	1	N/A	N/A	N/A	N/A	0	N/A	N/A
Fuentes-Leonarte 2015	Prospective cohort	Otitis media	0	0	0	1	0	N/A	N/A	0	N/A	N/A	N/A	N/A	0	N/A	N/A
Håberg 2010	Prospective cohort	Otitis media	1	0	1	0	0	N/A	N/A	0	N/A	N/A	N/A	N/A	0	N/A	N/A
Jensen 2013	Prospective cohort	Otitis media	1	0	0	1	1	N/A	N/A	0	N/A	N/A	N/A	N/A	0	N/A	N/A
Koch 2011	Prospective cohort	Otitis media	1	0	0	1	0	N/A	N/A	0	N/A	N/A	N/A	N/A	0	N/A	N/A
Niclasen 2016	Prospective cohort	Otitis media	0	0	1	1	0	N/A	N/A	1	N/A	N/A	N/A	N/A	0	N/A	N/A
Prins-van 2017	Prospective cohort	Otitis media	0	0	1	1	0	N/A	N/A	0	N/A	N/A	N/A	N/A	0	N/A	N/A
Pukander 1985	Case-control	Otitis media	0	1	0	1	0	N/A	N/A	1	N/A	N/A	N/A	N/A	0	N/A	N/A

Table S20. Bias covariates tested in the primary model for each health outcome, by study

Author	Study design	Health outcome	Subpopulation	Not prosp. cohort	Out. self-reported	Exp. assessment	Potential selection bias	Adj. level 0	Adj. level 1	Adj. level 2	Not adj. smoking	Females only	Broad location exp.	Not current exp.	Asthma children	Hem. stroke	Isch. stroke
Samson 2020	Prospective cohort	Otitis media	0	0	0	1	1	N/A	N/A	0	N/A	N/A	N/A	N/A	0	N/A	N/A
Stenstrom 1993	Case-control	Otitis media	0	1	0	1	0	N/A	N/A	0	N/A	N/A	N/A	N/A	0	N/A	N/A
Ståhlberg 1986	Case-control	Otitis media	1	1	0	1	0	N/A	N/A	1	N/A	N/A	N/A	N/A	0	N/A	N/A
Tainio 1988	Prospective cohort	Otitis media	0	0	0	0	0	N/A	N/A	1	N/A	N/A	N/A	N/A	0	N/A	N/A
Teele 1989	Prospective cohort	Otitis media	0	0	0	1	1	N/A	N/A	1	N/A	N/A	N/A	N/A	0	N/A	N/A
Wijayanti 2021	Case-control	Otitis media	0	1	0	1	1	N/A	N/A	1	N/A	N/A	N/A	N/A	0	N/A	N/A
Yang 1999	Case-control	Otitis media	0	1	1	1	0	N/A	N/A	0	N/A	N/A	N/A	N/A	0	N/A	N/A
Anderson 2004	Case-control	Stroke	1	1	0	1	1	1	1	0	N/A	0	1	0	N/A	1	0
Bonita 1999	Case-control	Stroke	1	1	0	1	0	1	0	0	N/A	0	0	1	N/A	0	0
Diver 2018	Prospective cohort	Stroke	1	0	0	0	0	0	0	0	N/A	0	1	0	N/A	0	0
Gallo 2010	Prospective cohort	Stroke	0	0	0	1	1	1	0	0	N/A	0	0	0	N/A	0	0
Glymour 2008	Prospective cohort	Stroke	0	0	1	1	0	0	0	0	N/A	0	1	0	N/A	0	0
He 2012	Prospective cohort	Stroke	0	0	0	1	0	0	0	0	N/A	0	0	1	N/A	0	0

Table S20. Bias covariates tested in the primary model for each health outcome, by study

Author	Study design	Health outcome	Subpopulation	Not prosp. cohort	Out. self-reported	Exp. assessment	Potential selection bias	Adj. level 0	Adj. level 1	Adj. level 2	Not adj. smoking	Females only	Broad location exp.	Not current exp.	Asthma children	Hem. stroke	Isch. stroke
Hill 2007	Prospective cohort	Stroke	0	0	0	1	0	1	1	0	N/A	0	1	0	N/A	0	0
Hill 2007	Prospective cohort	Stroke	0	0	0	1	0	1	0	0	N/A	0	1	0	N/A	0	0
Hou 2017	Case-control	Stroke	0	1	0	1	1	1	0	0	N/A	0	1	1	N/A	0	0
Kastorini 2013	Case-control	Stroke	1	1	0	1	0	0	0	0	N/A	0	1	0	N/A	0	1
Kobayashi 2022	Prospective cohort	Stroke	0	0	0	1	1	0	0	0	N/A	0	1	0	N/A	0	0
Lee 1986	Case-control	Stroke	0	1	0	1	0	1	1	1	N/A	1	1	1	N/A	0	0
Malek 2015	Prospective cohort	Stroke	0	0	0	1	0	0	0	0	N/A	0	1	0	N/A	0	0
McGhee 2005	Case-control	Stroke	0	1	0	1	0	1	1	0	N/A	0	1	1	N/A	0	0
Nishino 2014	Prospective cohort	Stroke	0	0	0	1	0	0	0	0	N/A	0	1	0	N/A	0	0
Poulsen 2021	Case-cohort	Stroke	0	1	0	1	0	1	1	1	N/A	1	0	1	N/A	0	0
Qureshi 2005	Prospective cohort	Stroke	1	0	0	1	0	0	0	0	N/A	0	1	0	N/A	0	0
Sandler 1989	Prospective cohort	Stroke	1	0	0	1	1	1	0	0	N/A	0	1	0	N/A	0	0
Wen 2006	Prospective cohort	Stroke	1	0	0	0	0	0	0	0	N/A	0	1	0	N/A	0	0

Table S20. Bias covariates tested in the primary model for each health outcome, by study

Author	Study design	Health outcome	Subpopulation	Not prosp. cohort	Out. self-reported	Exp. assessment	Potential selection bias	Adj. level 0	Adj. level 1	Adj. level 2	Not adj. smoking	Females only	Broad location exp.	Not current exp.	Asthma children	Hem. stroke	Isch. stroke
Yamada 2003	Prospective cohort	Stroke	1	0	0	1	0	1	1	1	N/A	0	1	1	N/A	1	0
You 1999	Case-control	Stroke	1	1	0	1	0	1	0	0	N/A	0	1	1	N/A	0	1
Abdel-Rahman 2020	Prospective cohort	Tracheal, bronchus, and lung cancer	0	0	N/A	1	1	1	1	0	0	0	0	1	N/A	N/A	N/A
Al-Zoughool 2013	Case-control	Tracheal, bronchus, and lung cancer	0	1	N/A	1	1	1	1	0	0	0	1	1	N/A	N/A	N/A
Asomaning 2008	Case-control	Tracheal, bronchus, and lung cancer	0	1	N/A	1	0	1	1	1	0	1	0	0	N/A	N/A	N/A
Behera 2005	Case-control	Tracheal, bronchus, and lung cancer	0	1	N/A	1	1	1	1	1	0	0	1	1	N/A	N/A	N/A
Boffetta 1998	Case-control	Tracheal, bronchus, and lung cancer	0	1	N/A	1	1	1	1	0	0	0	0	1	N/A	N/A	N/A
Boffetta 1999	Case-control	Tracheal, bronchus, and lung cancer	0	1	N/A	1	0	1	1	0	0	0	0	1	N/A	N/A	N/A
Brownson 1992	Case-control	Tracheal, bronchus, and lung cancer	0	1	N/A	1	0	1	1	0	0	0	1	1	N/A	N/A	N/A
Bräuner 2012	Prospective cohort	Tracheal, bronchus, and lung cancer	0	0	N/A	1	0	1	1	1	1	1	0	0	N/A	N/A	N/A

Table S20. Bias covariates tested in the primary model for each health outcome, by study

Author	Study design	Health outcome	Subpopulation	Not prosp. cohort	Out. self-reported	Exp. assessment	Potential selection bias	Adj. level 0	Adj. level 1	Adj. level 2	Not adj. smoking	Females only	Broad location exp.	Not current exp.	Asthma children	Hem. stroke	Isch. stroke
Cardenas 1997	Prospective cohort	Tracheal, bronchus, and lung cancer	0	0	N/A	1	0	0	0	0	0	0	1	0	N/A	N/A	N/A
Cassidy 2006	Case-control	Tracheal, bronchus, and lung cancer	1	1	N/A	1	1	1	1	1	1	0	0	1	N/A	N/A	N/A
Chan-Yeung 2003	Case-control	Tracheal, bronchus, and lung cancer	0	1	N/A	1	1	1	0	0	0	0	0	0	N/A	N/A	N/A
Chen 2008	Case-control	Tracheal, bronchus, and lung cancer	0	1	N/A	1	1	1	1	1	1	1	0	0	N/A	N/A	N/A
Cheng 2022	Prospective cohort	Tracheal, bronchus, and lung cancer	0	0	N/A	1	0	0	0	0	0	0	1	0	N/A	N/A	N/A
Consonni 2018	Case-control	Tracheal, bronchus, and lung cancer	1	1	N/A	1	0	1	1	1	1	0	0	1	N/A	N/A	N/A
Dalager 1986	Case-control	Tracheal, bronchus, and lung cancer	0	1	N/A	1	0	1	1	0	0	0	1	1	N/A	N/A	N/A
Davis 2018	Case-control	Tracheal, bronchus, and lung cancer	0	1	N/A	1	1	1	1	0	0	0	0	1	N/A	N/A	N/A
Du 1995	Case-control	Tracheal, bronchus, and lung cancer	0	1	N/A	1	1	1	0	0	0	0	1	1	N/A	N/A	N/A

Table S20. Bias covariates tested in the primary model for each health outcome, by study

Author	Study design	Health outcome	Subpopulation	Not prosp. cohort	Out. self-reported	Exp. assessment	Potential selection bias	Adj. level 0	Adj. level 1	Adj. level 2	Not adj. smoking	Females only	Broad location exp.	Not current exp.	Asthma children	Hem. stroke	Isch. stroke
Enstrom 2003	Prospective cohort	Tracheal, bronchus, and lung cancer	0	0	N/A	0	0	0	0	0	0	0	1	0	N/A	N/A	N/A
Ferreccio 2013	Case-control	Tracheal, bronchus, and lung cancer	1	1	N/A	1	1	1	1	1	0	1	1	1	N/A	N/A	N/A
Fontham 1994	Case-control	Tracheal, bronchus, and lung cancer	1	1	N/A	1	1	0	0	0	0	0	0	1	N/A	N/A	N/A
Franco-Marina 2006	Case-control	Tracheal, bronchus, and lung cancer	1	1	N/A	1	1	1	1	0	0	0	1	1	N/A	N/A	N/A
Galeone 2008	Case-control	Tracheal, bronchus, and lung cancer	0	1	N/A	1	1	1	1	0	0	0	0	1	N/A	N/A	N/A
Gallegos-Arreola 2008	Case-control	Tracheal, bronchus, and lung cancer	0	1	N/A	1	1	1	1	1	0	1	1	0	N/A	N/A	N/A
Gao 1987	Case-control	Tracheal, bronchus, and lung cancer	1	1	N/A	1	0	1	1	0	0	0	1	1	N/A	N/A	N/A
Garfinkel 1985	Case-control	Tracheal, bronchus, and lung cancer	0	1	N/A	1	1	1	1	0	0	0	1	1	N/A	N/A	N/A
Ger 1993	Case-control	Tracheal, bronchus, and lung cancer	1	1	N/A	1	1	1	1	1	1	0	0	1	N/A	N/A	N/A

Table S20. Bias covariates tested in the primary model for each health outcome, by study

Author	Study design	Health outcome	Subpopulation	Not prosp. cohort	Out. self-reported	Exp. assessment	Potential selection bias	Adj. level 0	Adj. level 1	Adj. level 2	Not adj. smoking	Females only	Broad location exp.	Not current exp.	Asthma children	Hem. stroke	Isch. stroke
Gorlova 2008	Case-control	Tracheal, bronchus, and lung cancer	0	1	N/A	1	1	1	1	0	0	0	0	1	N/A	N/A	N/A
Han 2017	Case-control	Tracheal, bronchus, and lung cancer	0	1	N/A	1	1	1	1	0	0	0	0	0	N/A	N/A	N/A
Hansen 2021	Prospective cohort	Tracheal, bronchus, and lung cancer	1	0	N/A	1	1	1	0	0	0	0	1	1	N/A	N/A	N/A
He 2012	Prospective cohort	Tracheal, bronchus, and lung cancer	0	0	N/A	1	0	0	0	0	0	0	0	1	N/A	N/A	N/A
He 2013	Case-control	Tracheal, bronchus, and lung cancer	1	1	N/A	1	0	1	1	1	1	0	0	0	N/A	N/A	N/A
He 2017	Case-control	Tracheal, bronchus, and lung cancer	0	1	N/A	1	1	1	1	1	1	0	0	0	N/A	N/A	N/A
Hernández-Garduño 2004	Case-control	Tracheal, bronchus, and lung cancer	0	1	N/A	1	1	1	1	1	0	0	1	0	N/A	N/A	N/A
Hill 2007	Prospective cohort	Tracheal, bronchus, and lung cancer	0	0	N/A	1	0	0	0	0	0	0	1	0	N/A	N/A	N/A
Hirayama 1984	Prospective cohort	Tracheal, bronchus, and lung cancer	0	0	N/A	1	1	1	1	0	0	0	1	0	N/A	N/A	N/A

Table S20. Bias covariates tested in the primary model for each health outcome, by study

Author	Study design	Health outcome	Subpopulation	Not prosp. cohort	Out. self-reported	Exp. assessment	Potential selection bias	Adj. level 0	Adj. level 1	Adj. level 2	Not adj. smoking	Females only	Broad location exp.	Not current exp.	Asthma children	Hem. stroke	Isch. stroke
Hole 1989	Prospective cohort	Tracheal, bronchus, and lung cancer	1	0	N/A	1	0	1	1	0	0	0	1	0	N/A	N/A	N/A
Humble 1987	Case-control	Tracheal, bronchus, and lung cancer	0	1	N/A	1	0	1	1	0	0	0	1	1	N/A	N/A	N/A
Janerich 1990	Case-control	Tracheal, bronchus, and lung cancer	0	1	N/A	1	0	1	1	0	0	0	1	1	N/A	N/A	N/A
Jee 1999	Prospective cohort	Tracheal, bronchus, and lung cancer	0	0	N/A	1	0	1	0	0	0	0	1	0	N/A	N/A	N/A
Jin 2014	Case-control	Tracheal, bronchus, and lung cancer	1	1	N/A	1	1	0	0	0	0	0	1	0	N/A	N/A	N/A
Johnson 2001	Case-control	Tracheal, bronchus, and lung cancer	0	1	N/A	1	1	1	0	0	0	0	0	1	N/A	N/A	N/A
Jöckel 1998	Case-control	Tracheal, bronchus, and lung cancer	0	1	N/A	1	1	1	1	0	0	0	1	1	N/A	N/A	N/A
Kabat 1984	Case-control	Tracheal, bronchus, and lung cancer	0	1	N/A	1	1	1	1	0	0	0	0	0	N/A	N/A	N/A
Kabat 1995	Case-control	Tracheal, bronchus, and lung cancer	0	1	N/A	1	1	1	0	0	0	0	1	1	N/A	N/A	N/A

Table S20. Bias covariates tested in the primary model for each health outcome, by study

Author	Study design	Health outcome	Subpopulation	Not prosp. cohort	Out. self-reported	Exp. assessment	Potential selection bias	Adj. level 0	Adj. level 1	Adj. level 2	Not adj. smoking	Females only	Broad location exp.	Not current exp.	Asthma children	Hem. stroke	Isch. stroke
Kabat 1996	Case-control	Tracheal, bronchus, and lung cancer	0	1	N/A	1	0	1	0	0	0	0	1	1	N/A	N/A	N/A
Kalandidi 1990	Case-control	Tracheal, bronchus, and lung cancer	0	1	N/A	1	0	1	0	0	0	0	1	1	N/A	N/A	N/A
Koo 1987	Case-control	Tracheal, bronchus, and lung cancer	0	1	N/A	1	0	1	0	0	0	0	1	1	N/A	N/A	N/A
Kurahashi 2008	Prospective cohort	Tracheal, bronchus, and lung cancer	0	0	N/A	1	0	1	0	0	0	0	0	0	N/A	N/A	N/A
Lam 1987	Case-control	Tracheal, bronchus, and lung cancer	0	1	N/A	1	0	1	1	0	0	0	1	1	N/A	N/A	N/A
Lan 2008	Case-control	Tracheal, bronchus, and lung cancer	1	1	N/A	1	0	1	1	1	1	0	1	1	N/A	N/A	N/A
Lee 1986	Case-control	Tracheal, bronchus, and lung cancer	0	1	N/A	1	0	1	1	0	0	0	1	1	N/A	N/A	N/A
Lee 2000	Case-control	Tracheal, bronchus, and lung cancer	0	1	N/A	1	0	1	1	0	0	0	0	1	N/A	N/A	N/A
Li 2002	Case-cohort	Tracheal, bronchus, and lung cancer	0	1	N/A	1	0	1	1	0	0	0	1	0	N/A	N/A	N/A

Table S20. Bias covariates tested in the primary model for each health outcome, by study

Author	Study design	Health outcome	Subpopulation	Not prosp. cohort	Out. self-reported	Exp. assessment	Potential selection bias	Adj. level 0	Adj. level 1	Adj. level 2	Not adj. smoking	Females only	Broad location exp.	Not current exp.	Asthma children	Hem. stroke	Isch. stroke
Li 2020	Prospective cohort	Tracheal, bronchus, and lung cancer	0	0	N/A	1	1	0	0	0	0	0	0	1	N/A	N/A	N/A
Liang 2009	Case-control	Tracheal, bronchus, and lung cancer	1	1	N/A	1	0	1	0	0	0	0	1	1	N/A	N/A	N/A
Liang 2019	Case-control	Tracheal, bronchus, and lung cancer	0	1	N/A	1	1	1	0	0	0	0	0	1	N/A	N/A	N/A
Liu 1991	Case-control	Tracheal, bronchus, and lung cancer	0	1	N/A	1	1	1	1	1	1	0	1	0	N/A	N/A	N/A
Masjedi 2013	Case-control	Tracheal, bronchus, and lung cancer	0	1	N/A	1	0	1	1	1	1	0	1	0	N/A	N/A	N/A
Mbeje 2022	Case-control	Tracheal, bronchus, and lung cancer	0	1	N/A	1	0	0	0	0	0	0	0	0	N/A	N/A	N/A
McGhee 2005	Case-control	Tracheal, bronchus, and lung cancer	0	1	N/A	1	0	1	1	0	0	0	1	1	N/A	N/A	N/A
Miller 2003	Case-control	Tracheal, bronchus, and lung cancer	0	1	N/A	1	0	1	1	0	0	0	1	0	N/A	N/A	N/A
Minichilli 2022	Case-control	Tracheal, bronchus, and lung cancer	0	1	N/A	1	1	1	1	1	1	0	1	0	N/A	N/A	N/A

Table S20. Bias covariates tested in the primary model for each health outcome, by study

Author	Study design	Health outcome	Subpopulation	Not prosp. cohort	Out. self-reported	Exp. assessment	Potential selection bias	Adj. level 0	Adj. level 1	Adj. level 2	Not adj. smoking	Females only	Broad location exp.	Not current exp.	Asthma children	Hem. stroke	Isch. stroke
Ng 2005	Case-control	Tracheal, bronchus, and lung cancer	0	1	N/A	1	1	1	1	0	0	0	1	0	N/A	N/A	N/A
Nishino 2001	Prospective cohort	Tracheal, bronchus, and lung cancer	0	0	N/A	1	0	1	1	0	0	0	1	0	N/A	N/A	N/A
Phukan 2014	Case-control	Tracheal, bronchus, and lung cancer	0	1	N/A	1	1	1	1	1	1	0	1	0	N/A	N/A	N/A
Pirie 2016	Prospective cohort	Tracheal, bronchus, and lung cancer	0	0	N/A	1	1	1	1	0	0	0	1	1	N/A	N/A	N/A
Rapiti 1999	Case-control	Tracheal, bronchus, and lung cancer	0	1	N/A	1	1	1	1	0	0	0	1	1	N/A	N/A	N/A
Raspanti 2016	Case-control	Tracheal, bronchus, and lung cancer	0	1	N/A	1	1	1	1	0	0	0	0	0	N/A	N/A	N/A
Ren 2013	Case-control	Tracheal, bronchus, and lung cancer	0	1	N/A	1	0	1	1	1	0	0	1	0	N/A	N/A	N/A
Robles 2014	Case-control	Tracheal, bronchus, and lung cancer	0	1	N/A	1	1	1	1	1	0	1	1	1	N/A	N/A	N/A
Rylander 2006	Case-control	Tracheal, bronchus, and lung cancer	0	1	N/A	1	1	1	1	1	0	1	1	1	N/A	N/A	N/A

Table S20. Bias covariates tested in the primary model for each health outcome, by study

Author	Study design	Health outcome	Subpop ulation	Not prosp. cohort	Out. self-reporte d	Exp. assessm ent	Potentia l selectio n bias	Adj. level 0	Adj. level 1	Adj. level 2	Not adj. smoking	Females only	Broad location exp.	Not current exp.	Asthma children	Hem. stroke	Isch. stroke
Sasco 2002	Case-control	Tracheal, bronchus, and lung cancer	0	1	N/A	1	0	1	1	0	0	0	0	1	N/A	N/A	N/A
Schwartz 2007	Case-control	Tracheal, bronchus, and lung cancer	0	1	N/A	1	1	1	1	1	1	0	0	1	N/A	N/A	N/A
Seki 2013	Case-control	Tracheal, bronchus, and lung cancer	0	1	N/A	1	0	1	0	0	0	0	1	1	N/A	N/A	N/A
Shen 1998	Case-control	Tracheal, bronchus, and lung cancer	0	1	N/A	1	1	1	1	0	0	0	1	1	N/A	N/A	N/A
Sloan 2012	Case-control	Tracheal, bronchus, and lung cancer	0	1	N/A	1	1	1	1	0	0	0	1	1	N/A	N/A	N/A
Sobue 1990	Case-control	Tracheal, bronchus, and lung cancer	0	1	N/A	1	0	1	1	0	0	0	1	1	N/A	N/A	N/A
Speizer 1999	Prospective cohort	Tracheal, bronchus, and lung cancer	0	0	N/A	1	0	1	1	0	0	0	1	1	N/A	N/A	N/A
Spitz 2011	Case-control	Tracheal, bronchus, and lung cancer	0	1	N/A	1	1	1	1	0	0	0	1	1	N/A	N/A	N/A
Stockwell 1992	Case-control	Tracheal, bronchus, and lung cancer	1	1	N/A	1	0	1	1	0	0	0	1	1	N/A	N/A	N/A

Table S20. Bias covariates tested in the primary model for each health outcome, by study

Author	Study design	Health outcome	Subpopulation	Not prosp. cohort	Out. self-reported	Exp. assessment	Potential selection bias	Adj. level 0	Adj. level 1	Adj. level 2	Not adj. smoking	Females only	Broad location exp.	Not current exp.	Asthma children	Hem. stroke	Isch. stroke
Sun 2017	Prospective cohort	Tracheal, bronchus, and lung cancer	1	0	N/A	1	0	0	0	0	0	0	1	1	N/A	N/A	N/A
Svensson 1989	Case-control	Tracheal, bronchus, and lung cancer	0	1	N/A	1	1	1	1	0	0	0	0	1	N/A	N/A	N/A
Torres-Durán 2015	Case-control	Tracheal, bronchus, and lung cancer	0	1	N/A	1	1	1	1	0	0	0	1	1	N/A	N/A	N/A
Tubío-Pérez 2022	Case-control	Tracheal, bronchus, and lung cancer	0	1	N/A	1	1	1	1	0	0	0	1	1	N/A	N/A	N/A
Veglia 2007	Prospective cohort	Tracheal, bronchus, and lung cancer	0	0	N/A	1	1	1	1	1	0	0	1	0	N/A	N/A	N/A
Villeneuve 2014	Case-control	Tracheal, bronchus, and lung cancer	0	1	N/A	1	1	1	1	1	1	1	0	0	N/A	N/A	N/A
Wang 1994	Case-control	Tracheal, bronchus, and lung cancer	0	1	N/A	1	1	1	1	0	0	0	1	1	N/A	N/A	N/A
Wang 1996	Case-control	Tracheal, bronchus, and lung cancer	0	1	N/A	1	1	1	0	0	0	0	0	0	N/A	N/A	N/A
Wang 1996	Case-control	Tracheal, bronchus, and lung cancer	0	1	N/A	1	1	1	1	0	0	0	1	1	N/A	N/A	N/A

Table S20. Bias covariates tested in the primary model for each health outcome, by study

Author	Study design	Health outcome	Subpopulation	Not prosp. cohort	Out. self-reported	Exp. assessment	Potential selection bias	Adj. level 0	Adj. level 1	Adj. level 2	Not adj. smoking	Females only	Broad location exp.	Not current exp.	Asthma children	Hem. stroke	Isch. stroke
Wang 2009	Case-control	Tracheal, bronchus, and lung cancer	0	1	N/A	1	1	1	0	0	0	0	0	1	N/A	N/A	N/A
Wang 2015	Prospective cohort	Tracheal, bronchus, and lung cancer	0	0	N/A	1	0	0	0	0	0	0	0	1	N/A	N/A	N/A
Weiss 2008	Prospective cohort	Tracheal, bronchus, and lung cancer	0	0	N/A	1	0	1	1	1	0	0	0	1	N/A	N/A	N/A
Wen 2006	Prospective cohort	Tracheal, bronchus, and lung cancer	1	0	N/A	0	0	0	0	0	0	0	1	0	N/A	N/A	N/A
Wenzlaff 2005	Case-control	Tracheal, bronchus, and lung cancer	0	1	N/A	1	1	1	1	0	0	0	1	1	N/A	N/A	N/A
Wu 1985	Case-control	Tracheal, bronchus, and lung cancer	0	1	N/A	1	1	1	1	0	0	0	0	1	N/A	N/A	N/A
Xu 2020	Case-control	Tracheal, bronchus, and lung cancer	0	1	N/A	1	0	1	1	1	1	0	1	0	N/A	N/A	N/A
Yang 2015	Case-control	Tracheal, bronchus, and lung cancer	0	1	N/A	1	1	1	0	0	0	0	1	1	N/A	N/A	N/A
Yang 2016	Case-control	Tracheal, bronchus, and lung cancer	0	1	N/A	1	0	1	1	0	0	0	1	0	N/A	N/A	N/A

Table S20. Bias covariates tested in the primary model for each health outcome, by study

Author	Study design	Health outcome	Subpopulation	Not prosp. cohort	Out. self-reported	Exp. assessment	Potential selection bias	Adj. level 0	Adj. level 1	Adj. level 2	Not adj. smoking	Females only	Broad location exp.	Not current exp.	Asthma children	Hem. stroke	Isch. stroke
Yin 2014	Case-control	Tracheal, bronchus, and lung cancer	0	1	N/A	1	1	1	1	0	0	0	1	0	N/A	N/A	N/A
Yoon 2008	Case-control	Tracheal, bronchus, and lung cancer	0	1	N/A	1	0	1	1	0	0	0	0	0	N/A	N/A	N/A
Zaridze 1998	Case-control	Tracheal, bronchus, and lung cancer	0	1	N/A	1	0	1	1	0	0	0	0	1	N/A	N/A	N/A
Zatloukal 2003	Case-control	Tracheal, bronchus, and lung cancer	0	1	N/A	1	1	1	1	1	0	0	1	1	N/A	N/A	N/A
Zhong 1999	Case-control	Tracheal, bronchus, and lung cancer	1	1	N/A	1	0	1	0	0	0	0	0	1	N/A	N/A	N/A
Zhuang 2022	Case-control	Tracheal, bronchus, and lung cancer	1	1	N/A	1	1	0	0	0	0	0	1	0	N/A	N/A	N/A

N/A, not available

Section 6: Between-study Heterogeneity

The gamma solution (gamma) quantifies the degree of heterogeneity observed in the included observations, while its standard deviation (SD) quantifies the uncertainty around gamma, both of which contribute to our final conservative BPRF and RR uncertainty estimates. Across the GBD, within all dichotomous risk factors evaluated using the BPRF methodology, gamma solution values range from 0 to 0.59 with a mean of 0.07 (SD: 0.15).

Table S21. Between-study heterogeneity (gamma) for each risk-outcome pair

Health outcome	Gamma solution
Ischemic heart disease	0.002
Stroke	0.000
Diabetes mellitus type 2	0.000
Lower respiratory infections	0.033
Breast cancer	0.035
Tracheal, bronchus, and lung cancer	0.018
Otitis media	0.004
Asthma	0.015
Chronic obstructive pulmonary disease	0.060
